# Raw and processed microscope images of fixed cells at baseline and following various experimental perturbations

**DOI:** 10.1016/j.dib.2016.01.044

**Published:** 2016-01-29

**Authors:** P. Mason McClatchey, Amy C. Keller, Ron Bouchard, Leslie A. Knaub, Jane E.B. Reusch

**Affiliations:** a Division of Endocrinology, University of Colorado Anschutz Medical Campus, Aurora, CO, USA; b Department of Medicine, Denver VA Medical Center, Denver, CO, USA; c Department of Bioengineering, University of Colorado Anschutz Medical Campus, Aurora, CO, USA; d Center for Women’s Health Research, University of Colorado School of Medicine, Aurora, CO, USA

## Abstract

The data included in this article comprise raw and processed images of fixed cells at baseline and subjected to various experimental perturbations. This dataset includes images of HUVEC cells fixed and subsequently incubated at either 37 °C or room temperature, primary rat vascular smooth muscle cells exposed to 25 mM glucose, and SH-SY5Y neurons exposed to hydrogen peroxide. Raw images appear exactly as they were captured on the microscope, while processed images show the binarization provided by software used for measurements of mitochondrial morphology. For in-depth discussion of the experiments and computational methods pertaining to this data, please refer to the corresponding research article titled “Fully automated software for quantitative measurements of mitochondrial morphology” (McClatchey et al., in press) [1].

## Specifications Table

**Table:** 

Subject area	*Biology*
More specific subject area	*Mitochondrial dynamics*
Type of data	*Images*
How data was acquired	*Microscopy and image processing*
Data format	*PNG*
Experimental factors	*HUVEC cells, primary SMCs from Wistar and GK rats, and SH-SY5Y neurons cultured for 6–8 passages in vitro.*
Experimental features	*Fixed cells stained for Tom20 (mitochondria), nitrotyrosine (cytoplasm), and DAPI (nuclei) and binarized using computational methods*[1], [2], [3]
Data source location	*Aurora, Colorado, United States of America*
Data accessibility	*Data is within this article.*

## Value of the data

• Investigators considering use of this mitochondrial morphology measurement technique can refer to these images for assessment of software quality.
• Readers to whom the endpoints reported in the associated primary research article [1] are interesting can use these images as a visual reference.
• Investigators considering use of this mitochondrial morphology measurement technique can compare their images to these to assess whether this software is applicable.

## 1 Data

The images shown here consist of raw microscope images (left) and software-binarized images (right) acquired or generated for individual cell culture experiments in the associated primary research article [1]. The first image acquired for each treatment group is shown; please refer to the Supplementary materials for the full set of raw microscope images. The information included above (i.e. specifications table, value of the data, data description in this paragraph) applies to all image sets below. Experimental design, materials and methods are included in both general and experiment-specific terms.

## 2 Experimental design, materials and methods

### 2.1 Reagents

Dulbecco׳s Modified Eagles Medium (DMEM) 5 mM and 25 mM glucose and non-essential amino acids and Laughlin’s F12 Medium were obtained from Thermo Scientific Hyclone, and trypsin and trypsin inhibitor were purchased from Fisher Scientific. Fetal bovine serum (FBS) was procured from Gemini Bioproducts. Hank׳s Balanced Salt Solution (HBSS) was purchased from Corning Life Sciences. Secondary detection antibodies Alex Fluor 488 and 546 were purchased from Life Technologies. Antibodies to TOM20 (rabbit) and nitrotyrosine (mouse) were procured from Santa Cruz Biotechnology.

### 2.2 Cell culture

Primary rat vascular smooth muscle cells (SMCs) were cultured in low glucose (5 mM) DMEM with 10% fetal bovine serum (FBS), 1% L-glutamine, 1% non-essential amino acid blend, and 1% Pen/Strep, all expressed as % by volume. For SMC starvation media, the same proportions were maintained, except that FBS was reduced to 0.1%. Human umbilical vein endothelial cells (HUVECs) were cultured in F-12k medium (Hyclone #SH30526.01) with 10% FBS, 1% Pen/Strep, 0.05 mg/mL endothelial cell growth supplement, and 0.1 mg/mL heparin. SH-SY5Ys (ATTC neuronal cell line) were cultured in 45% F-12k medium, 45% low glucose DMEM, and 10% FBS. All cells were cultured at 37 °C at 5% CO_2_. Media was changed at least every three days, and cells were split 2:1 at 80% confluence. Experiments were performed on cells at 50–70% confluence. Unless otherwise specified, cell culture materials were obtained from Santa Cruz Biotechnology.

### 2.3 Fixation and staining

Prior to fixation, phosphate buffer solution (PBS) rinse and paraformaldehyde solutions were warmed to 37 °C (except as otherwise indicated). Samples were washed 3× in warm PBS prior to incubation in 4% paraformaldehyde at 37 °C for 15 min. Following fixation, samples were washed 3× in warm PBS and quenched in 50 mM NH_4_Cl for 15 min at room temperature. Samples were then rinsed 3× with room temperature PBS and stored immersed in PBS at 4 °C until staining. All samples were stained within one week of fixation. Prior to staining, cells were permeablized by incubation in 0.1% TX-1000 at 37 °C for 15 min. Following permeabilization, samples were rinsed 3× with room temperature PBS and then blocked by incubation in 10% fetal bovine serum (FBS) in PBS for 25 min and 37 °C. Samples were subsequently incubated in a primary antibody mix in 5% FBS in PBS at 37 °C for 1 h. Following treatment with primary antibody, samples were washed 3× with 5% FBS in PBS and incubated in a secondary antibody mix in 5% FBS in PBS at room temperature for 1 h. Samples were then washed 3× in PBS, incubated in DAPI diluted 1:10,000 in PBS at room temperature for 10 min, washed 3× in PBS again, then washed once in distilled water. Coverslips were then mounted to microscope slides for imaging. The primary antibody mix consisted of a Tom20 antibody (Santa Cruz Biotechnology sc-11415) diluted 400× to stain mitochondria and a nitrotyrosine antibody (Santa Cruz Biotechnology sc-32757) diluted 100× to stain the cytoplasm. The secondary antibody mix consisted of Alexafluor 488 (Life Technologies A-11008) diluted 1000× to react with the Tom20 antibody and Alexafluor 546 (Life Technologies A-11030) diluted 500× to react with the nitrotyrosine antibody. Following staining, slides were stored protected from light at 4 °C.

### 2.4 Microscopy and imaging techniques

The microscope used in these experiments was an Olympus FV1000 confocal microscope provided by the UC Denver Advanced Light Microscopy Core. A 488 nm laser was used to excite AlexaFluor 488 and a 543 nm laser was used to excite AlexaFluor 546. Built-in filter settings for these fluorophores were used to detect fluorescence. Slides were imaged using a 60× oil immersion objective at a resolution of 206.8 nm/pixel and an image size of 1024 pixels×1024 pixels. An exposure time of 15 ms per pixel was used and the average of three frame acquisitions was used as the raw microscope image.

### 2.5 Image processing

A custom Matlab script was used in the corresponding primary research article [1] to analyze all raw microscope images and perform fluorescent bead-based calibrations. Code and instructions for use are in Supplementary material. Please contact the study authors if any assistance is required.

*Note*: All information above applies to all image sets below. Experiment-specific details are included for each image set below.

## Experiment 1: baseline cell line characteristics

**Fig. 1:**
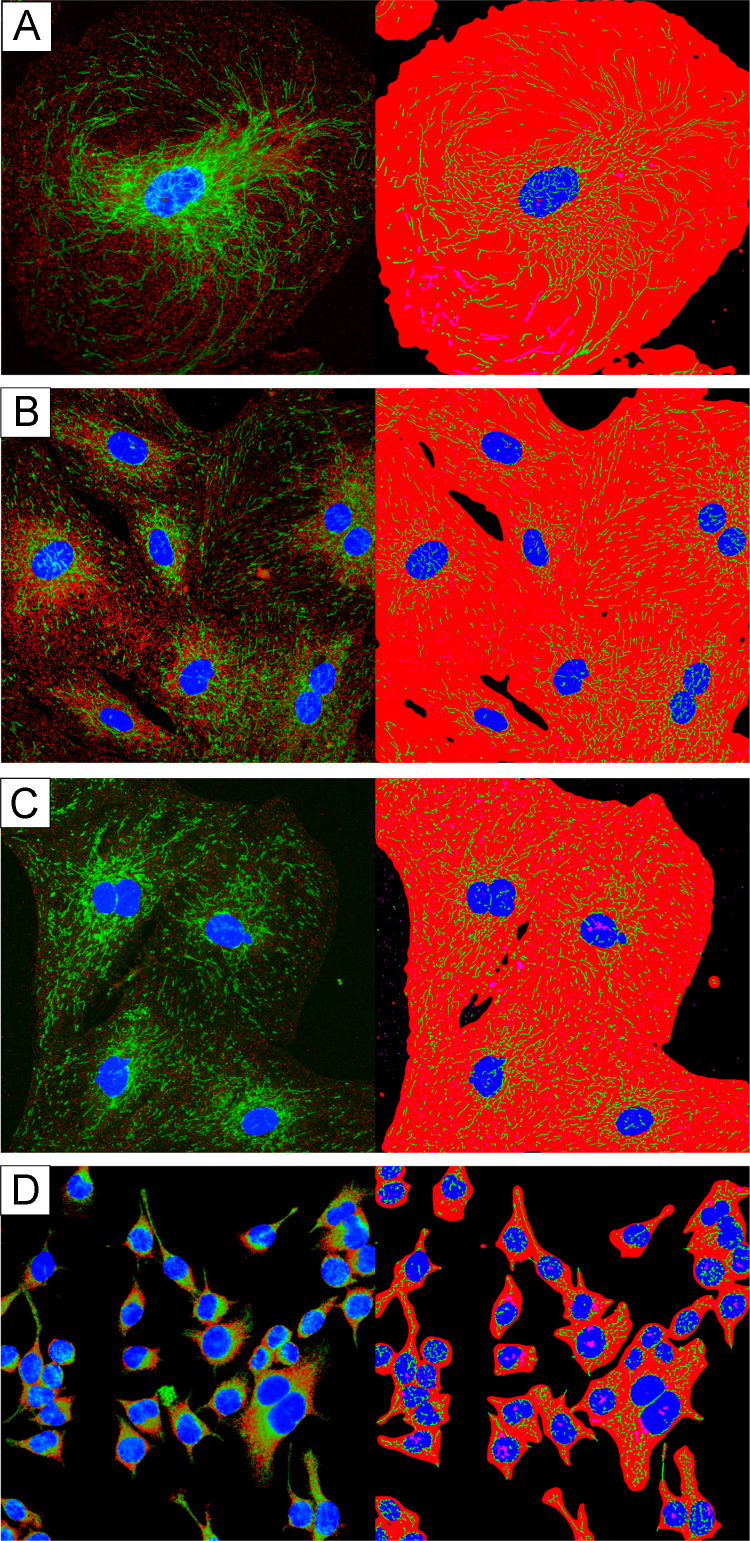
Baseline images of cell lines used in this study. The first raw microscope image (right) and software binarized image (left) from each group. (A) Human umbilical vein endothelial cells (HUVECs). (B) Goto-Kakizaki rat aortic primary smooth muscle cells (GK SMCs). (C) Wistar rat aortic primary smooth muscle cells (Wistar SMCs). (D) SH-SY5Y neurons.

This experiment investigated baseline mitochondrial network characteristics of GK vSMCs, Wistar vSMCs, HUVECs, and SH-SY5Ys. See Fig. 1.

## Experiment 2: effects of fixation temperature in HUVEC cells

**Fig. 2:**
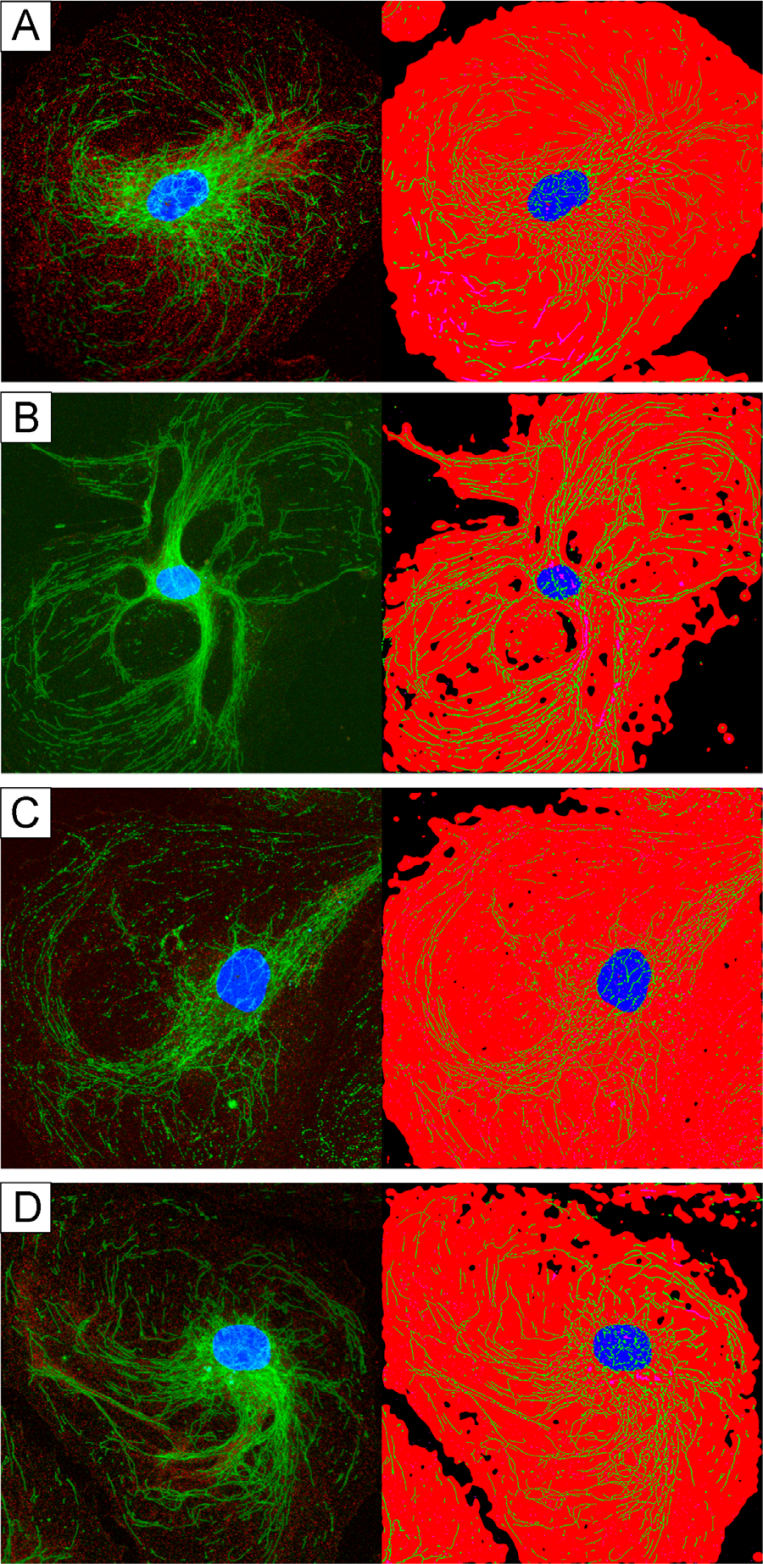
HUVEC endothelial cells fixed at various temperatures. The first raw microscope image (right) and software binarized image (left) from each group. (A) Fixed with reagents pre-warmed to 37 °C and subsequently incubated at 37 °C. (B) Fixed with reagents pre-warmed to 37 °C and subsequently incubated at room temperature. (C) Fixed with room temperature reagents and subsequently incubated at room temperature. (D) Fixed with room temperature reagents and subsequently incubated at 37 °C.

HUVEC cells were cultured without experimental perturbation for 6–10 passages. Cells were divided into one of four experimental groups and fixed according to the protocol described above. Group 1 was fixed using reagents pre-heated to 37 °C and all incubation steps during fixation were performed at 37 °C; Group 2 was fixed using room-temperature reagents and all incubation steps during fixation were performed at 37 °C; Group 3 was fixed using room-temperature reagents and all incubation steps during fixation were performed at room temperature; Group 4 was fixed using reagents pre-heated to 37 °C and all incubation steps during fixation were performed at room temperature. See Fig. 2.

## Experiment 3: high glucose exposure with primary rat SMCs

**Fig. 3:**
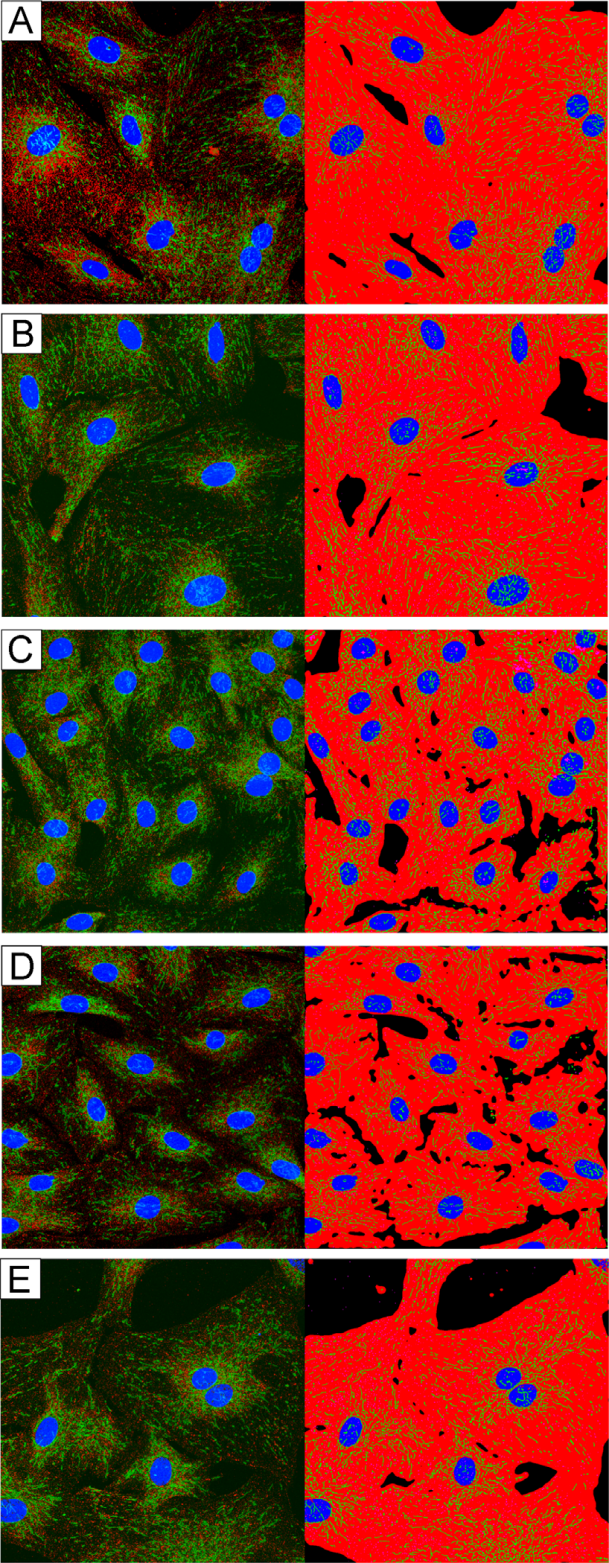
High-glucose exposure time course in GK SMCs. The first raw microscope image (right) and software binarized image (left) from each group. (A) 0 min exposure to 25 mM glucose. (B) 10 min exposure to 25 mM glucose. (C) 30 min exposure to 25 mM glucose. (D) 60 min exposure to 25 mM glucose. (E) 240 min exposure to 25 mM glucose.

Primary smooth muscle cells from the aorta of GK or Wistar rats were cultured in low glucose (5 mM) smooth muscle growth media until passages 8–10. Prior to treatment, cells were switched to low glucose, serum free starvation media for 48 h. Serum starved cells were exposed to high glucose (25 mM), serum free media. For each cell type (GK and Wistar) in each repetition of each experiment, 15 slides were prepared. Of these 15 slides, three were merely rinsed with high glucose media before fixation and staining (0 min exposure), three were fixed and stained after 10 min in high glucose media, three were fixed and stained after 30 min in high glucose media, three were fixed and stained after 30 min in high glucose media, three were fixed and stained after 60 min in high glucose media, and the remaining three were fixed and stained after 240 min in high glucose media. See Figs. 3 (GK) and 4 (Wistar).

## Experiment 4: hydrogen peroxide exposure with SH-SY5Y neurons

**Fig. 5:**
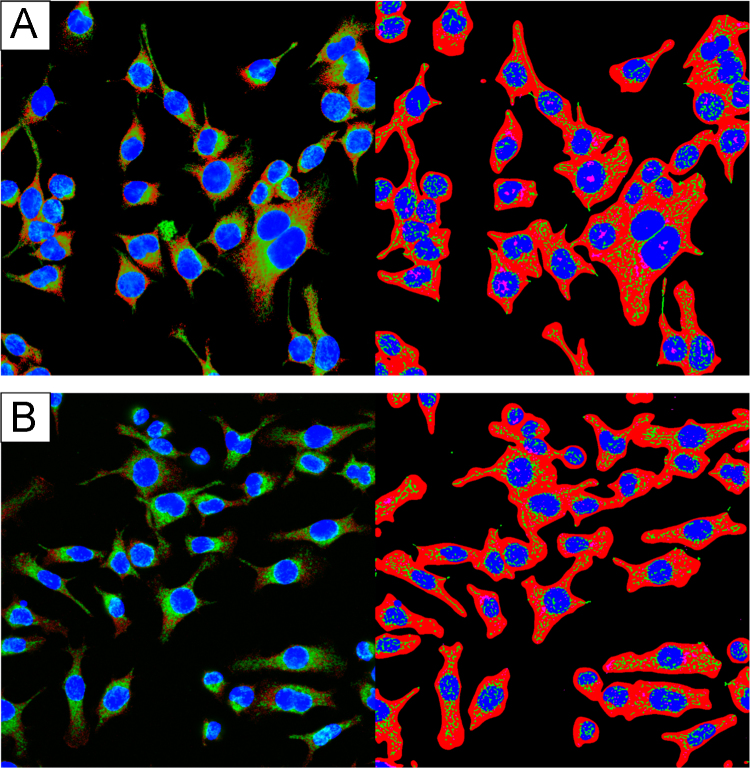
Hydrogen peroxide exposure in SH-SY5Y neurons. The first raw microscope image (right) and software binarized image (left) from each group. (A) Neurons not exposed to hydrogen peroxide. (B) Neurons exposed to 200 nM hydrogen peroxide for 2 h.

SH-SY5Y neuronal cells were cultured without experimental perturbation for a minimum of 5 passages. Cells were then changed to media containing either 200 nM or 0 nM H_2_O_2_ for 2 h at least 24 h after the previous media change. Cells were then fixed and stained as described above. See Fig. 5.

## Sources of funding

Sources of funding for this study were provided by VA Merit, Denver Research Institute, NIH-5T32HL007171 (ACK), NIH-5P01HL014985 (ACK), CCTSI-UL1RR025780 (JEBR), the Center for Women’s Health Research (JEBR), and the Department of Bioengineering (PMM). Flow Cytometry is supported through a National Cancer Institute Cancer Center Support Grant (P30CA046934).

## Disclosures

There is no conflict of interest related to this work for P.M.M., A.C.K., R.B., L.A.K., or J.E.B.R.

## Figures and Tables

**Fig. 4:**
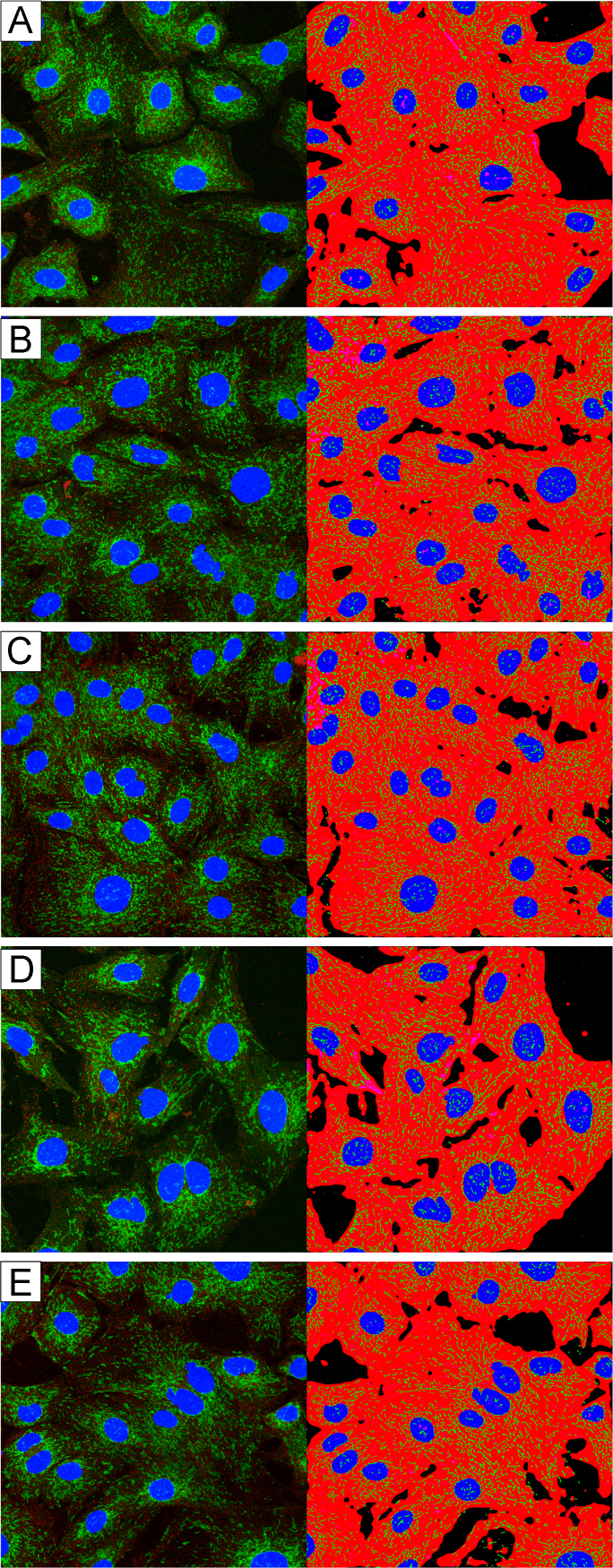
High-glucose exposure time course in Wistar SMCs. The first raw microscope image (right) and software binarized image (left) from each group. (A) 0 min exposure to 25 mM glucose. (B) 10 min exposure to 25 mM glucose. (C) 30 min exposure to 25 mM glucose. (D) 60 min exposure to 25 mM glucose. (E) 240 min exposure to 25 mM glucose.

## Footnotes

Appendix A Supplementary data associated with this article can be found in the online version at doi:10.1016/j.dib.2016.01.044.
