# Supplementary figures and images for: Raw and processed microscope images of fixed cells at baseline and following various experimental perturbations (part 2 of 4)
Source: Data Brief. 2016 Jan 29;6:998–1006. doi: 10.1016/j.dib.2016.01.044 (PMC4760184; doi:10.1016/j.dib.2016.01.044)

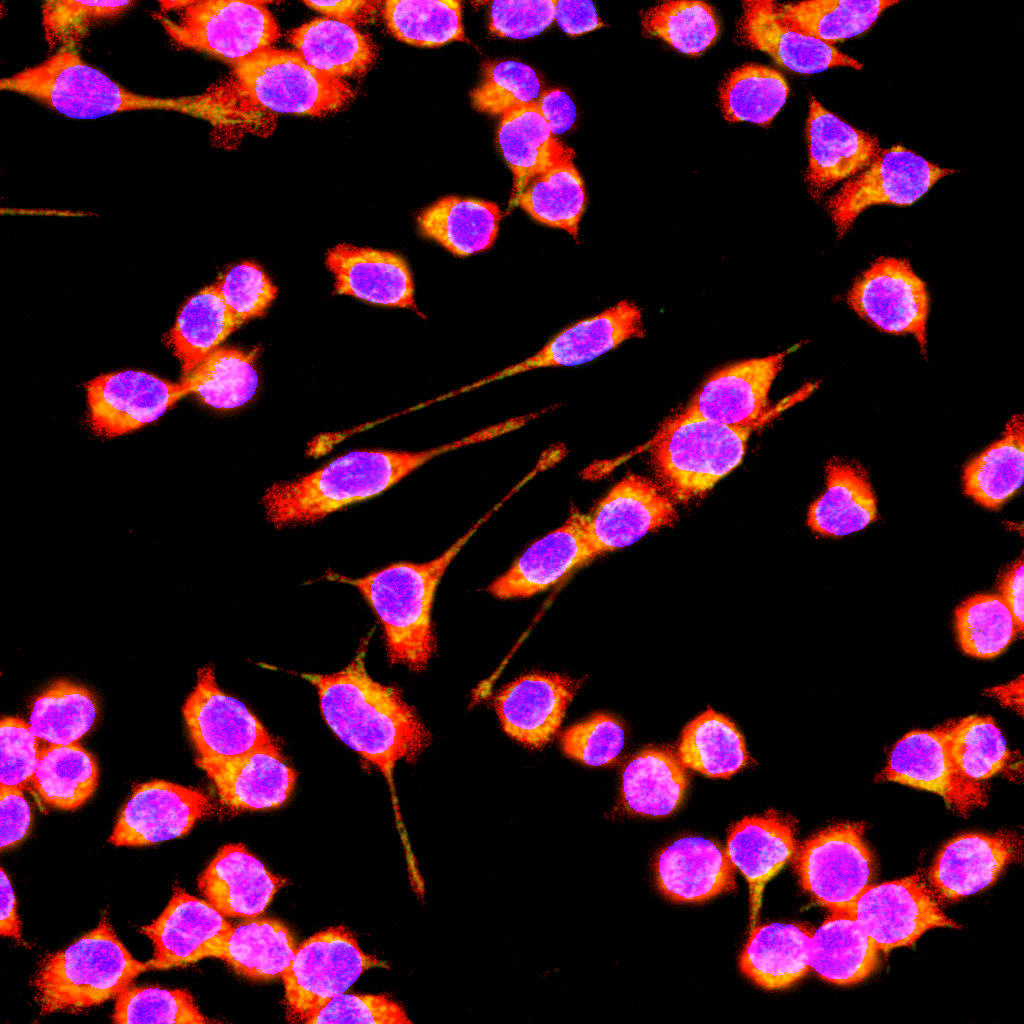

Supplement: Supplementary file 4 — Supplementary material [file mmc4.zip › Cell Types - 3rd Experiment/E3-G4-2.tif]

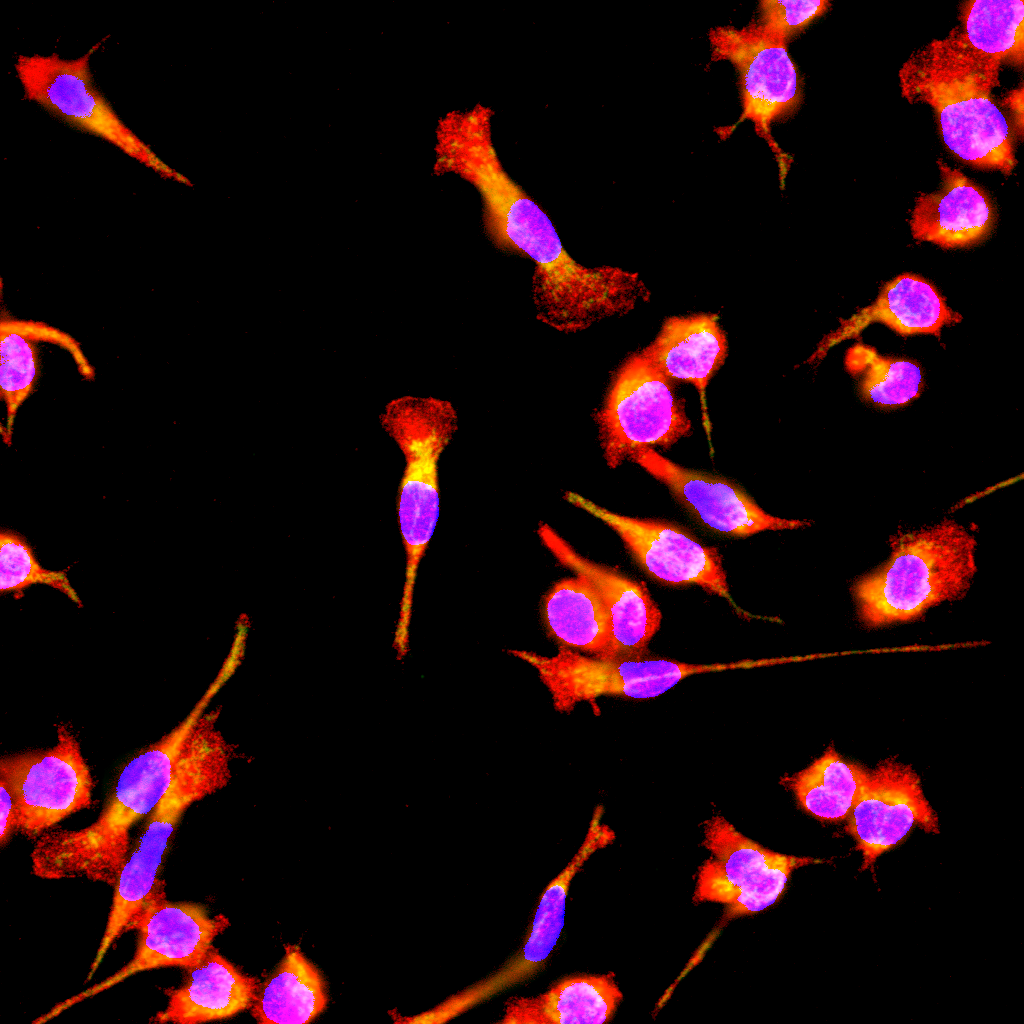

Supplement: Supplementary file 4 — Supplementary material [file mmc4.zip › Cell Types - 3rd Experiment/E3-G4-3.tif]

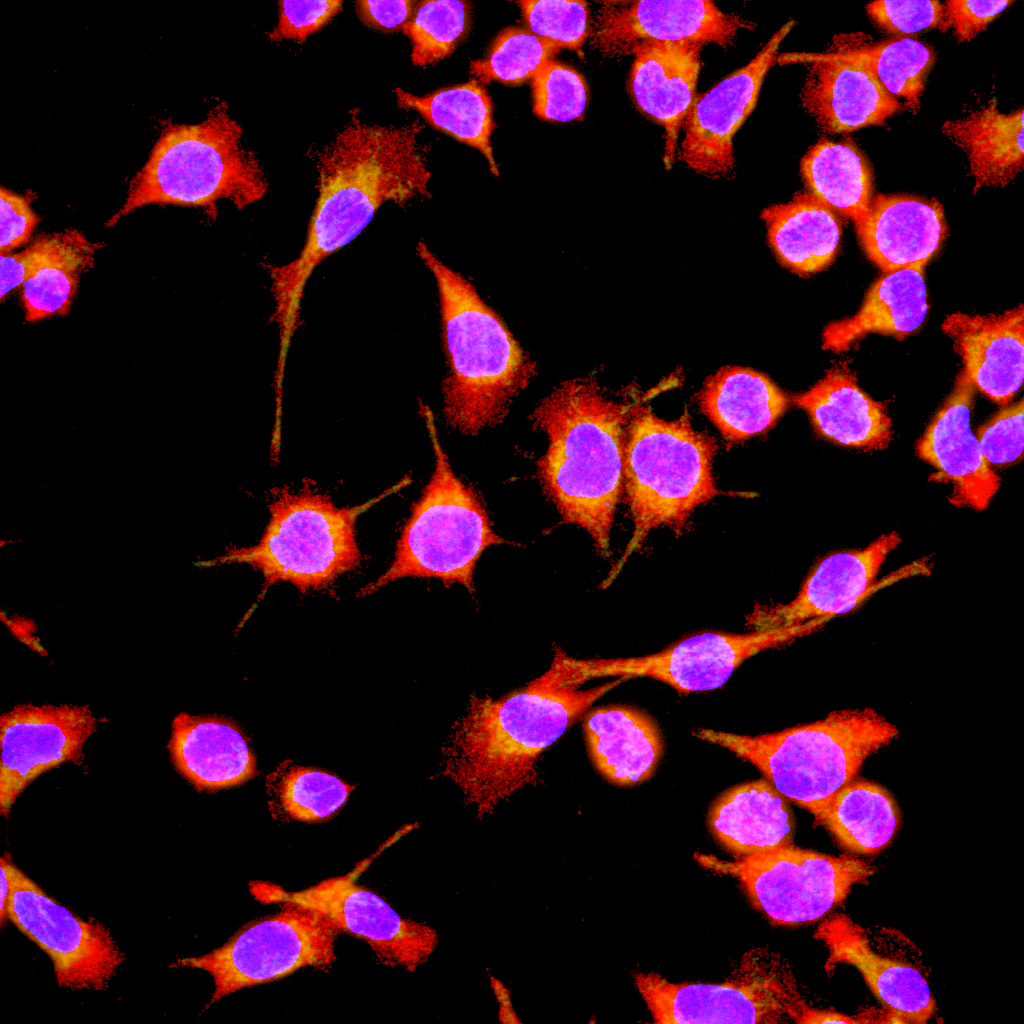

Supplement: Supplementary file 4 — Supplementary material [file mmc4.zip › Cell Types - 3rd Experiment/E3-G4-4.tif]

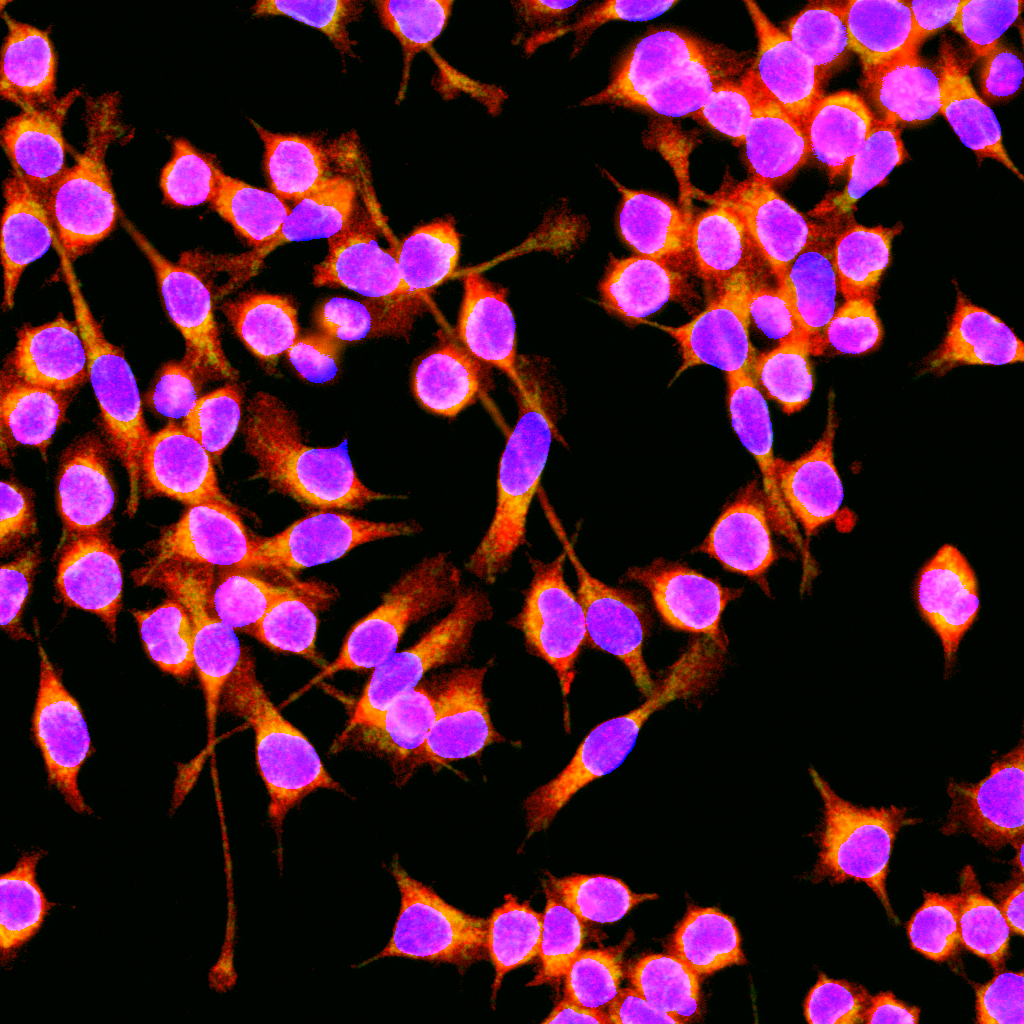

Supplement: Supplementary file 4 — Supplementary material [file mmc4.zip › Cell Types - 3rd Experiment/E3-G4-5.tif]

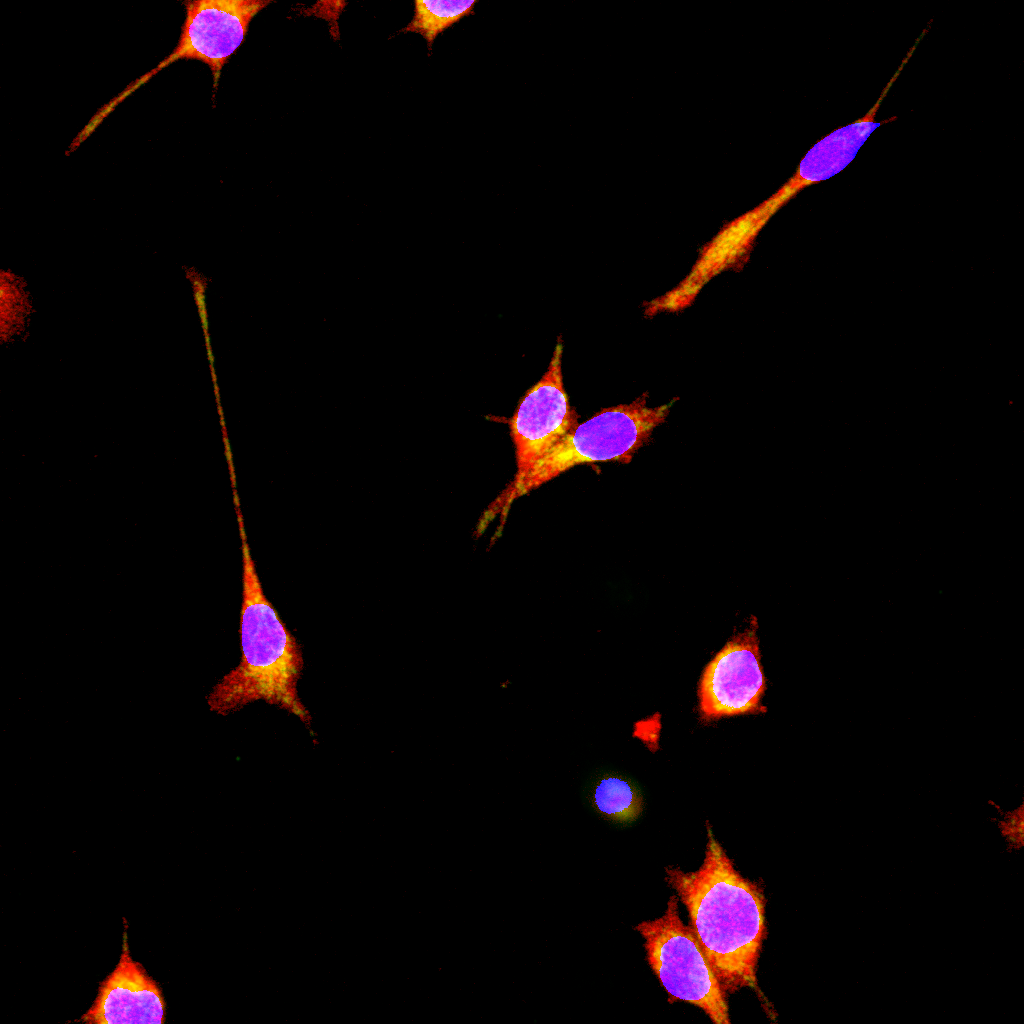

Supplement: Supplementary file 4 — Supplementary material [file mmc4.zip › Cell Types - 3rd Experiment/E3-G4-6.tif]

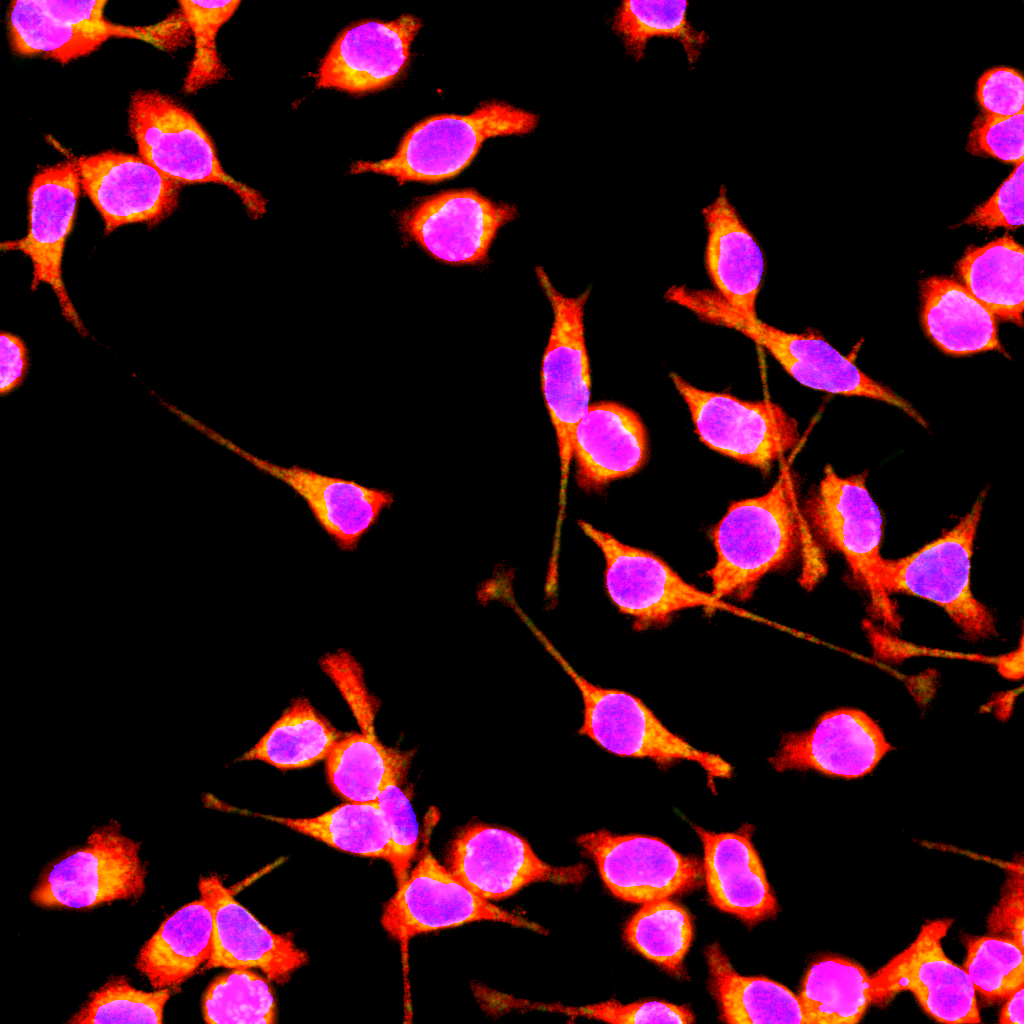

Supplement: Supplementary file 4 — Supplementary material [file mmc4.zip › Cell Types - 3rd Experiment/E3-G4-7.tif]

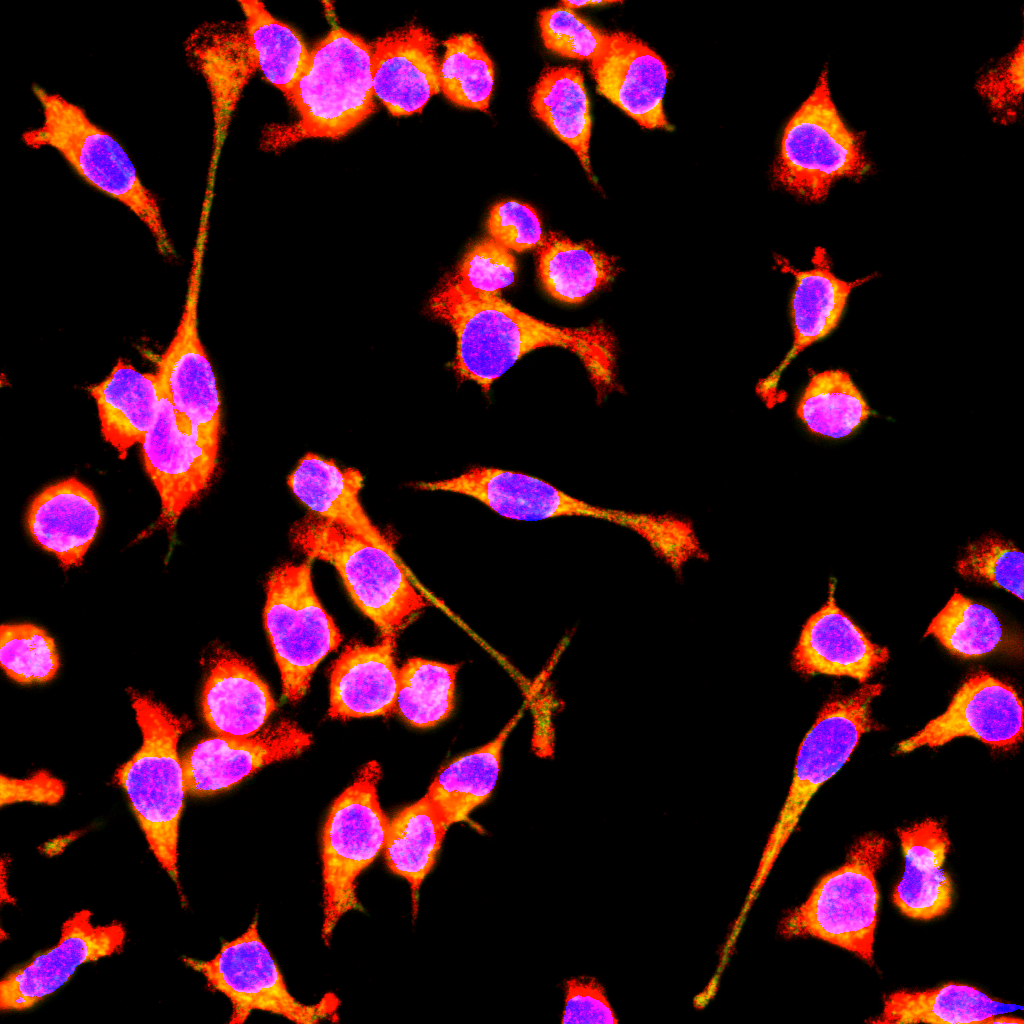

Supplement: Supplementary file 4 — Supplementary material [file mmc4.zip › Cell Types - 3rd Experiment/E3-G4-8.tif]

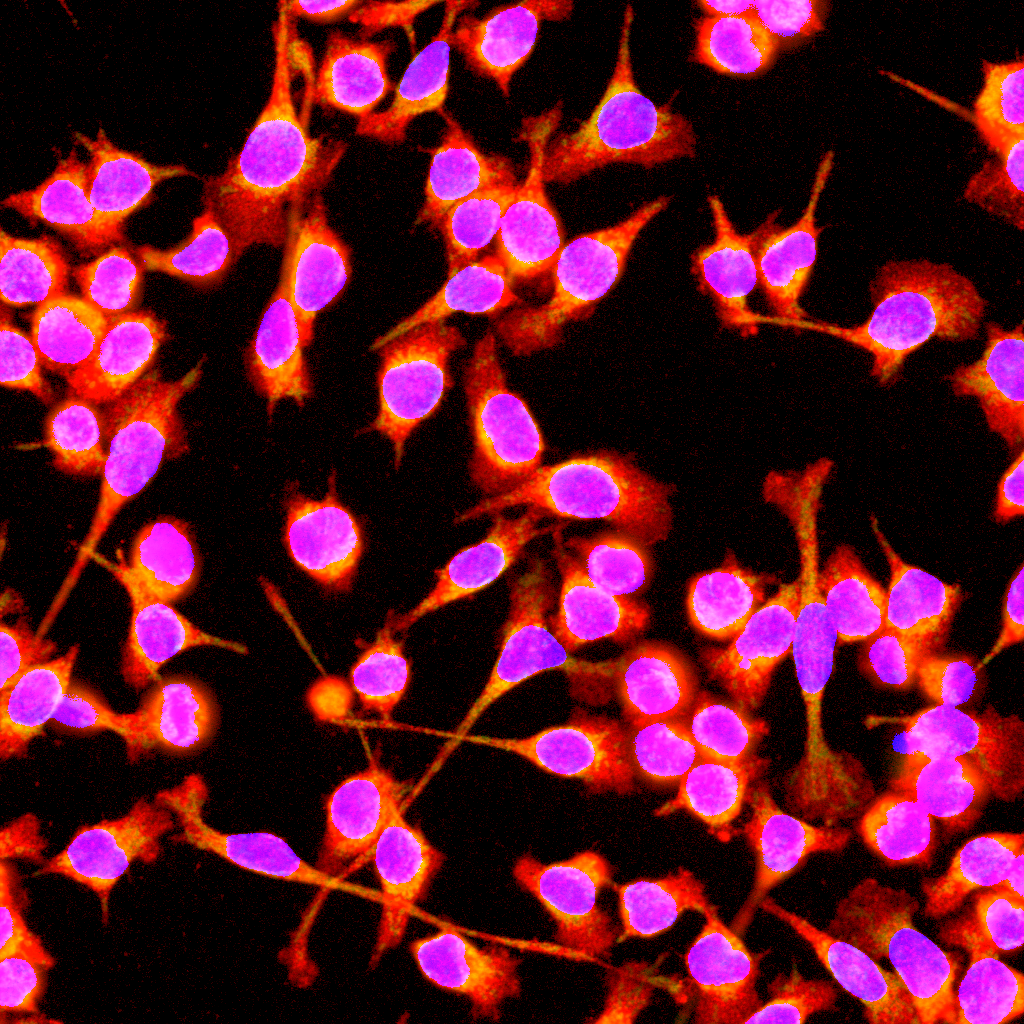

Supplement: Supplementary file 4 — Supplementary material [file mmc4.zip › Cell Types - 3rd Experiment/E3-G4-9.tif]

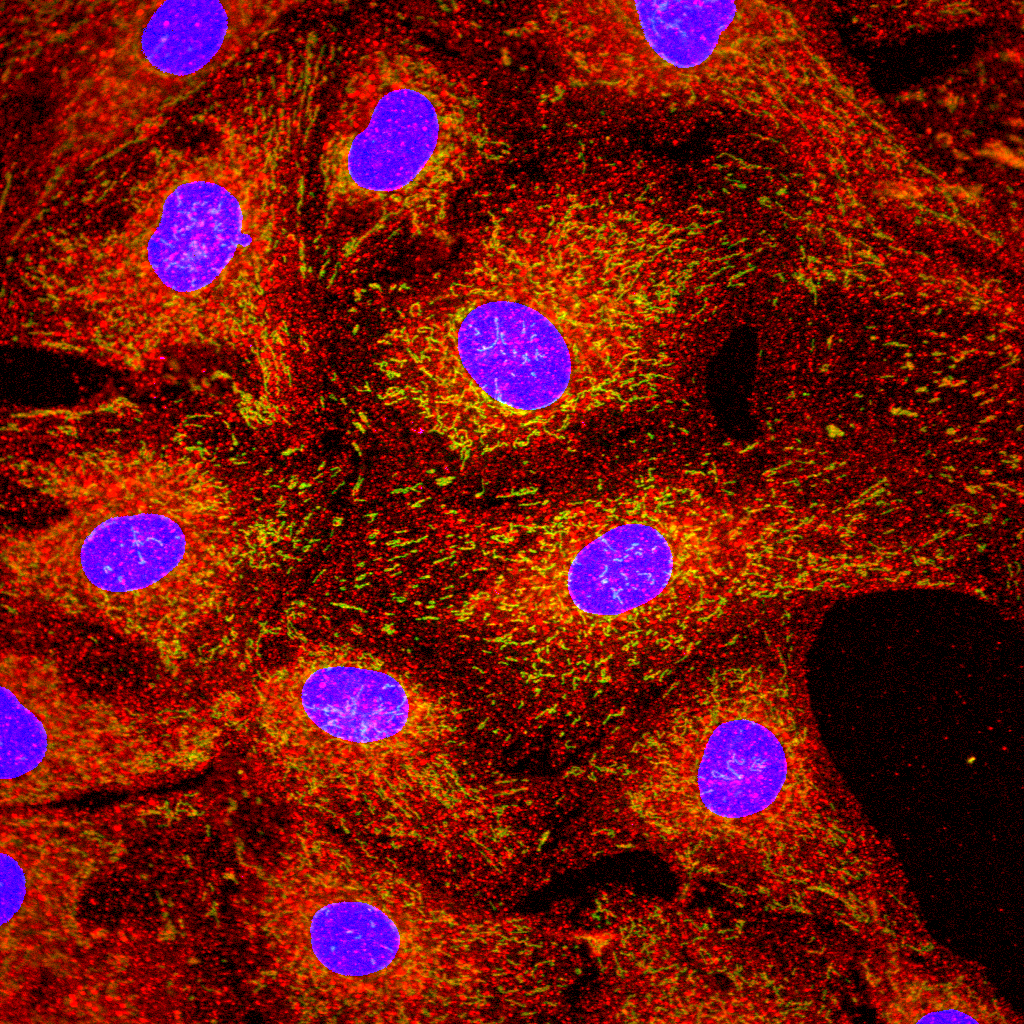

Supplement: Supplementary file 5 — Supplementary material [file mmc5.zip › GK Glucose Time Course - 1st Experiment/E1-G2-1.tif]

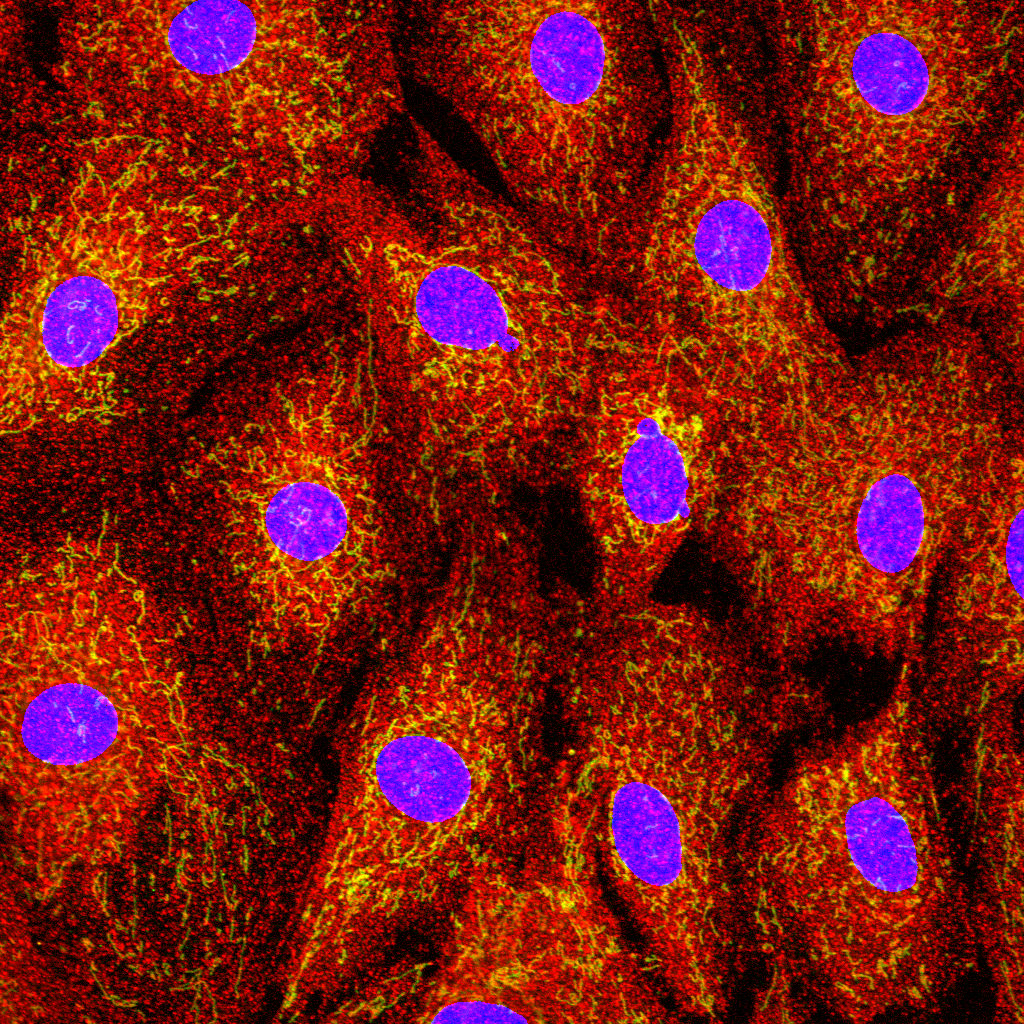

Supplement: Supplementary file 5 — Supplementary material [file mmc5.zip › GK Glucose Time Course - 1st Experiment/E1-G2-2.tif]

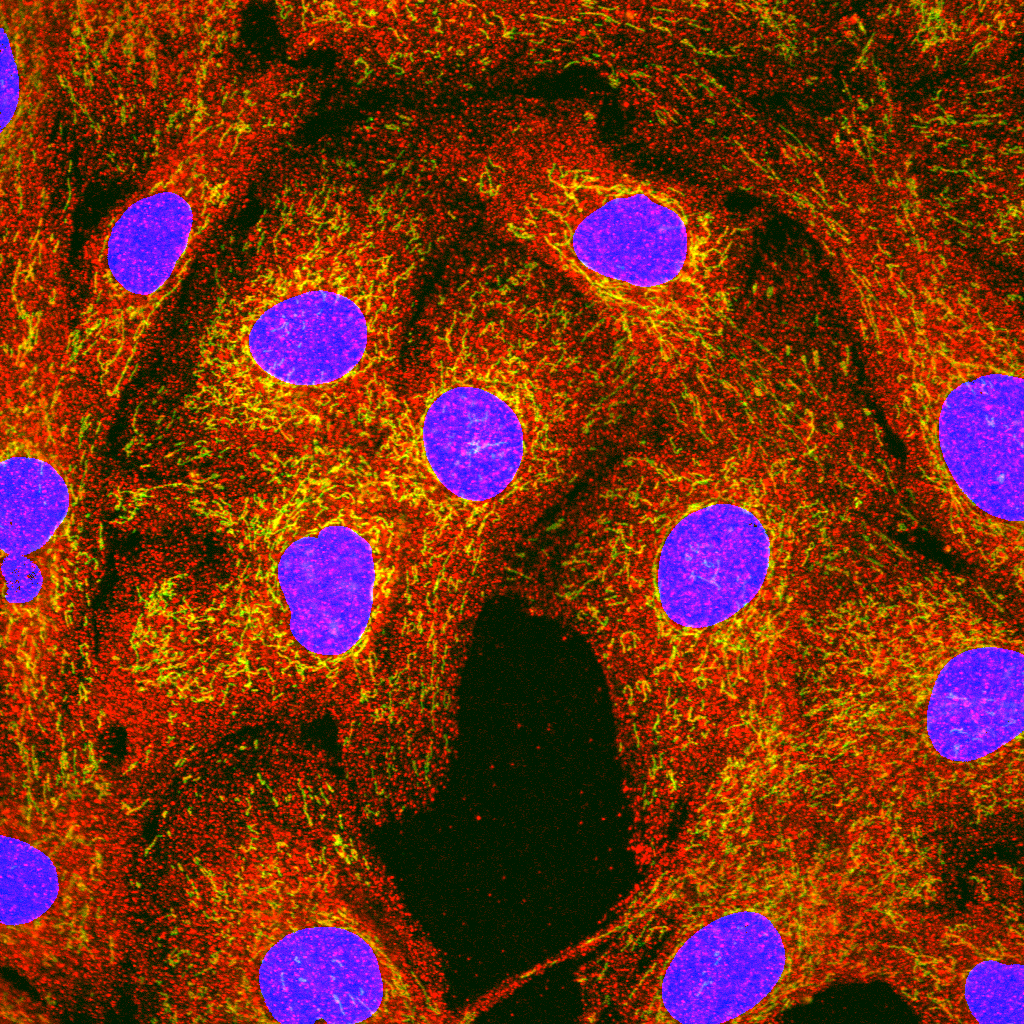

Supplement: Supplementary file 5 — Supplementary material [file mmc5.zip › GK Glucose Time Course - 1st Experiment/E1-G2-3.tif]

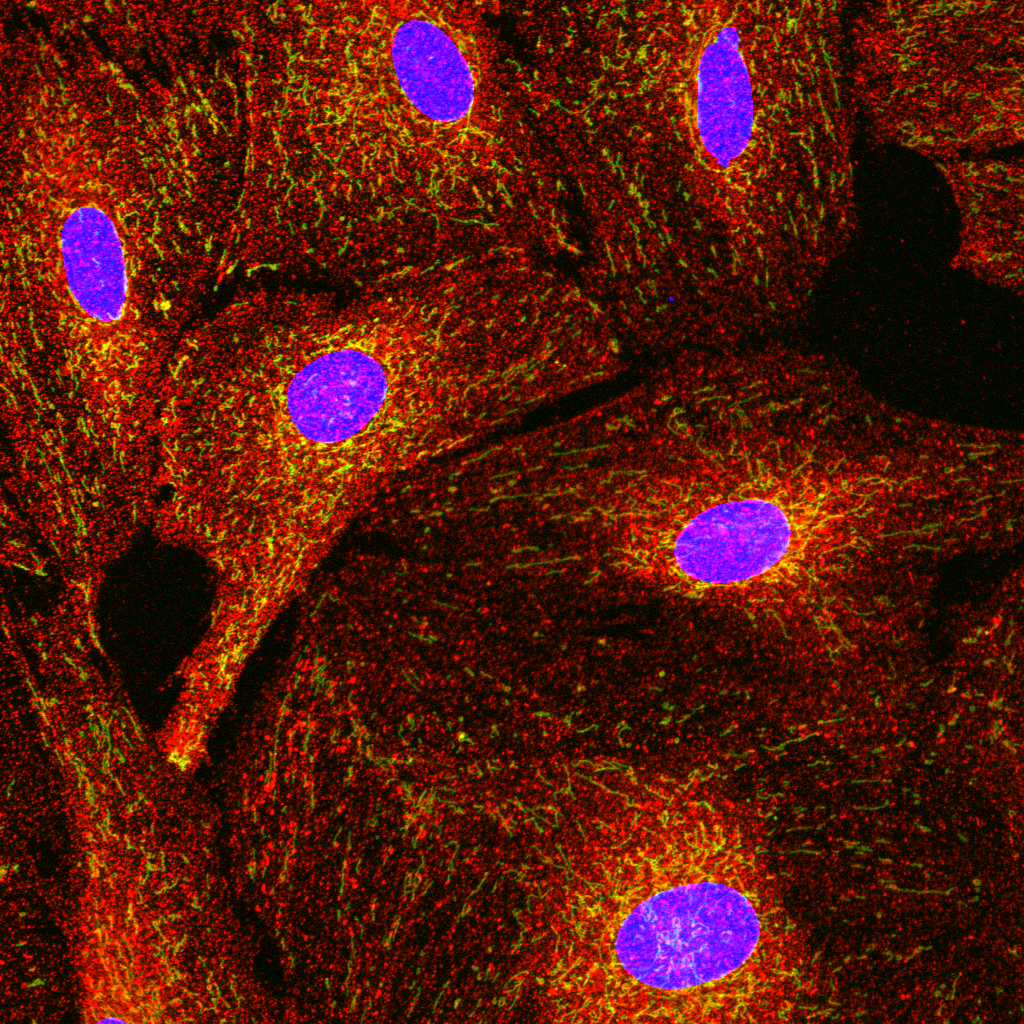

Supplement: Supplementary file 5 — Supplementary material [file mmc5.zip › GK Glucose Time Course - 1st Experiment/E1-G2-4.tif]

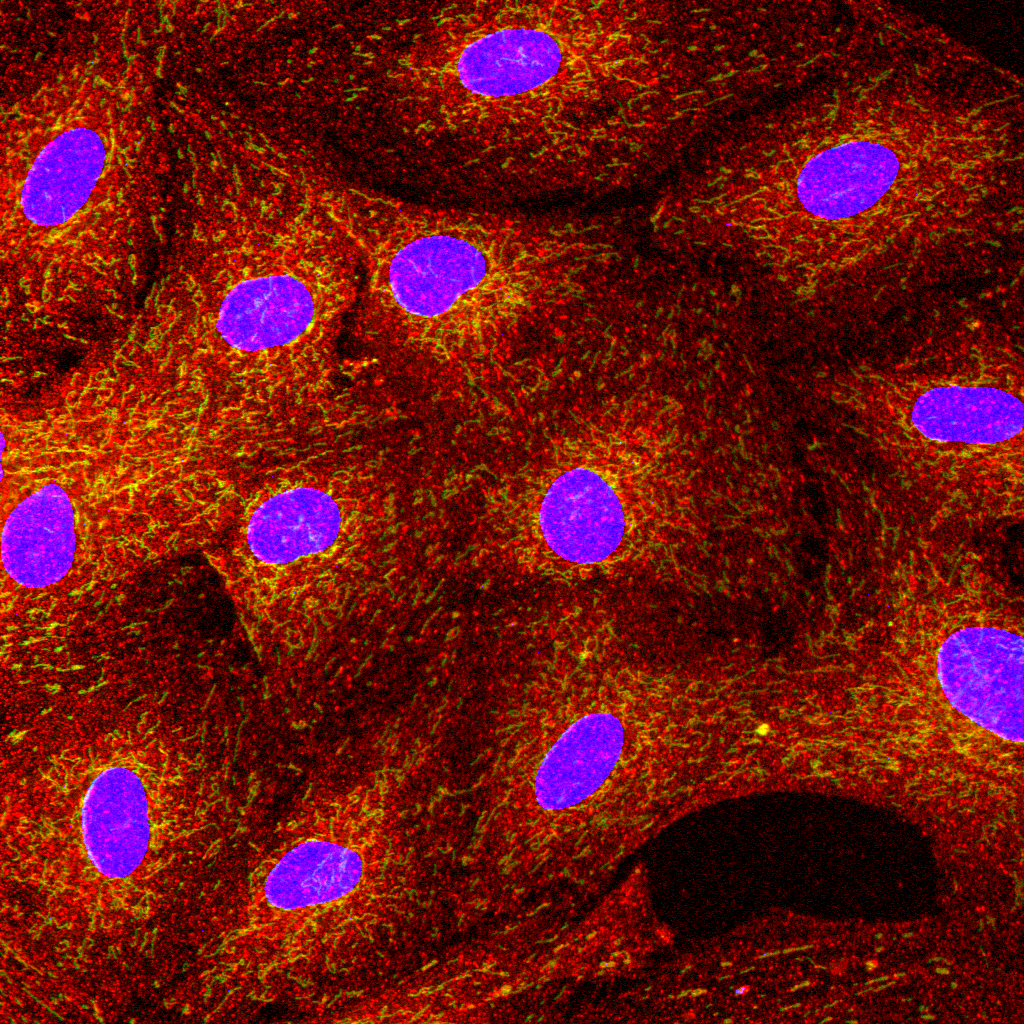

Supplement: Supplementary file 5 — Supplementary material [file mmc5.zip › GK Glucose Time Course - 1st Experiment/E1-G2-5.tif]

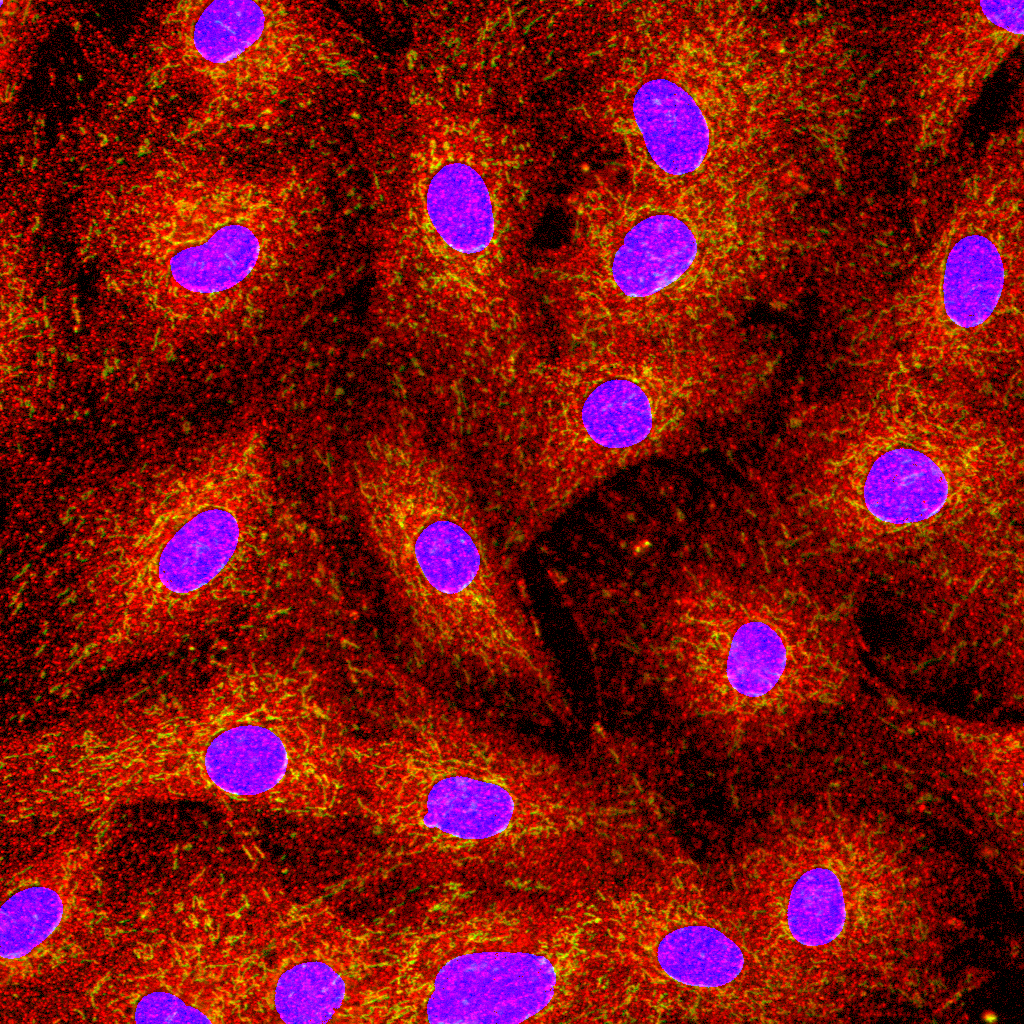

Supplement: Supplementary file 5 — Supplementary material [file mmc5.zip › GK Glucose Time Course - 1st Experiment/E1-G2-6.tif]

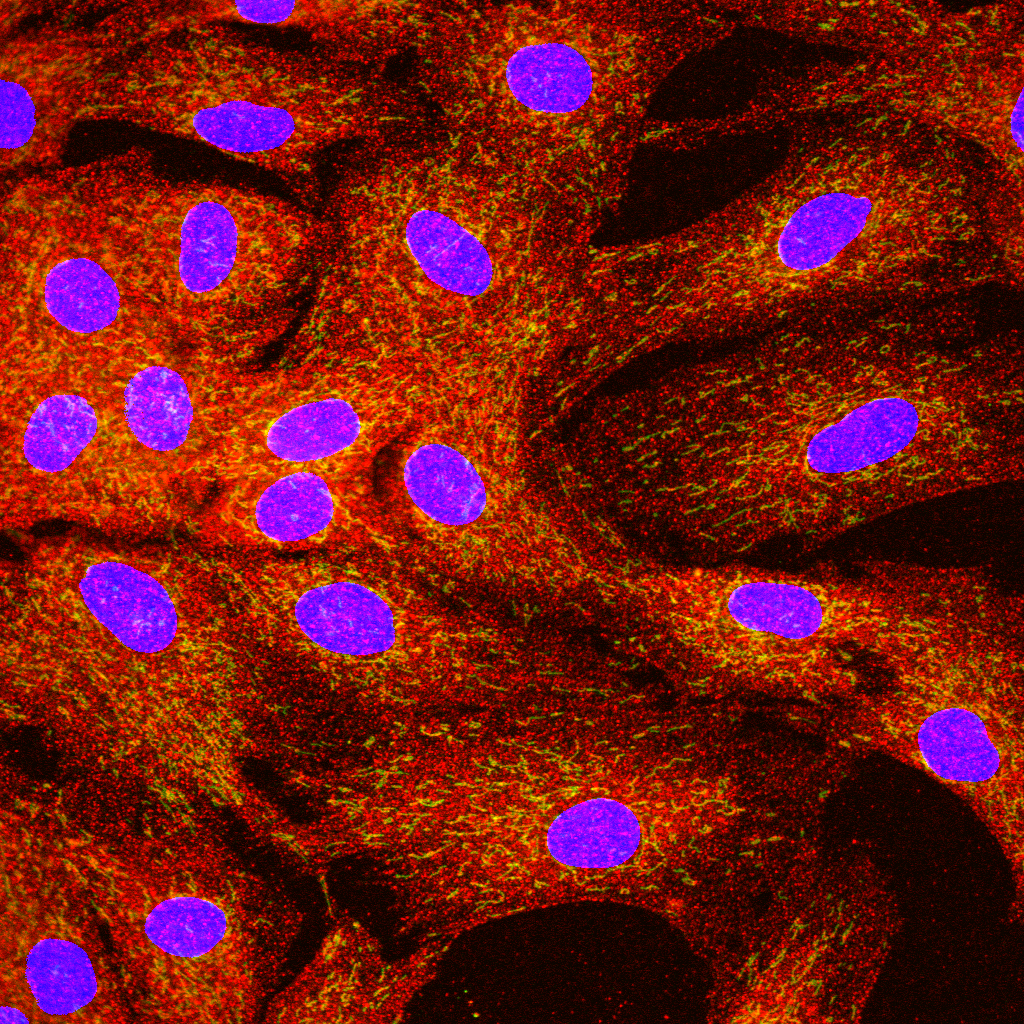

Supplement: Supplementary file 5 — Supplementary material [file mmc5.zip › GK Glucose Time Course - 1st Experiment/E1-G2-7.tif]

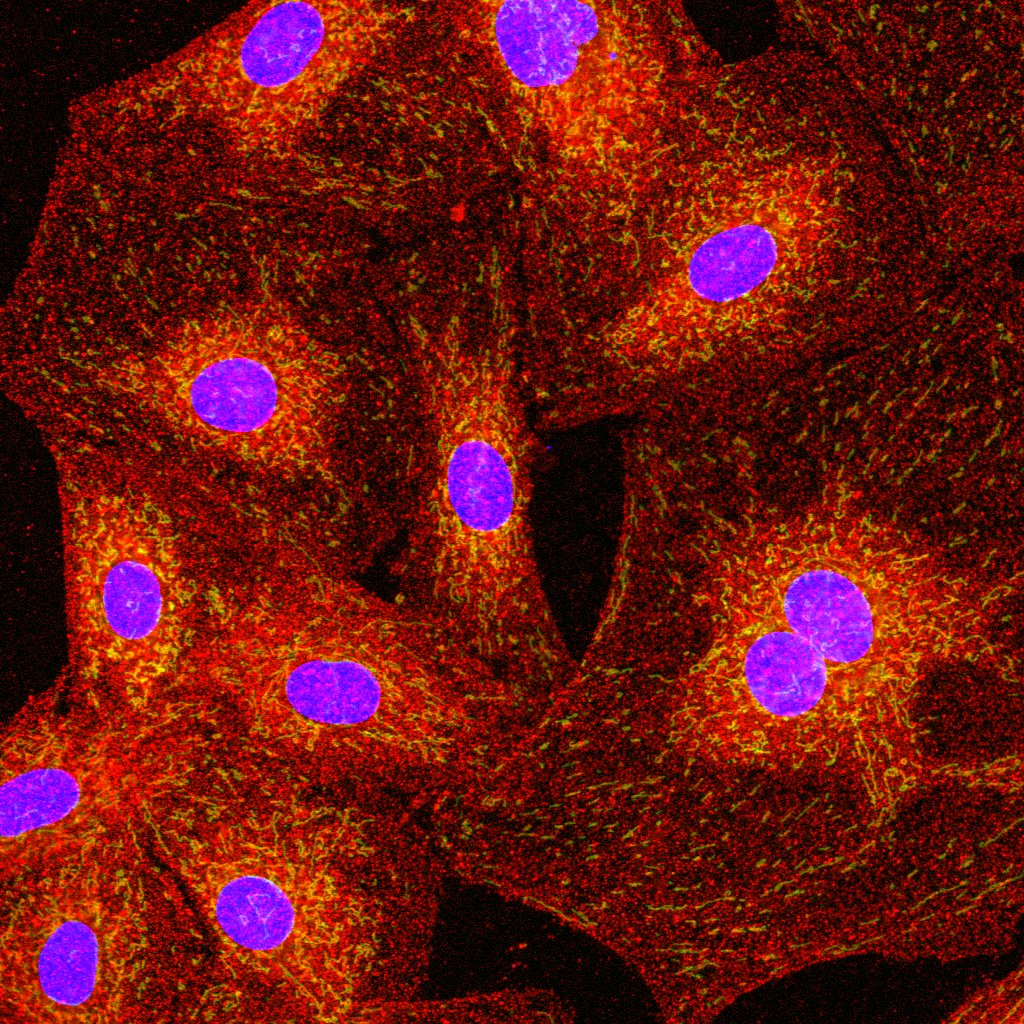

Supplement: Supplementary file 5 — Supplementary material [file mmc5.zip › GK Glucose Time Course - 1st Experiment/E1-G2-8.tif]

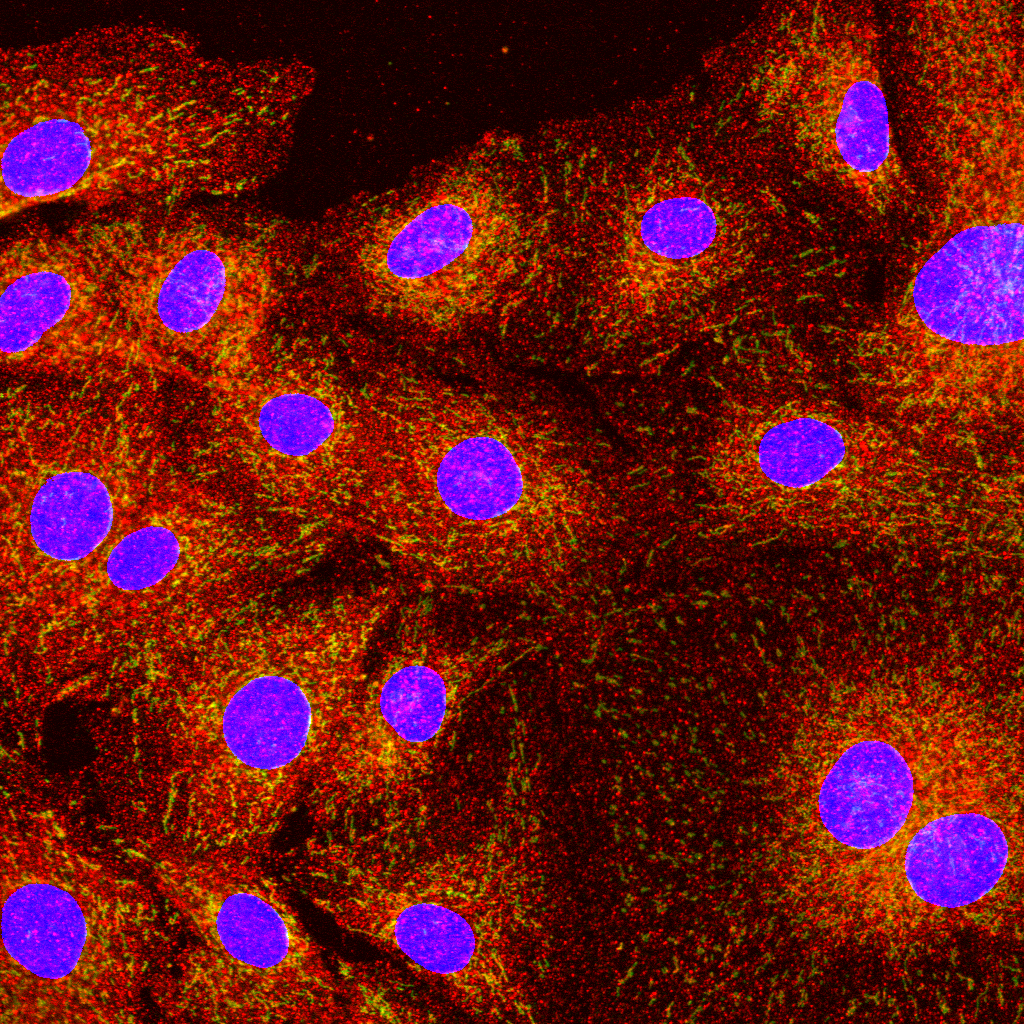

Supplement: Supplementary file 5 — Supplementary material [file mmc5.zip › GK Glucose Time Course - 1st Experiment/E1-G2-9.tif]

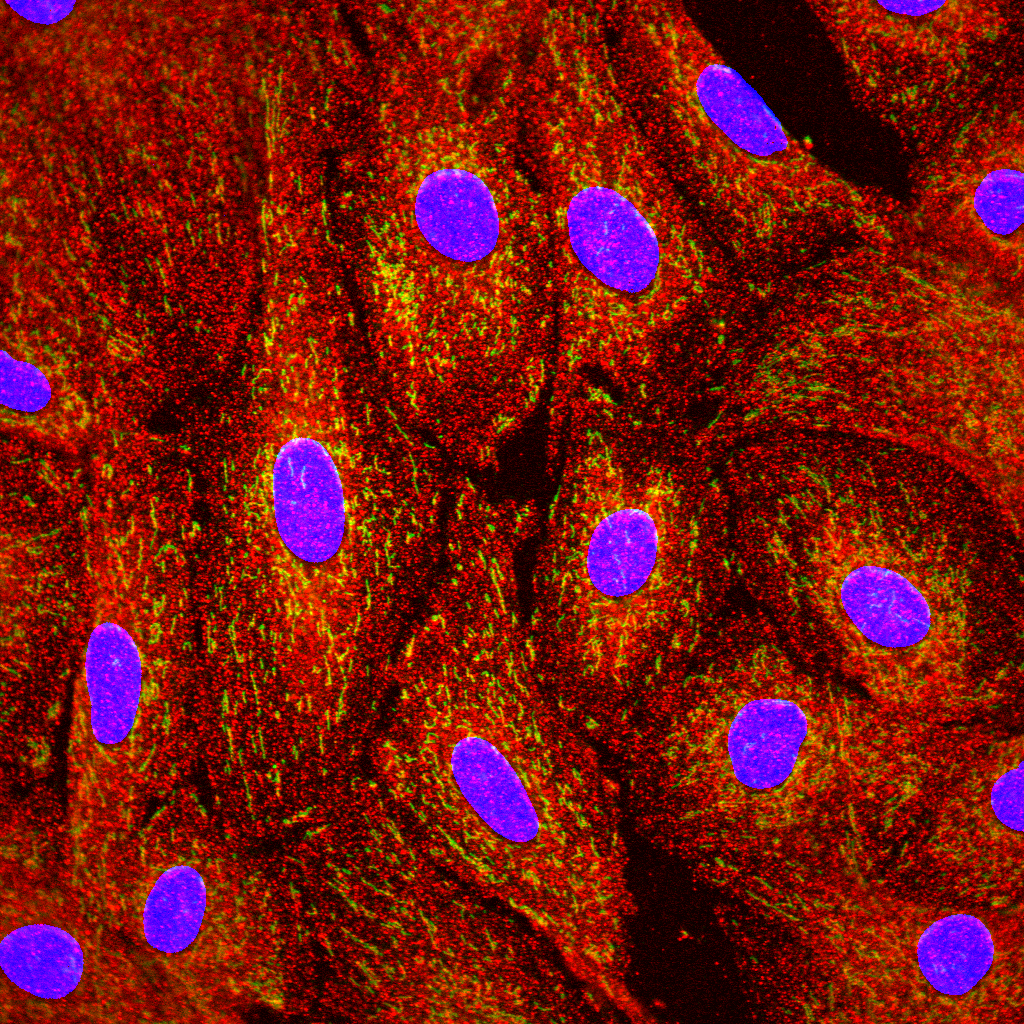

Supplement: Supplementary file 5 — Supplementary material [file mmc5.zip › GK Glucose Time Course - 1st Experiment/E1-G3-1.tif]

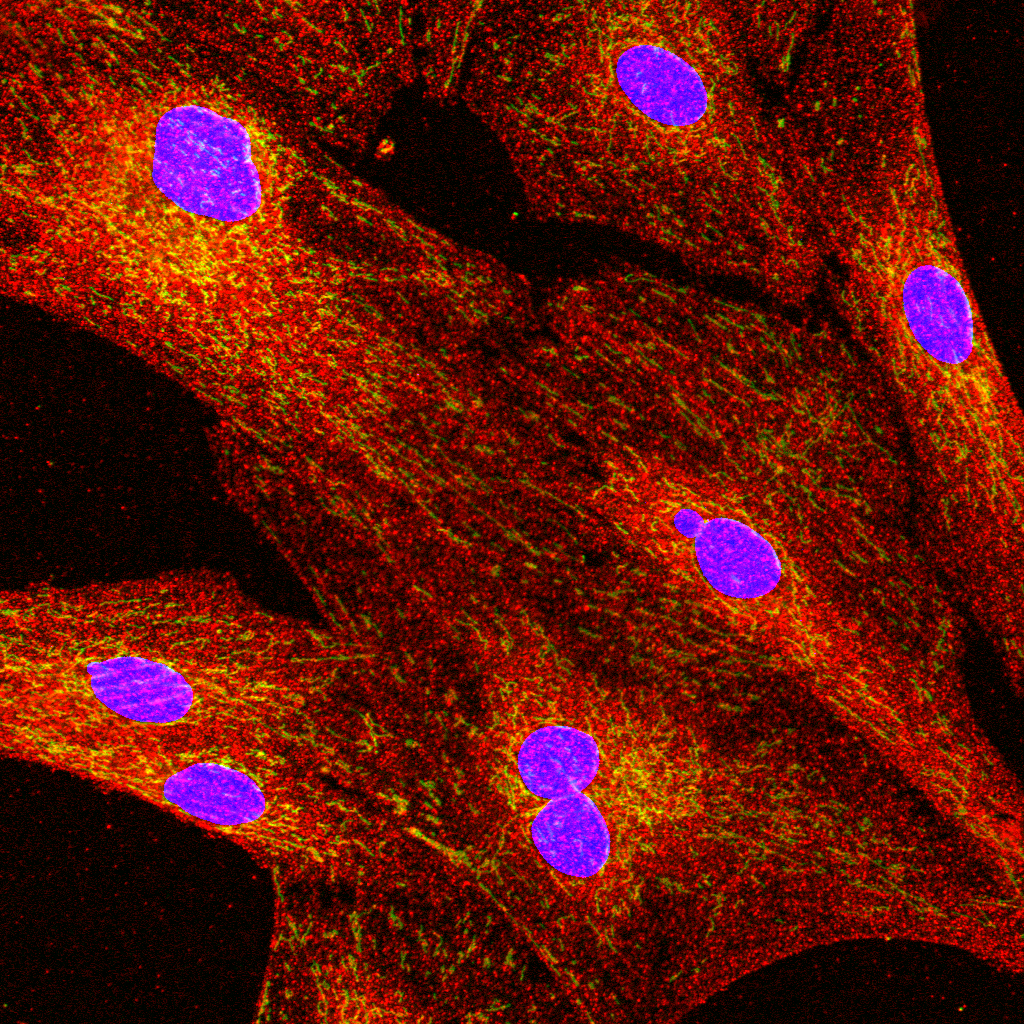

Supplement: Supplementary file 5 — Supplementary material [file mmc5.zip › GK Glucose Time Course - 1st Experiment/E1-G3-2.tif]

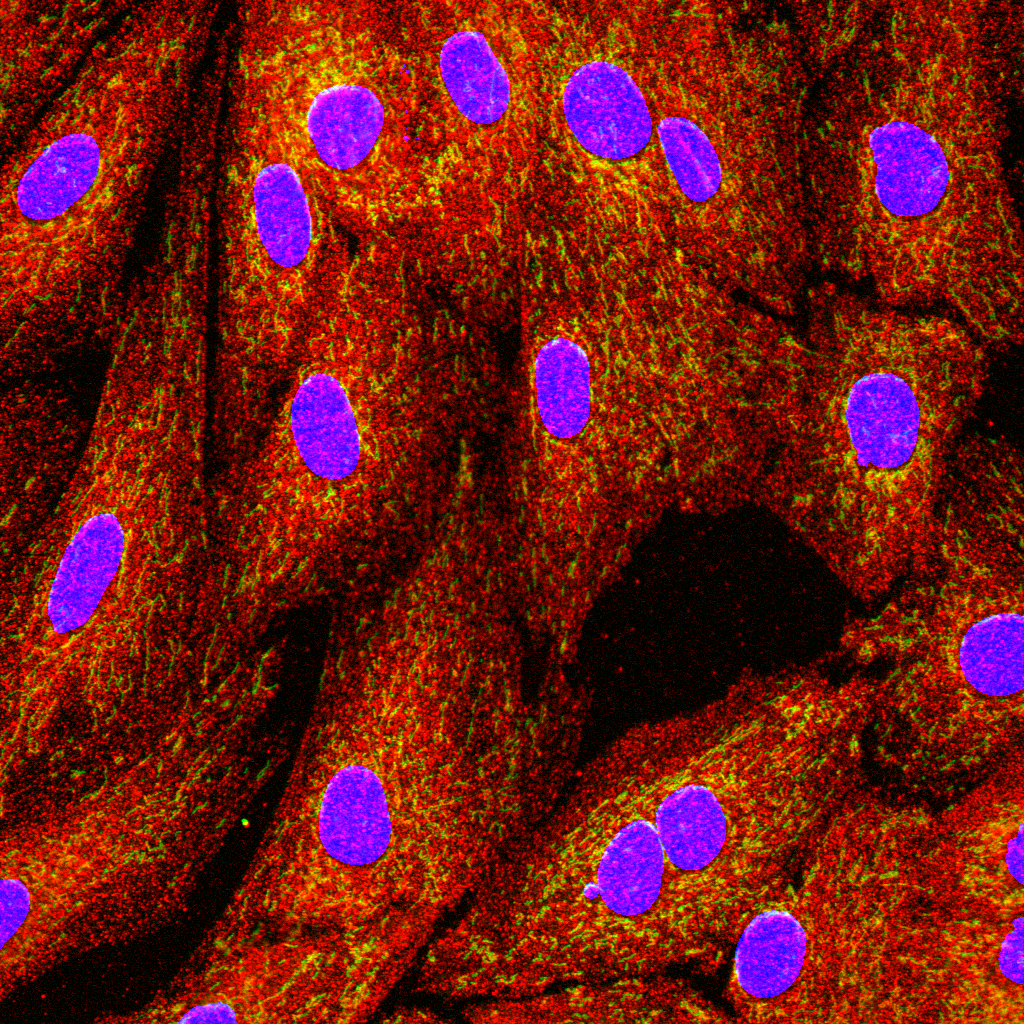

Supplement: Supplementary file 5 — Supplementary material [file mmc5.zip › GK Glucose Time Course - 1st Experiment/E1-G3-3.tif]

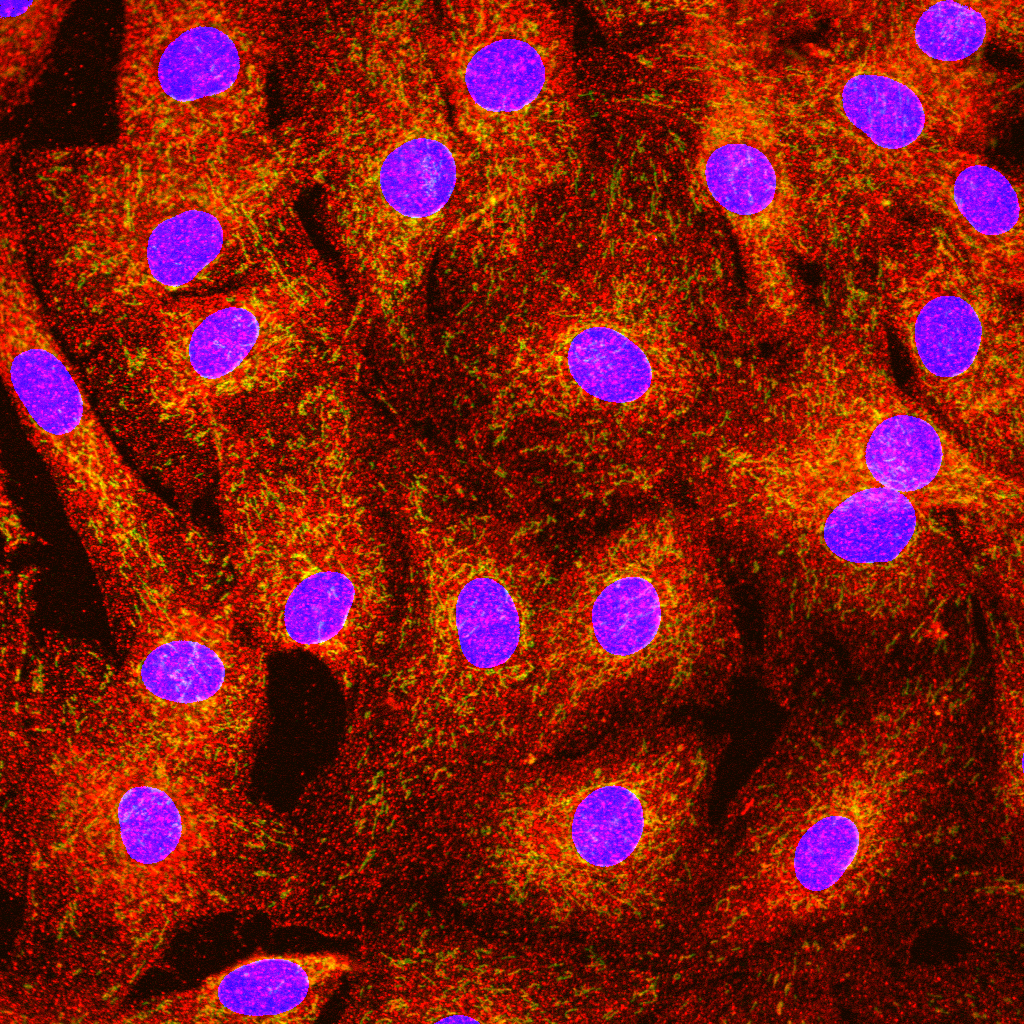

Supplement: Supplementary file 5 — Supplementary material [file mmc5.zip › GK Glucose Time Course - 1st Experiment/E1-G3-4.tif]

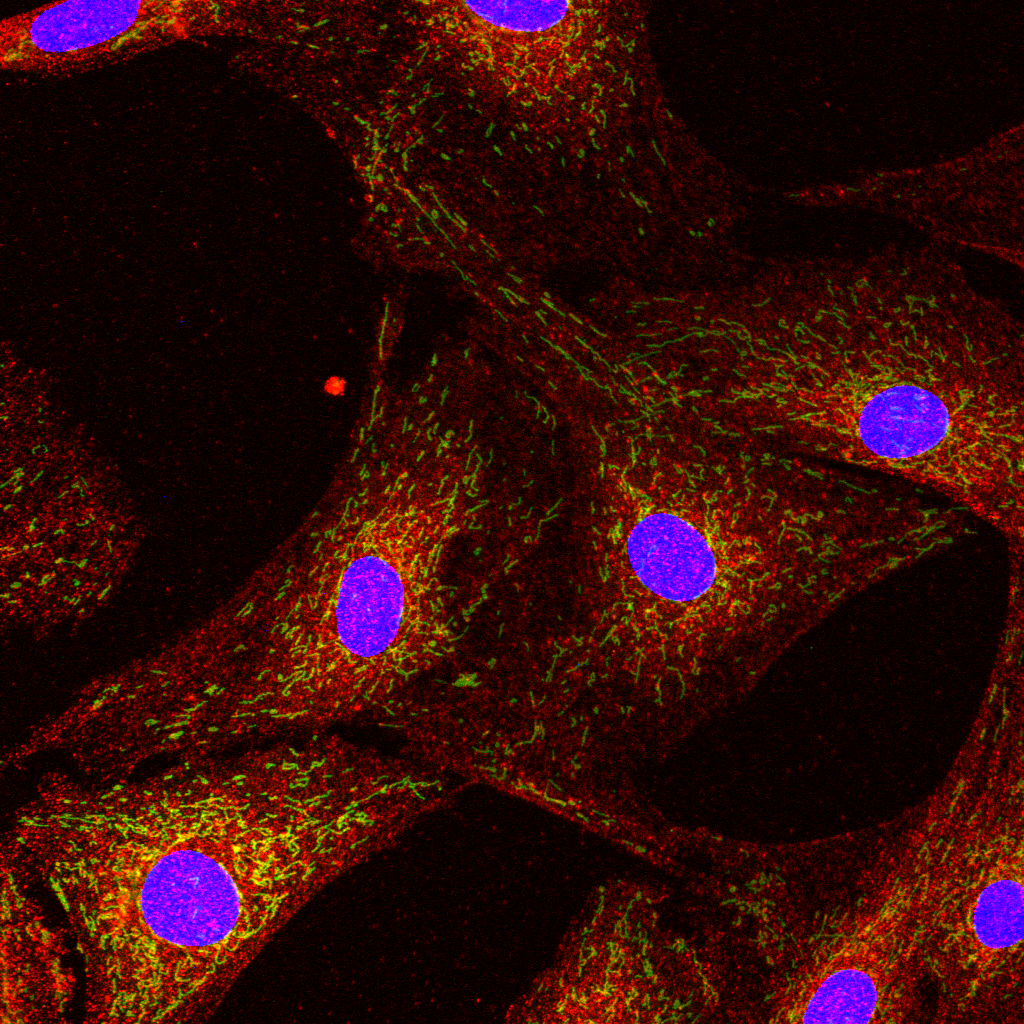

Supplement: Supplementary file 5 — Supplementary material [file mmc5.zip › GK Glucose Time Course - 1st Experiment/E1-G3-5.tif]

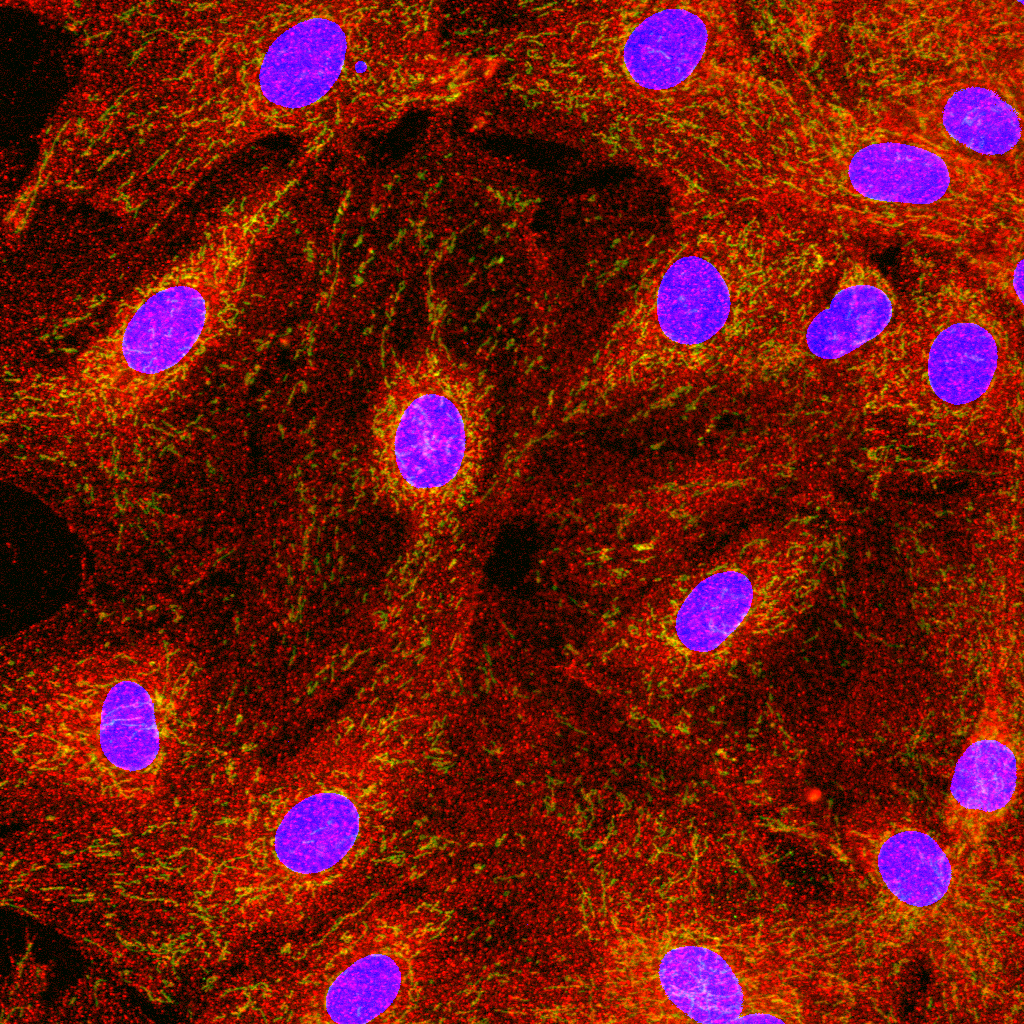

Supplement: Supplementary file 5 — Supplementary material [file mmc5.zip › GK Glucose Time Course - 1st Experiment/E1-G3-6.tif]

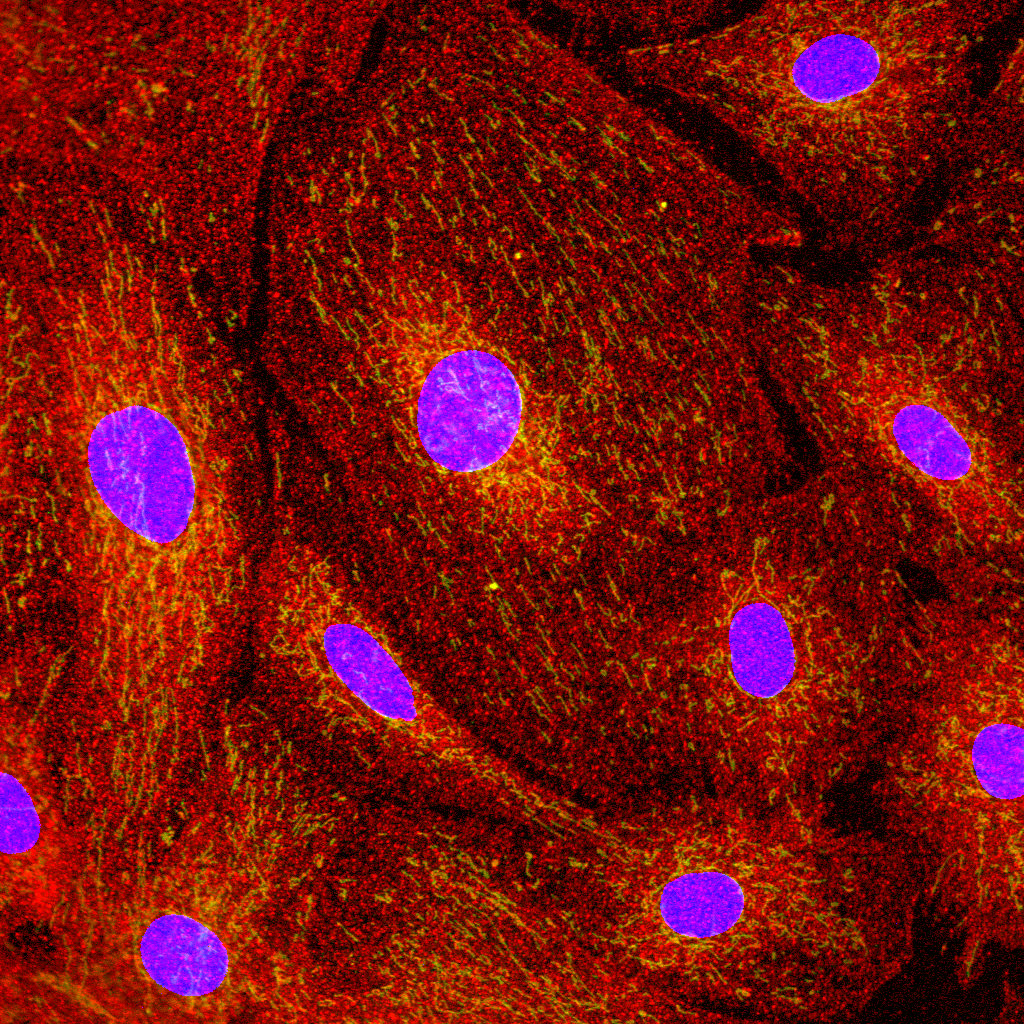

Supplement: Supplementary file 5 — Supplementary material [file mmc5.zip › GK Glucose Time Course - 1st Experiment/E1-G3-7.tif]

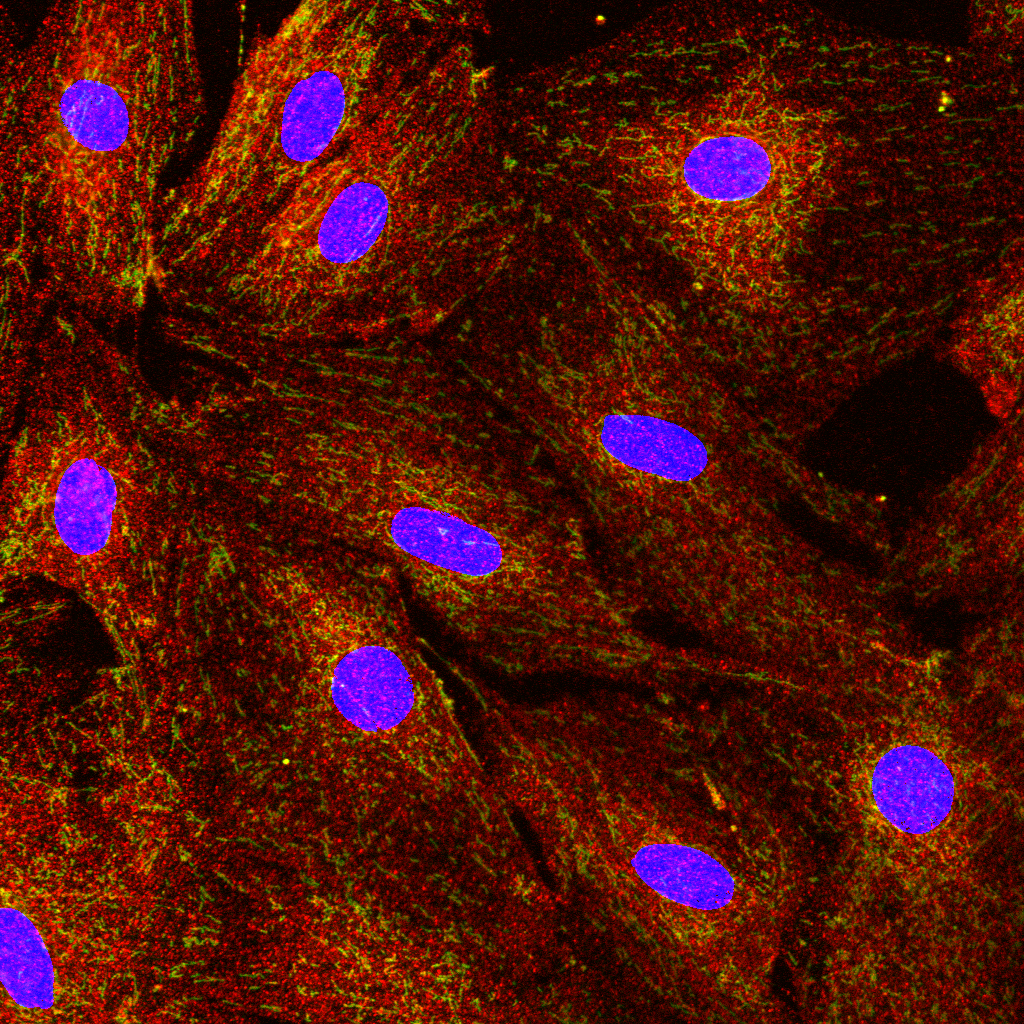

Supplement: Supplementary file 5 — Supplementary material [file mmc5.zip › GK Glucose Time Course - 1st Experiment/E1-G3-8.tif]

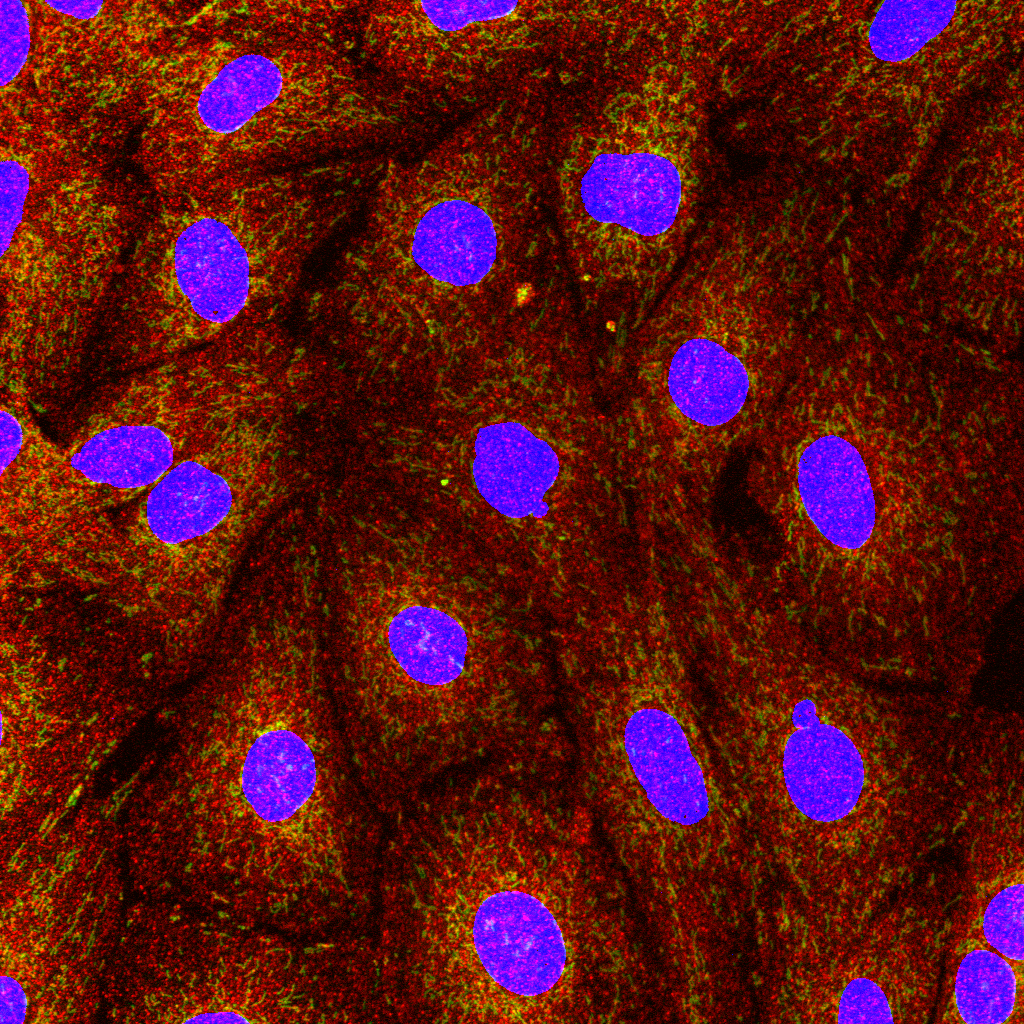

Supplement: Supplementary file 5 — Supplementary material [file mmc5.zip › GK Glucose Time Course - 1st Experiment/E1-G3-9.tif]

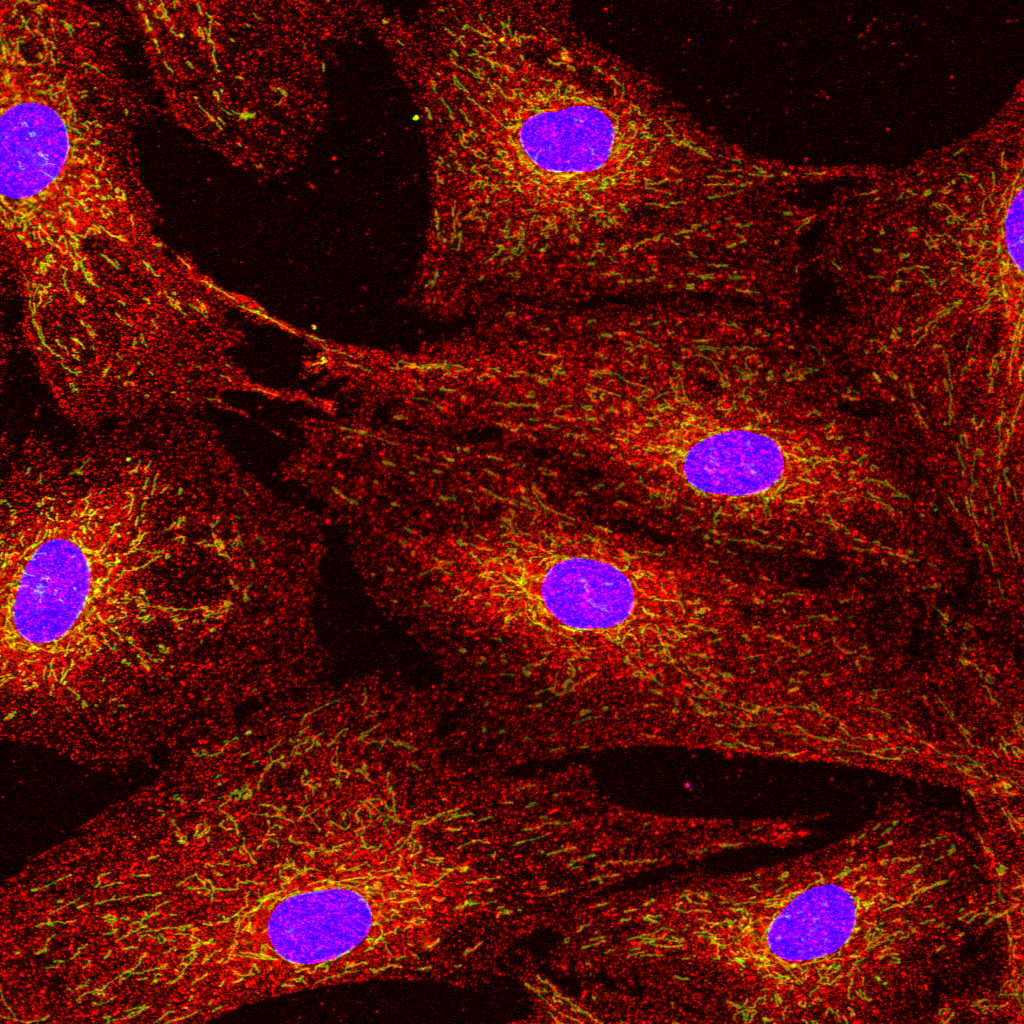

Supplement: Supplementary file 5 — Supplementary material [file mmc5.zip › GK Glucose Time Course - 1st Experiment/E1-G4-1.tif]

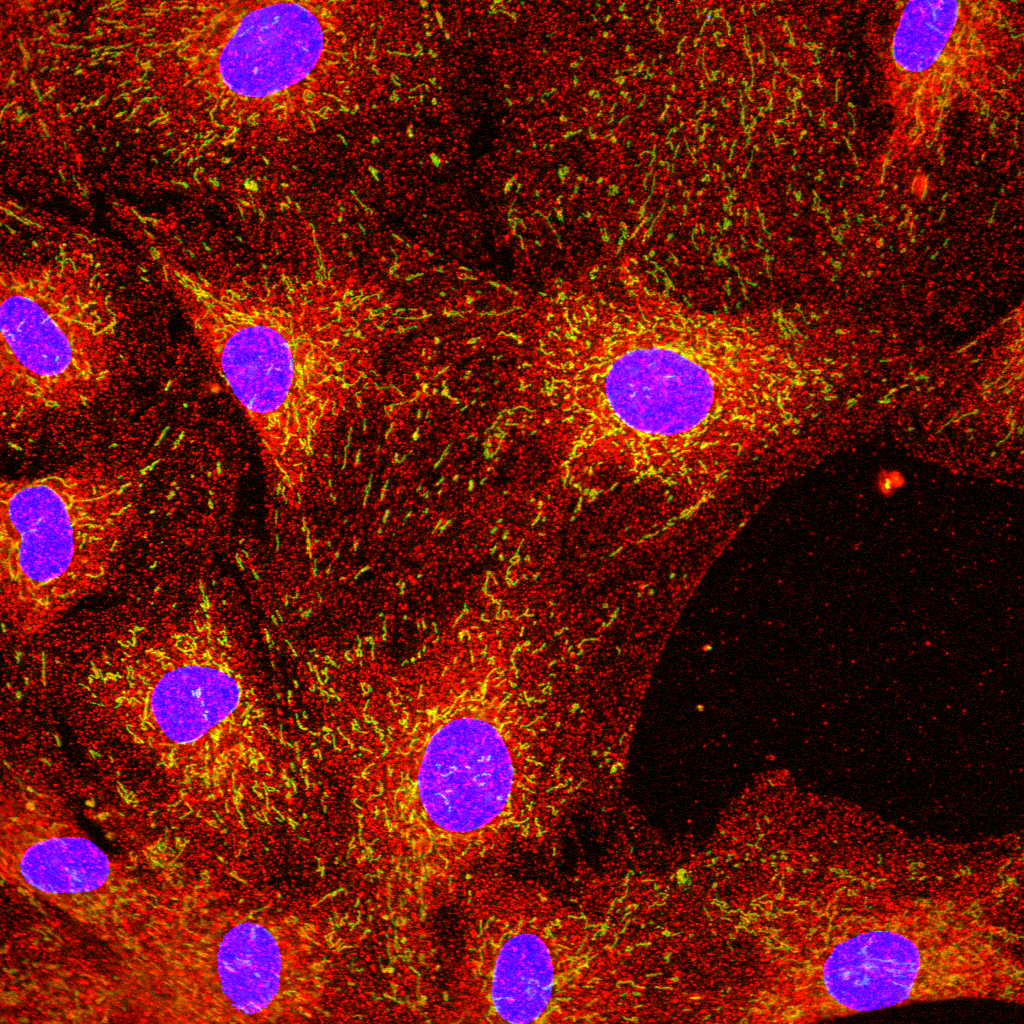

Supplement: Supplementary file 5 — Supplementary material [file mmc5.zip › GK Glucose Time Course - 1st Experiment/E1-G4-2.tif]

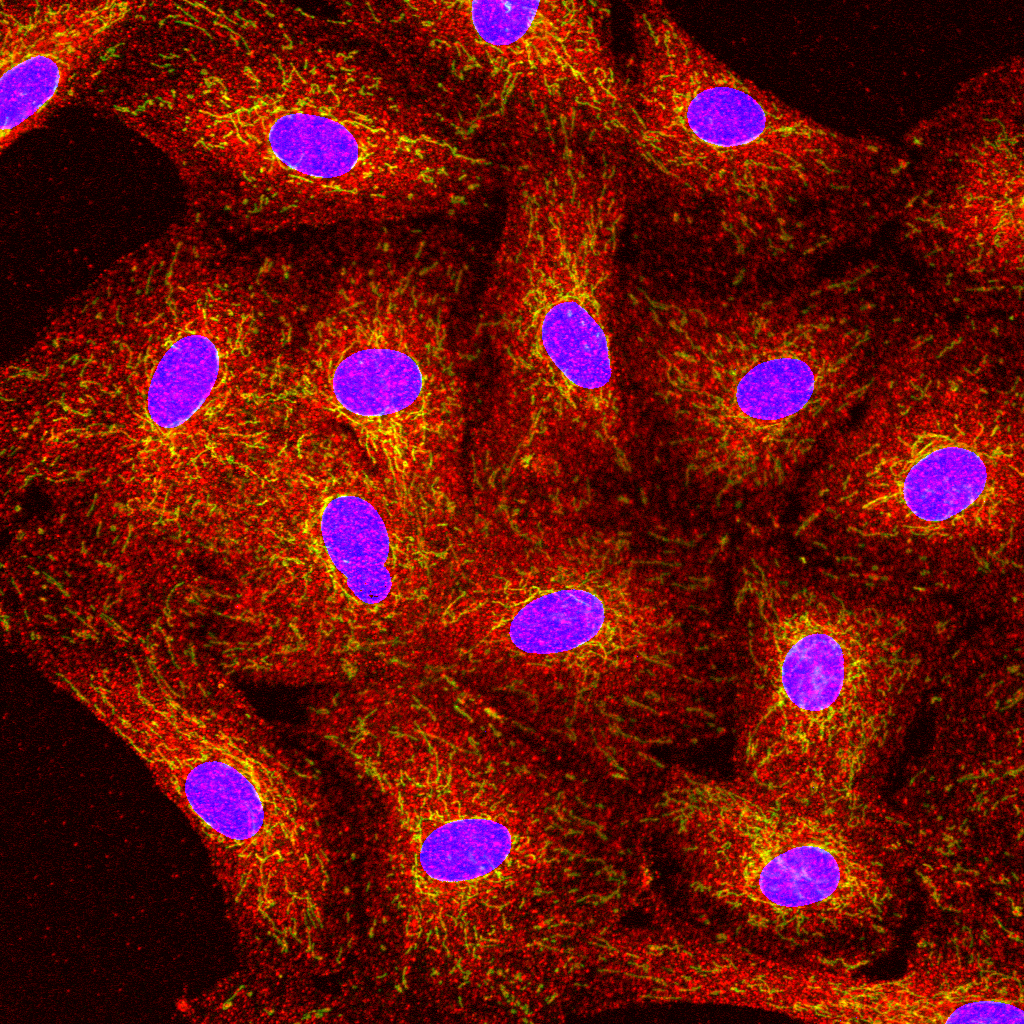

Supplement: Supplementary file 5 — Supplementary material [file mmc5.zip › GK Glucose Time Course - 1st Experiment/E1-G4-3.tif]

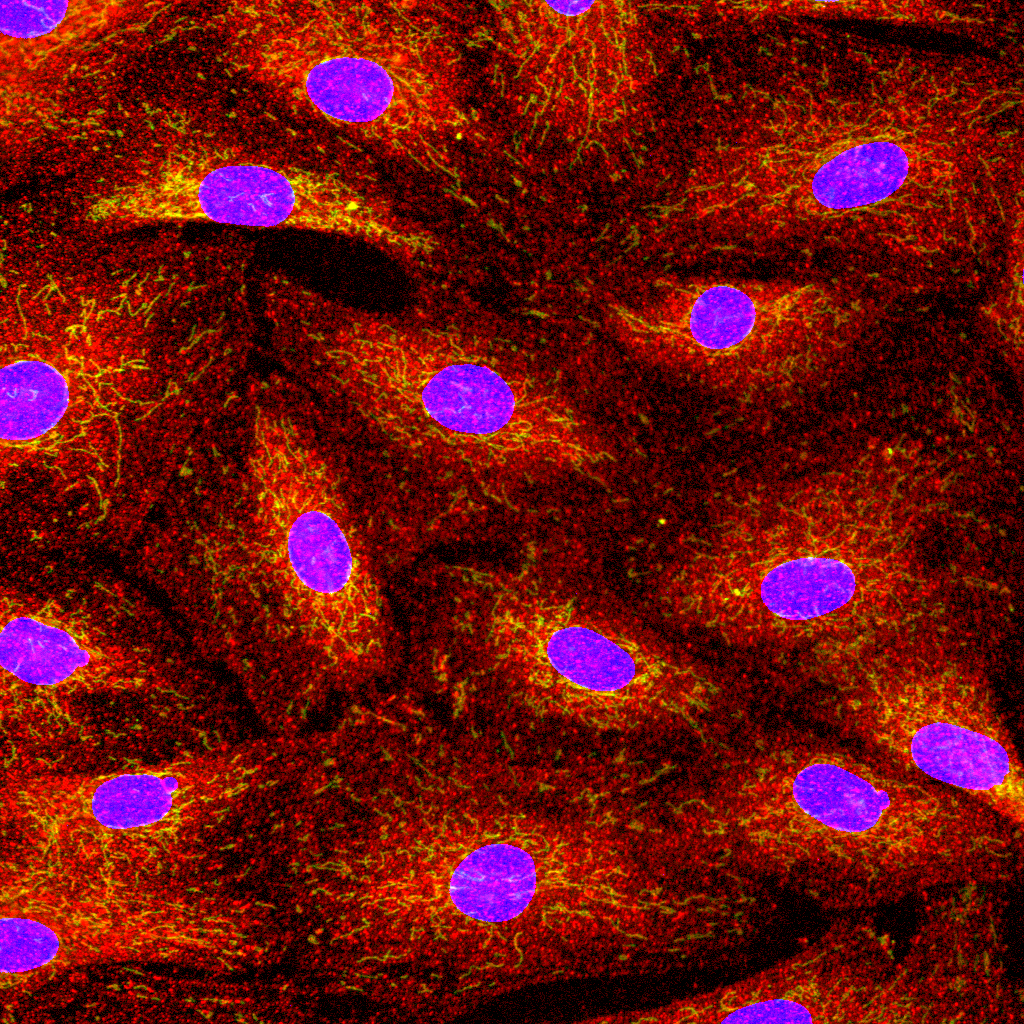

Supplement: Supplementary file 5 — Supplementary material [file mmc5.zip › GK Glucose Time Course - 1st Experiment/E1-G4-4.tif]

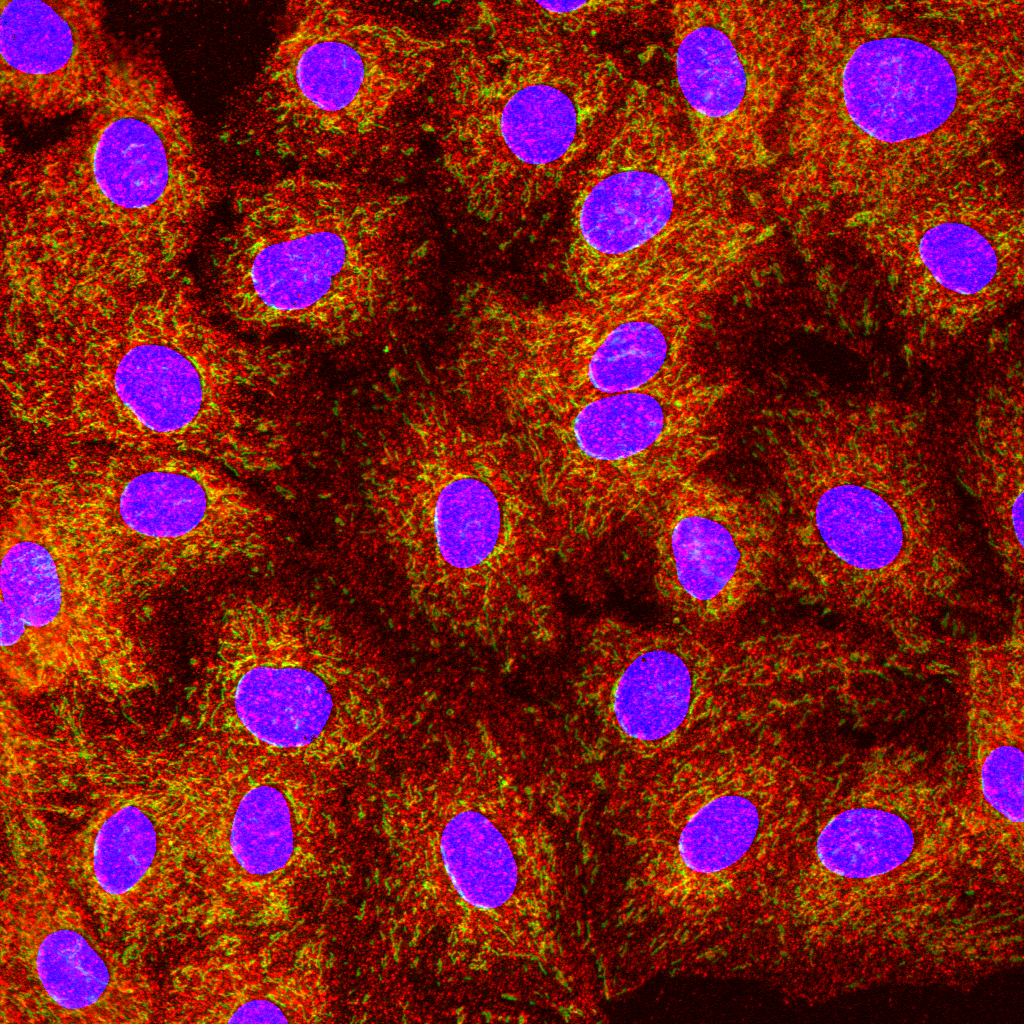

Supplement: Supplementary file 5 — Supplementary material [file mmc5.zip › GK Glucose Time Course - 1st Experiment/E1-G4-5.tif]

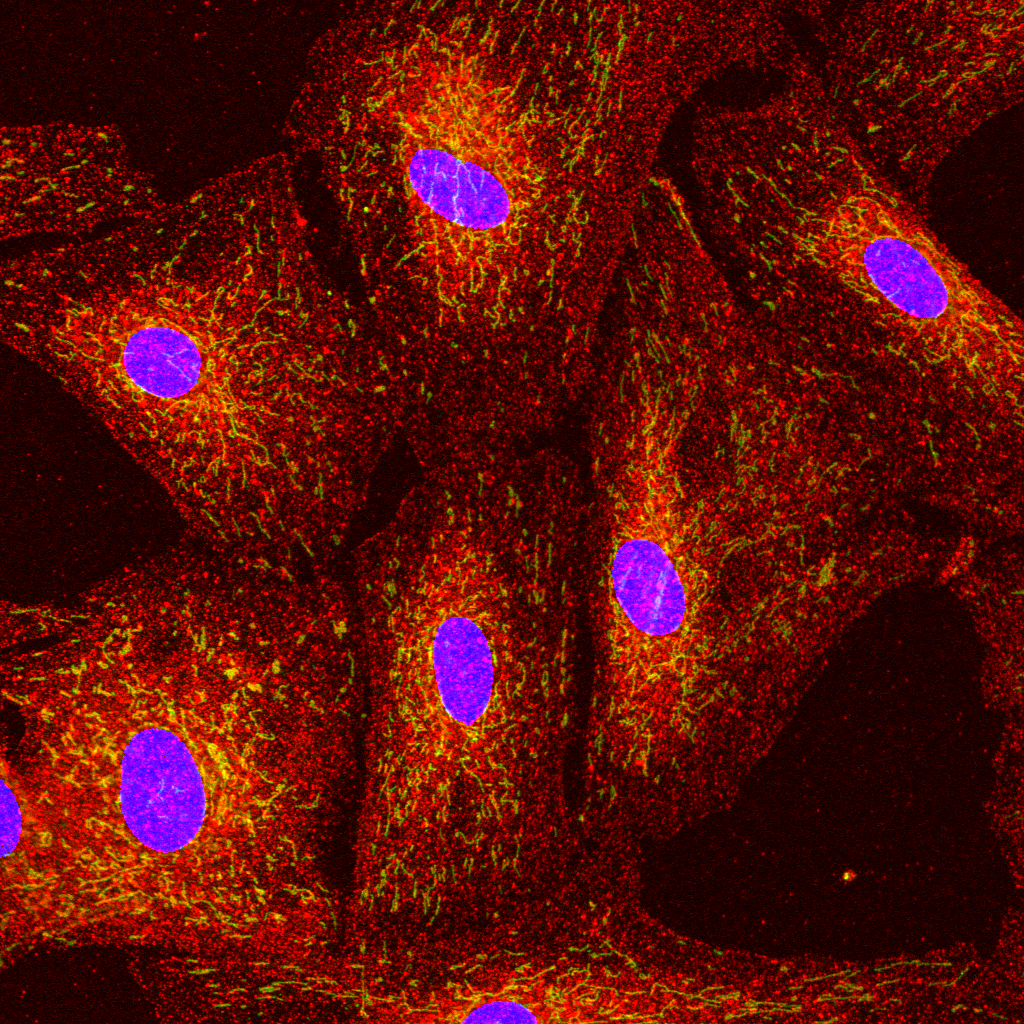

Supplement: Supplementary file 5 — Supplementary material [file mmc5.zip › GK Glucose Time Course - 1st Experiment/E1-G4-6.tif]

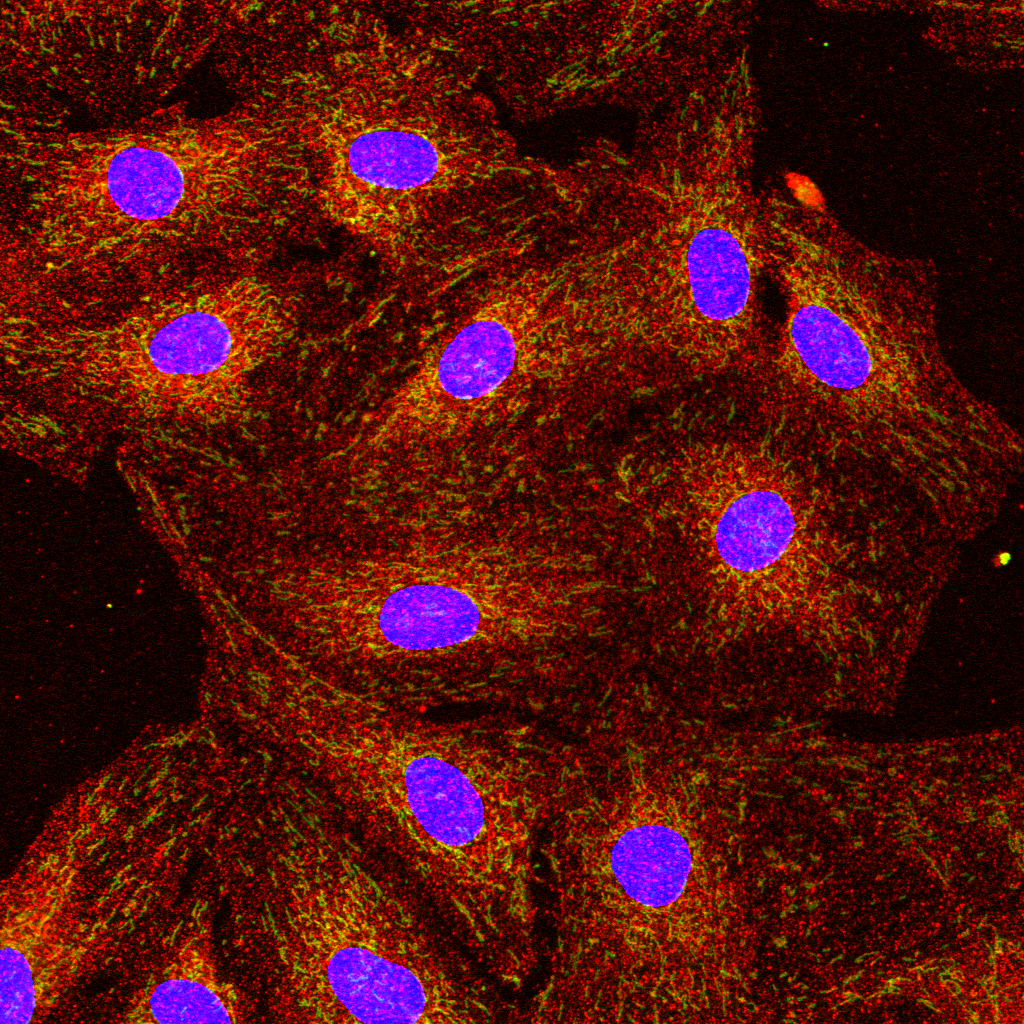

Supplement: Supplementary file 5 — Supplementary material [file mmc5.zip › GK Glucose Time Course - 1st Experiment/E1-G4-7.tif]

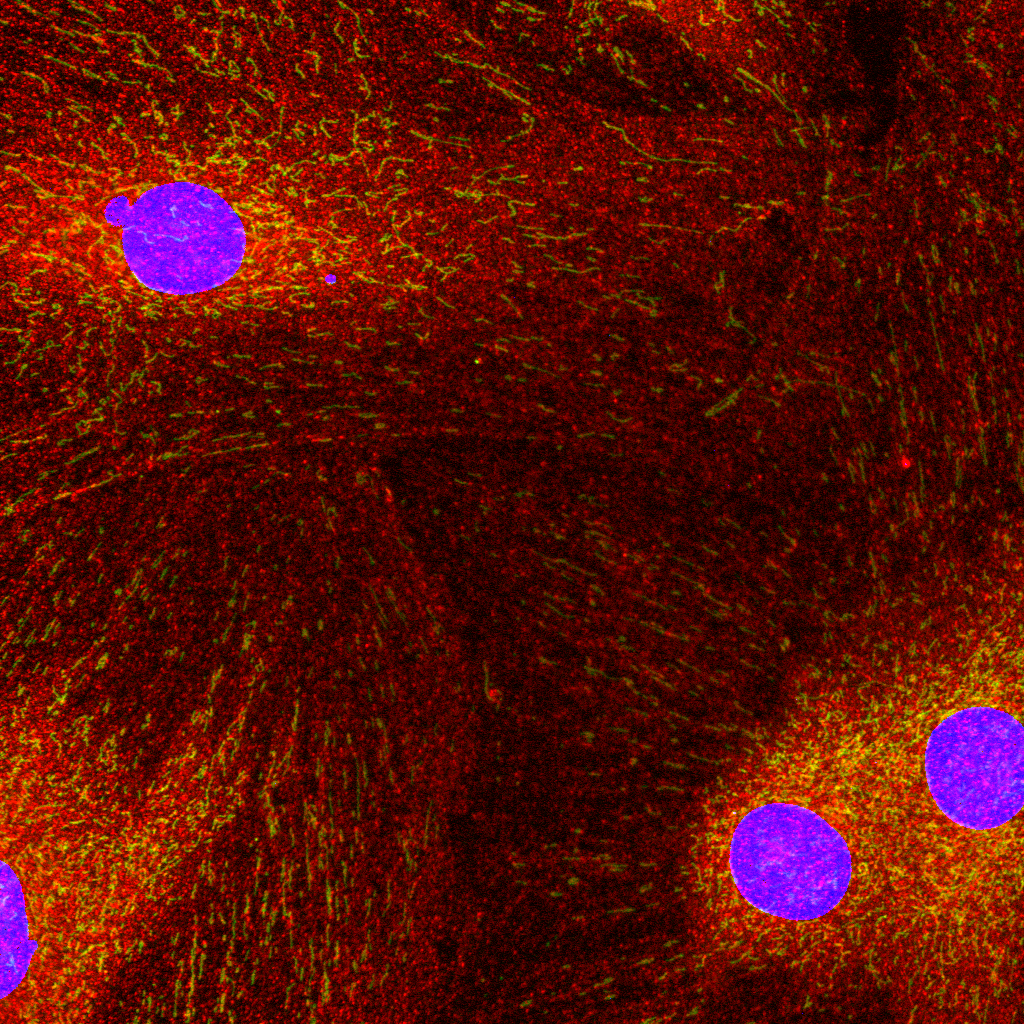

Supplement: Supplementary file 5 — Supplementary material [file mmc5.zip › GK Glucose Time Course - 1st Experiment/E1-G4-8.tif]

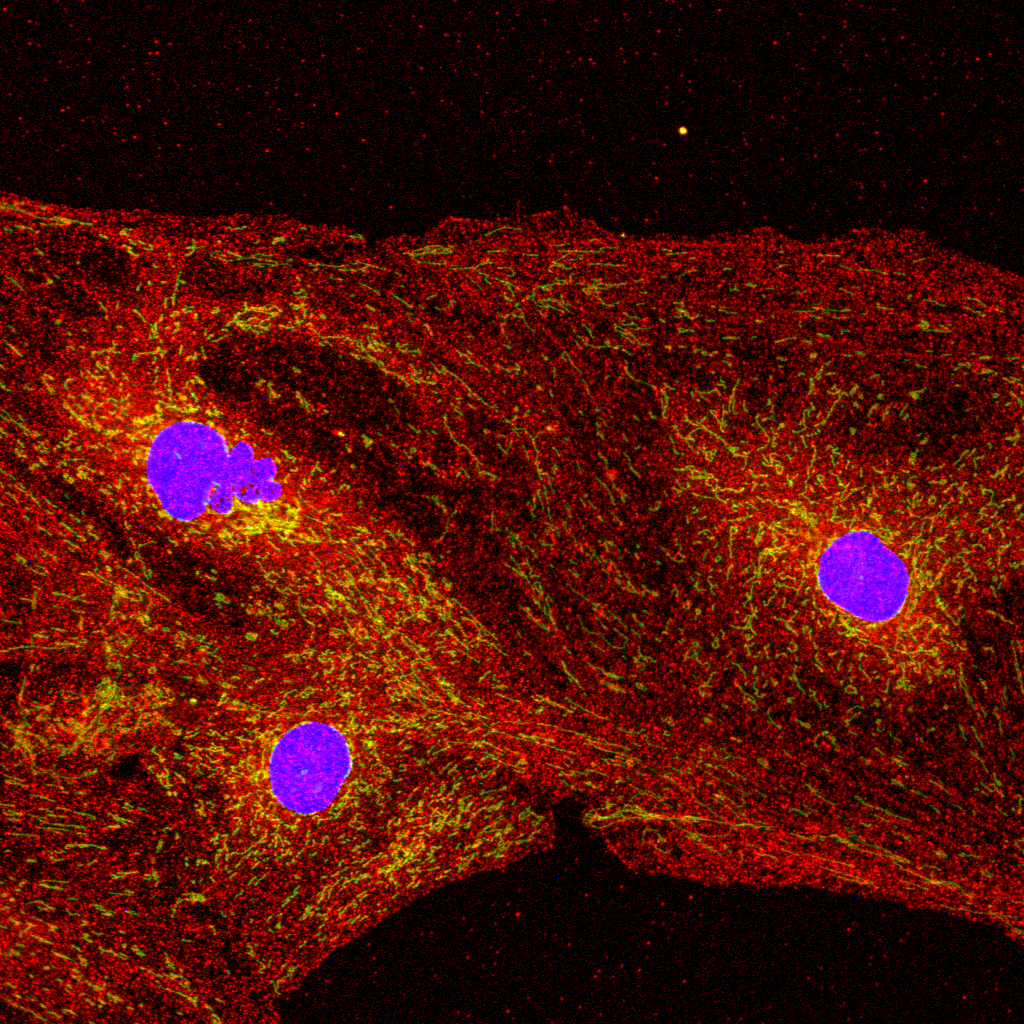

Supplement: Supplementary file 5 — Supplementary material [file mmc5.zip › GK Glucose Time Course - 1st Experiment/E1-G4-9.tif]

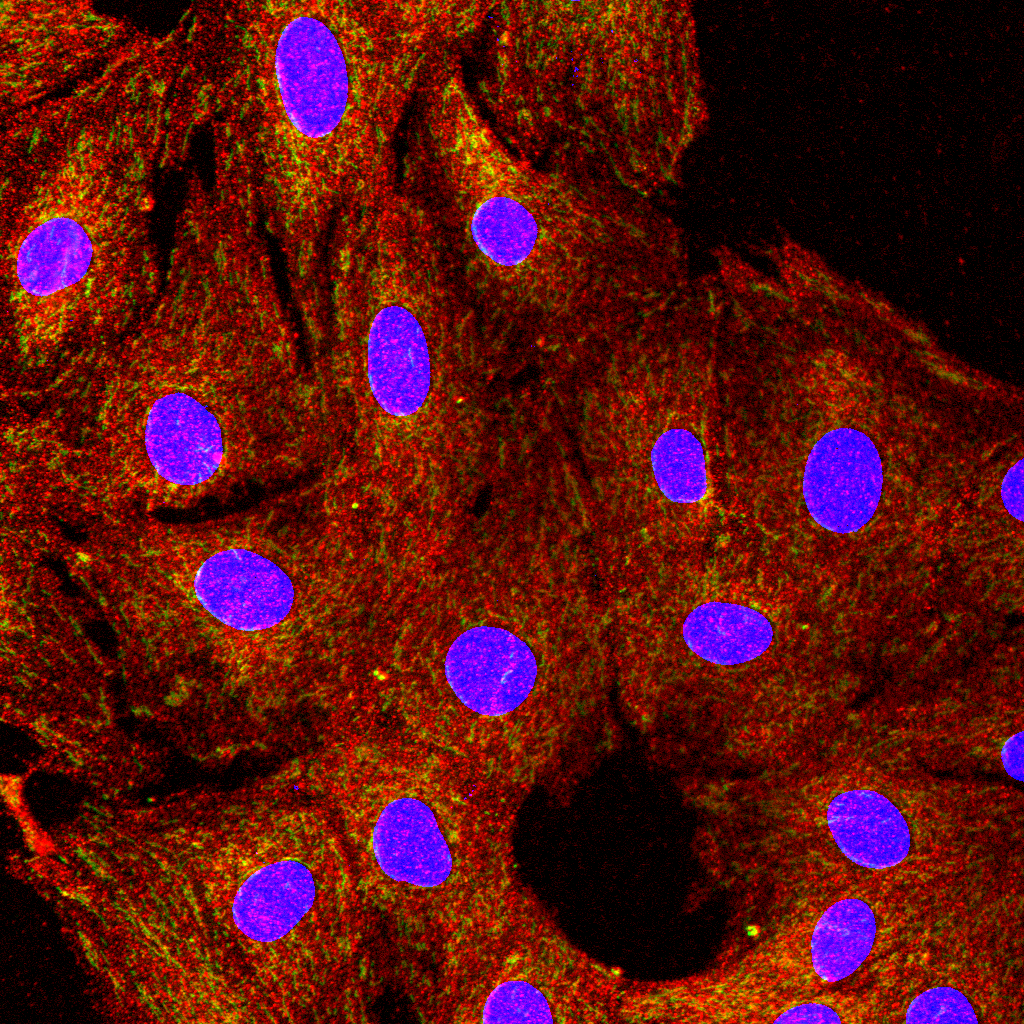

Supplement: Supplementary file 5 — Supplementary material [file mmc5.zip › GK Glucose Time Course - 1st Experiment/E1-G5-1.tif]

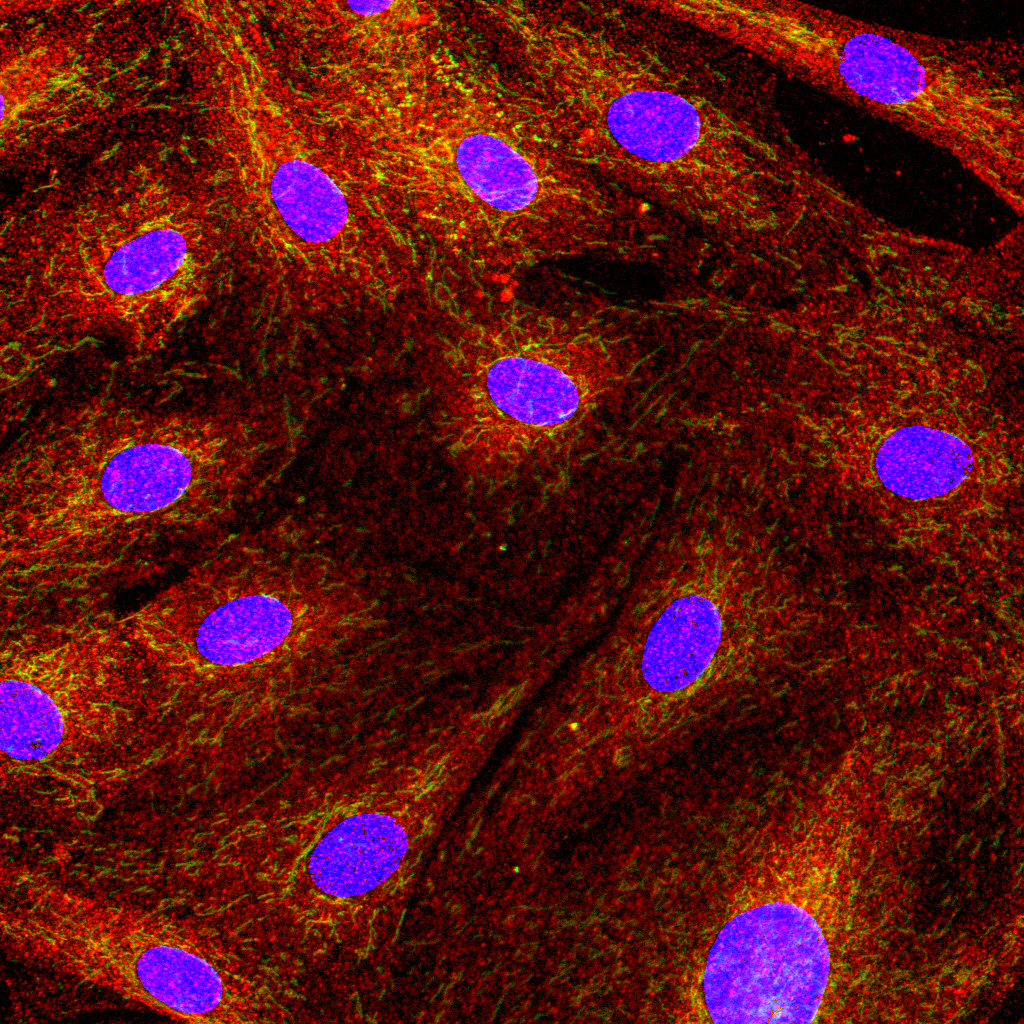

Supplement: Supplementary file 5 — Supplementary material [file mmc5.zip › GK Glucose Time Course - 1st Experiment/E1-G5-2.tif]

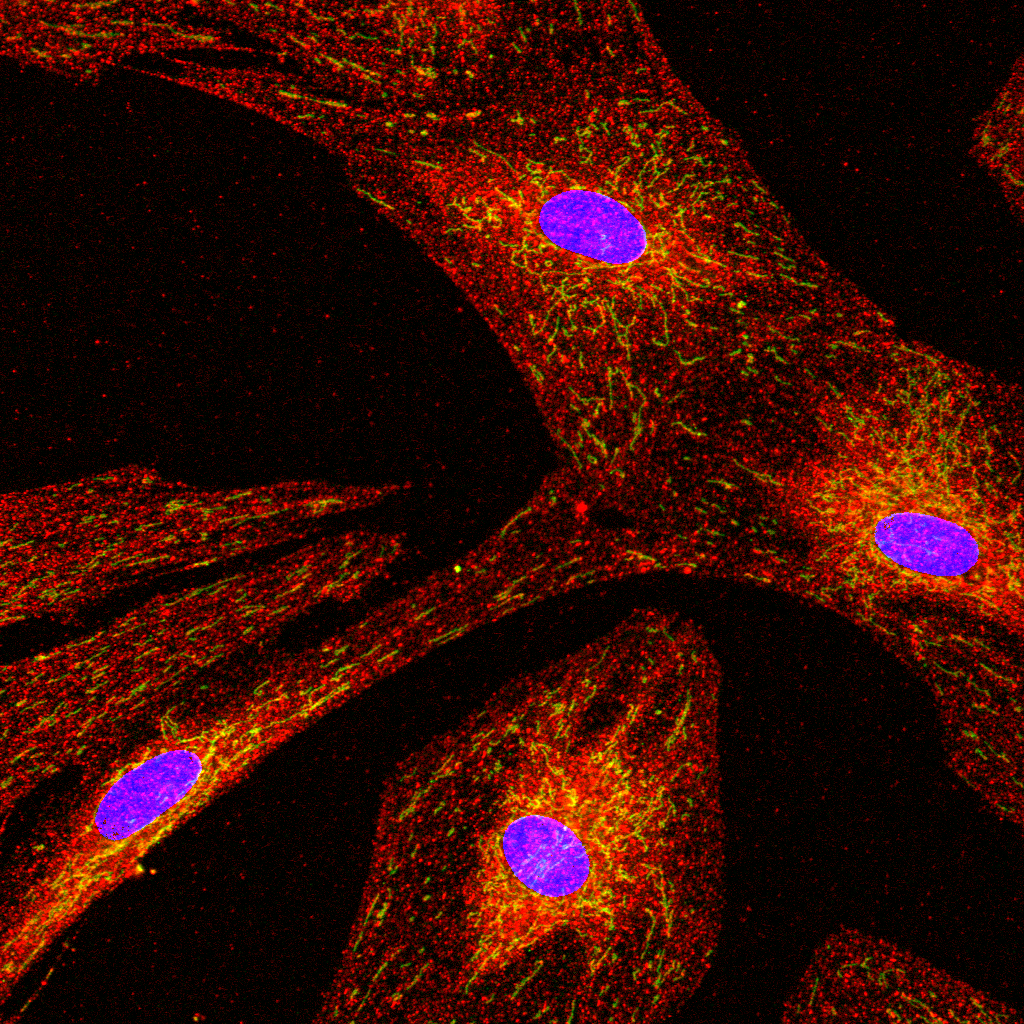

Supplement: Supplementary file 5 — Supplementary material [file mmc5.zip › GK Glucose Time Course - 1st Experiment/E1-G5-3.tif]

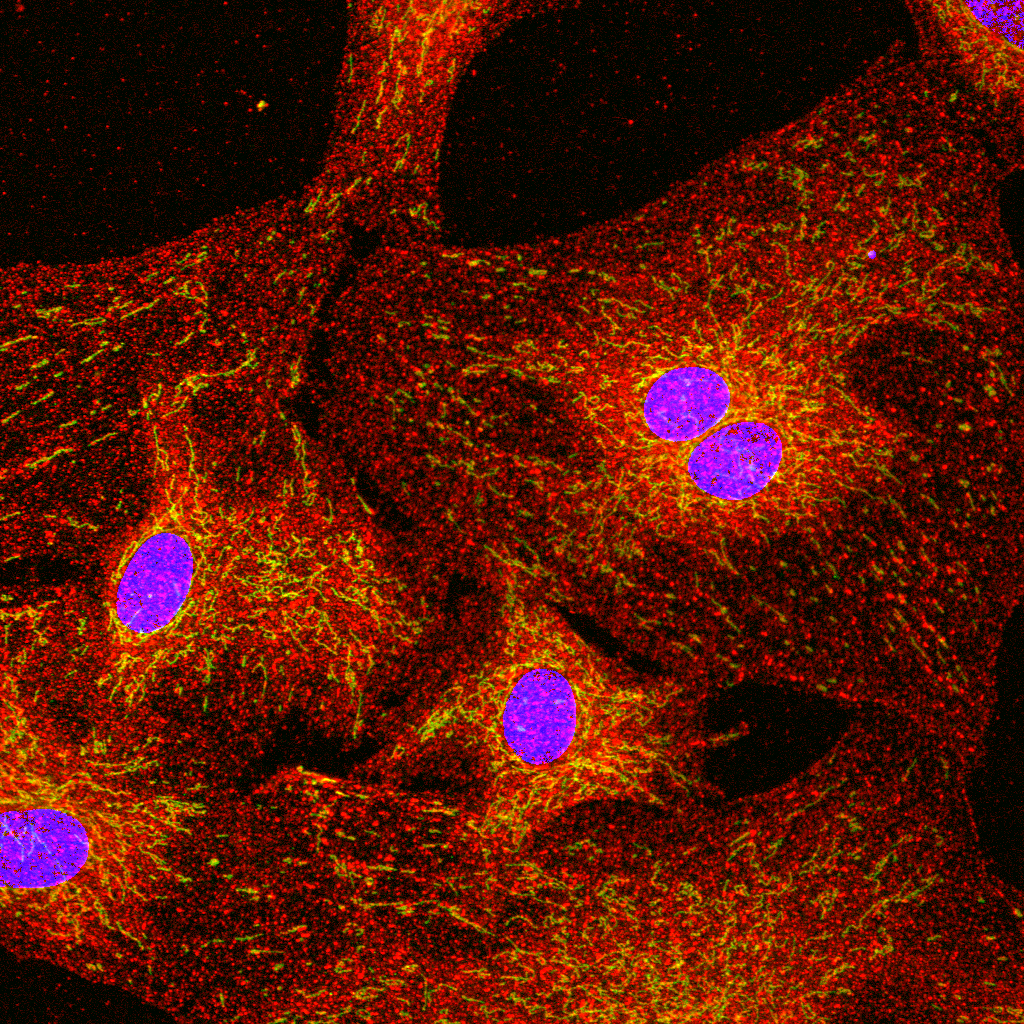

Supplement: Supplementary file 5 — Supplementary material [file mmc5.zip › GK Glucose Time Course - 1st Experiment/E1-G5-4.tif]

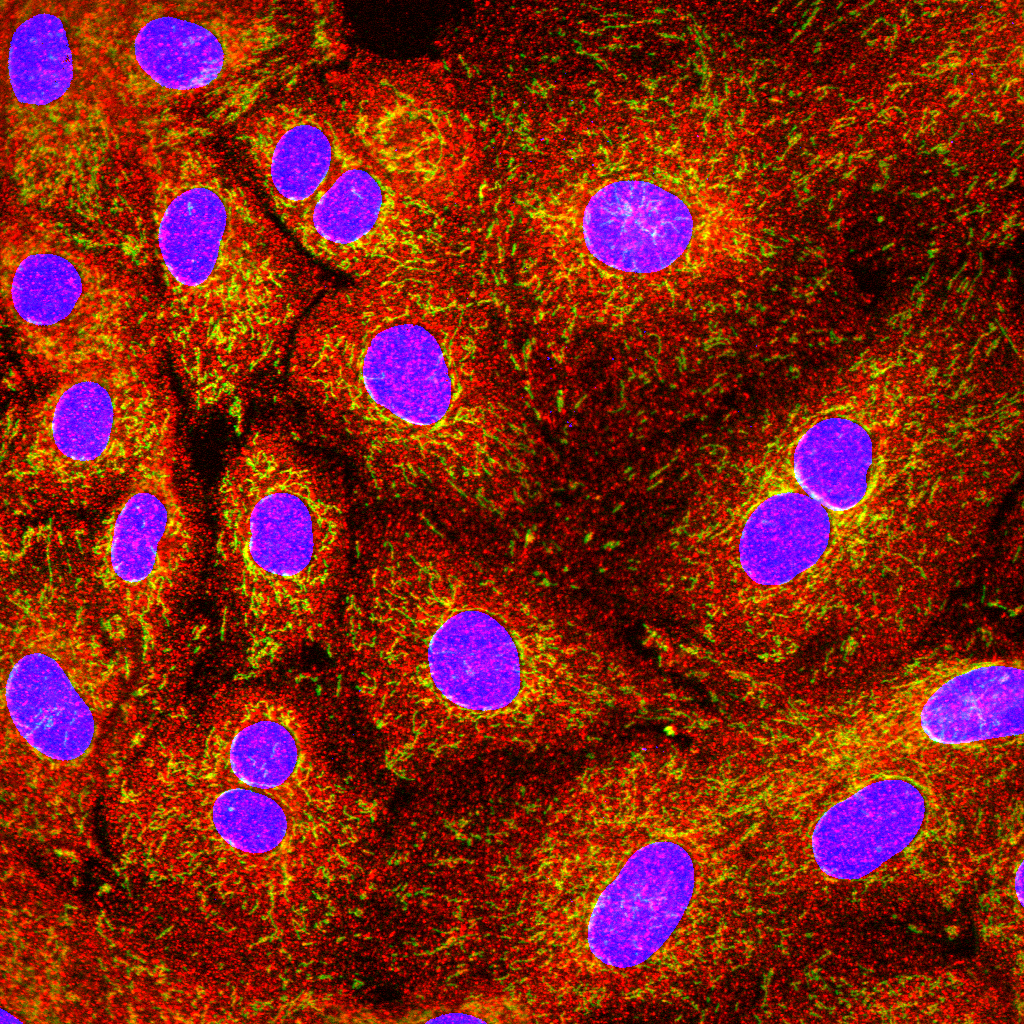

Supplement: Supplementary file 5 — Supplementary material [file mmc5.zip › GK Glucose Time Course - 1st Experiment/E1-G5-5.tif]

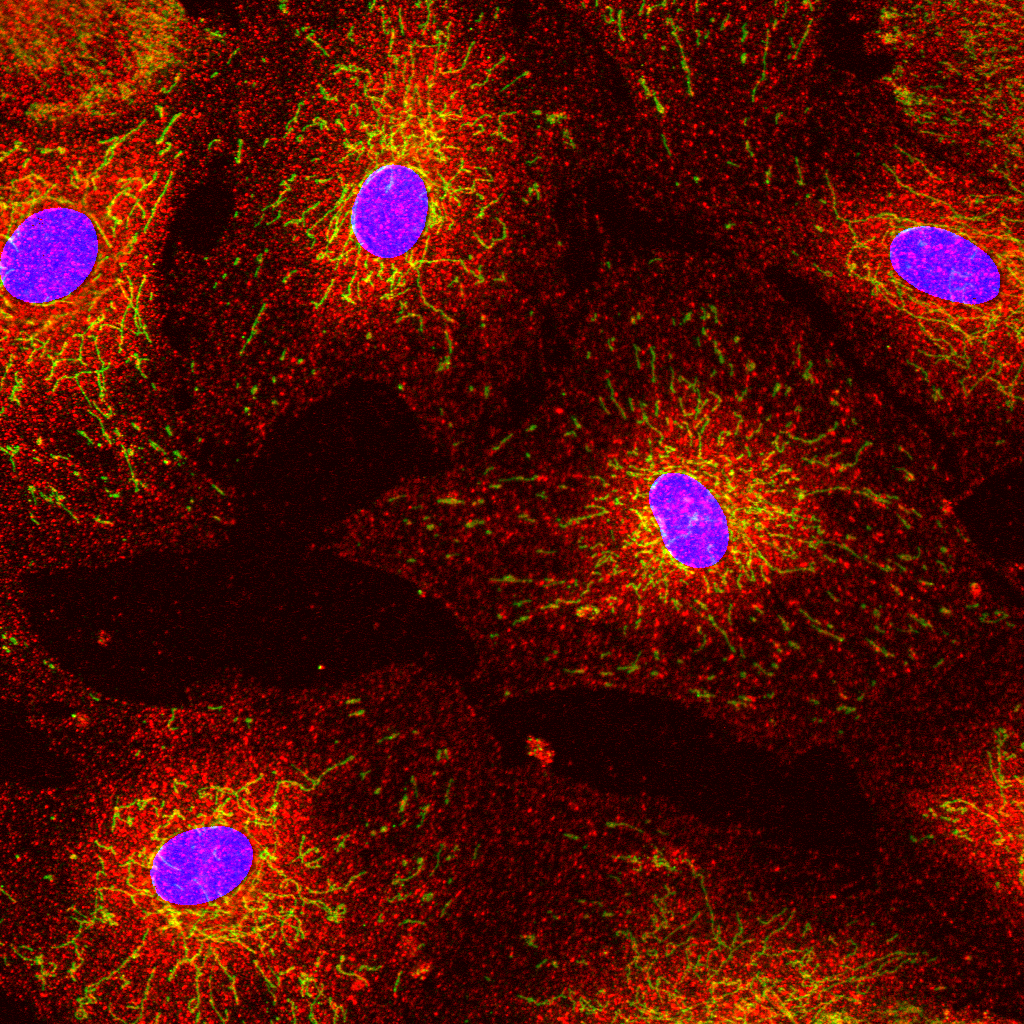

Supplement: Supplementary file 5 — Supplementary material [file mmc5.zip › GK Glucose Time Course - 1st Experiment/E1-G5-6.tif]

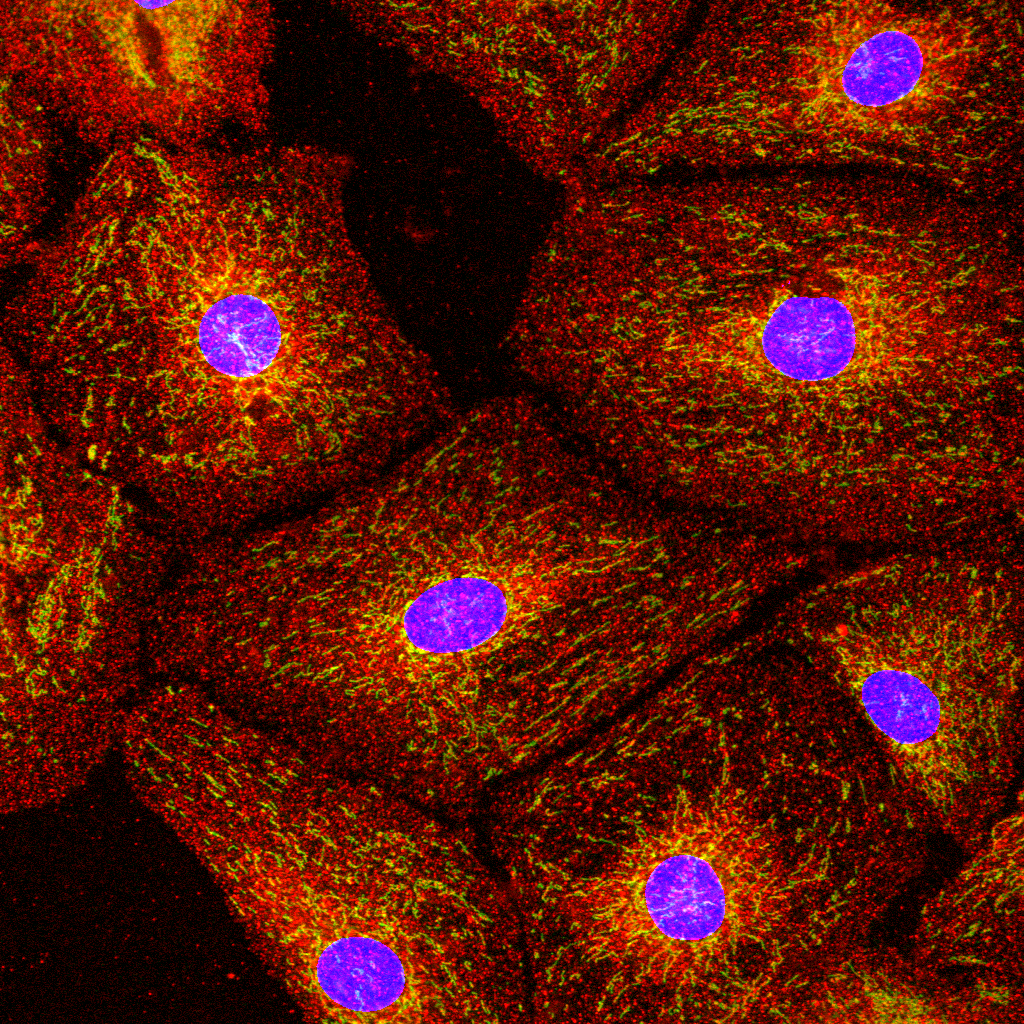

Supplement: Supplementary file 5 — Supplementary material [file mmc5.zip › GK Glucose Time Course - 1st Experiment/E1-G5-7.tif]

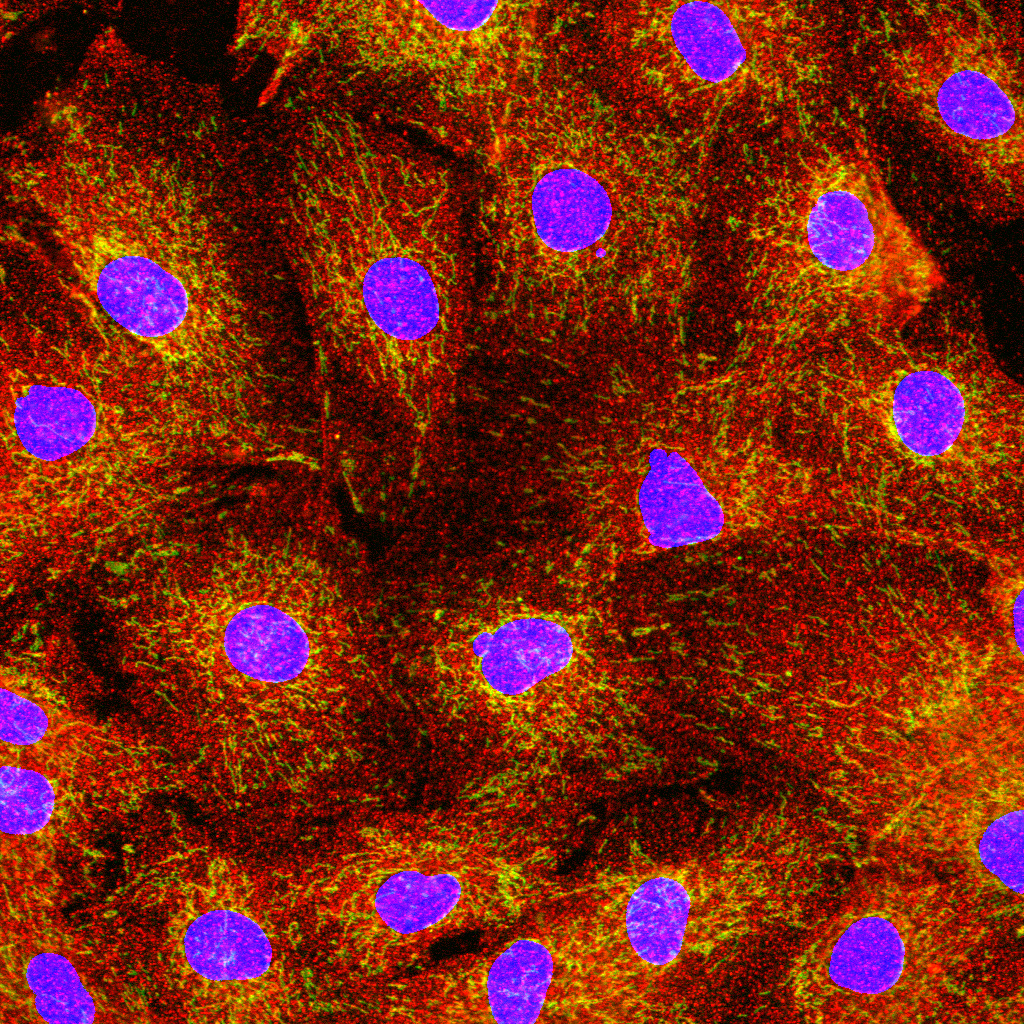

Supplement: Supplementary file 5 — Supplementary material [file mmc5.zip › GK Glucose Time Course - 1st Experiment/E1-G5-8.tif]

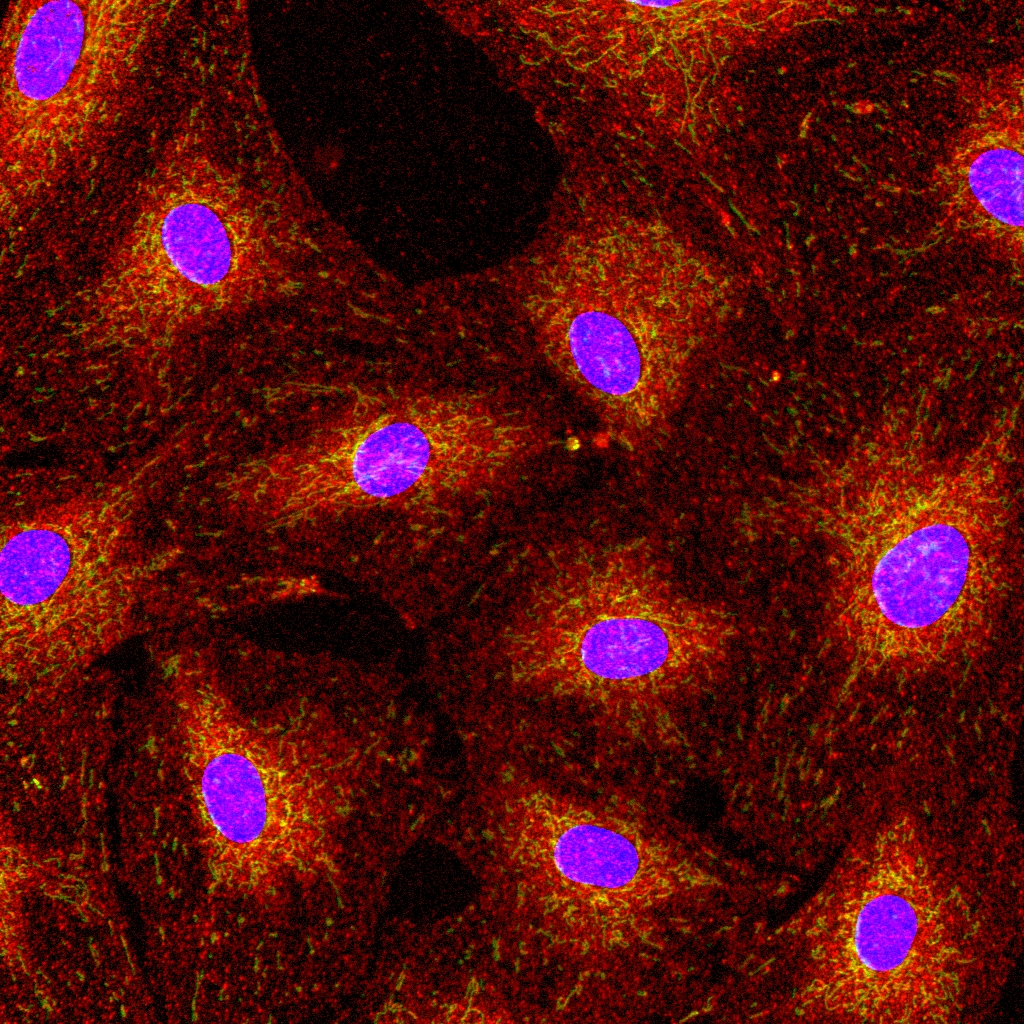

Supplement: Supplementary file 5 — Supplementary material [file mmc5.zip › GK Glucose Time Course - 1st Experiment/E1-G5-9.tif]

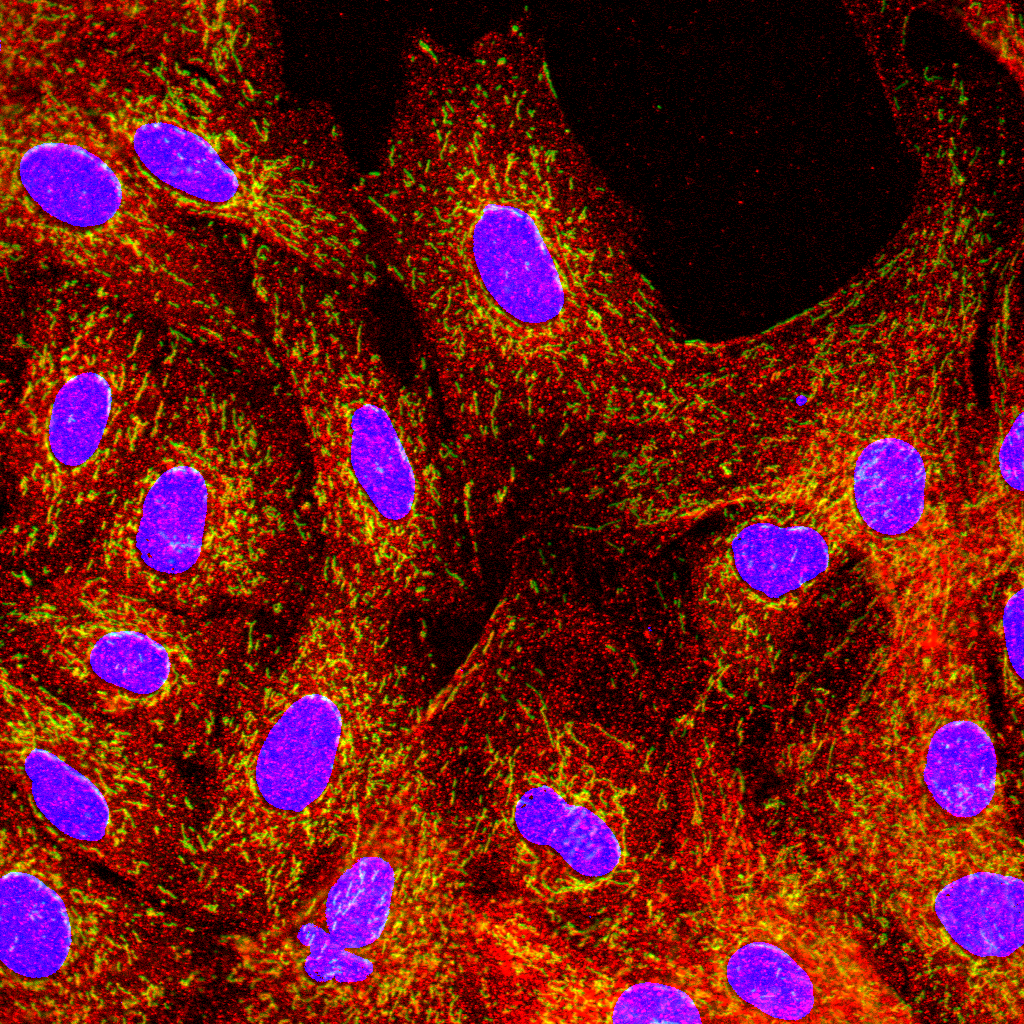

Supplement: Supplementary file 6 — Supplementary material [file mmc6.zip › GK Glucose Time Course - 2nd Experiment/E2-G2-1.tif]

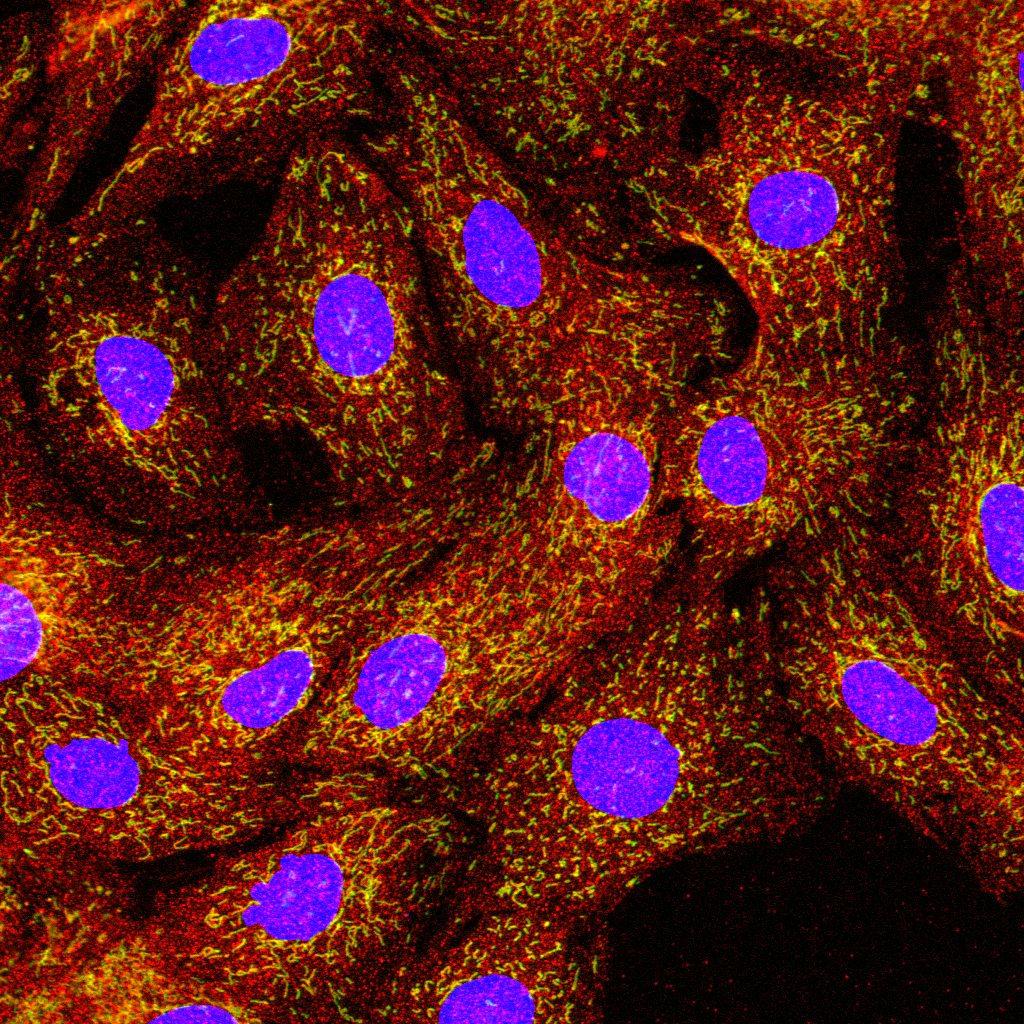

Supplement: Supplementary file 6 — Supplementary material [file mmc6.zip › GK Glucose Time Course - 2nd Experiment/E2-G2-2.tif]

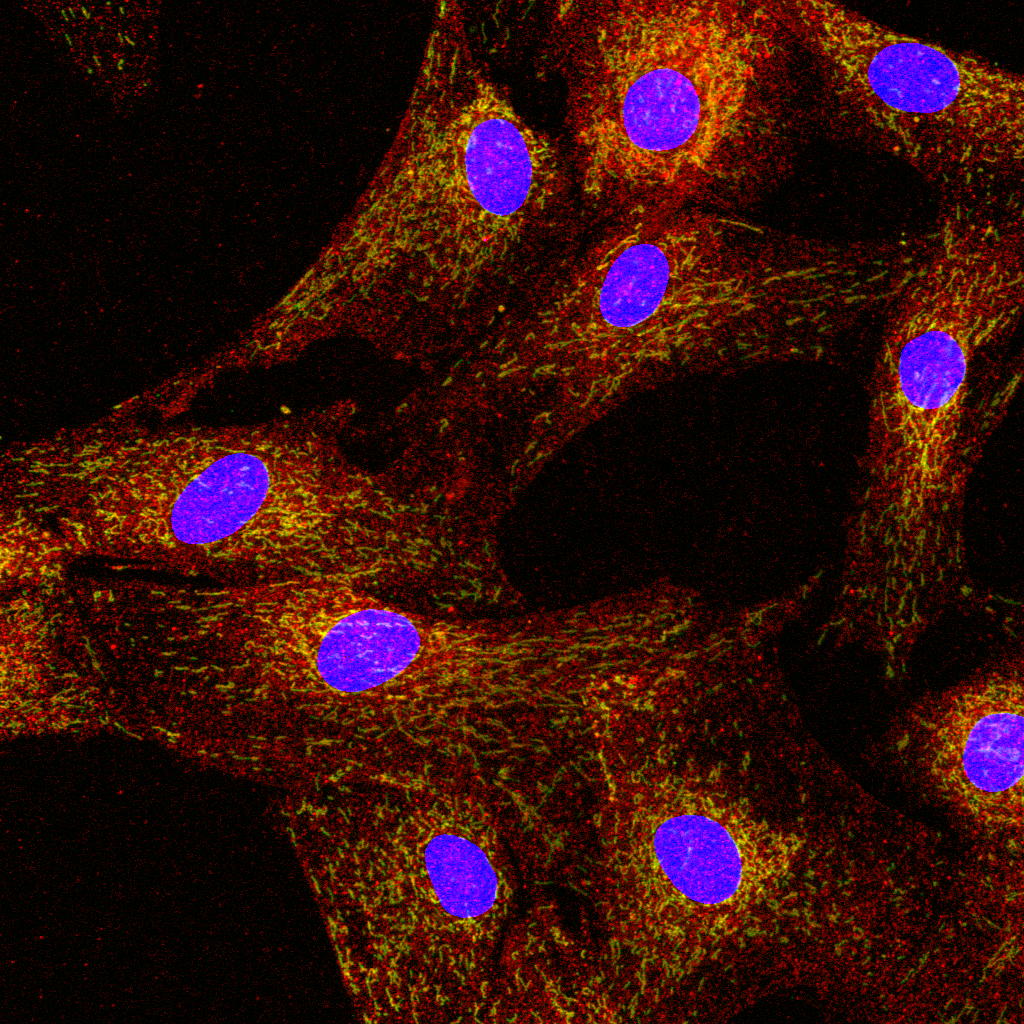

Supplement: Supplementary file 6 — Supplementary material [file mmc6.zip › GK Glucose Time Course - 2nd Experiment/E2-G2-3.tif]

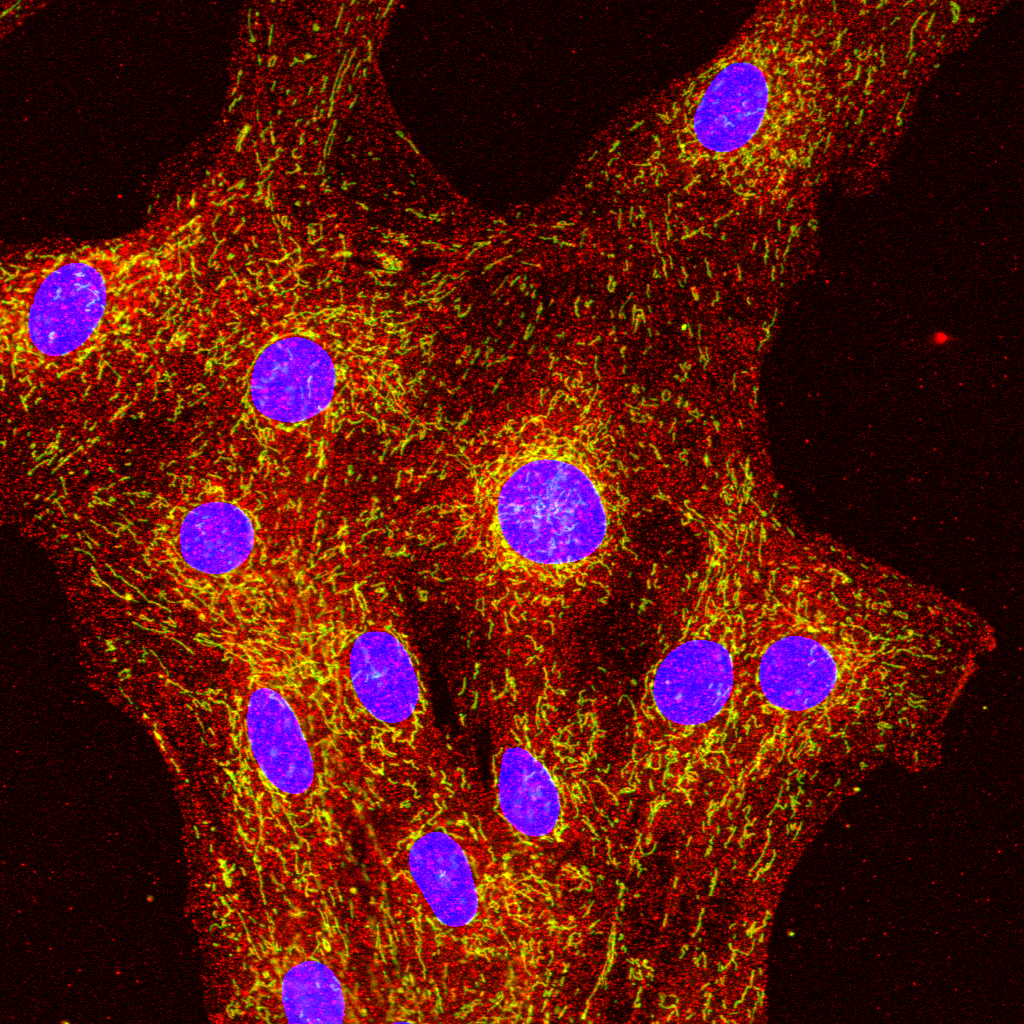

Supplement: Supplementary file 6 — Supplementary material [file mmc6.zip › GK Glucose Time Course - 2nd Experiment/E2-G2-4.tif]

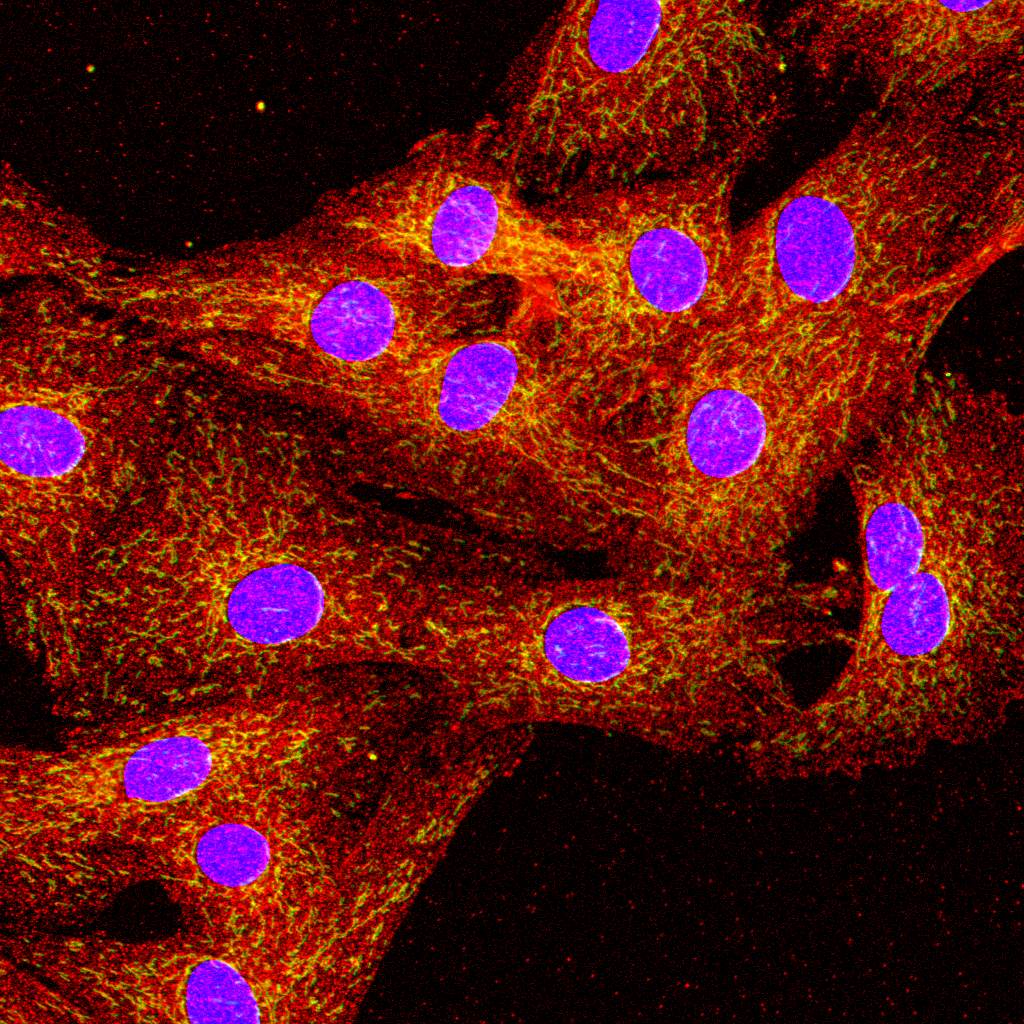

Supplement: Supplementary file 6 — Supplementary material [file mmc6.zip › GK Glucose Time Course - 2nd Experiment/E2-G2-5.tif]

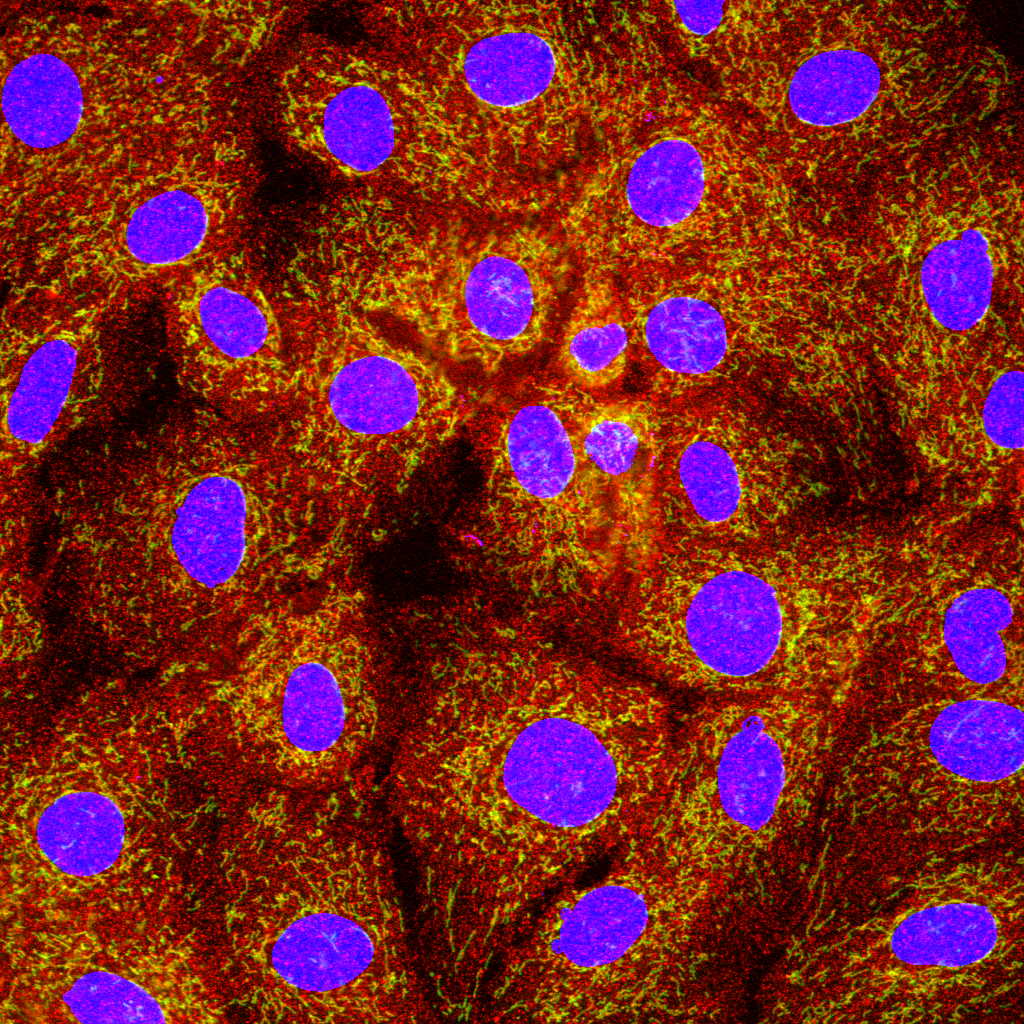

Supplement: Supplementary file 6 — Supplementary material [file mmc6.zip › GK Glucose Time Course - 2nd Experiment/E2-G2-6.tif]

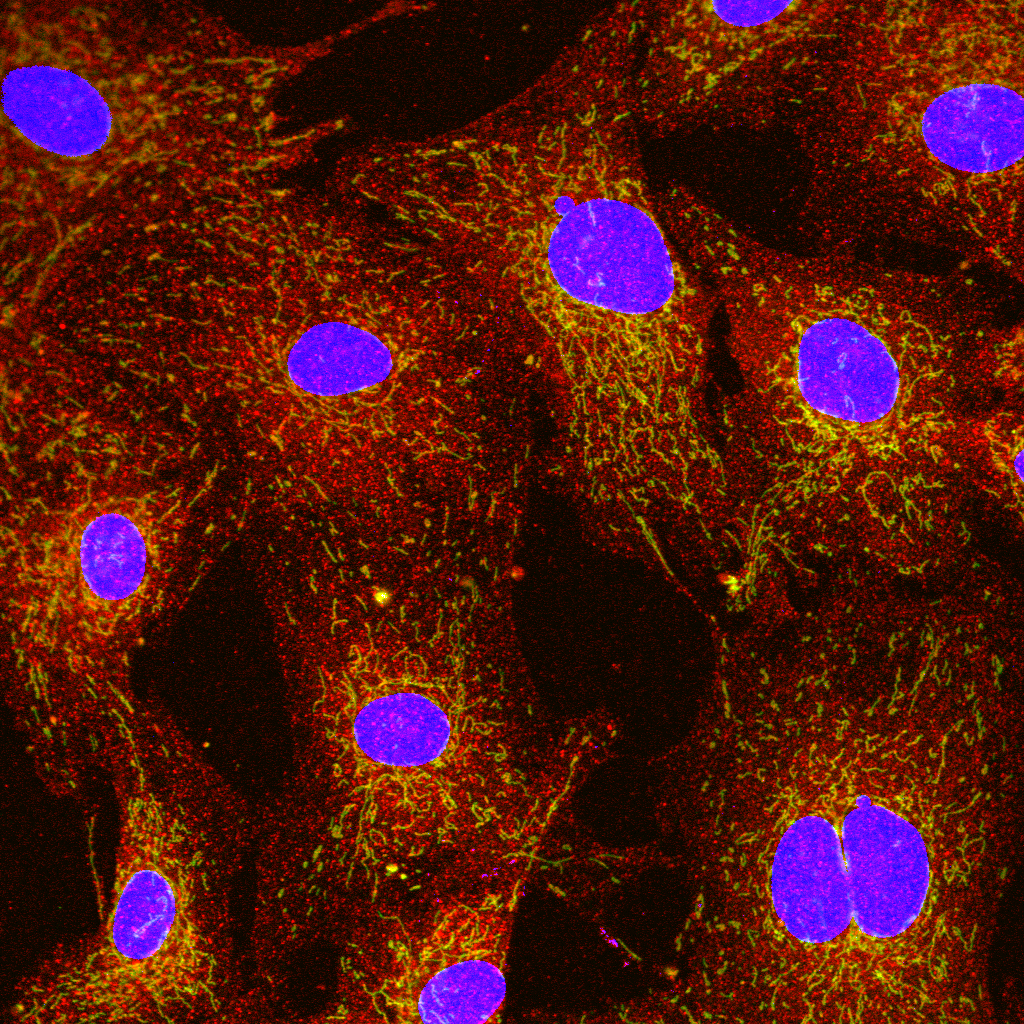

Supplement: Supplementary file 6 — Supplementary material [file mmc6.zip › GK Glucose Time Course - 2nd Experiment/E2-G2-7.tif]

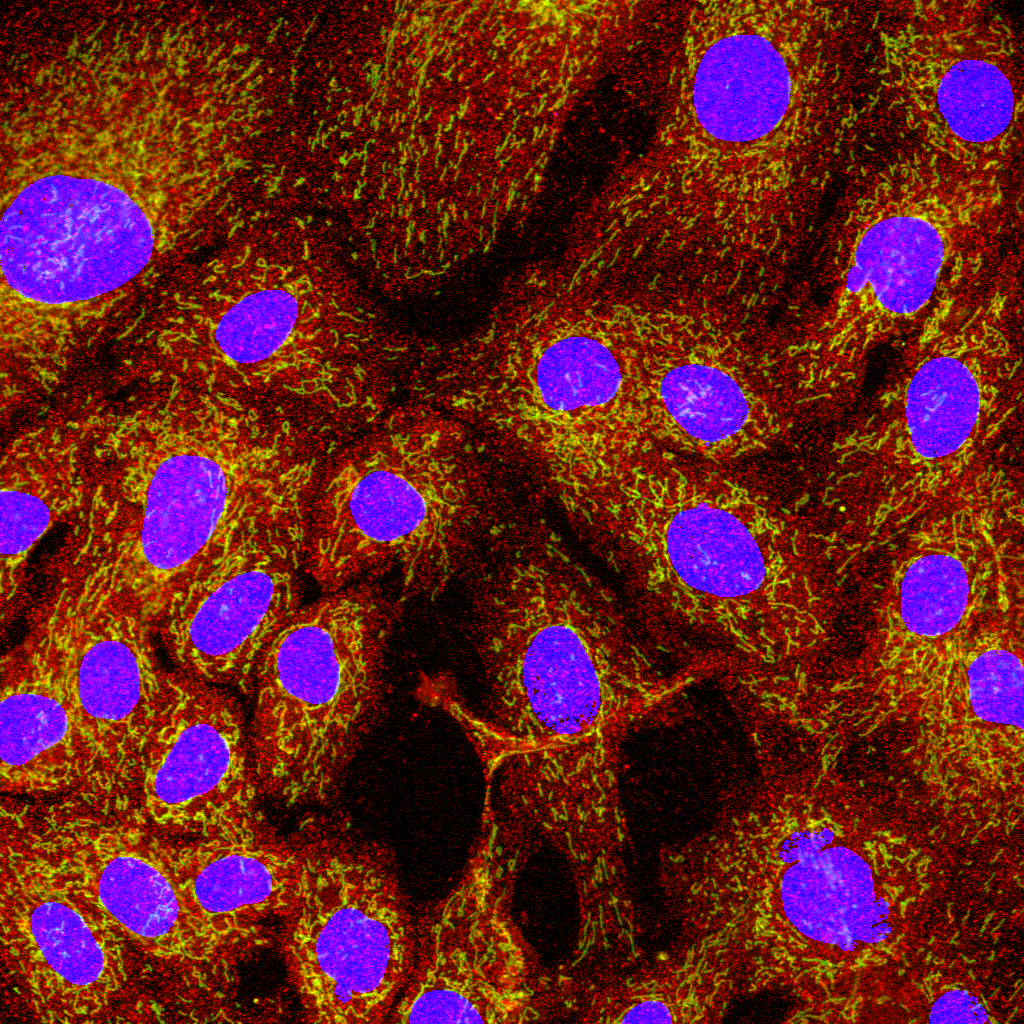

Supplement: Supplementary file 6 — Supplementary material [file mmc6.zip › GK Glucose Time Course - 2nd Experiment/E2-G2-8.tif]

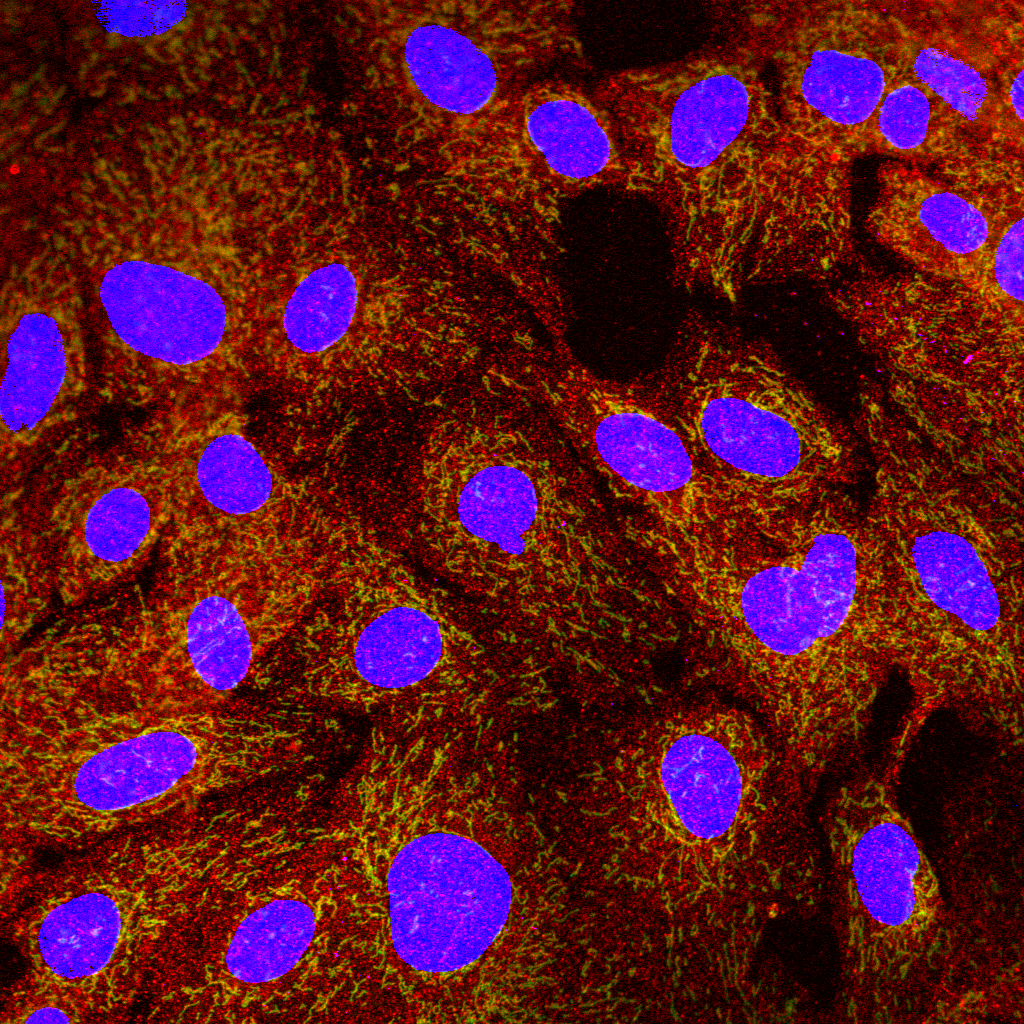

Supplement: Supplementary file 6 — Supplementary material [file mmc6.zip › GK Glucose Time Course - 2nd Experiment/E2-G2-9.tif]

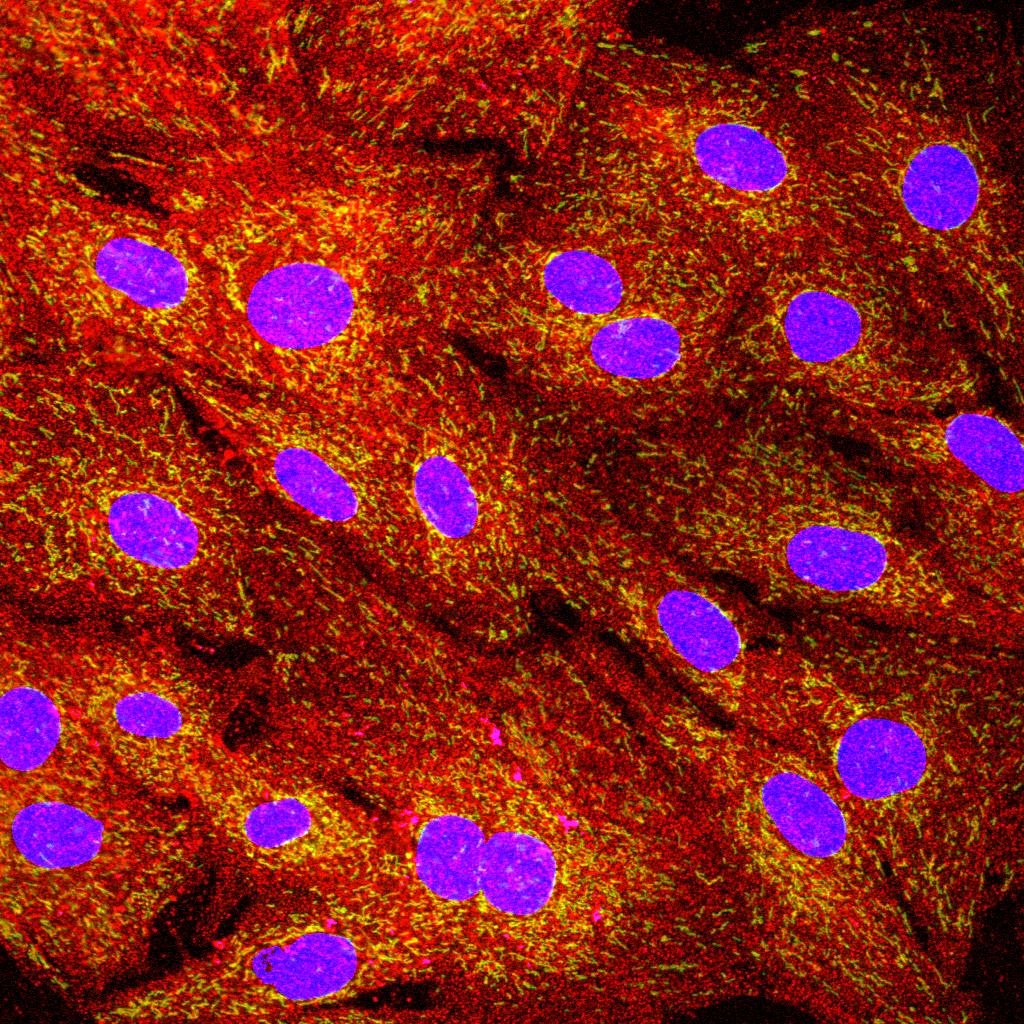

Supplement: Supplementary file 6 — Supplementary material [file mmc6.zip › GK Glucose Time Course - 2nd Experiment/E2-G3-1.tif]

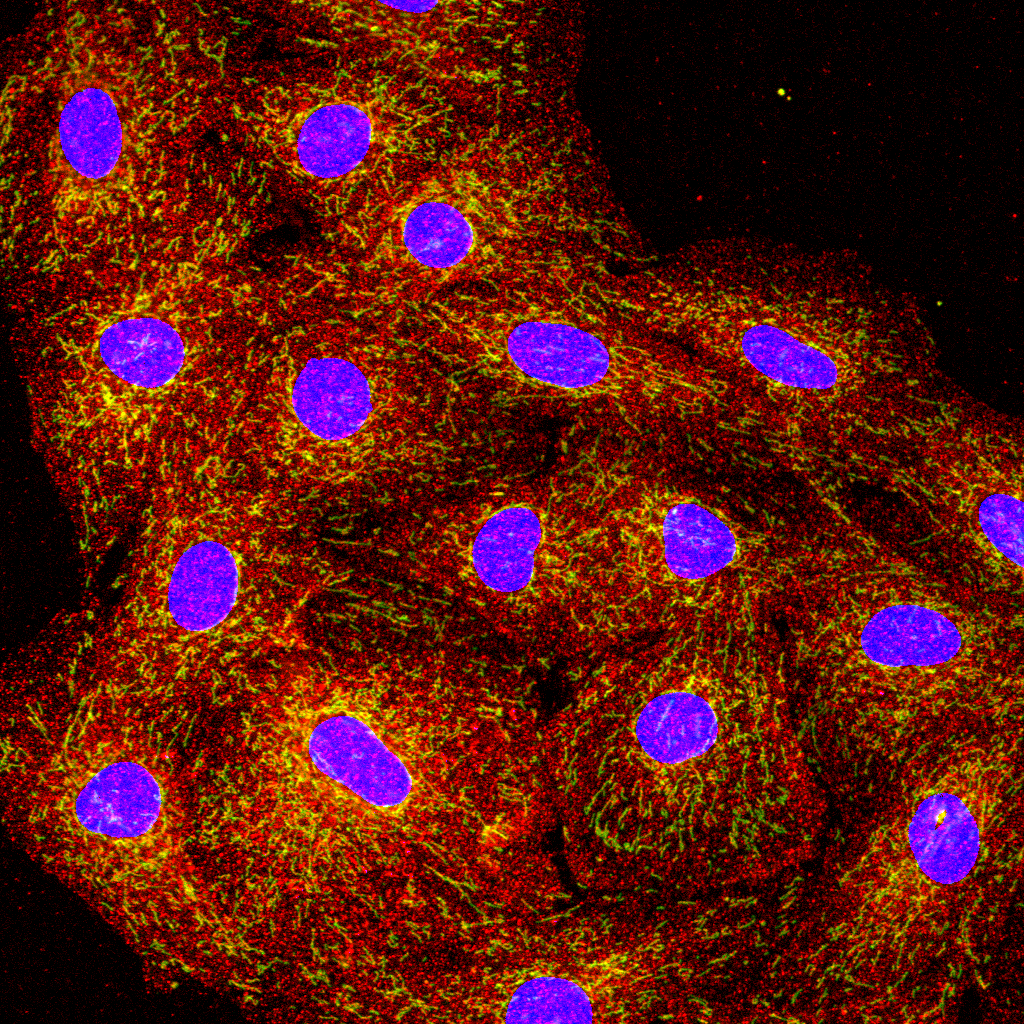

Supplement: Supplementary file 6 — Supplementary material [file mmc6.zip › GK Glucose Time Course - 2nd Experiment/E2-G3-2.tif]

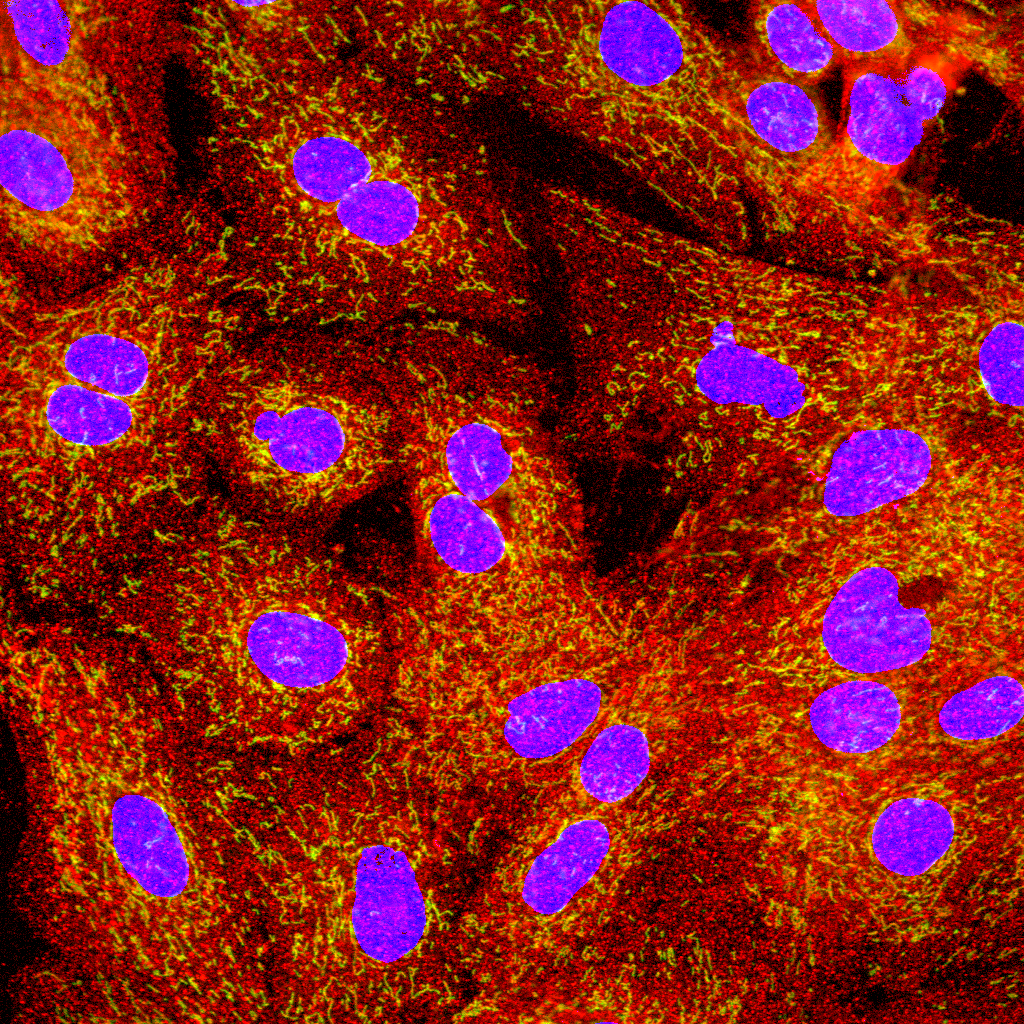

Supplement: Supplementary file 6 — Supplementary material [file mmc6.zip › GK Glucose Time Course - 2nd Experiment/E2-G3-3.tif]

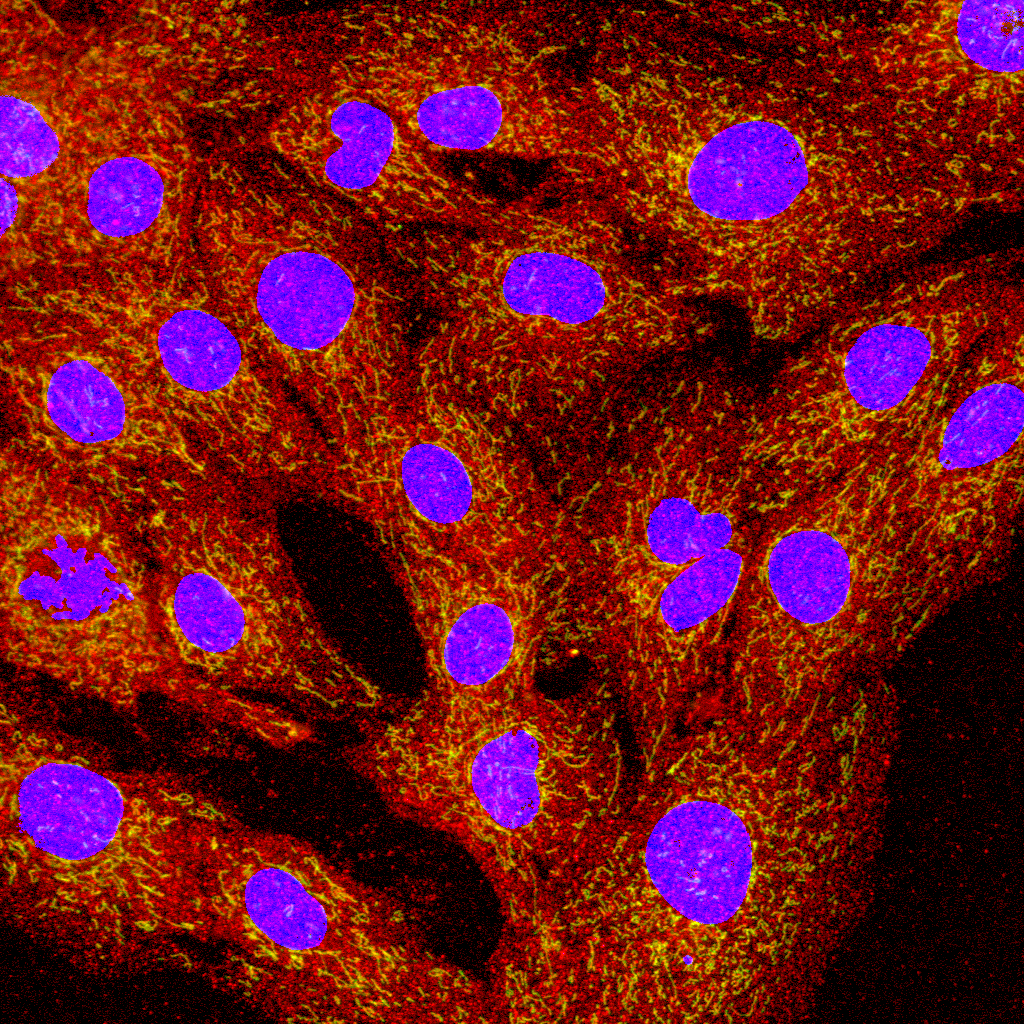

Supplement: Supplementary file 6 — Supplementary material [file mmc6.zip › GK Glucose Time Course - 2nd Experiment/E2-G3-4.tif]

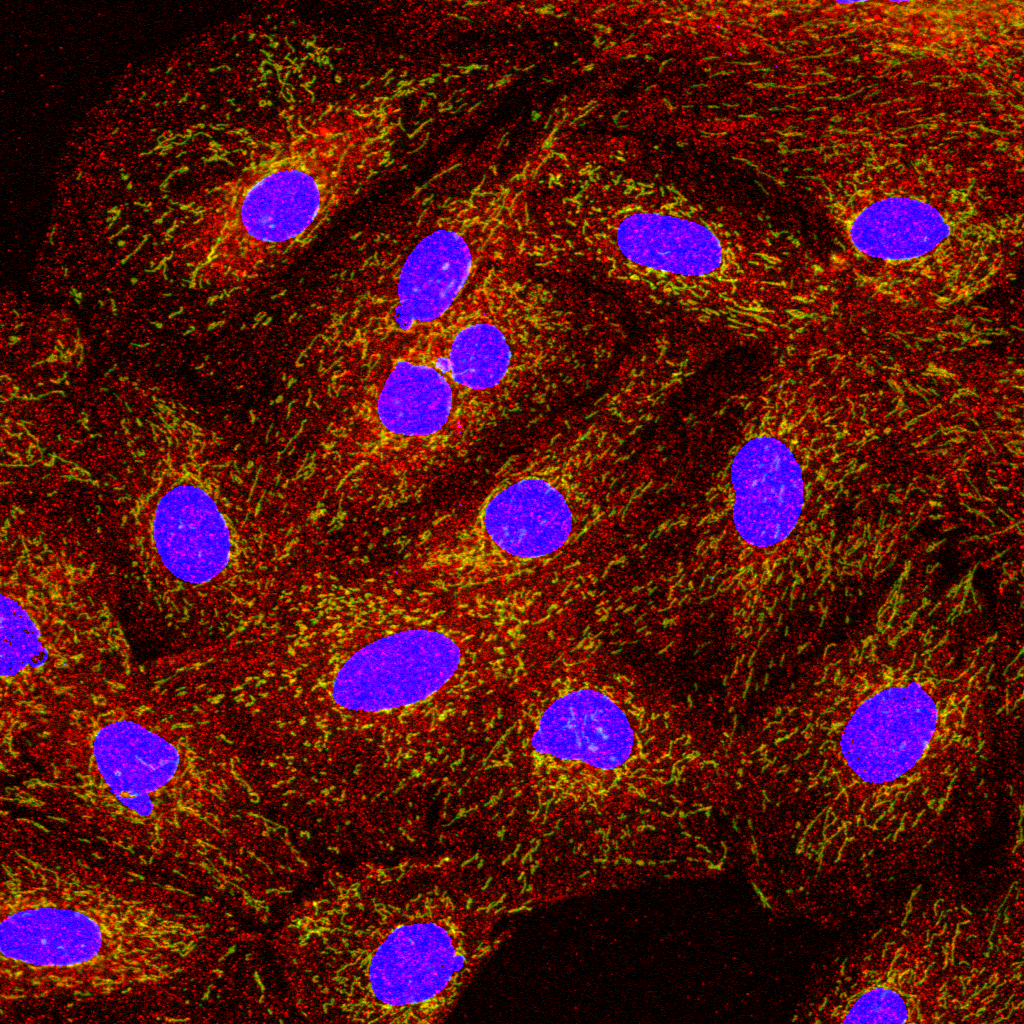

Supplement: Supplementary file 6 — Supplementary material [file mmc6.zip › GK Glucose Time Course - 2nd Experiment/E2-G3-5.tif]

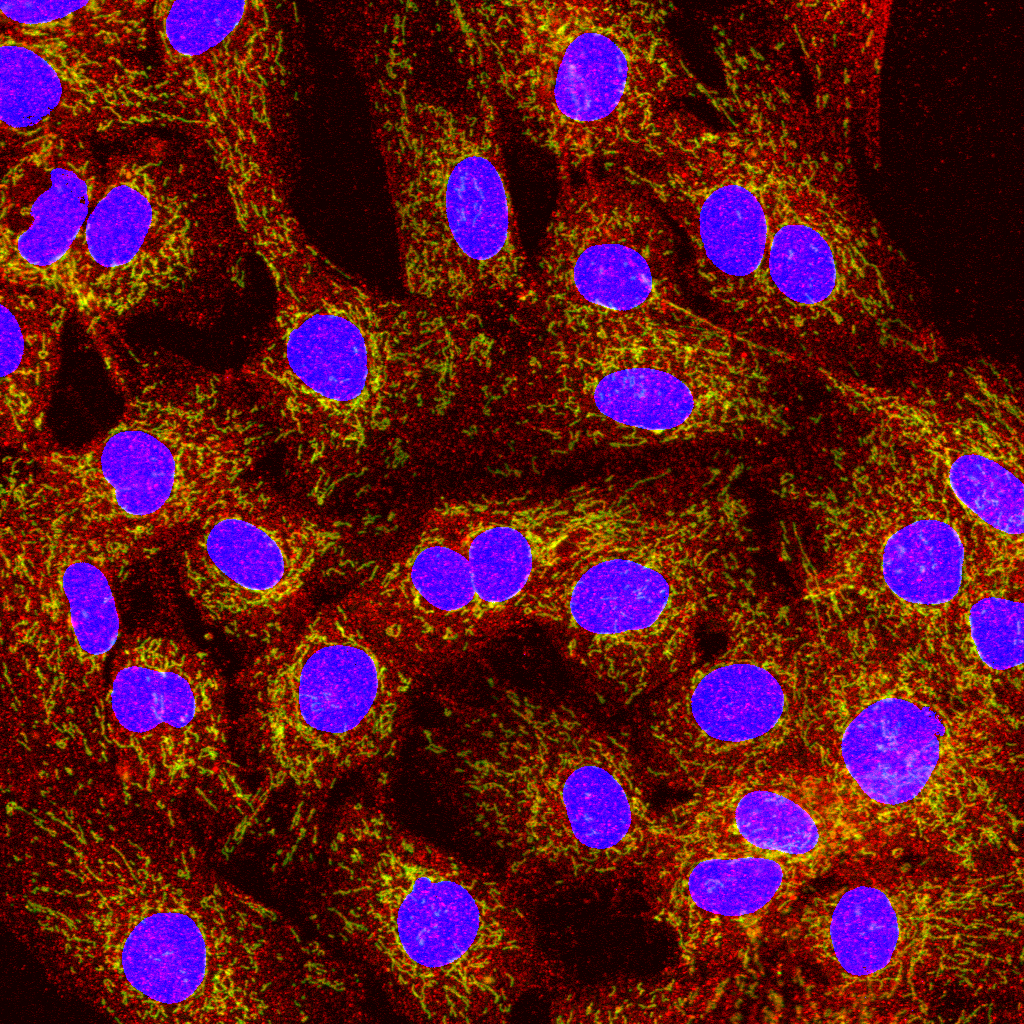

Supplement: Supplementary file 6 — Supplementary material [file mmc6.zip › GK Glucose Time Course - 2nd Experiment/E2-G3-6.tif]

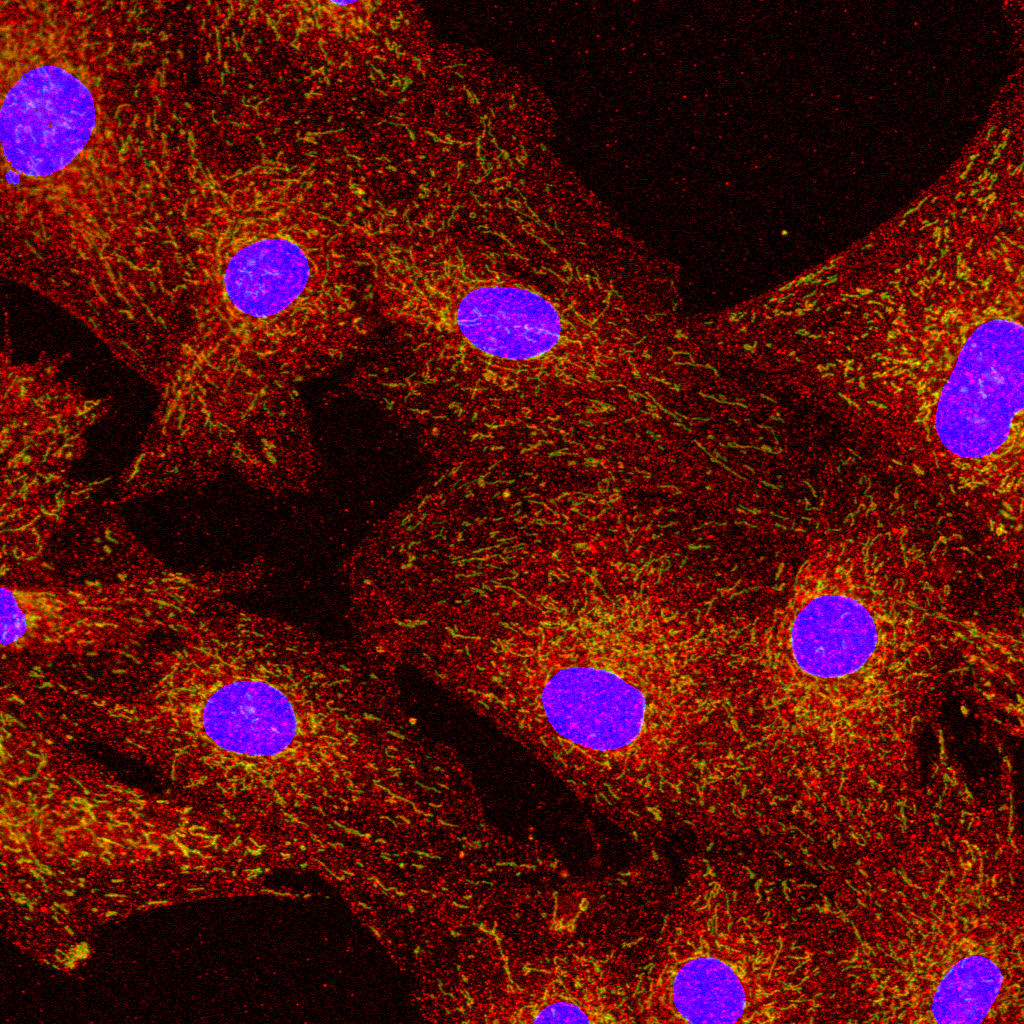

Supplement: Supplementary file 6 — Supplementary material [file mmc6.zip › GK Glucose Time Course - 2nd Experiment/E2-G3-7.tif]

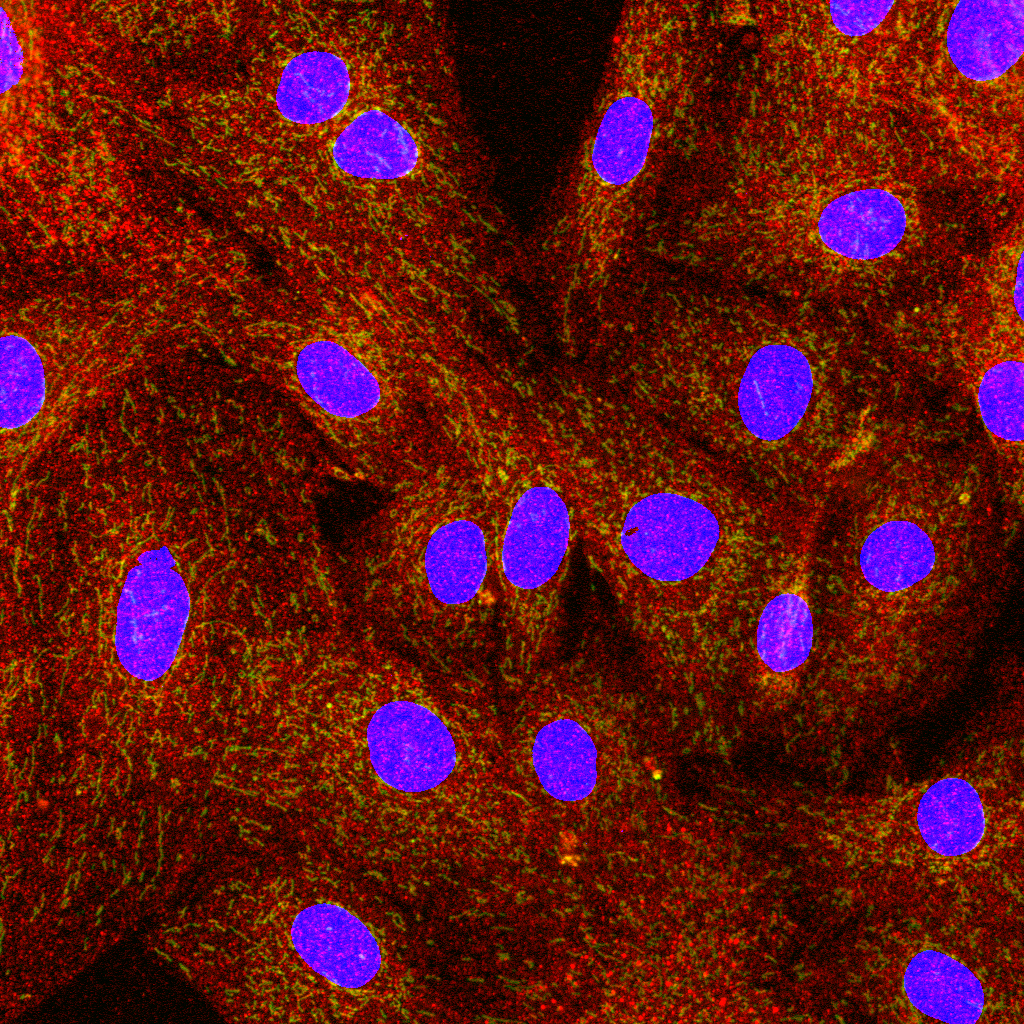

Supplement: Supplementary file 6 — Supplementary material [file mmc6.zip › GK Glucose Time Course - 2nd Experiment/E2-G3-8.tif]

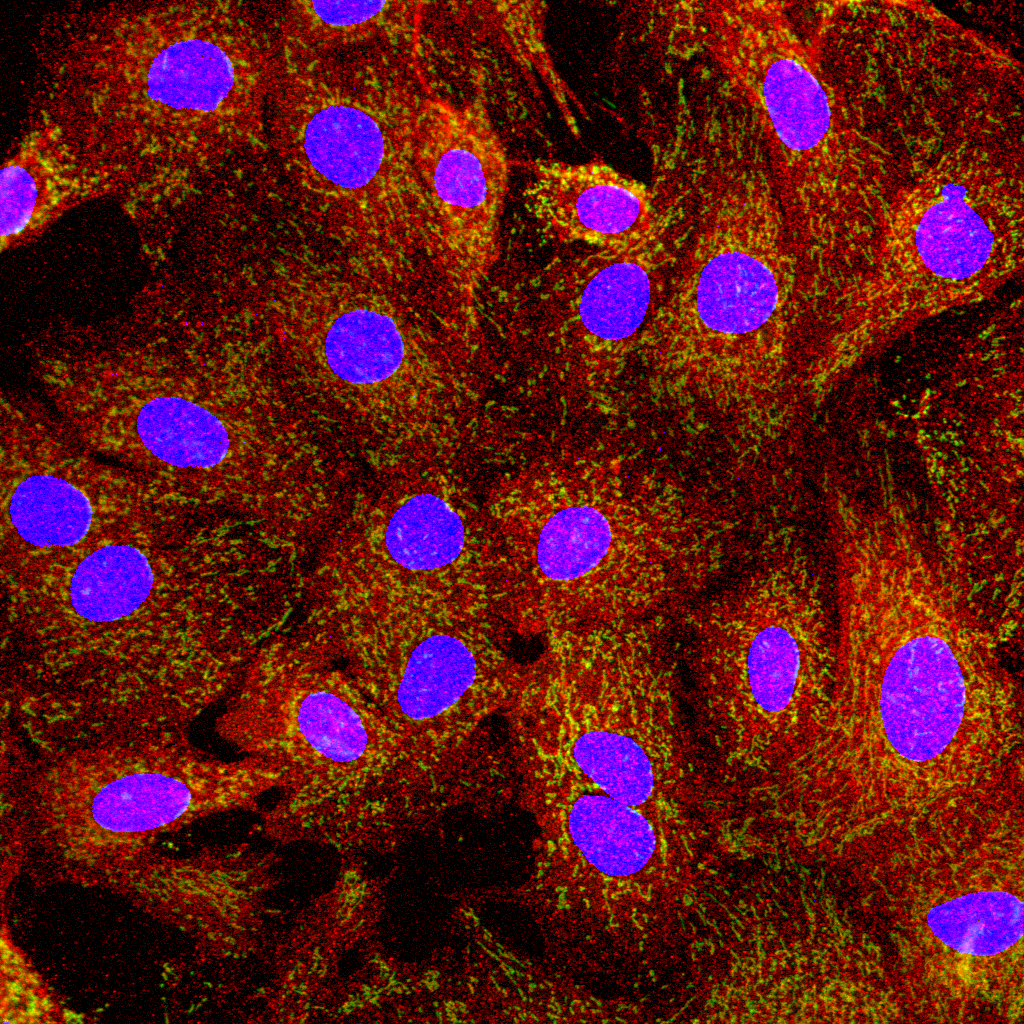

Supplement: Supplementary file 6 — Supplementary material [file mmc6.zip › GK Glucose Time Course - 2nd Experiment/E2-G3-9.tif]

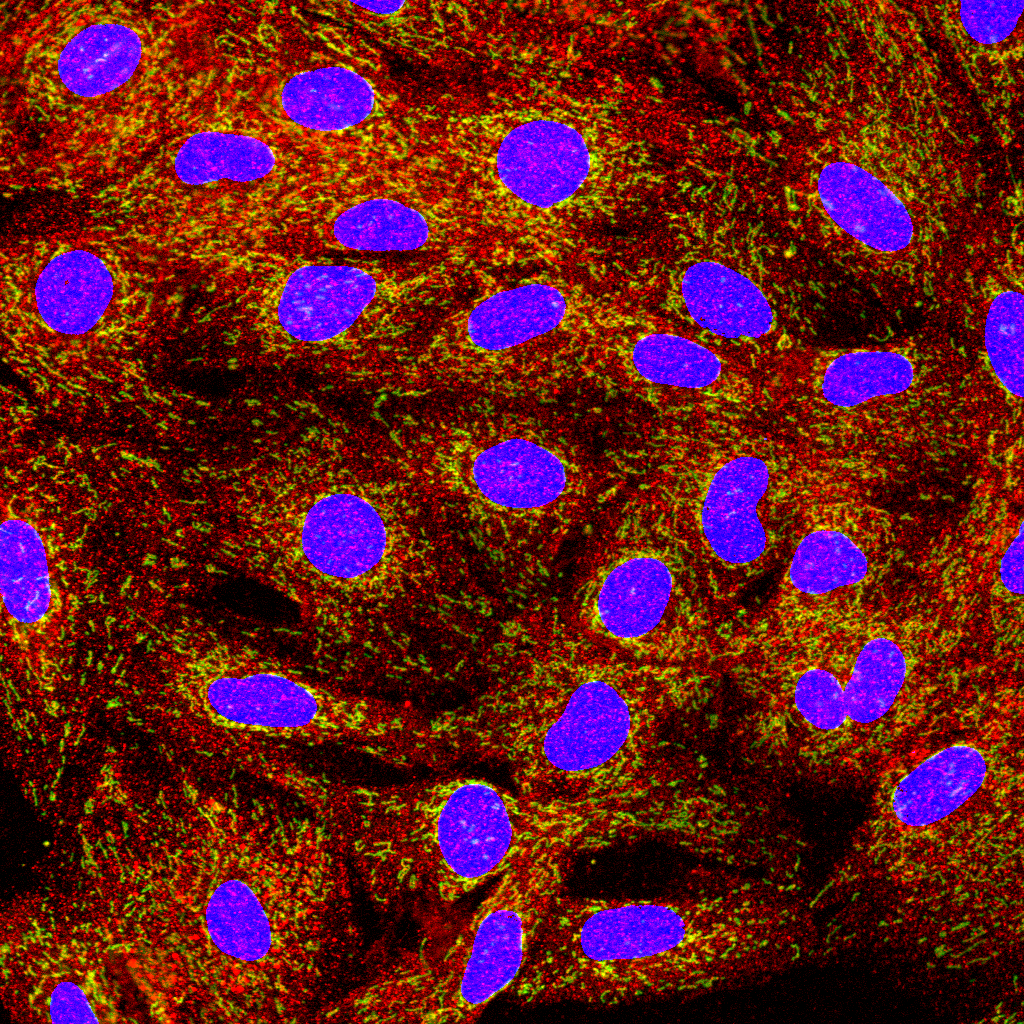

Supplement: Supplementary file 6 — Supplementary material [file mmc6.zip › GK Glucose Time Course - 2nd Experiment/E2-G4-1.tif]

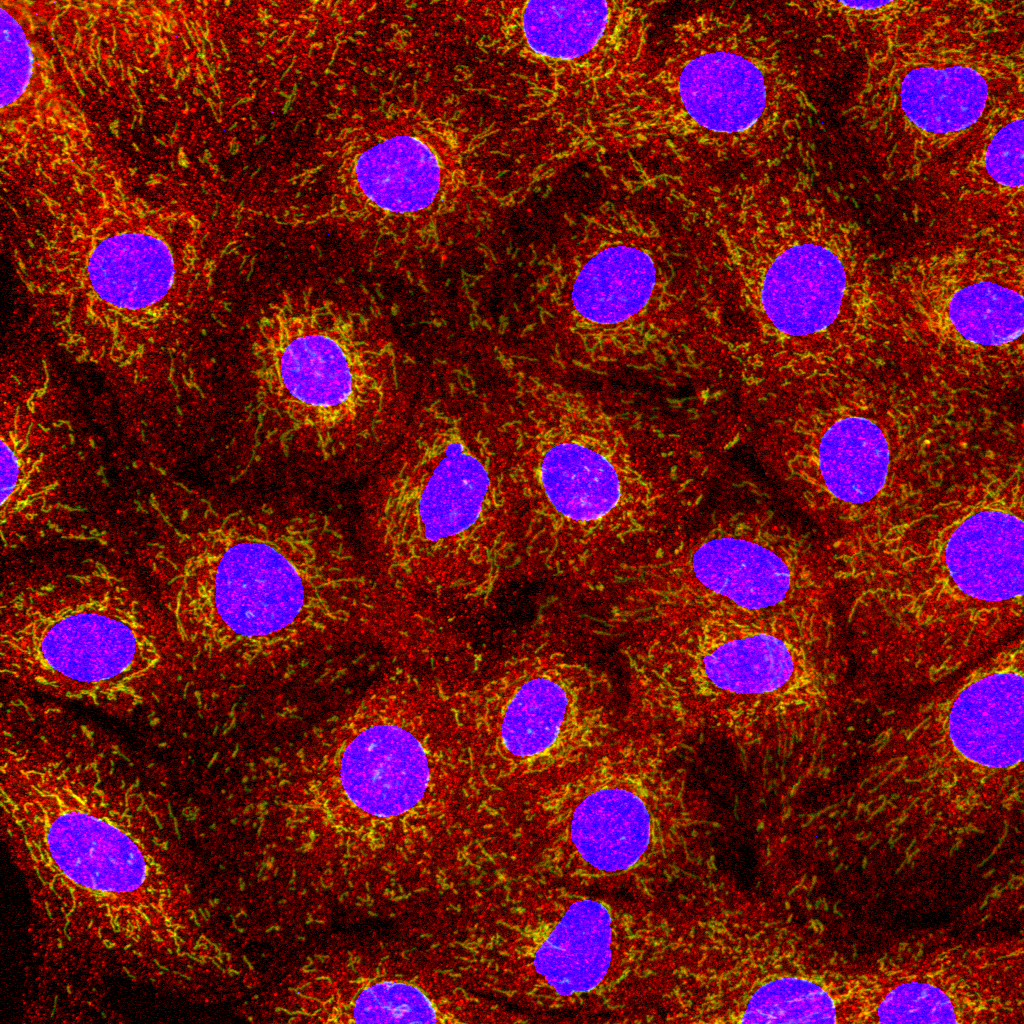

Supplement: Supplementary file 6 — Supplementary material [file mmc6.zip › GK Glucose Time Course - 2nd Experiment/E2-G4-2.tif]

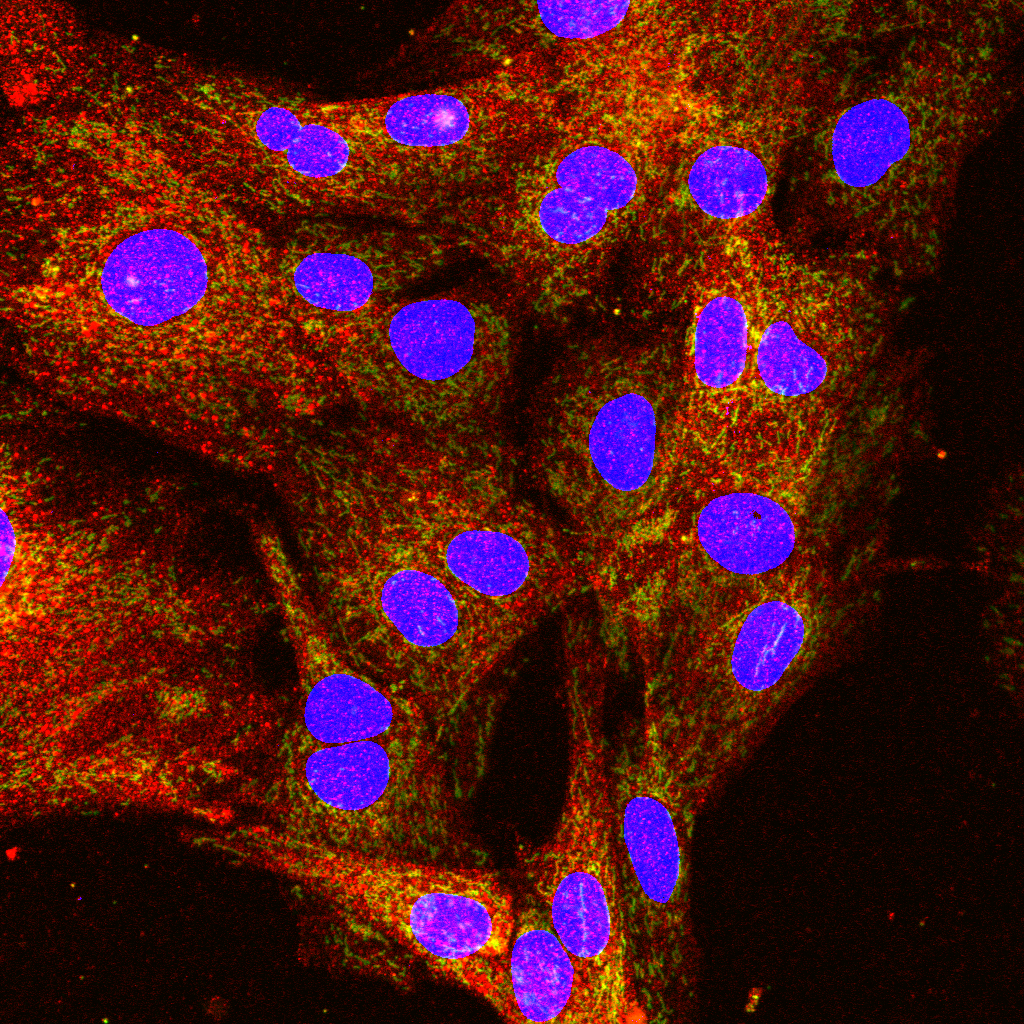

Supplement: Supplementary file 6 — Supplementary material [file mmc6.zip › GK Glucose Time Course - 2nd Experiment/E2-G4-3.tif]

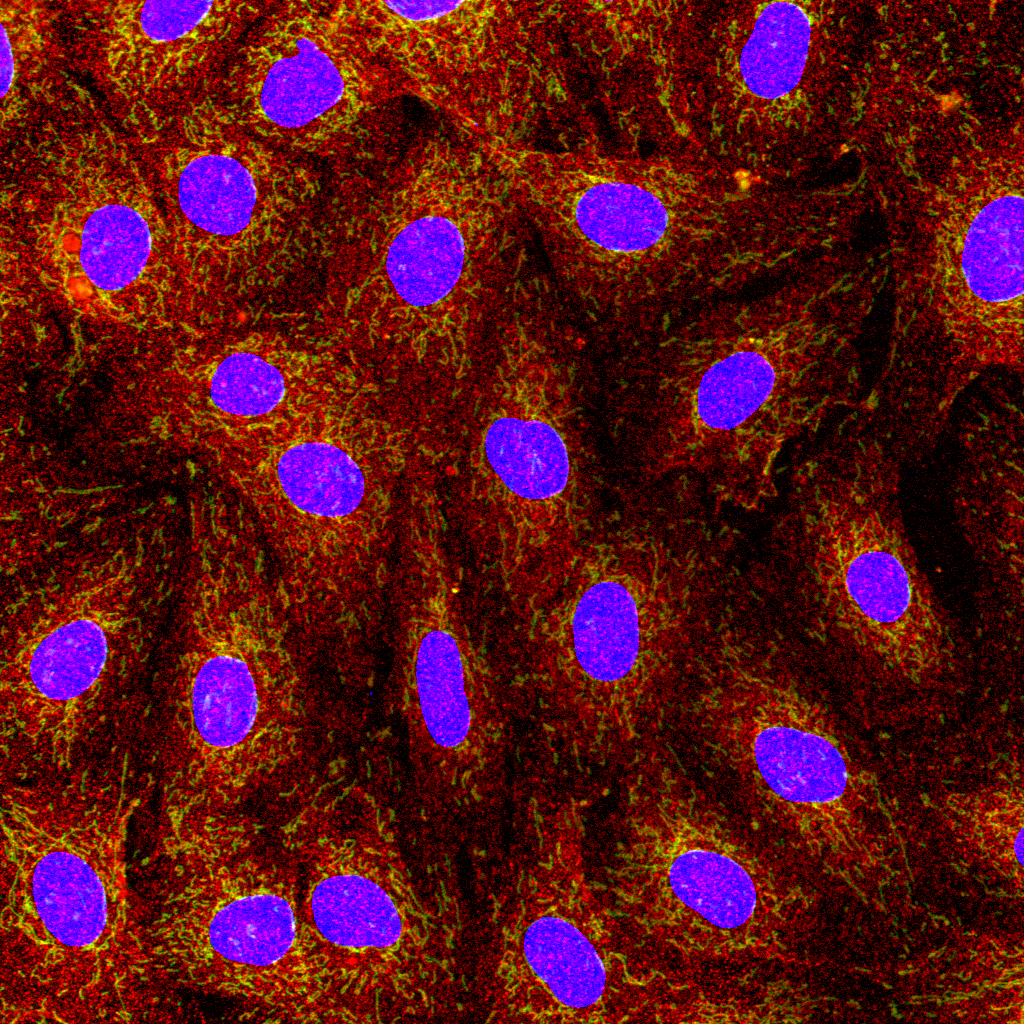

Supplement: Supplementary file 6 — Supplementary material [file mmc6.zip › GK Glucose Time Course - 2nd Experiment/E2-G4-4.tif]

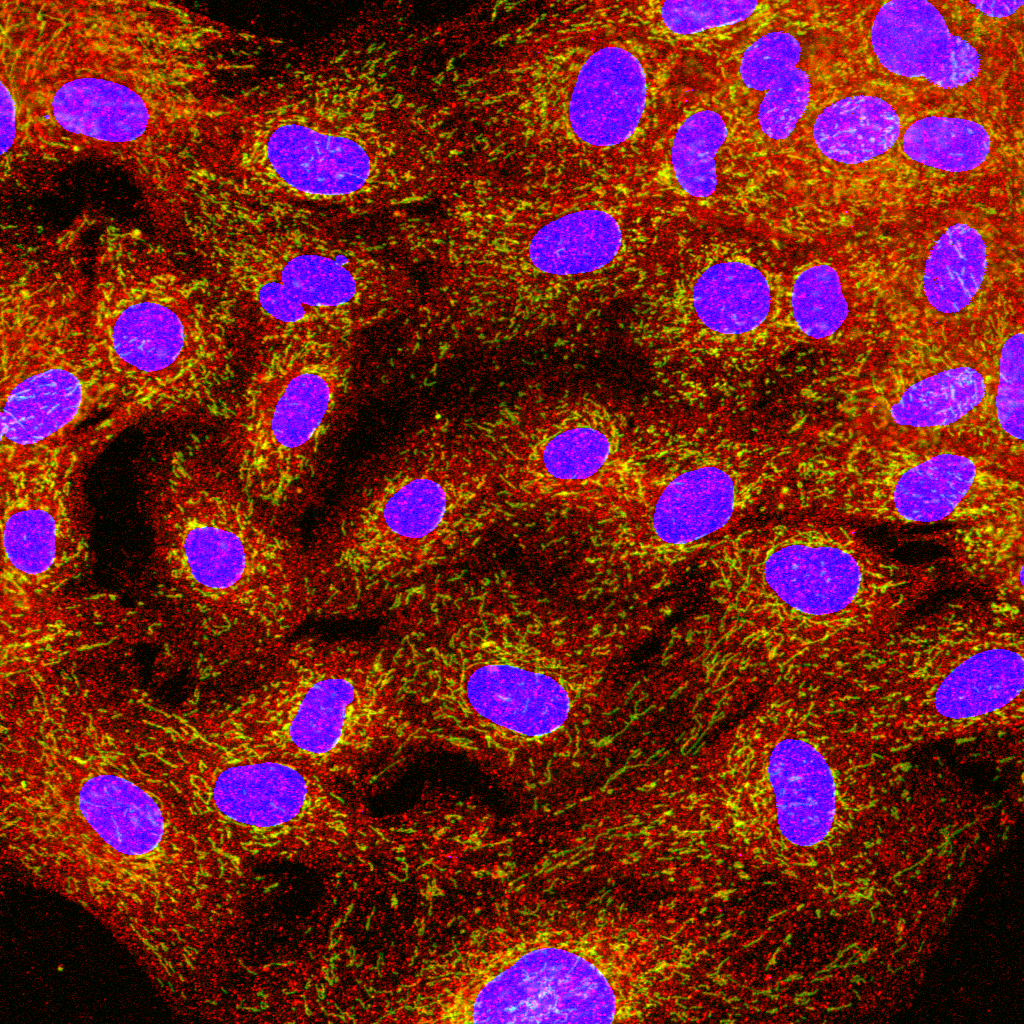

Supplement: Supplementary file 6 — Supplementary material [file mmc6.zip › GK Glucose Time Course - 2nd Experiment/E2-G4-5.tif]

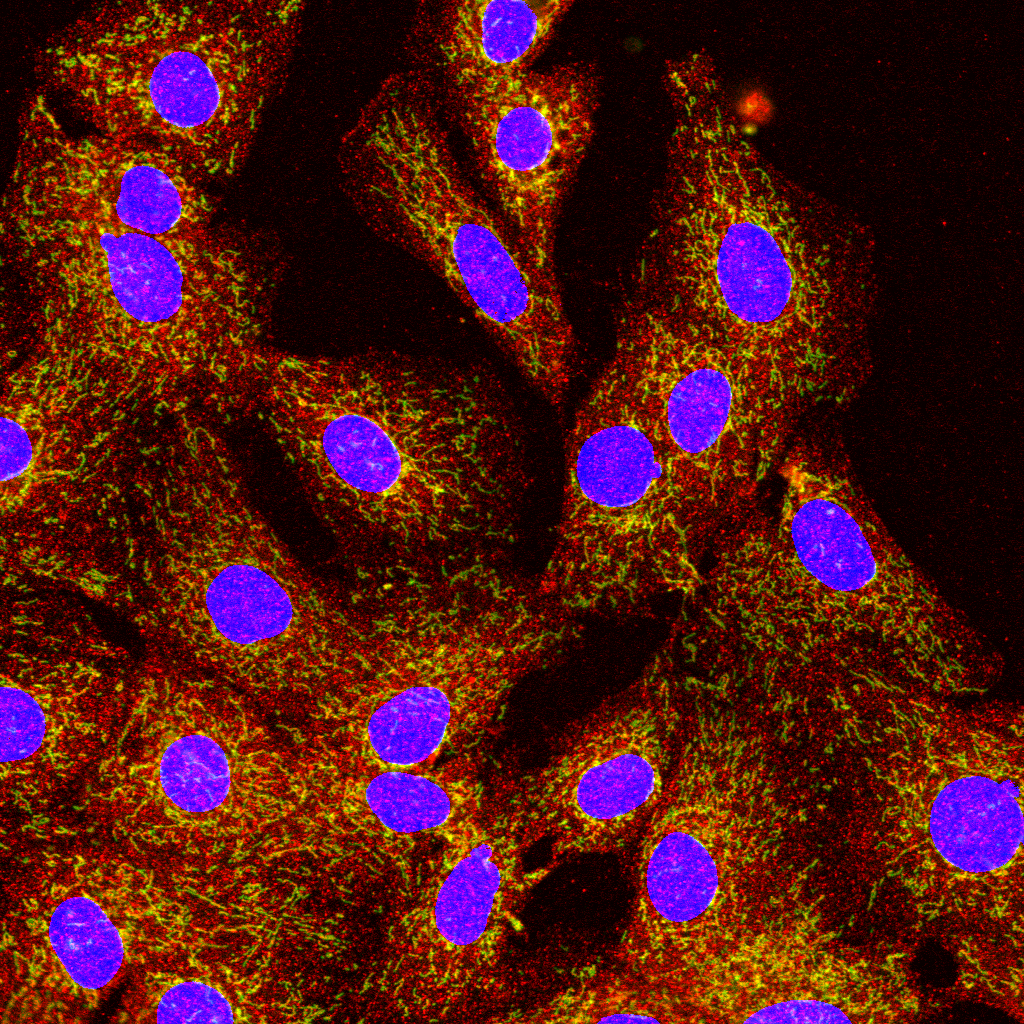

Supplement: Supplementary file 6 — Supplementary material [file mmc6.zip › GK Glucose Time Course - 2nd Experiment/E2-G4-6.tif]

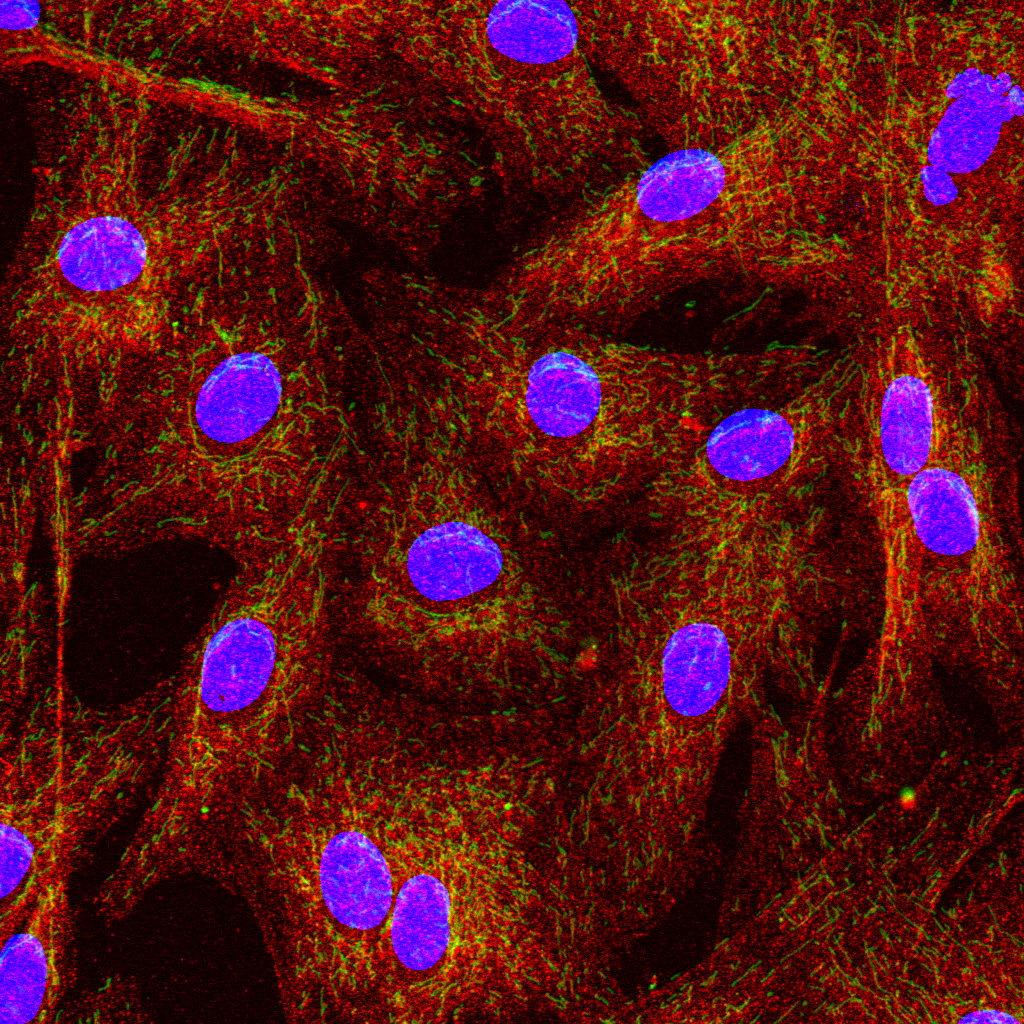

Supplement: Supplementary file 6 — Supplementary material [file mmc6.zip › GK Glucose Time Course - 2nd Experiment/E2-G4-7.tif]

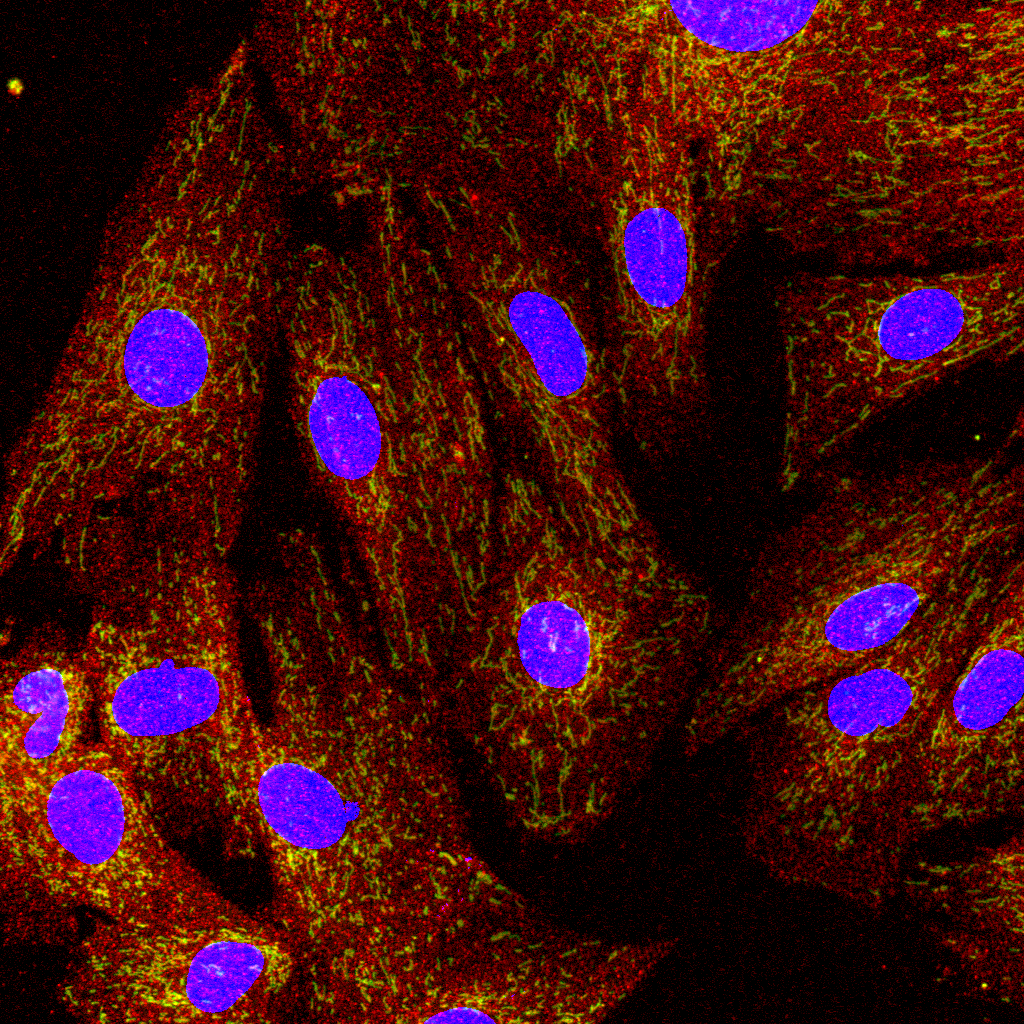

Supplement: Supplementary file 6 — Supplementary material [file mmc6.zip › GK Glucose Time Course - 2nd Experiment/E2-G4-8.tif]

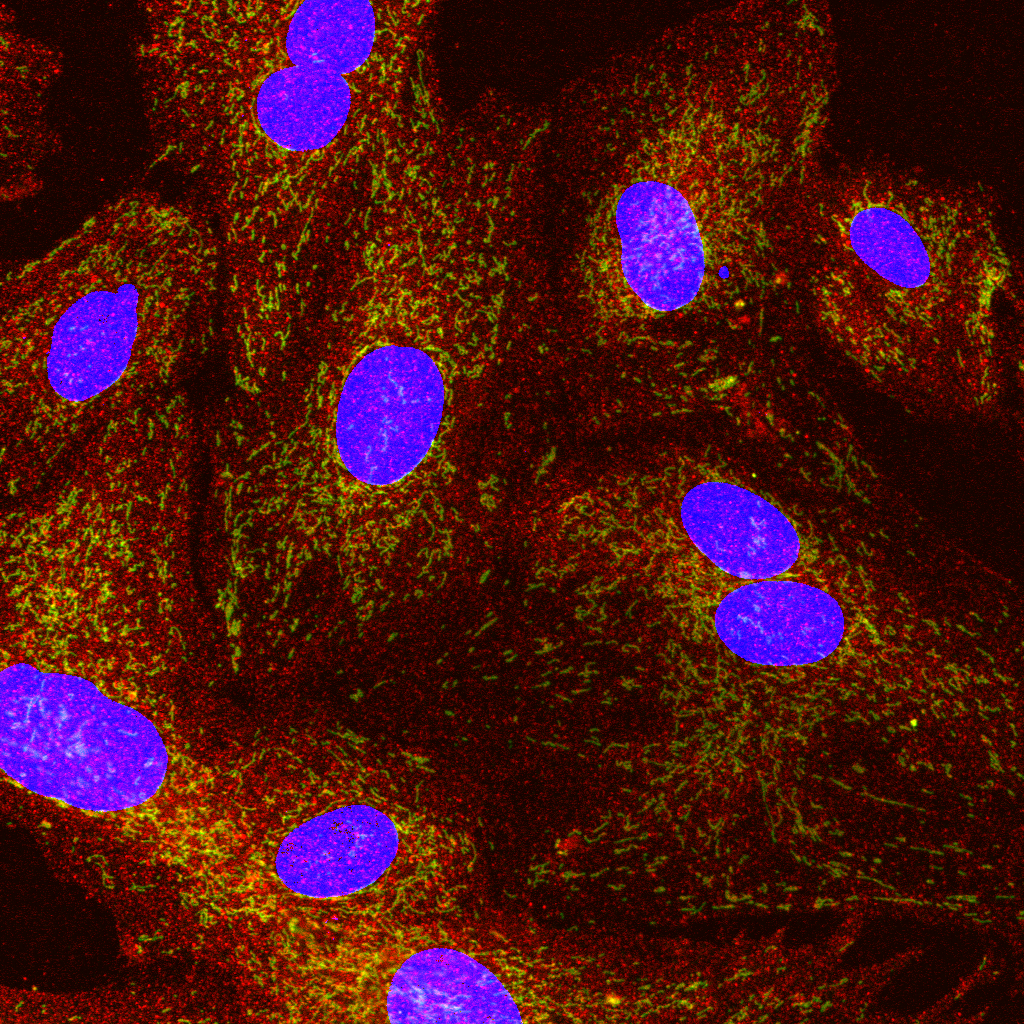

Supplement: Supplementary file 6 — Supplementary material [file mmc6.zip › GK Glucose Time Course - 2nd Experiment/E2-G4-9.tif]

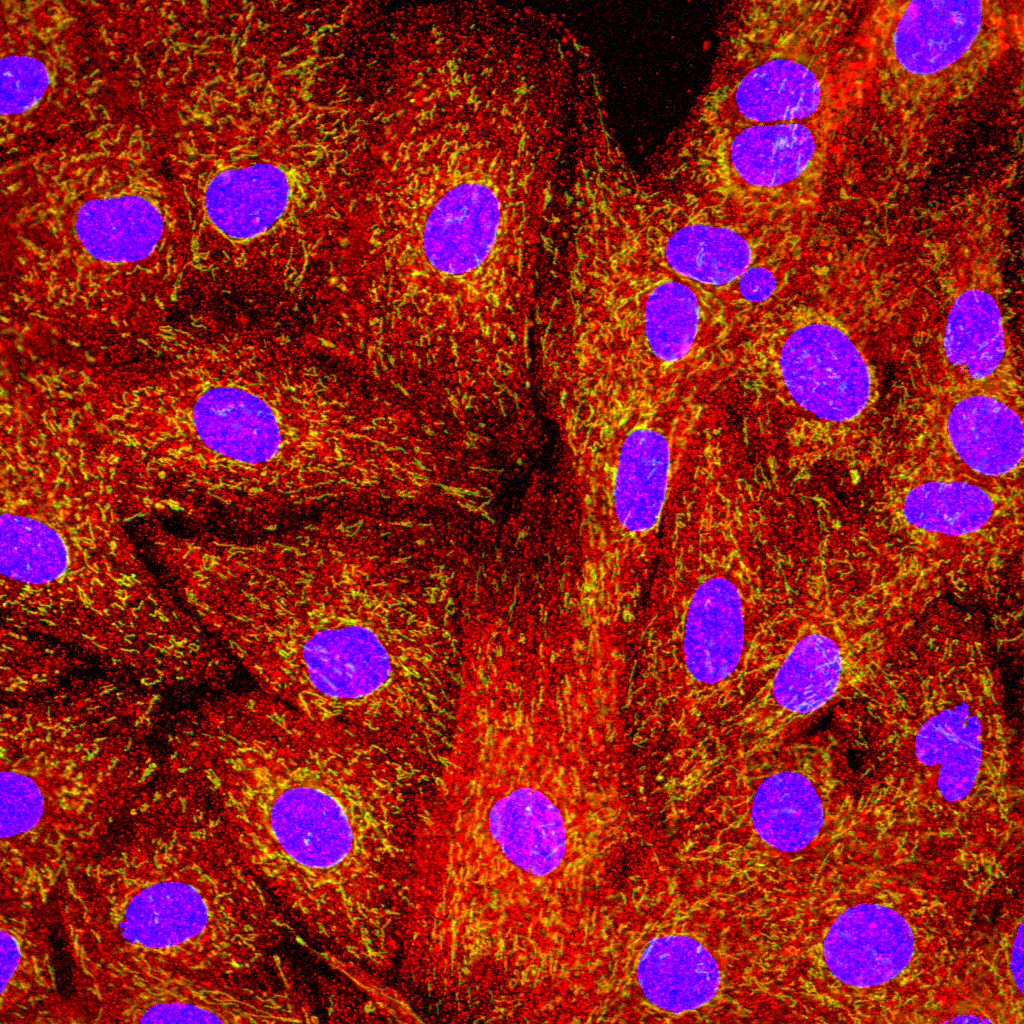

Supplement: Supplementary file 6 — Supplementary material [file mmc6.zip › GK Glucose Time Course - 2nd Experiment/E2-G5-1.tif]

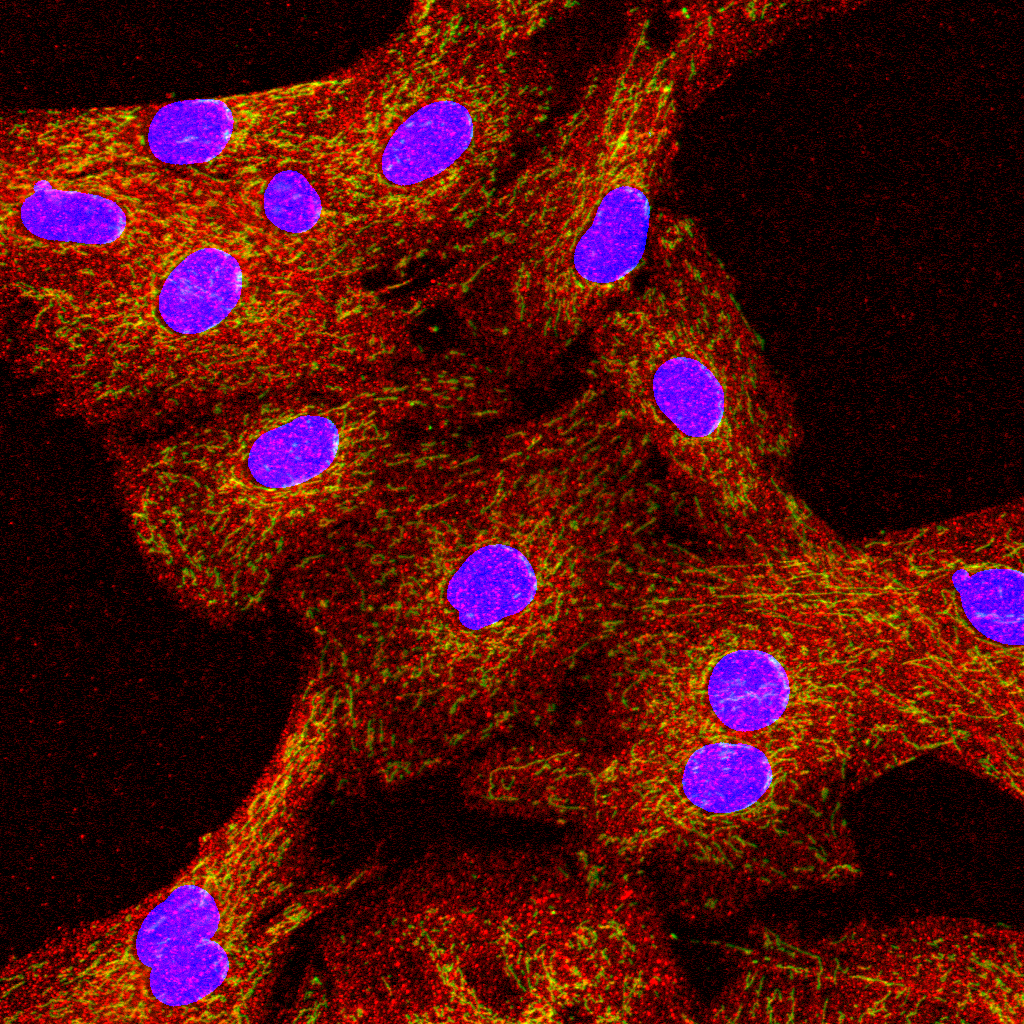

Supplement: Supplementary file 6 — Supplementary material [file mmc6.zip › GK Glucose Time Course - 2nd Experiment/E2-G5-2.tif]

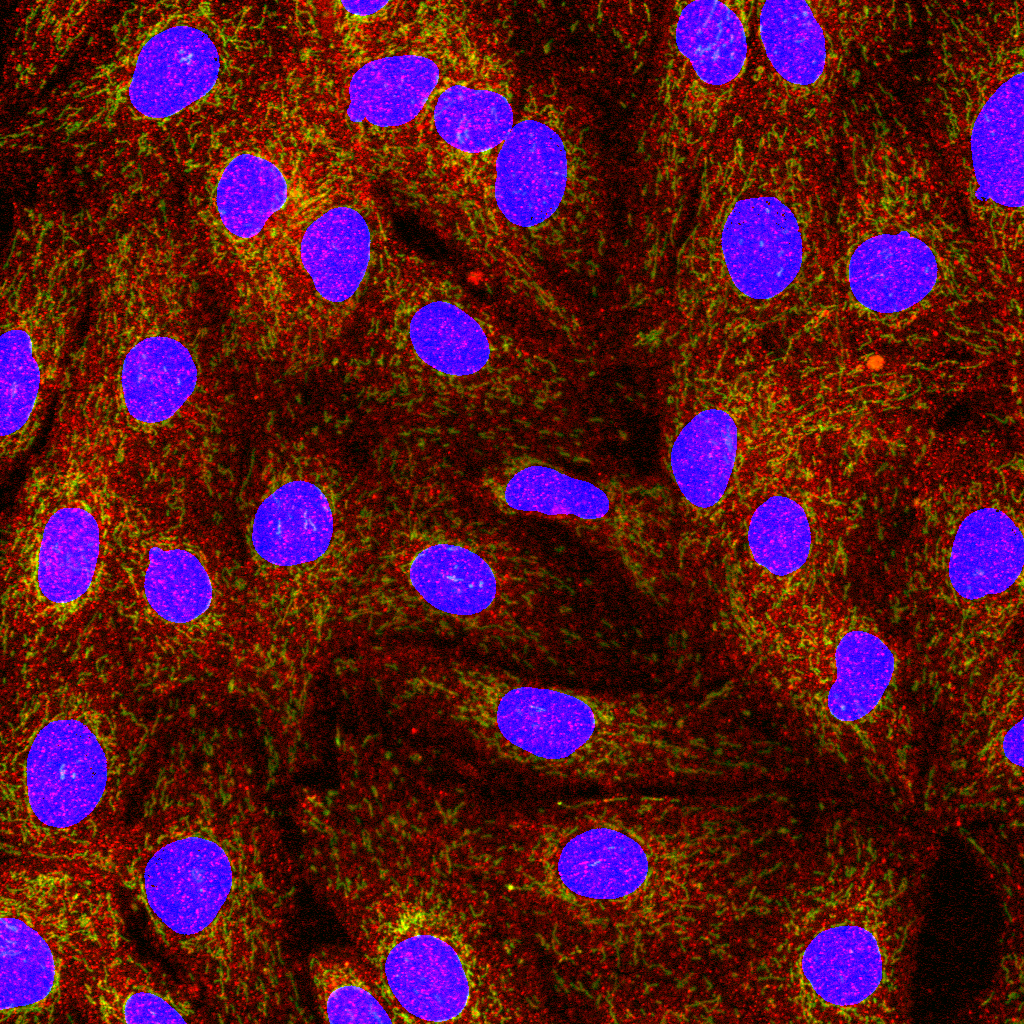

Supplement: Supplementary file 6 — Supplementary material [file mmc6.zip › GK Glucose Time Course - 2nd Experiment/E2-G5-3.tif]

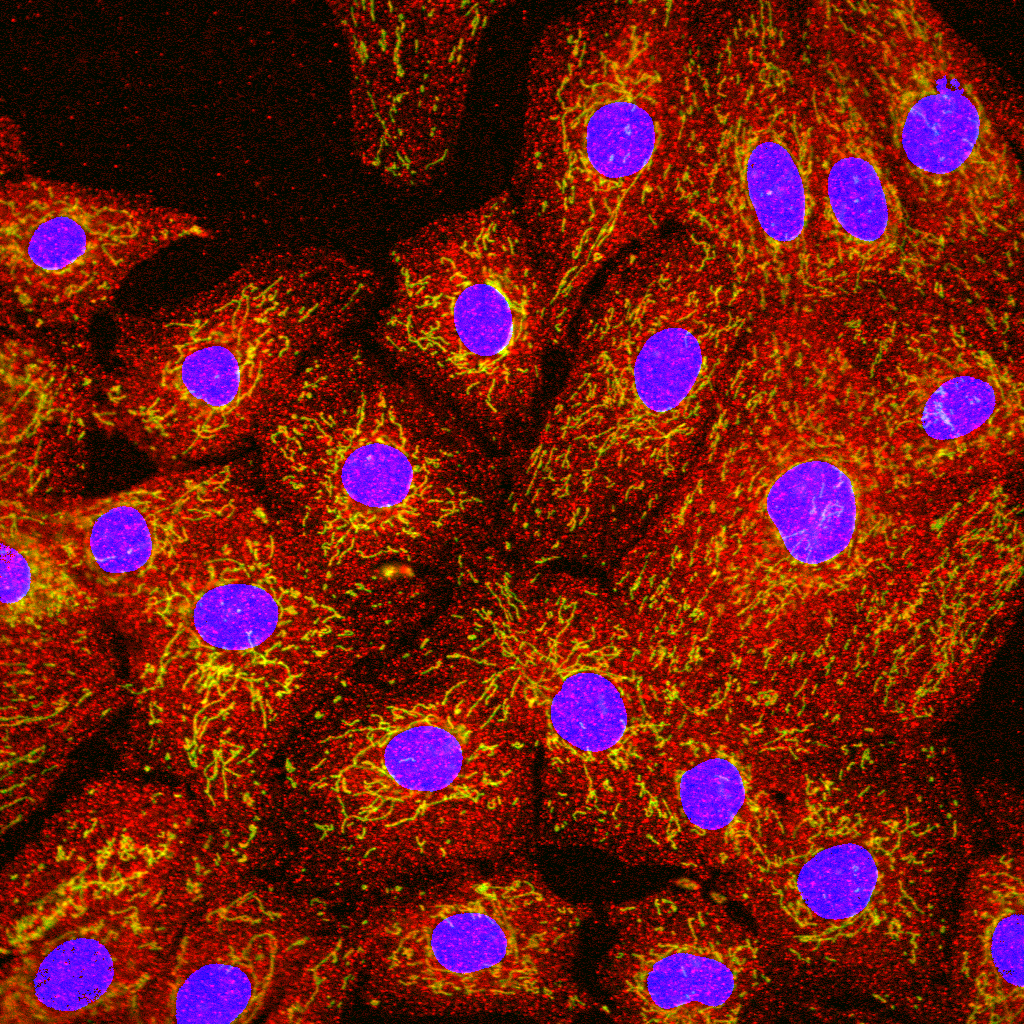

Supplement: Supplementary file 6 — Supplementary material [file mmc6.zip › GK Glucose Time Course - 2nd Experiment/E2-G5-4.tif]

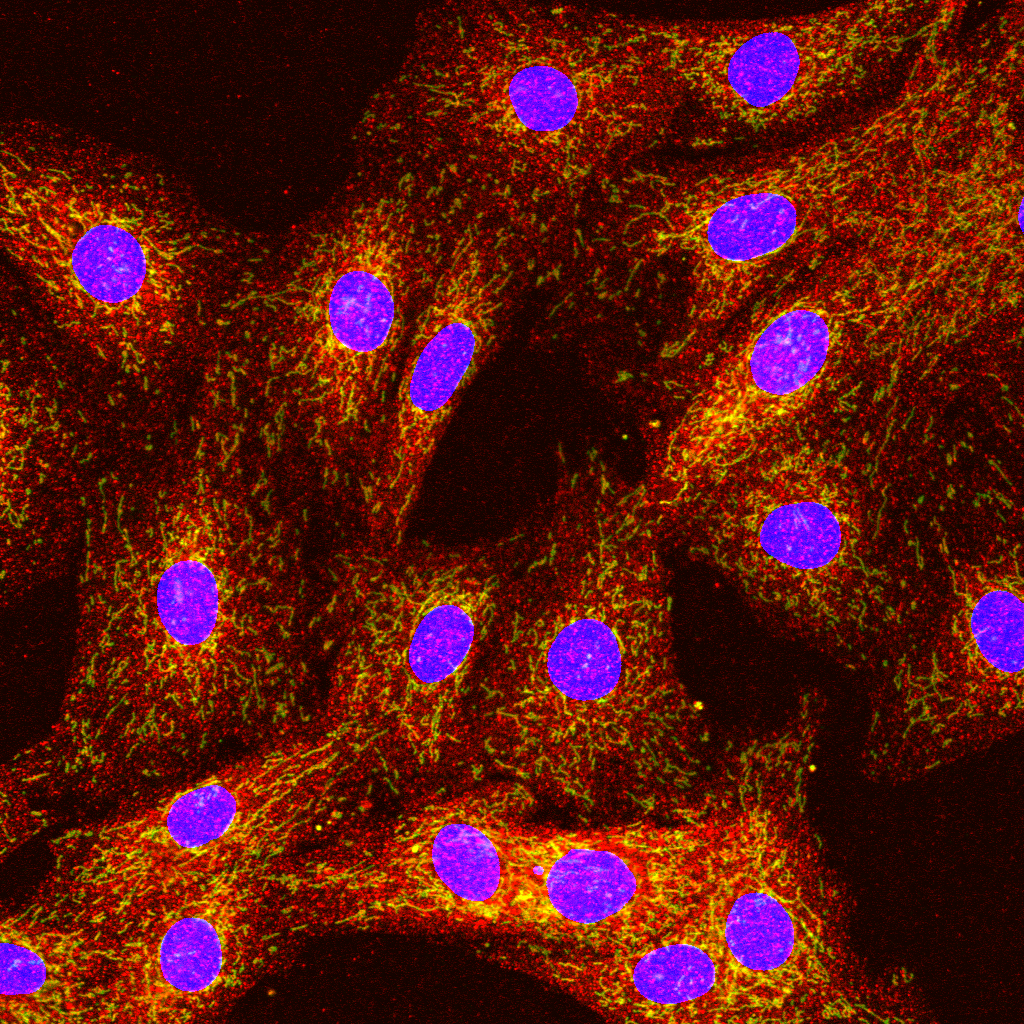

Supplement: Supplementary file 6 — Supplementary material [file mmc6.zip › GK Glucose Time Course - 2nd Experiment/E2-G5-5.tif]

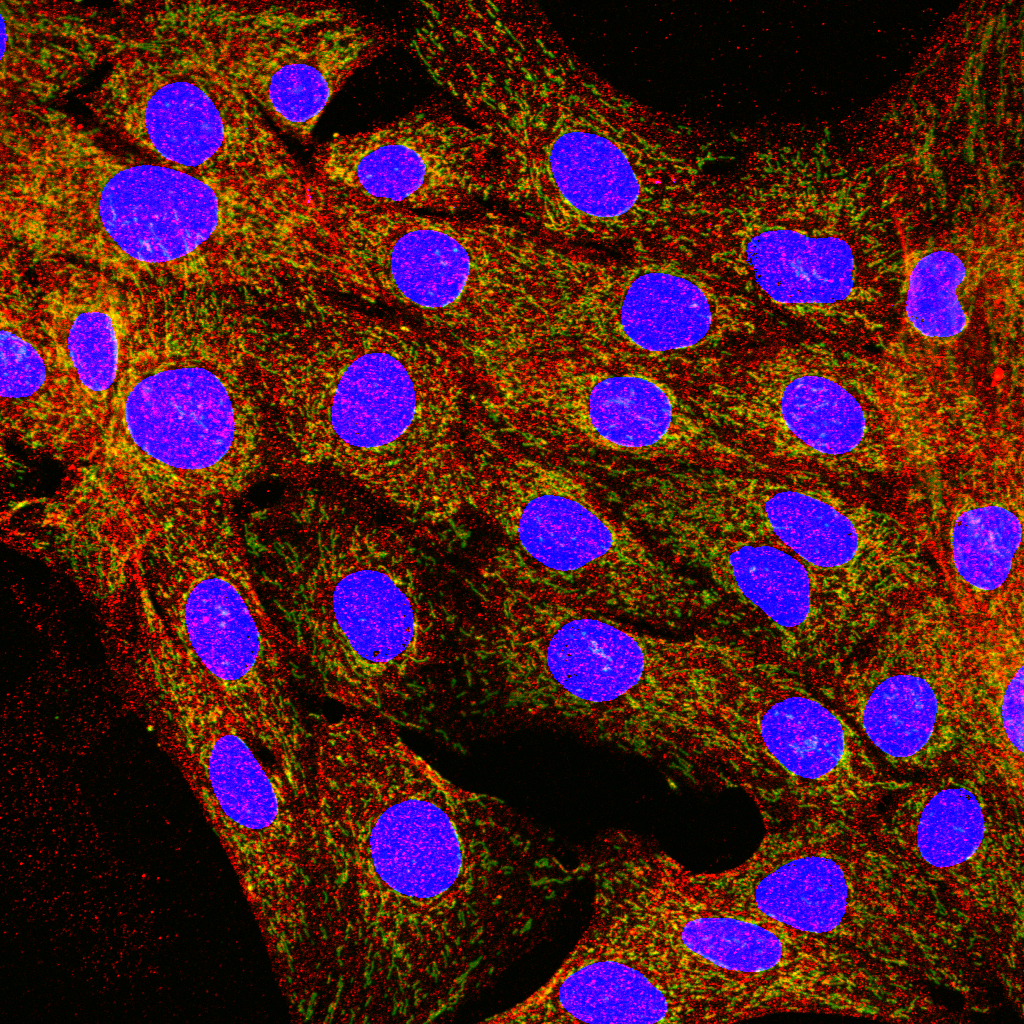

Supplement: Supplementary file 6 — Supplementary material [file mmc6.zip › GK Glucose Time Course - 2nd Experiment/E2-G5-6.tif]

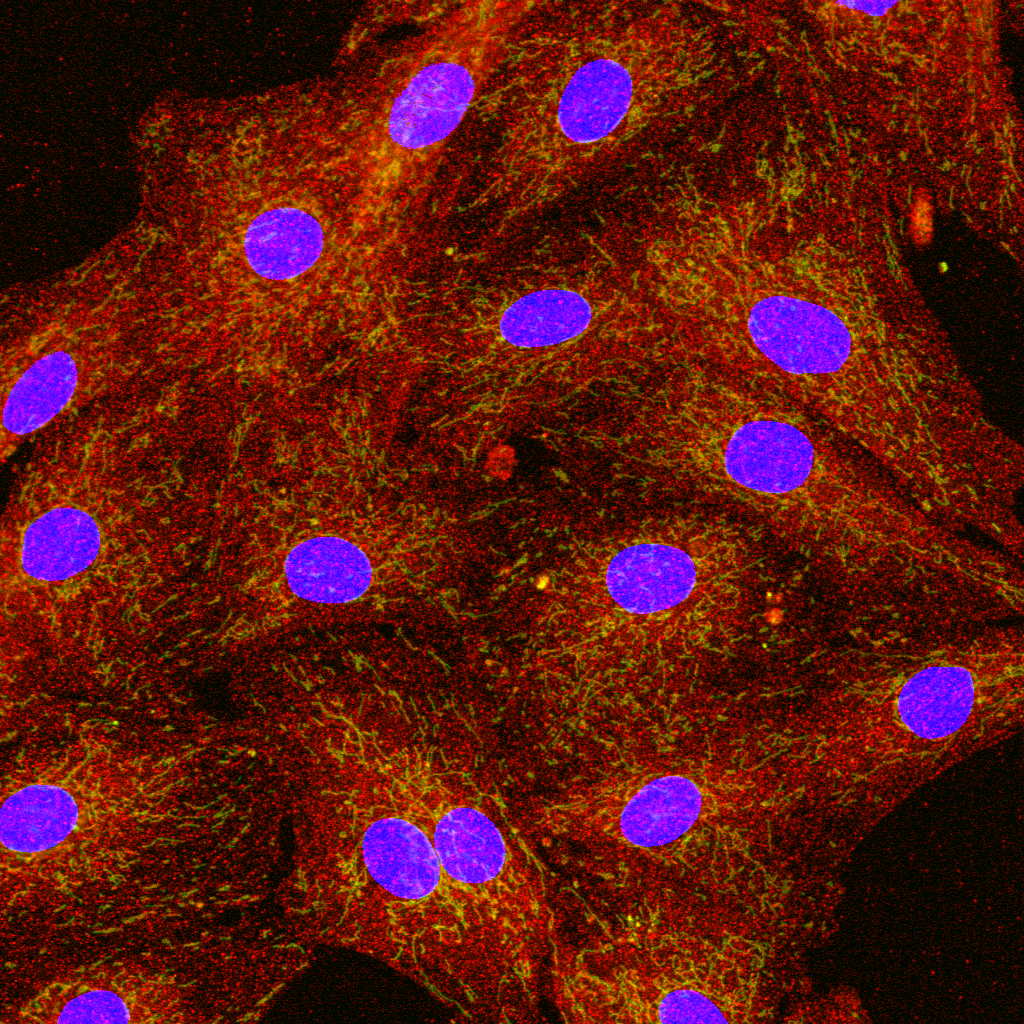

Supplement: Supplementary file 6 — Supplementary material [file mmc6.zip › GK Glucose Time Course - 2nd Experiment/E2-G5-7.tif]

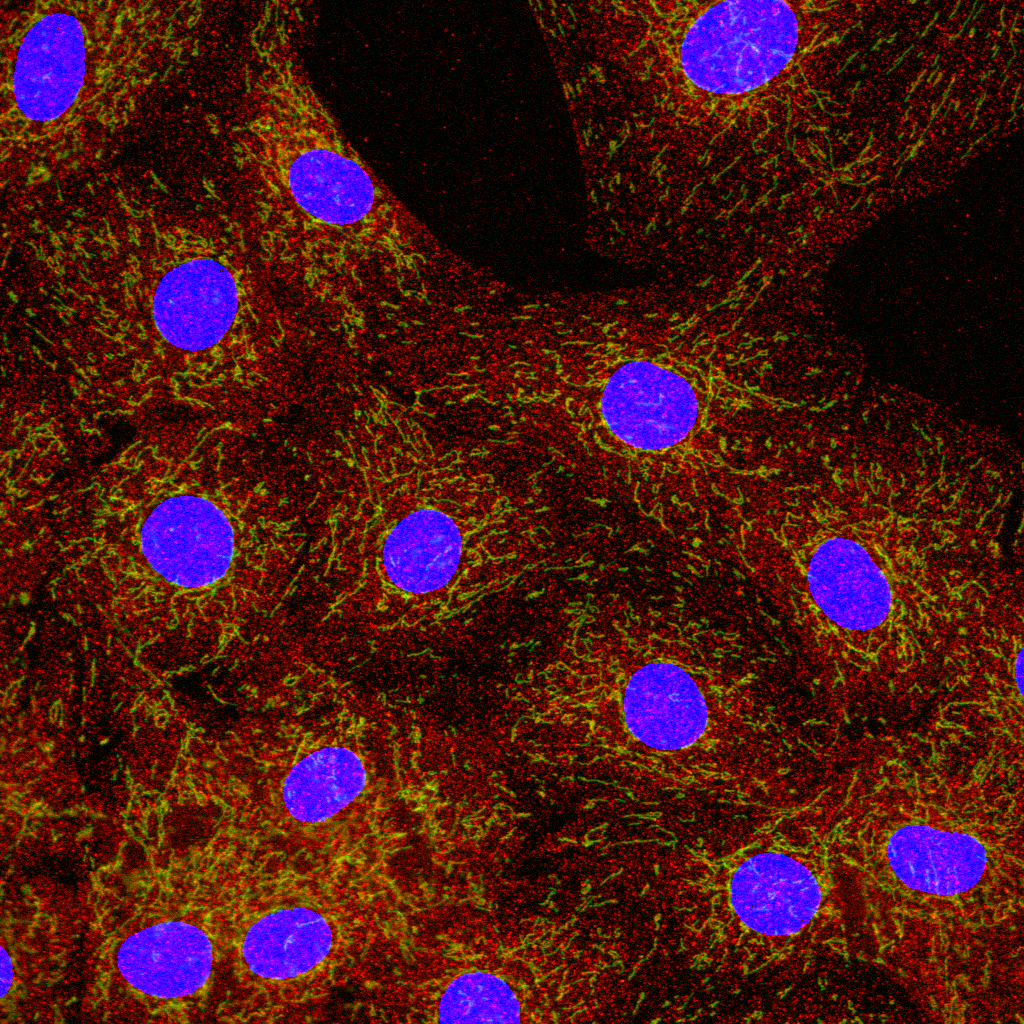

Supplement: Supplementary file 6 — Supplementary material [file mmc6.zip › GK Glucose Time Course - 2nd Experiment/E2-G5-8.tif]

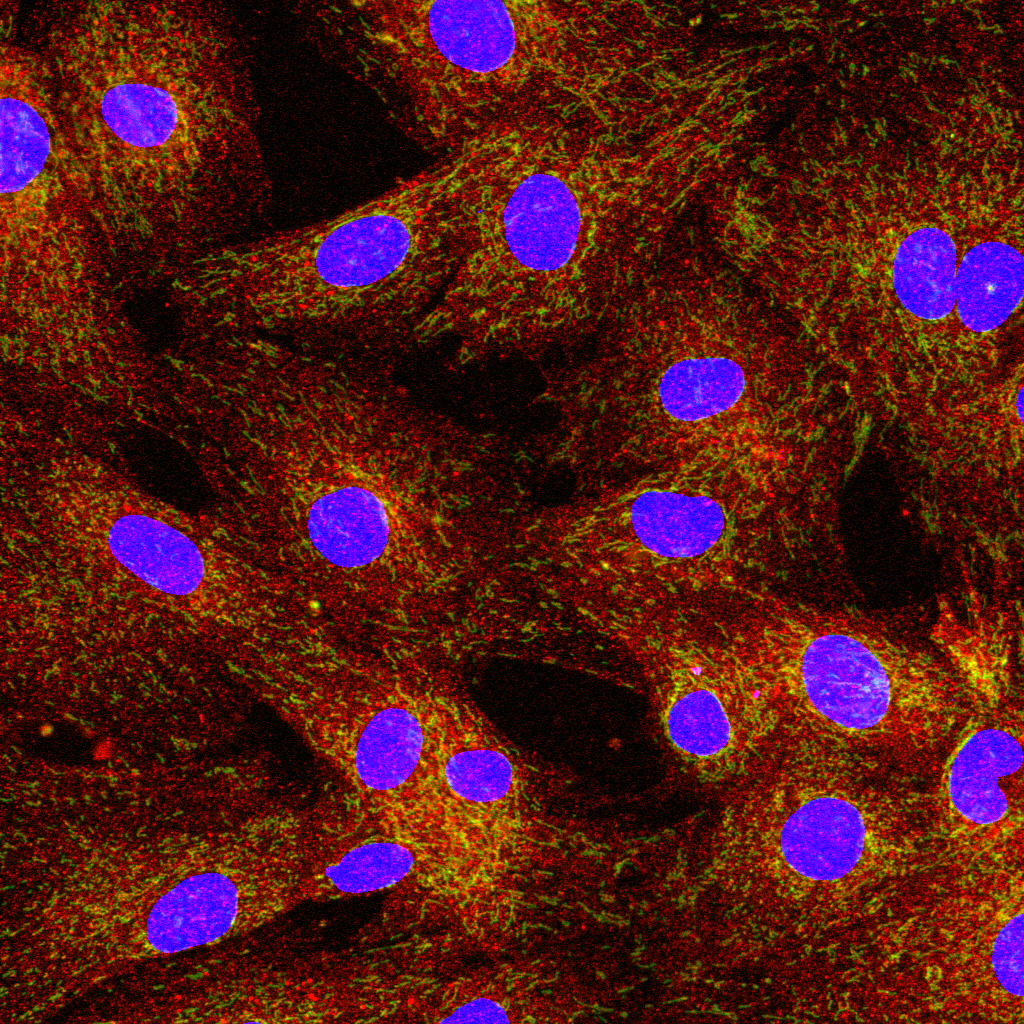

Supplement: Supplementary file 6 — Supplementary material [file mmc6.zip › GK Glucose Time Course - 2nd Experiment/E2-G5-9.tif]

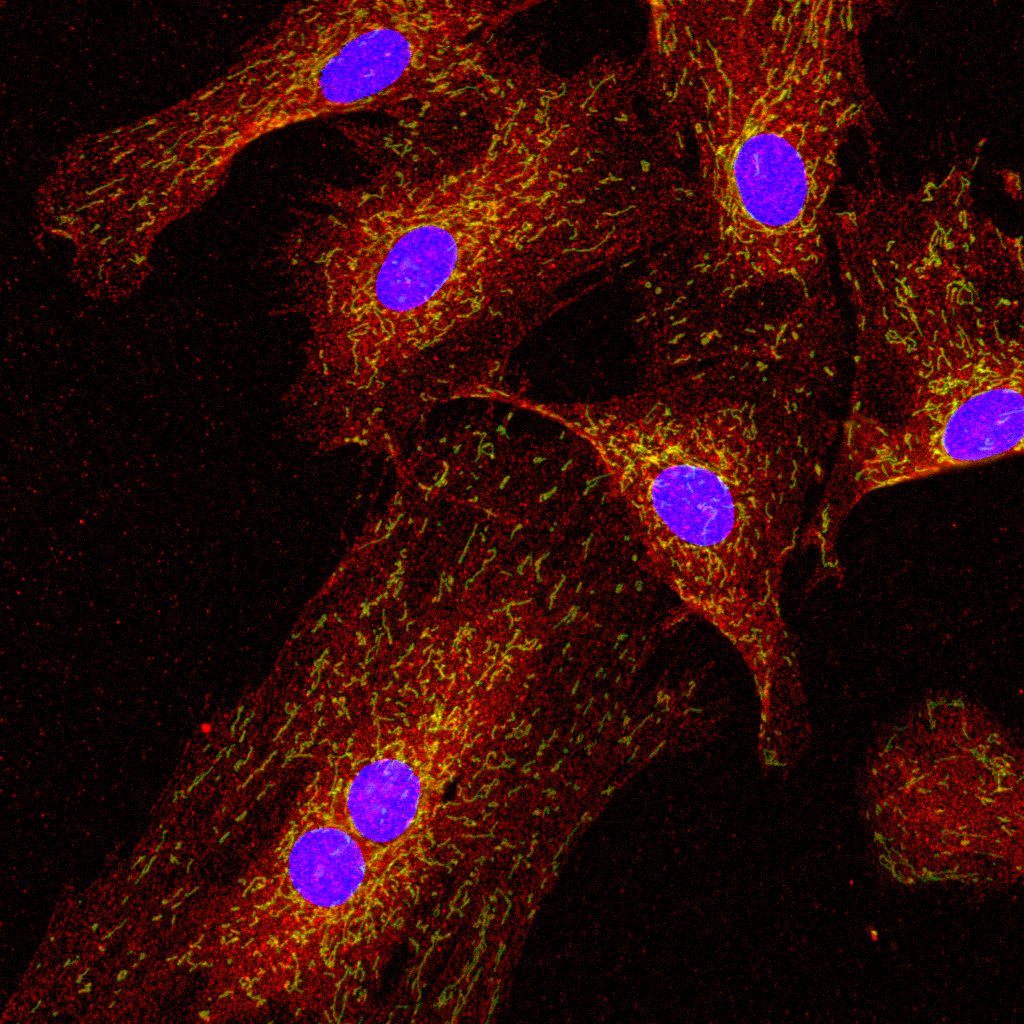

Supplement: Supplementary file 7 — Supplementary material [file mmc7.zip › GK Glucose Time Course - 3rd Experiment/E3-G2-1.tif]

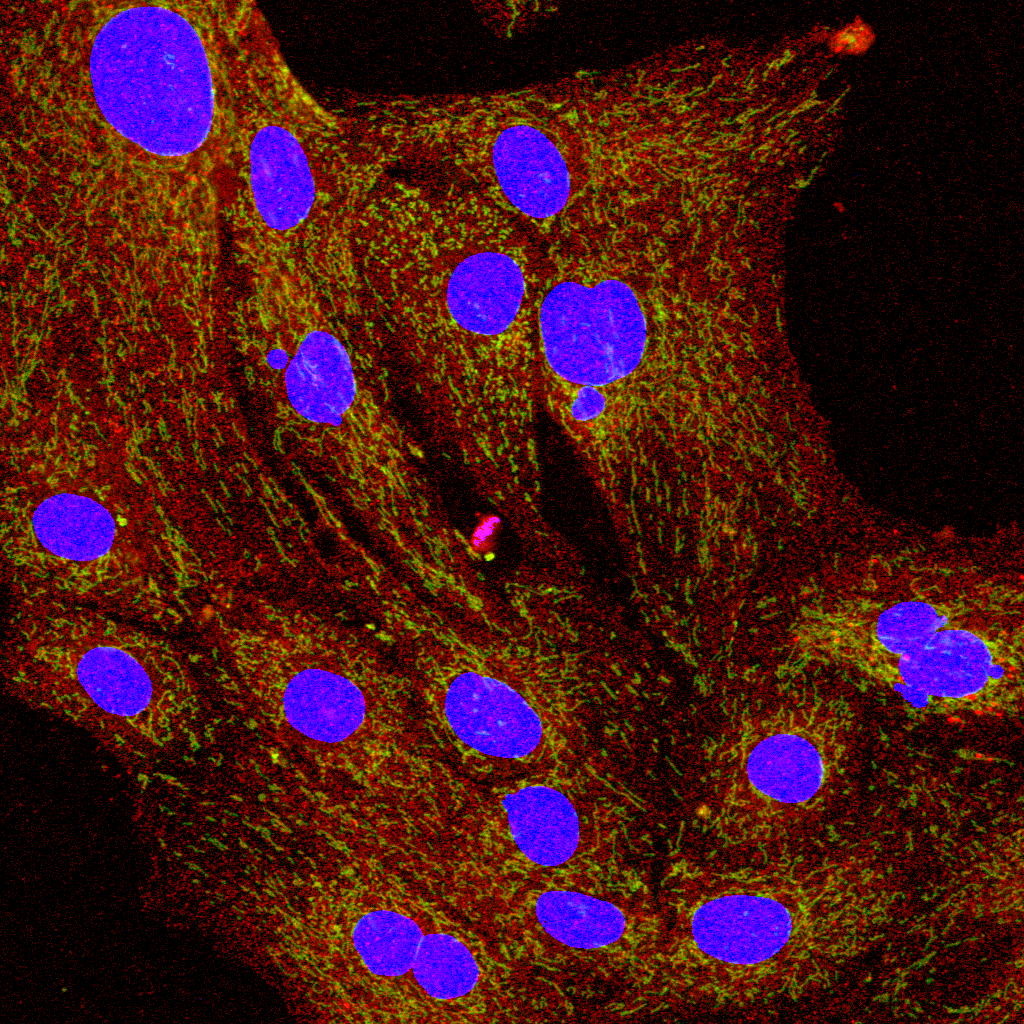

Supplement: Supplementary file 7 — Supplementary material [file mmc7.zip › GK Glucose Time Course - 3rd Experiment/E3-G2-2.tif]

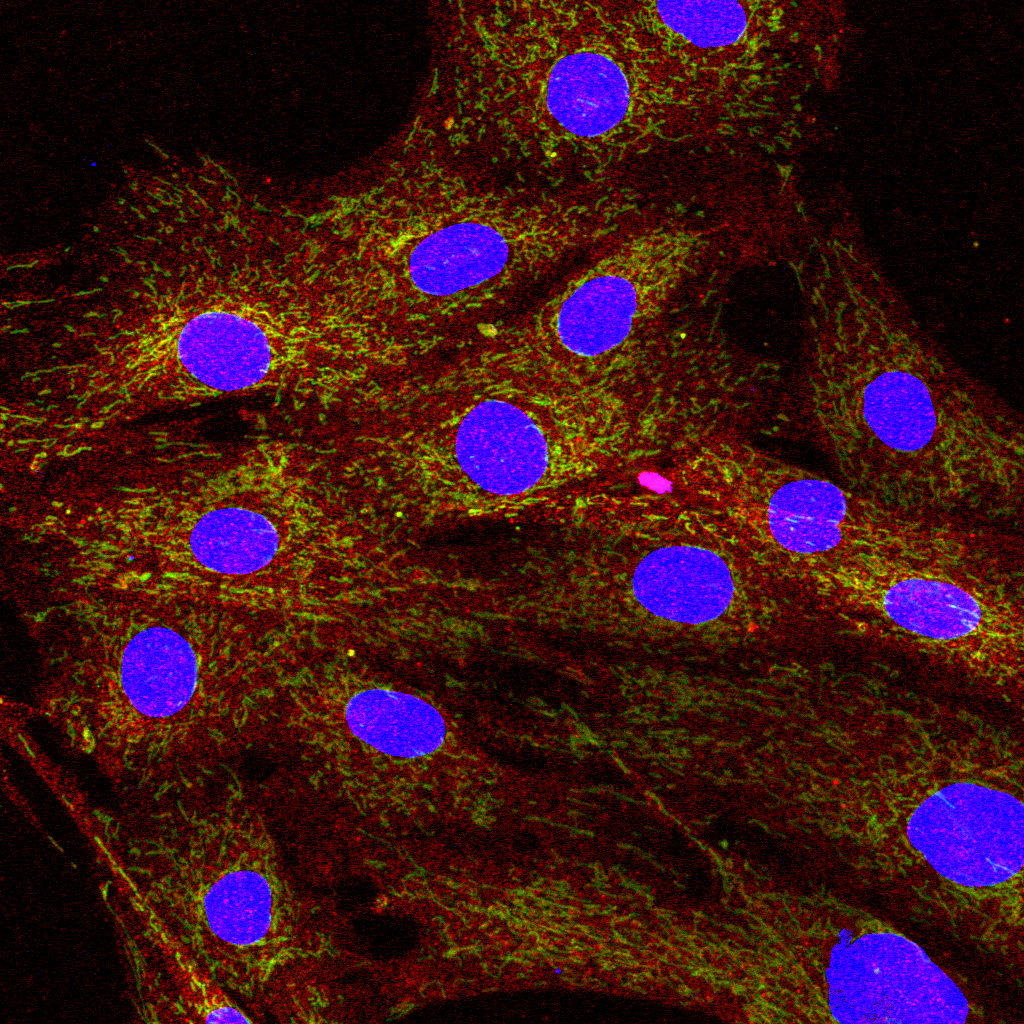

Supplement: Supplementary file 7 — Supplementary material [file mmc7.zip › GK Glucose Time Course - 3rd Experiment/E3-G2-3.tif]

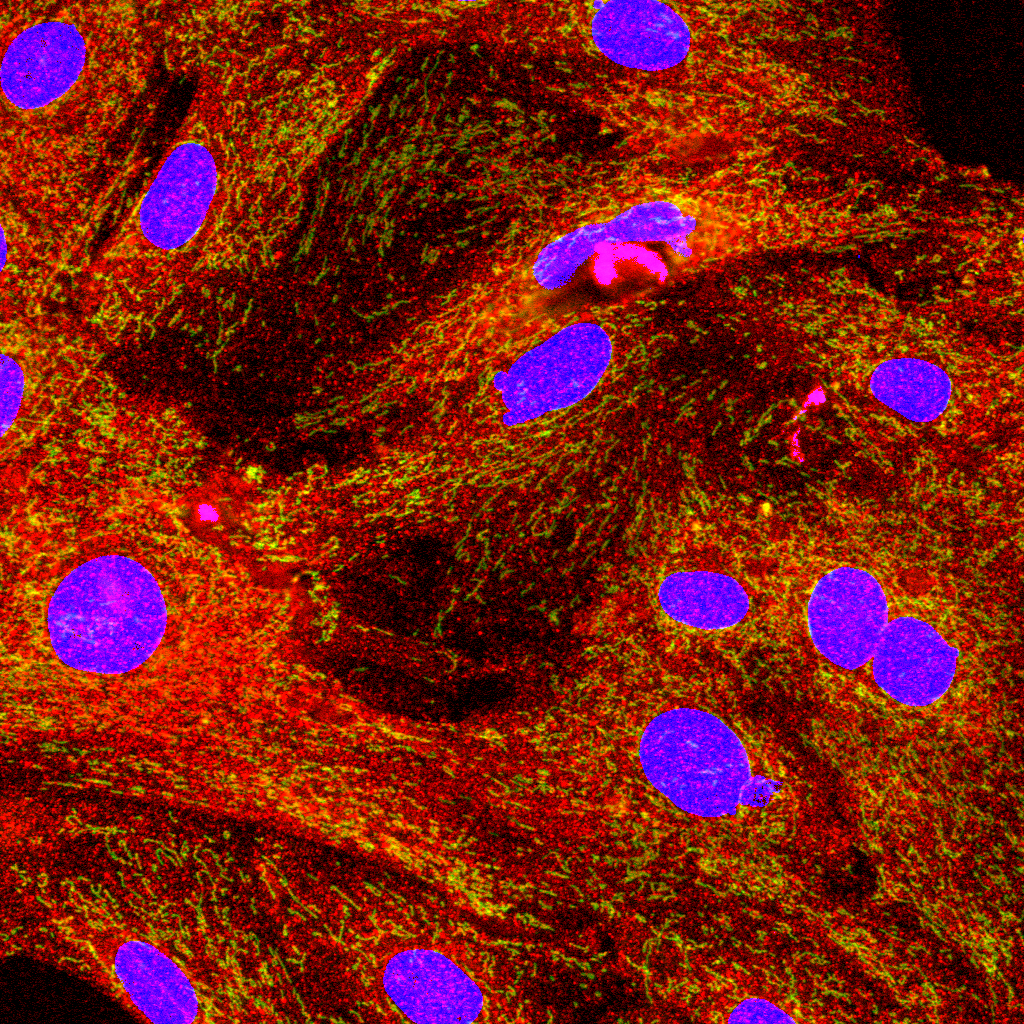

Supplement: Supplementary file 7 — Supplementary material [file mmc7.zip › GK Glucose Time Course - 3rd Experiment/E3-G2-4.tif]

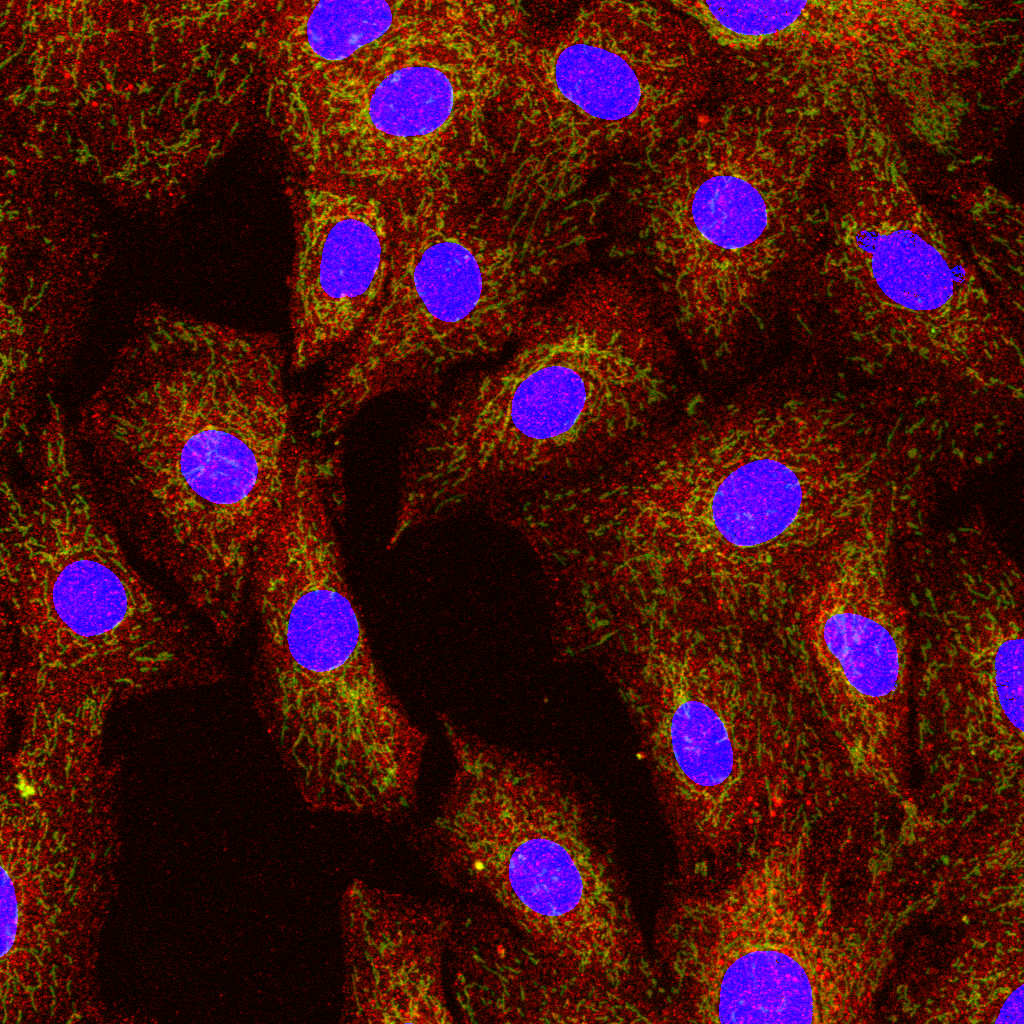

Supplement: Supplementary file 7 — Supplementary material [file mmc7.zip › GK Glucose Time Course - 3rd Experiment/E3-G2-5.tif]

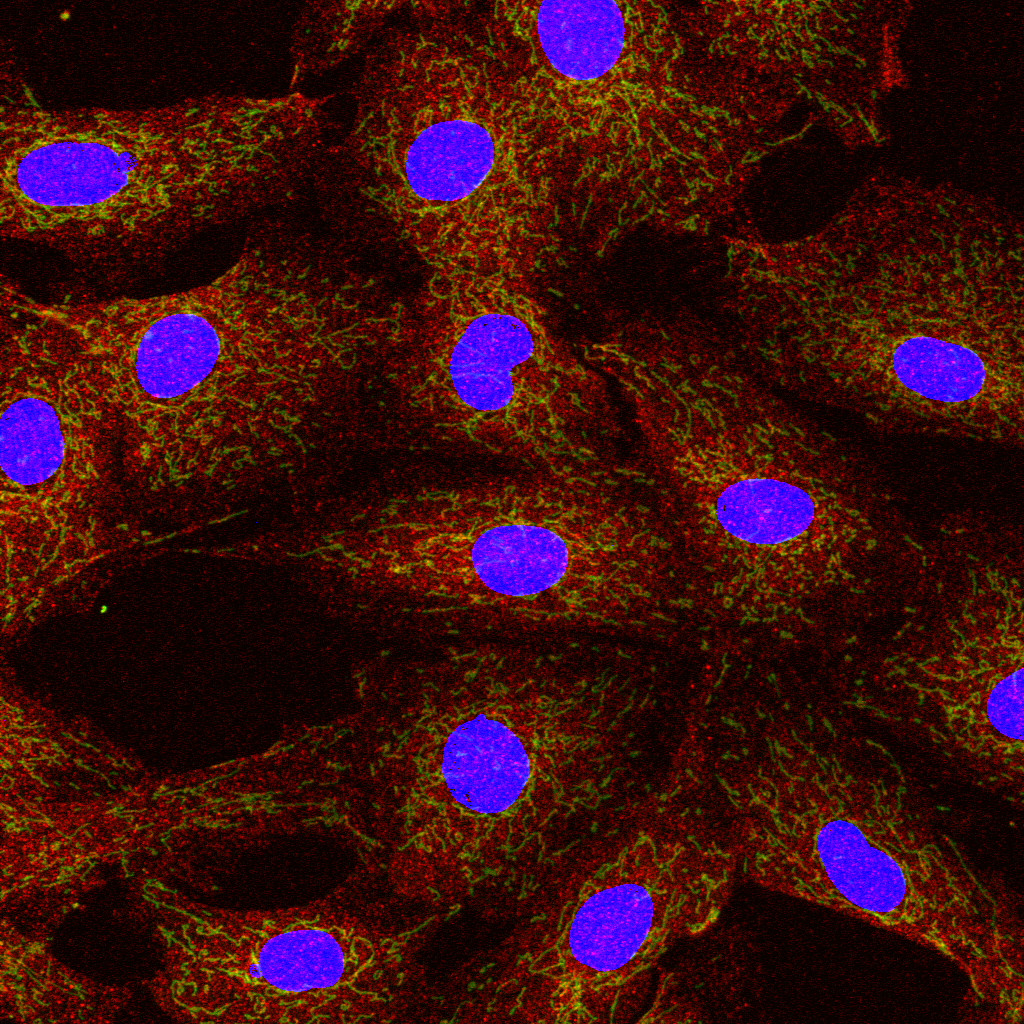

Supplement: Supplementary file 7 — Supplementary material [file mmc7.zip › GK Glucose Time Course - 3rd Experiment/E3-G2-6.tif]

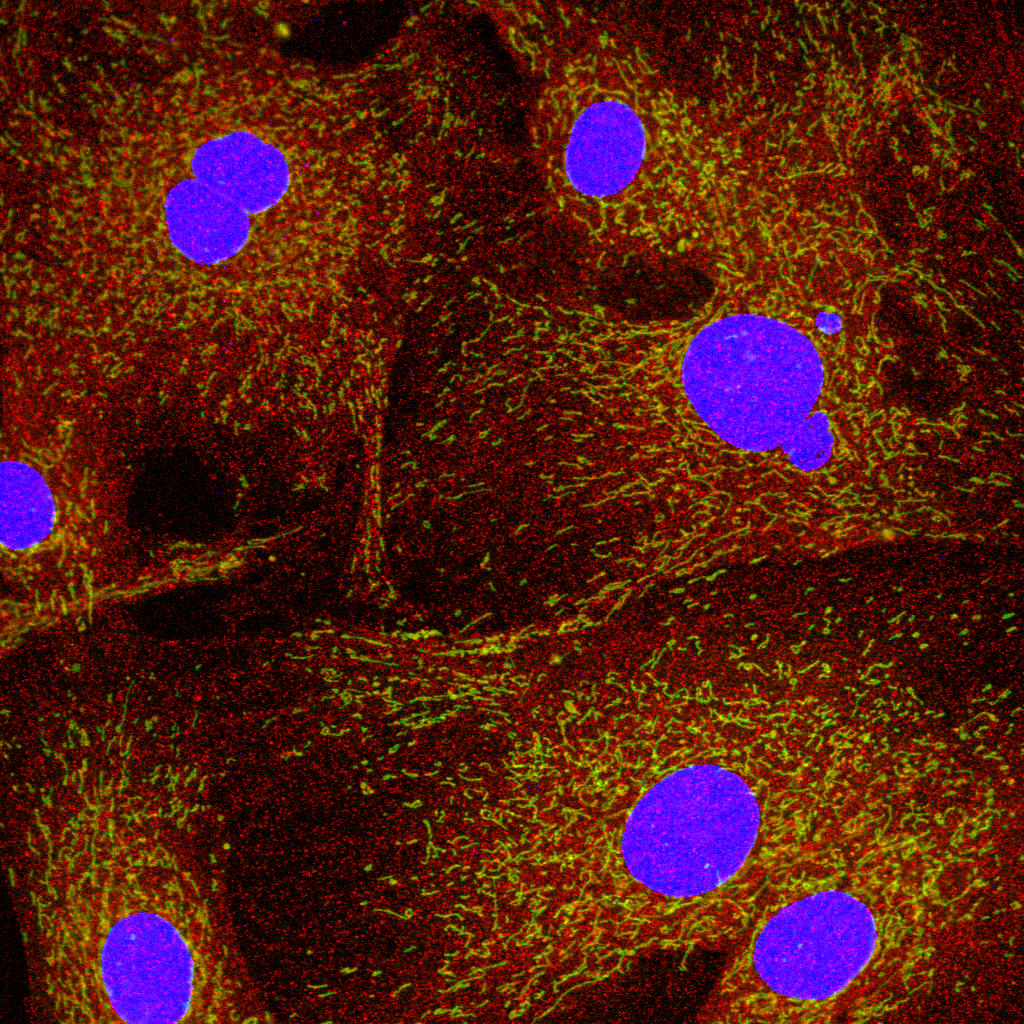

Supplement: Supplementary file 7 — Supplementary material [file mmc7.zip › GK Glucose Time Course - 3rd Experiment/E3-G2-7.tif]

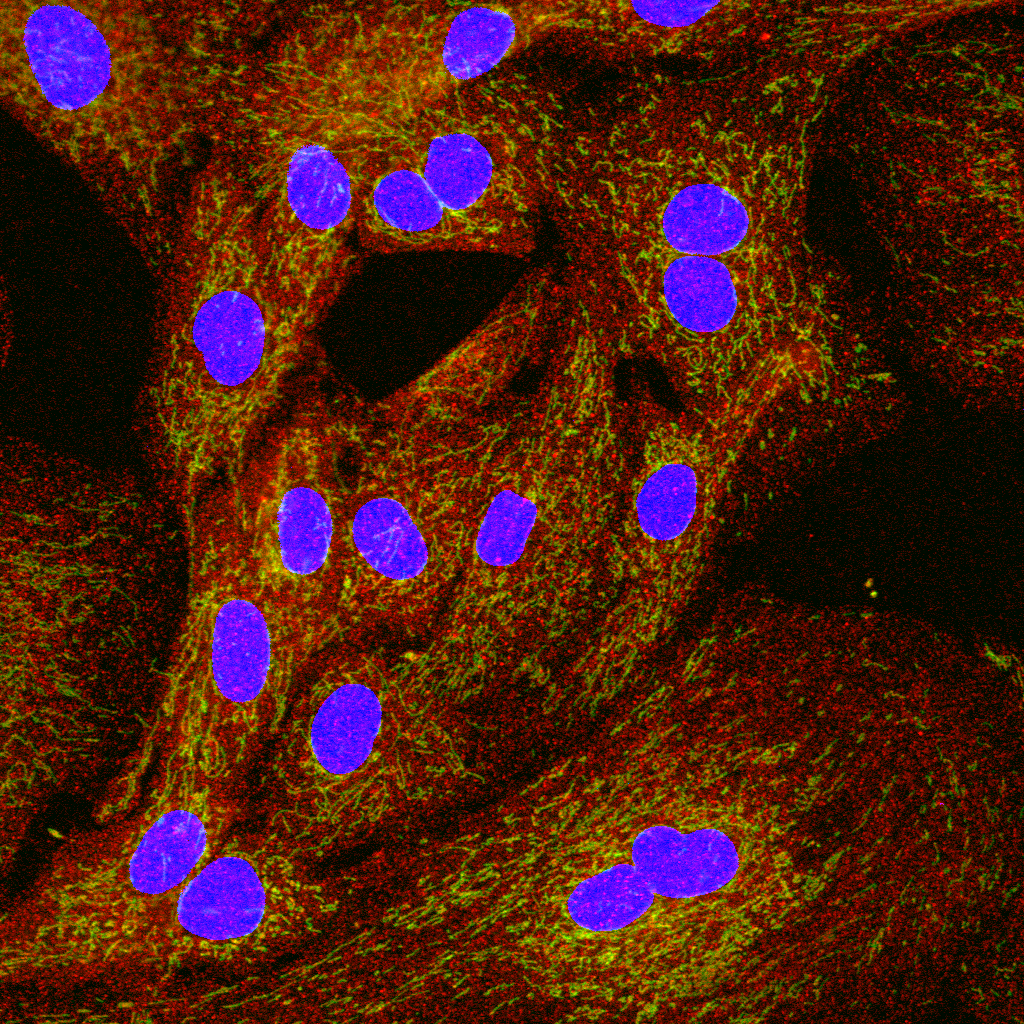

Supplement: Supplementary file 7 — Supplementary material [file mmc7.zip › GK Glucose Time Course - 3rd Experiment/E3-G2-8.tif]

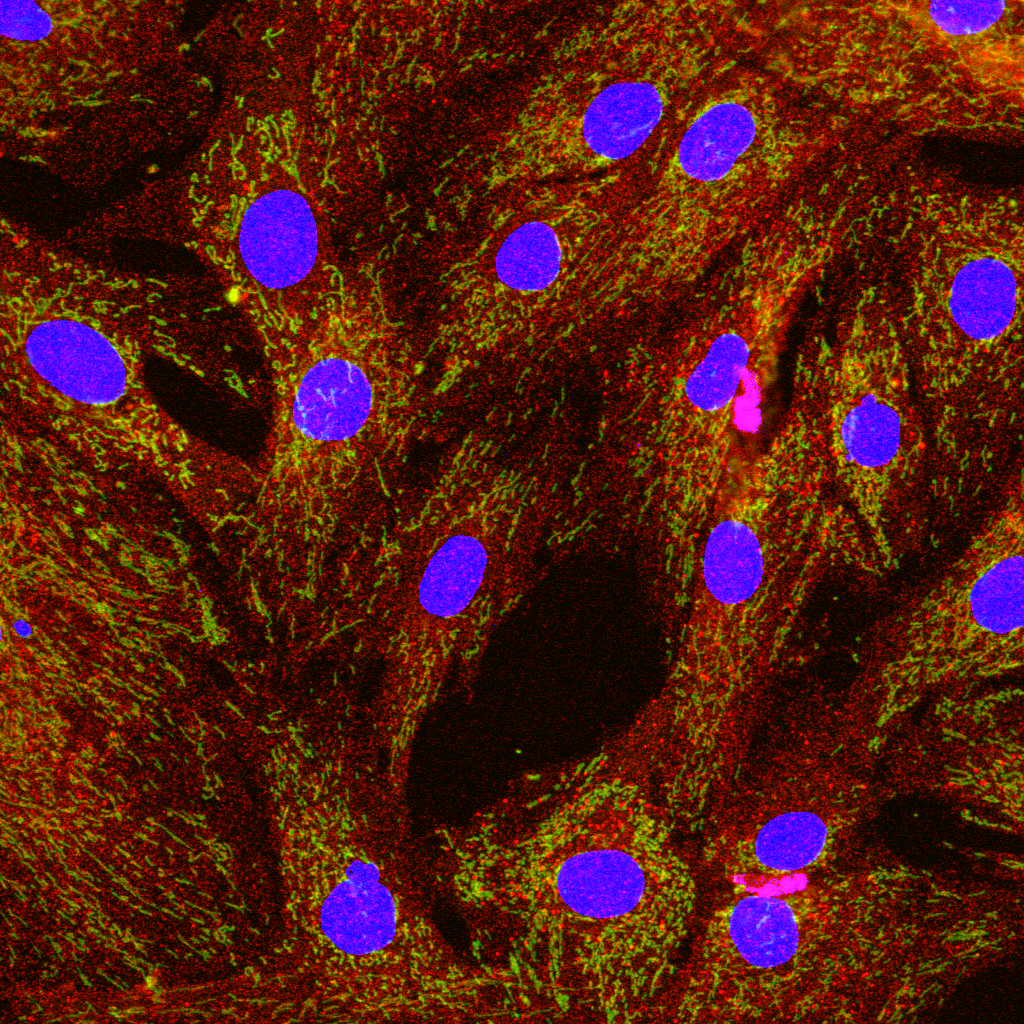

Supplement: Supplementary file 7 — Supplementary material [file mmc7.zip › GK Glucose Time Course - 3rd Experiment/E3-G2-9.tif]

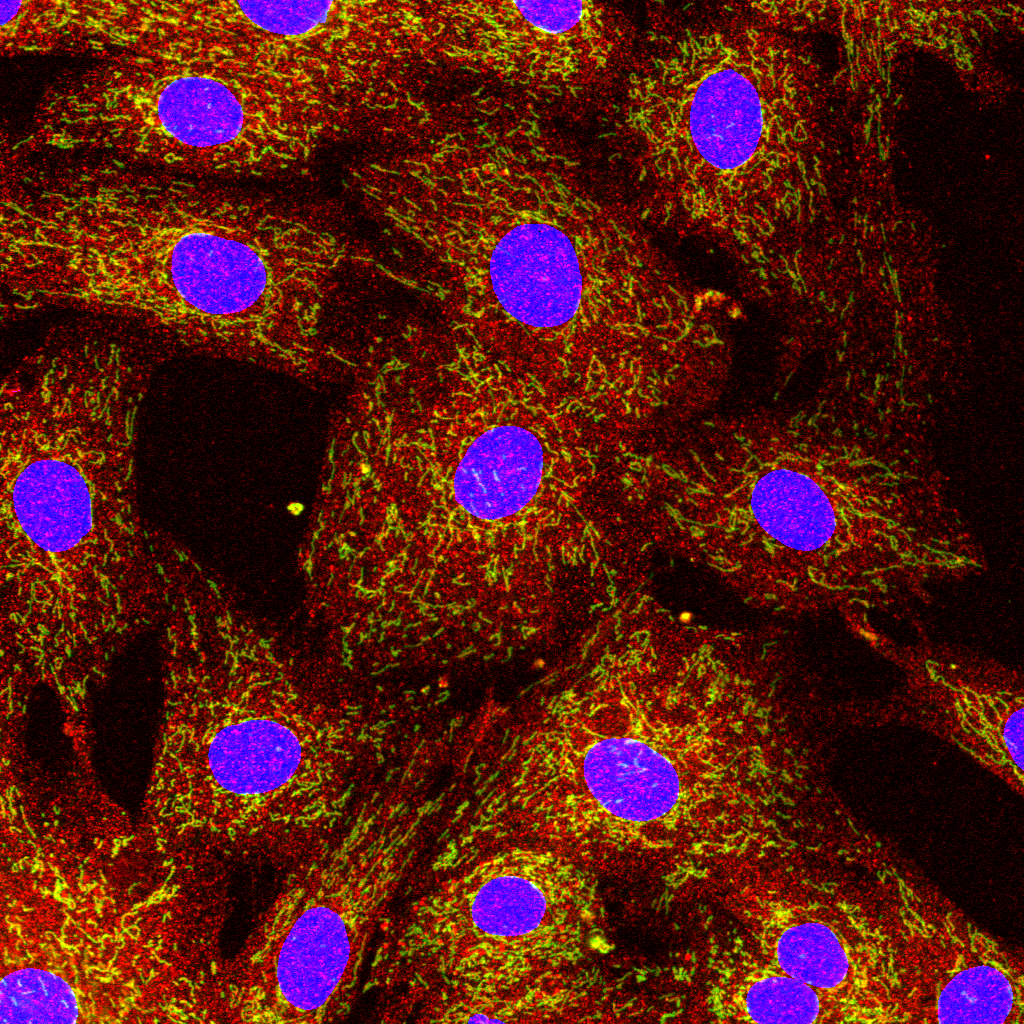

Supplement: Supplementary file 7 — Supplementary material [file mmc7.zip › GK Glucose Time Course - 3rd Experiment/E3-G3-1.tif]

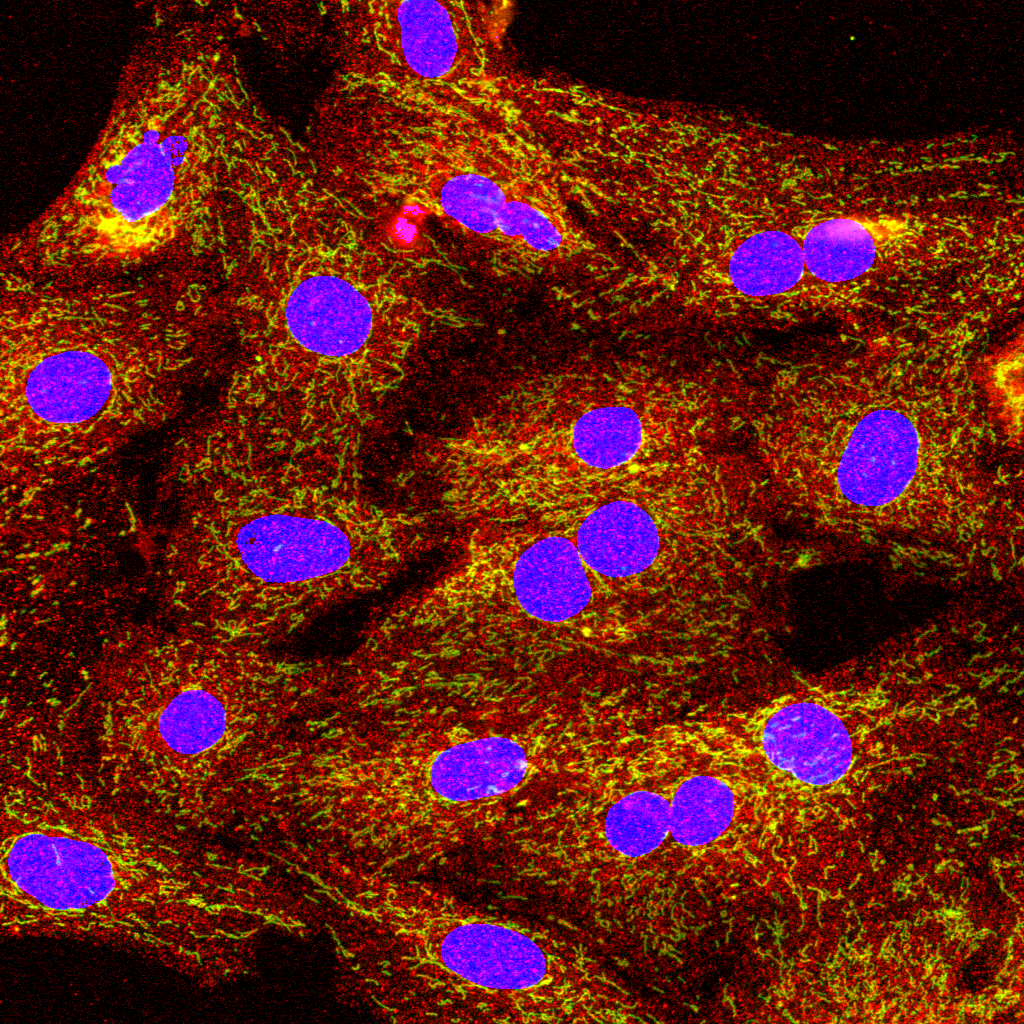

Supplement: Supplementary file 7 — Supplementary material [file mmc7.zip › GK Glucose Time Course - 3rd Experiment/E3-G3-2.tif]

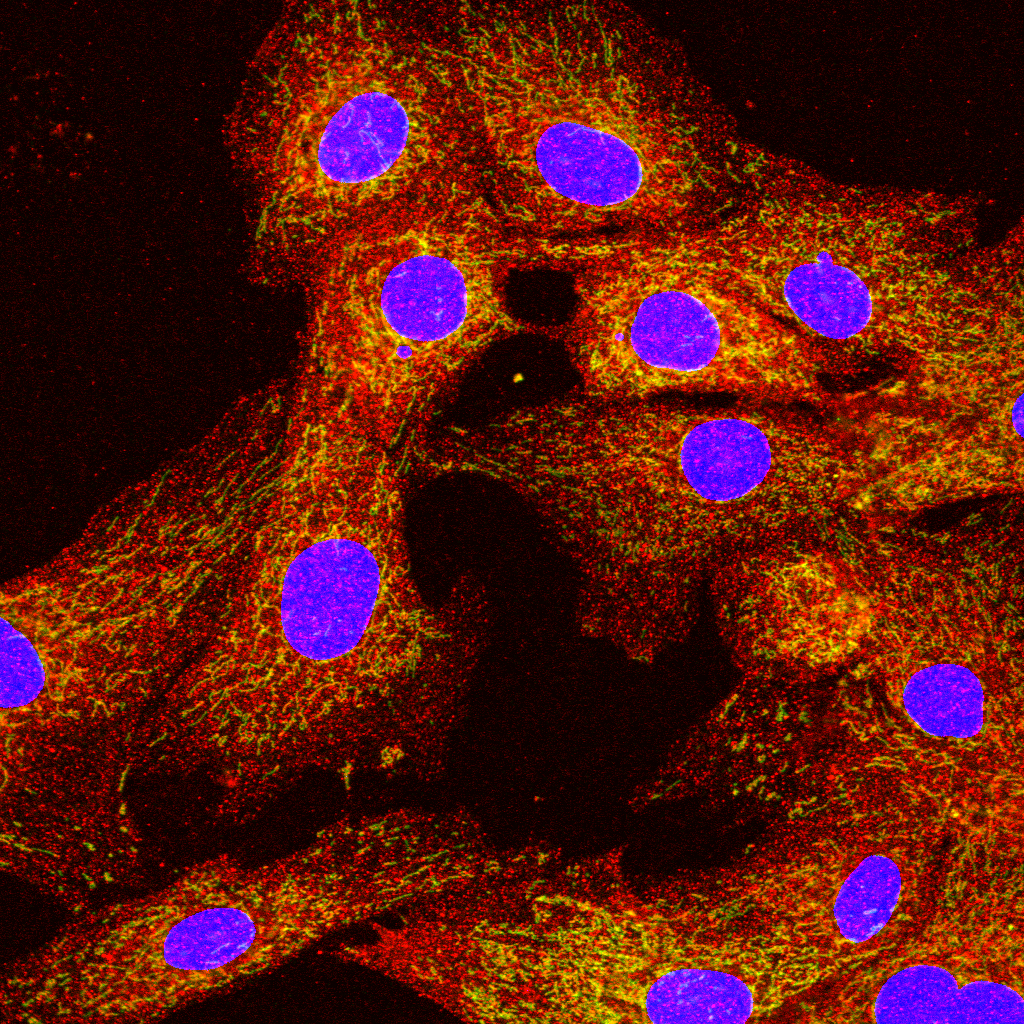

Supplement: Supplementary file 7 — Supplementary material [file mmc7.zip › GK Glucose Time Course - 3rd Experiment/E3-G3-3.tif]

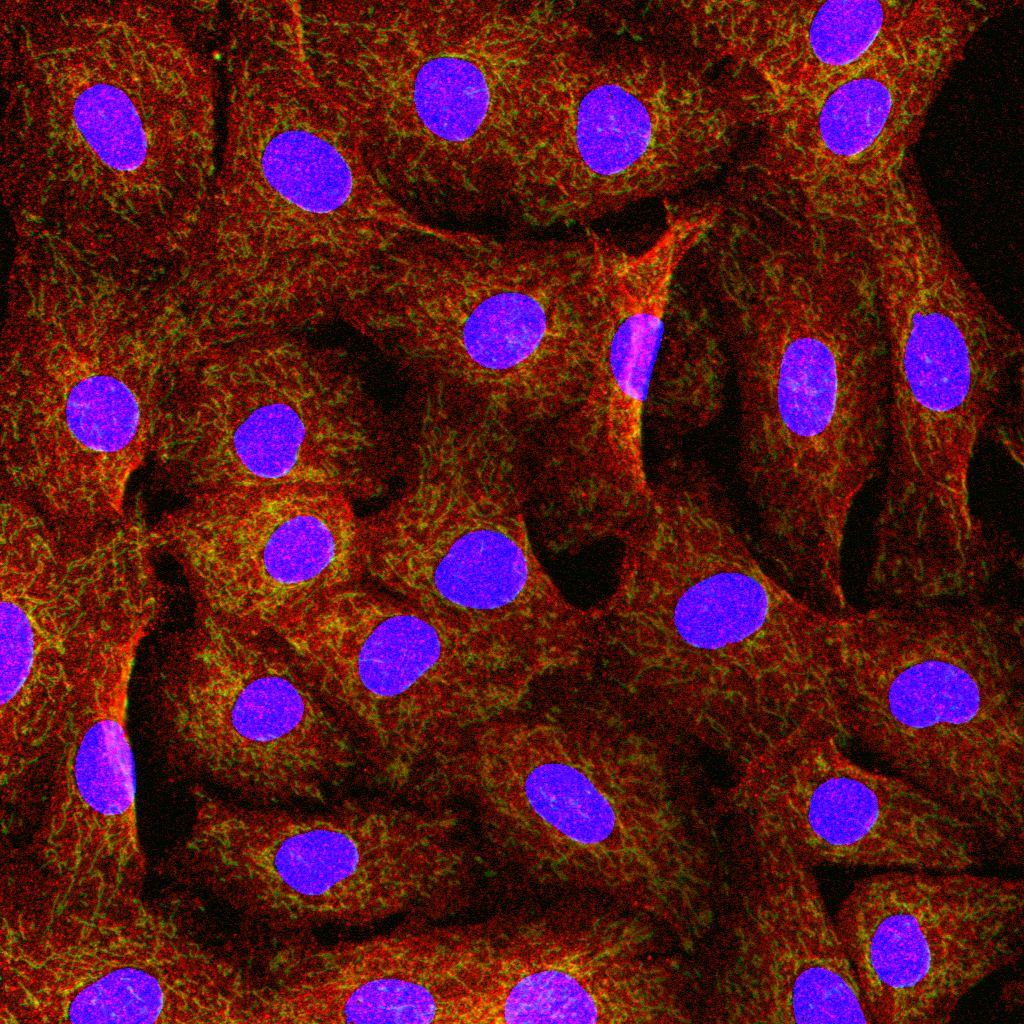

Supplement: Supplementary file 7 — Supplementary material [file mmc7.zip › GK Glucose Time Course - 3rd Experiment/E3-G3-4.tif]

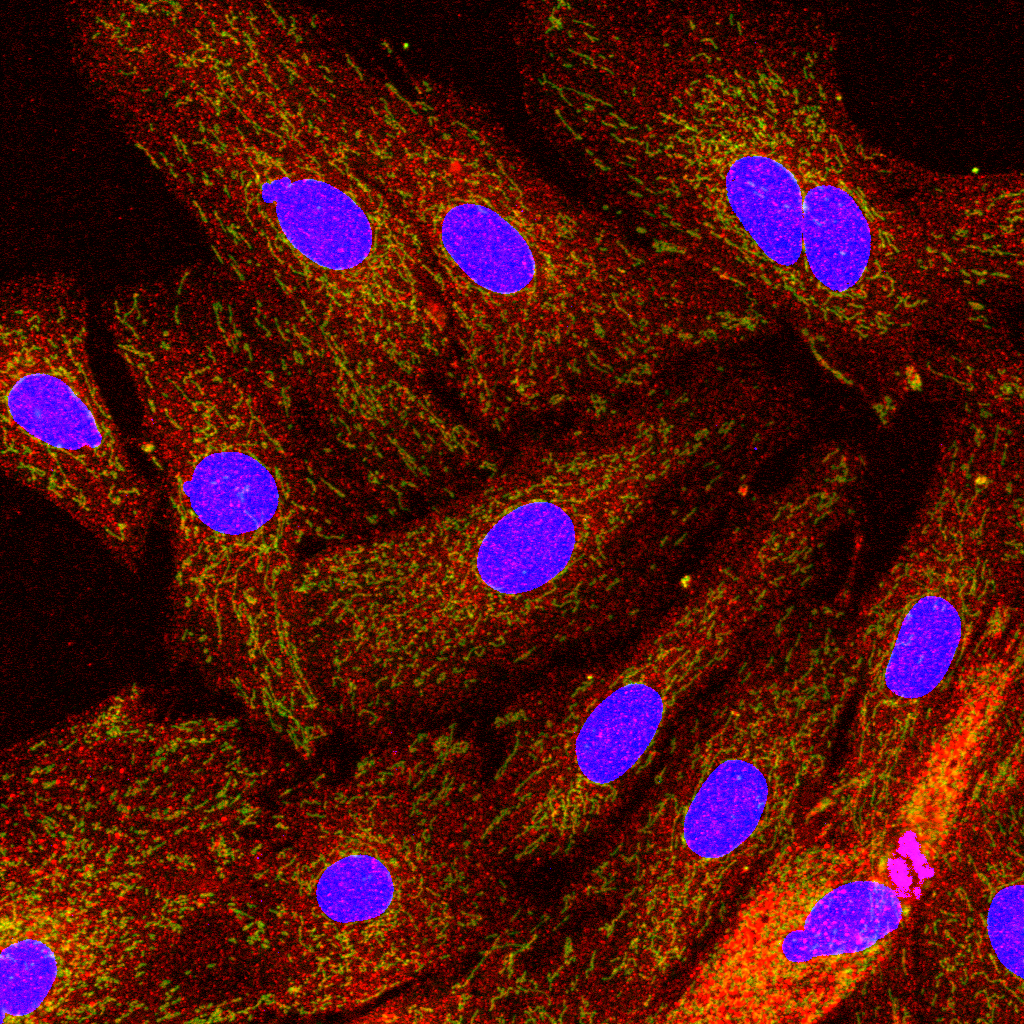

Supplement: Supplementary file 7 — Supplementary material [file mmc7.zip › GK Glucose Time Course - 3rd Experiment/E3-G3-5.tif]

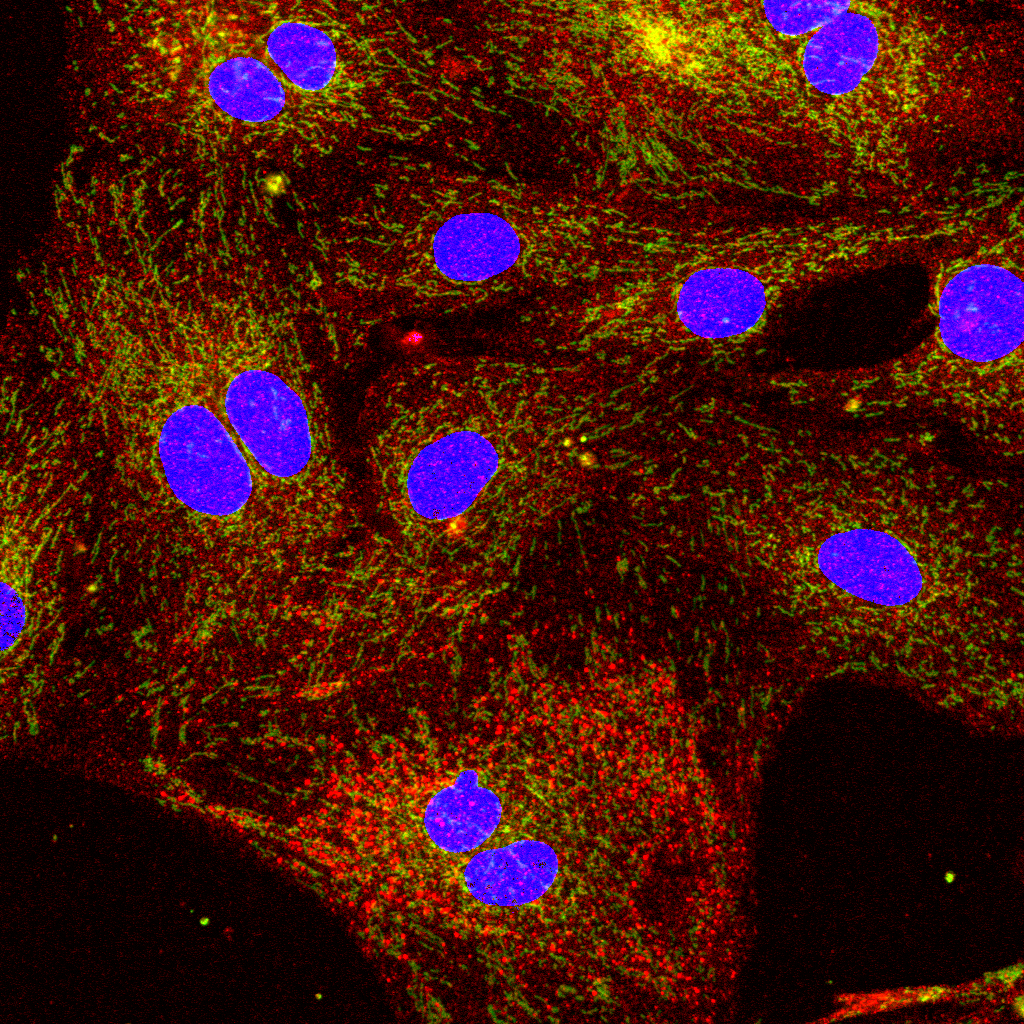

Supplement: Supplementary file 7 — Supplementary material [file mmc7.zip › GK Glucose Time Course - 3rd Experiment/E3-G3-6.tif]

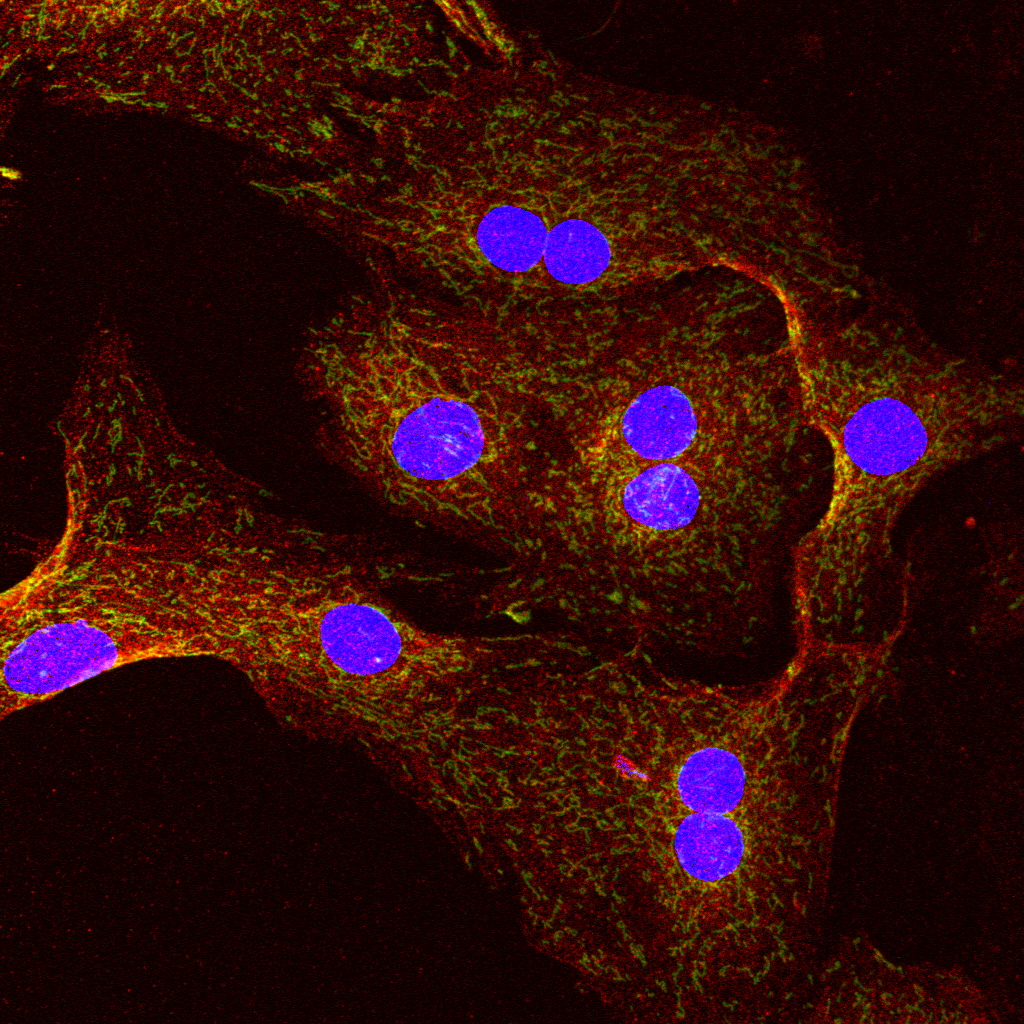

Supplement: Supplementary file 7 — Supplementary material [file mmc7.zip › GK Glucose Time Course - 3rd Experiment/E3-G3-7.tif]

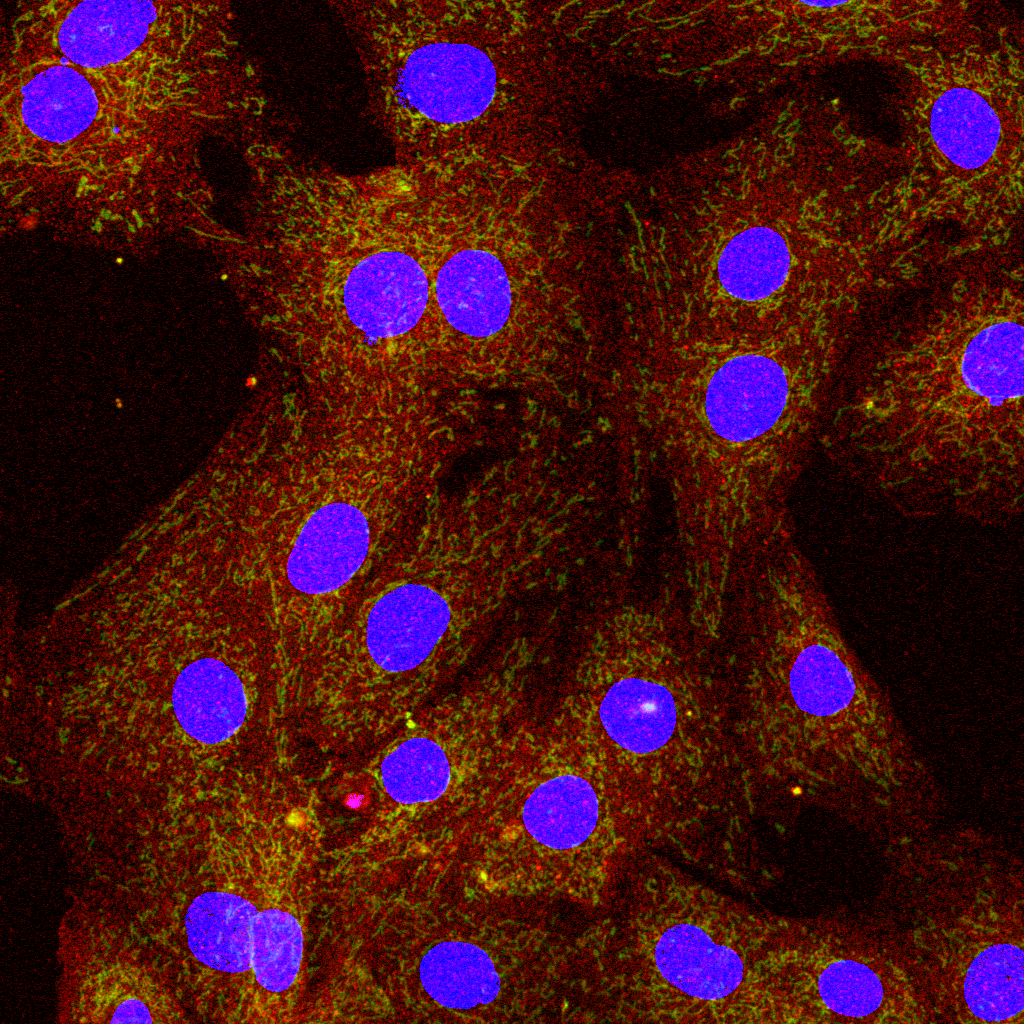

Supplement: Supplementary file 7 — Supplementary material [file mmc7.zip › GK Glucose Time Course - 3rd Experiment/E3-G3-8.tif]

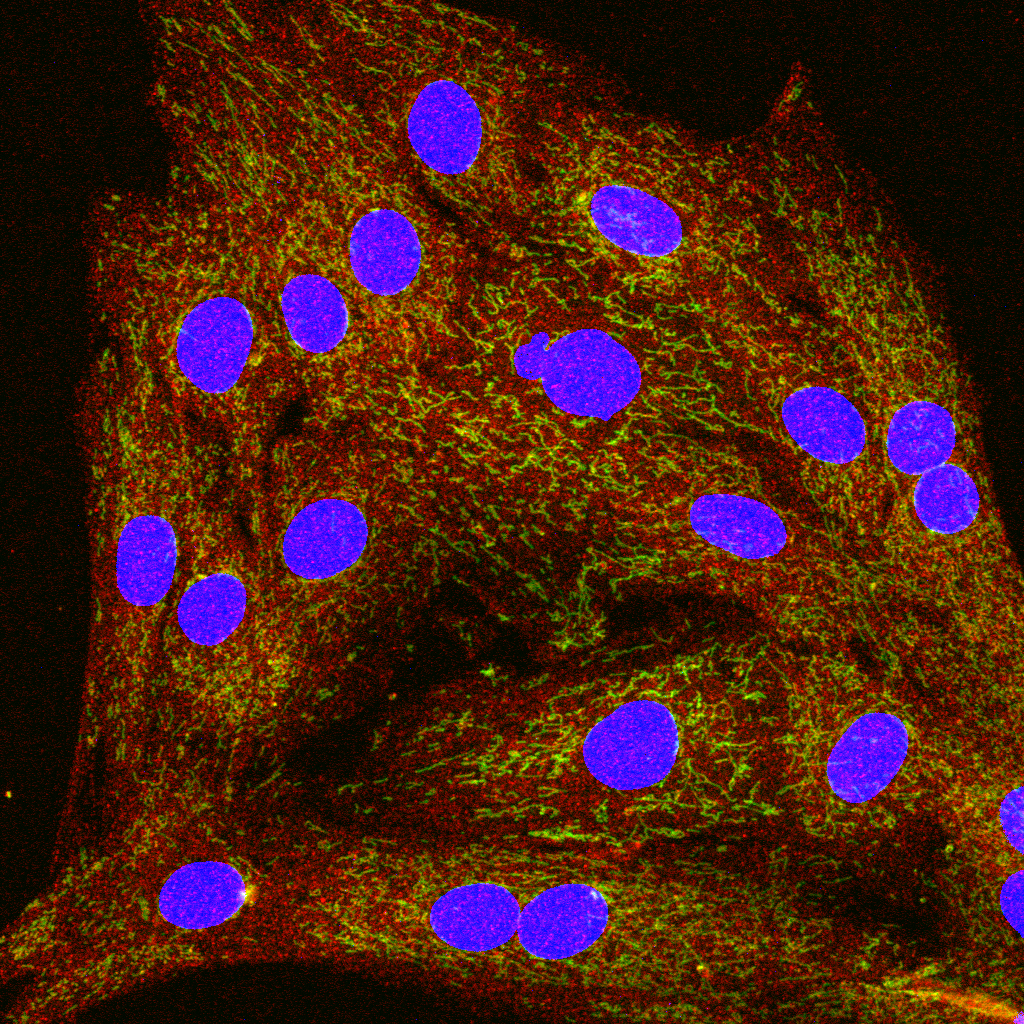

Supplement: Supplementary file 7 — Supplementary material [file mmc7.zip › GK Glucose Time Course - 3rd Experiment/E3-G3-9.tif]

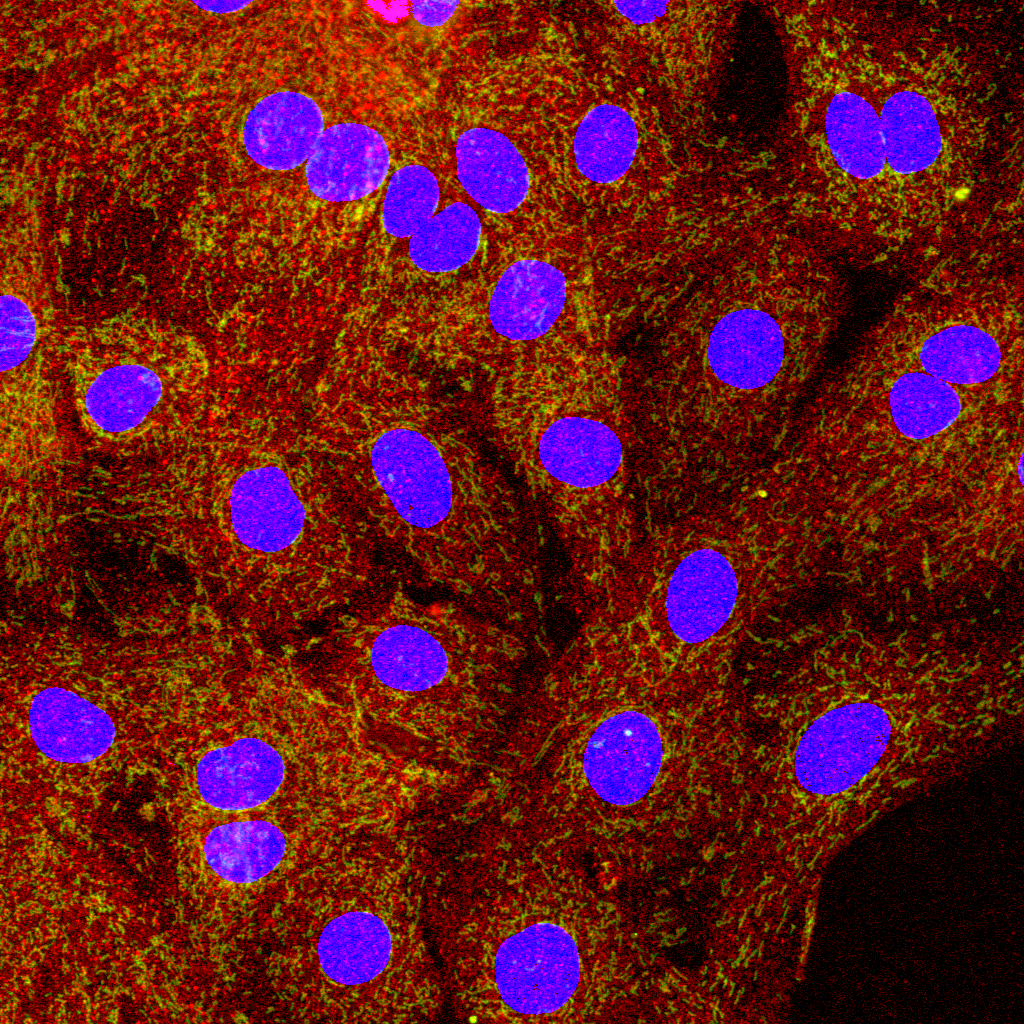

Supplement: Supplementary file 7 — Supplementary material [file mmc7.zip › GK Glucose Time Course - 3rd Experiment/E3-G4-1.tif]

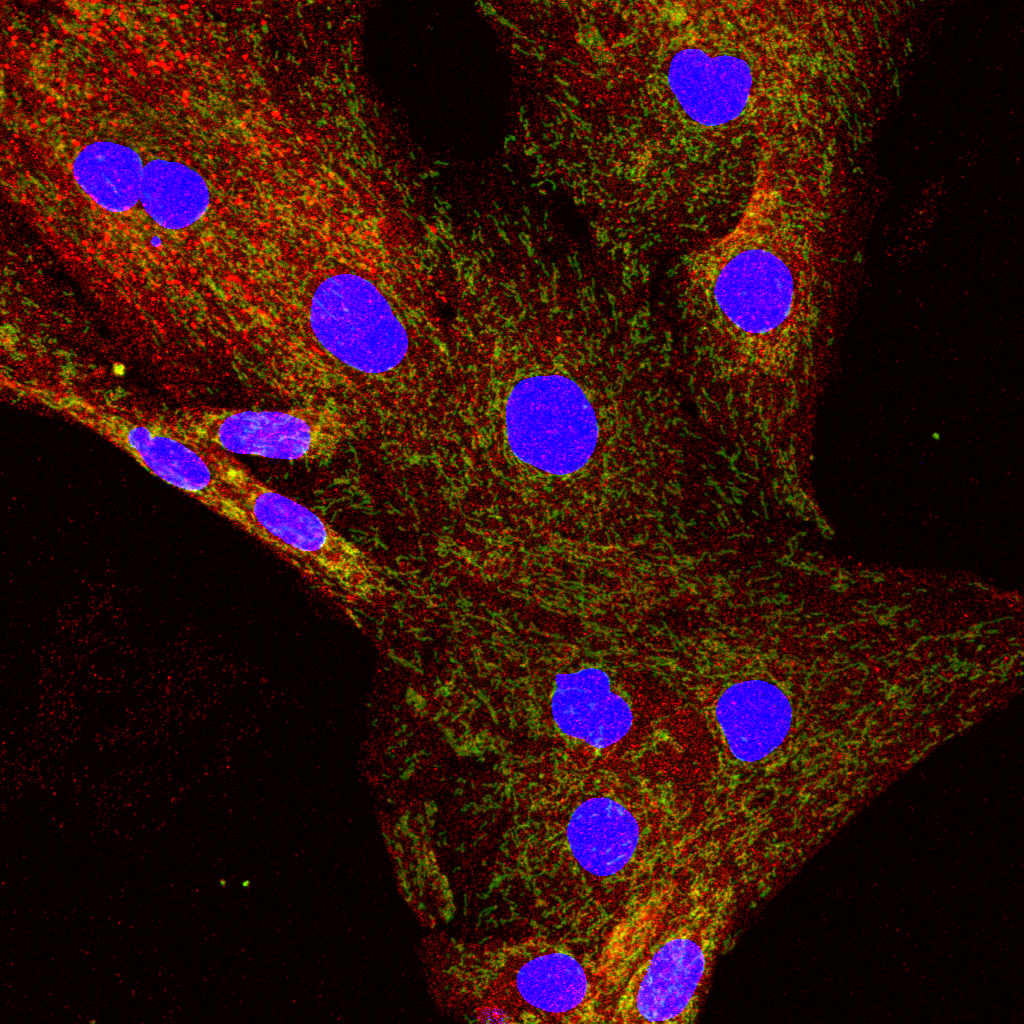

Supplement: Supplementary file 7 — Supplementary material [file mmc7.zip › GK Glucose Time Course - 3rd Experiment/E3-G4-2.tif]
