# Supplementary figures and images for: Raw and processed microscope images of fixed cells at baseline and following various experimental perturbations (part 4 of 4)
Source: Data Brief. 2016 Jan 29;6:998–1006. doi: 10.1016/j.dib.2016.01.044 (PMC4760184; doi:10.1016/j.dib.2016.01.044)

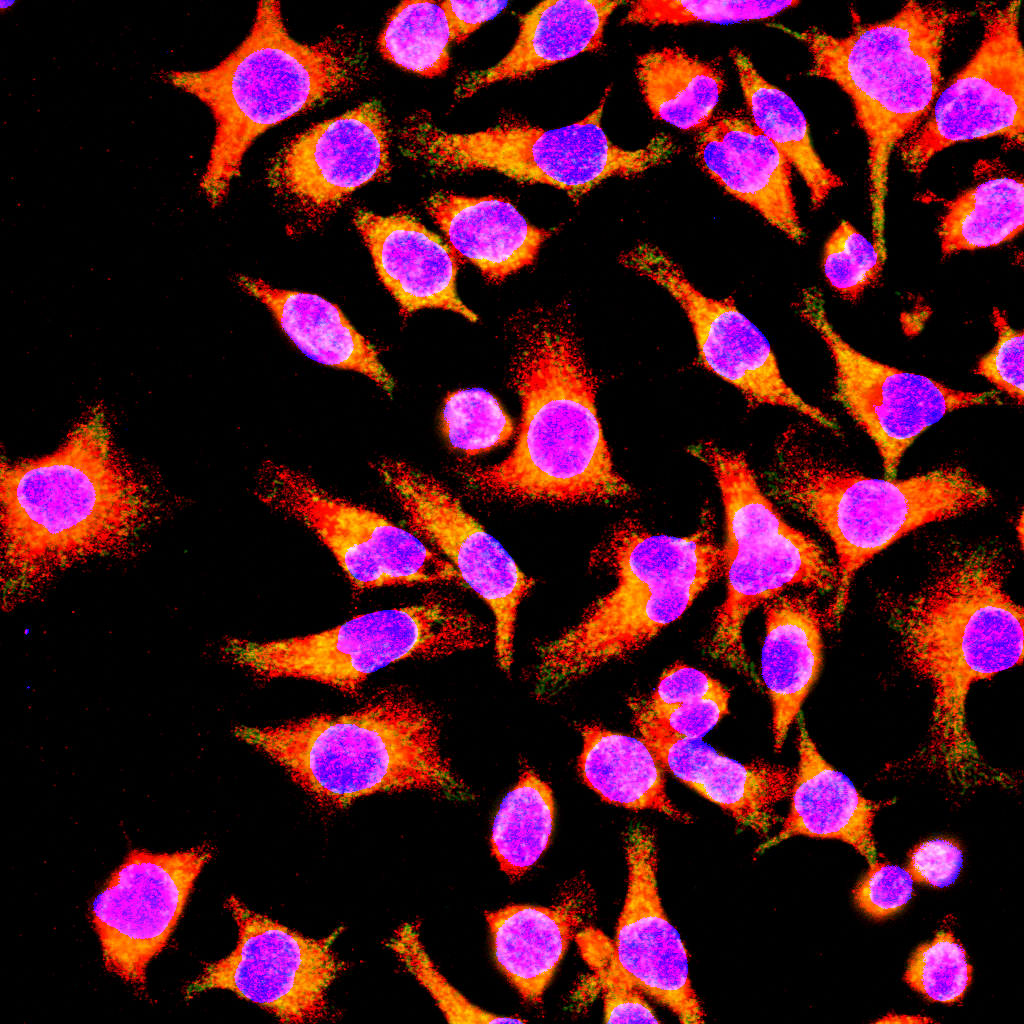

Supplement: Supplementary file 11 — Supplementary material [file mmc11.zip › SHSY5Y H2O2 Exposure - 1st Experiment/E1-G2-4.tif]

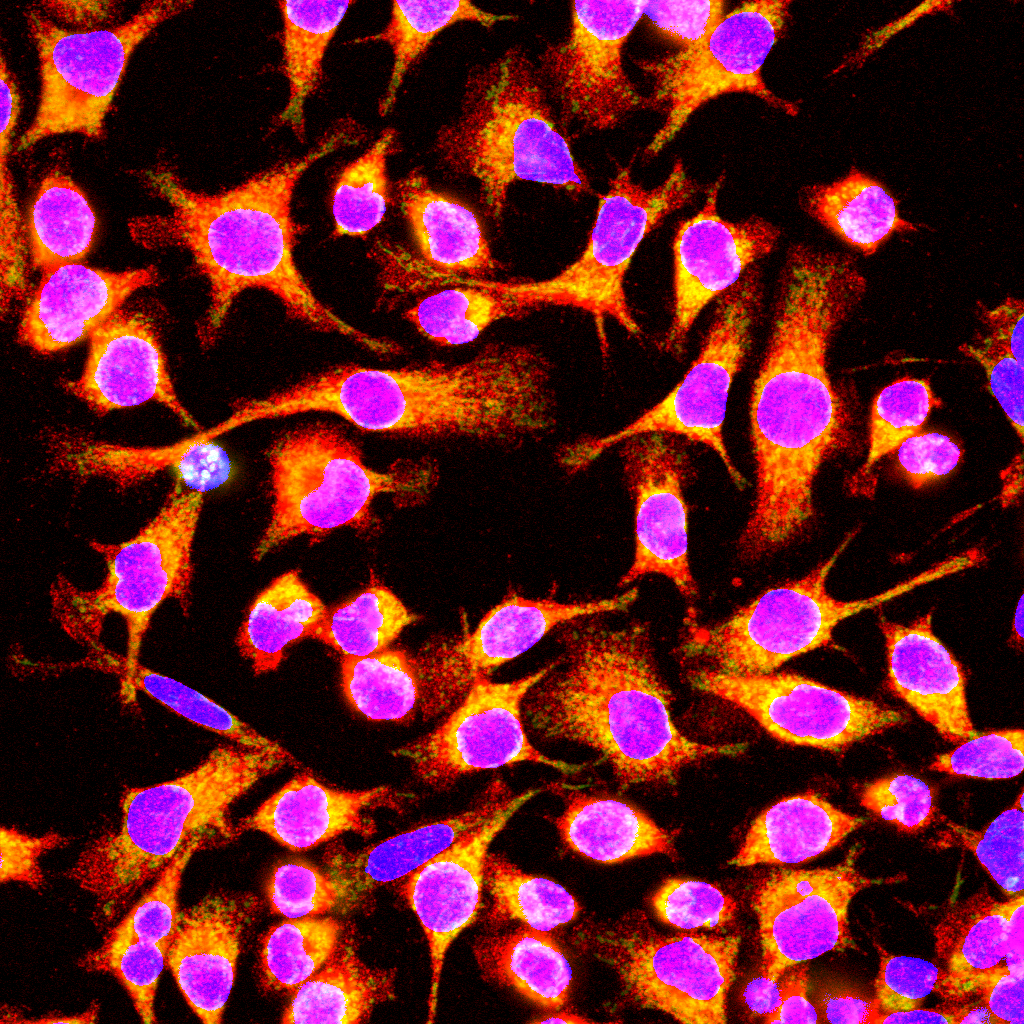

Supplement: Supplementary file 11 — Supplementary material [file mmc11.zip › SHSY5Y H2O2 Exposure - 1st Experiment/E1-G2-5.tif]

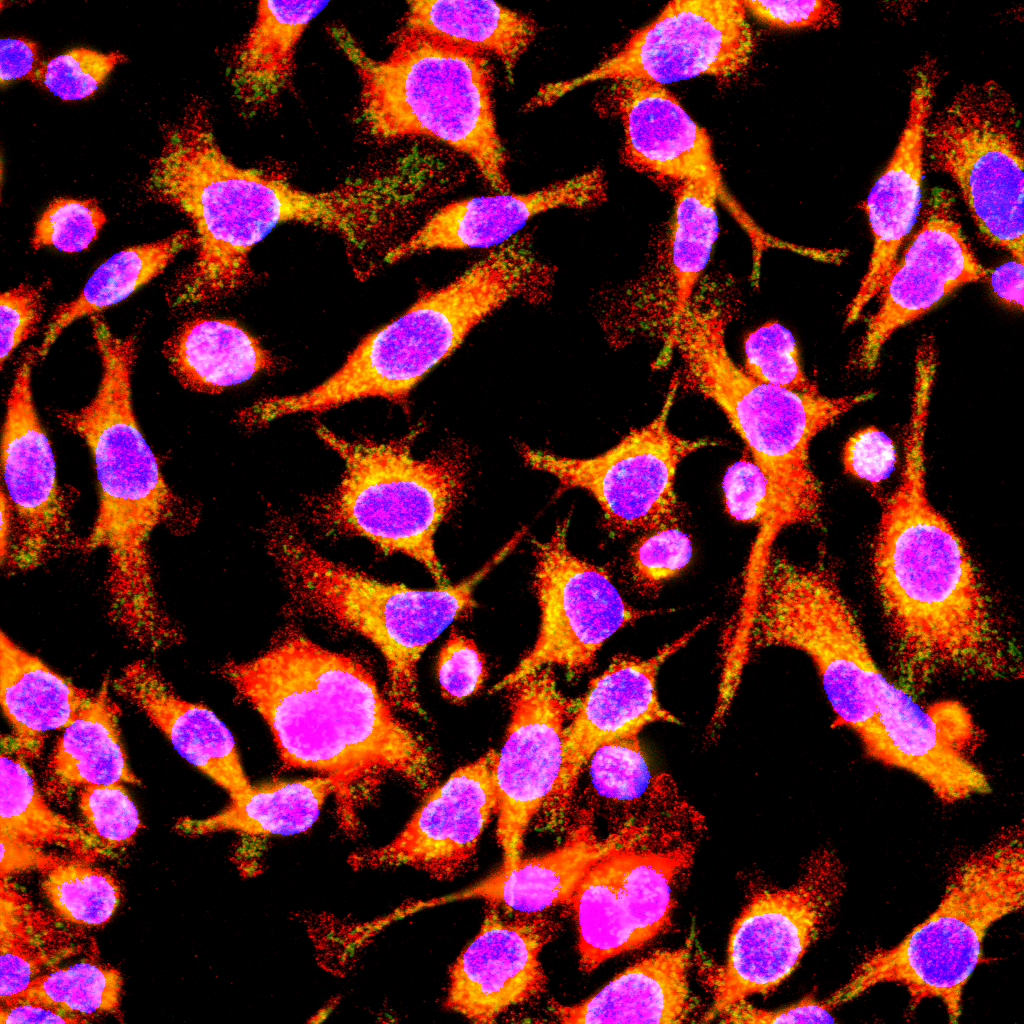

Supplement: Supplementary file 11 — Supplementary material [file mmc11.zip › SHSY5Y H2O2 Exposure - 1st Experiment/E1-G2-6.tif]

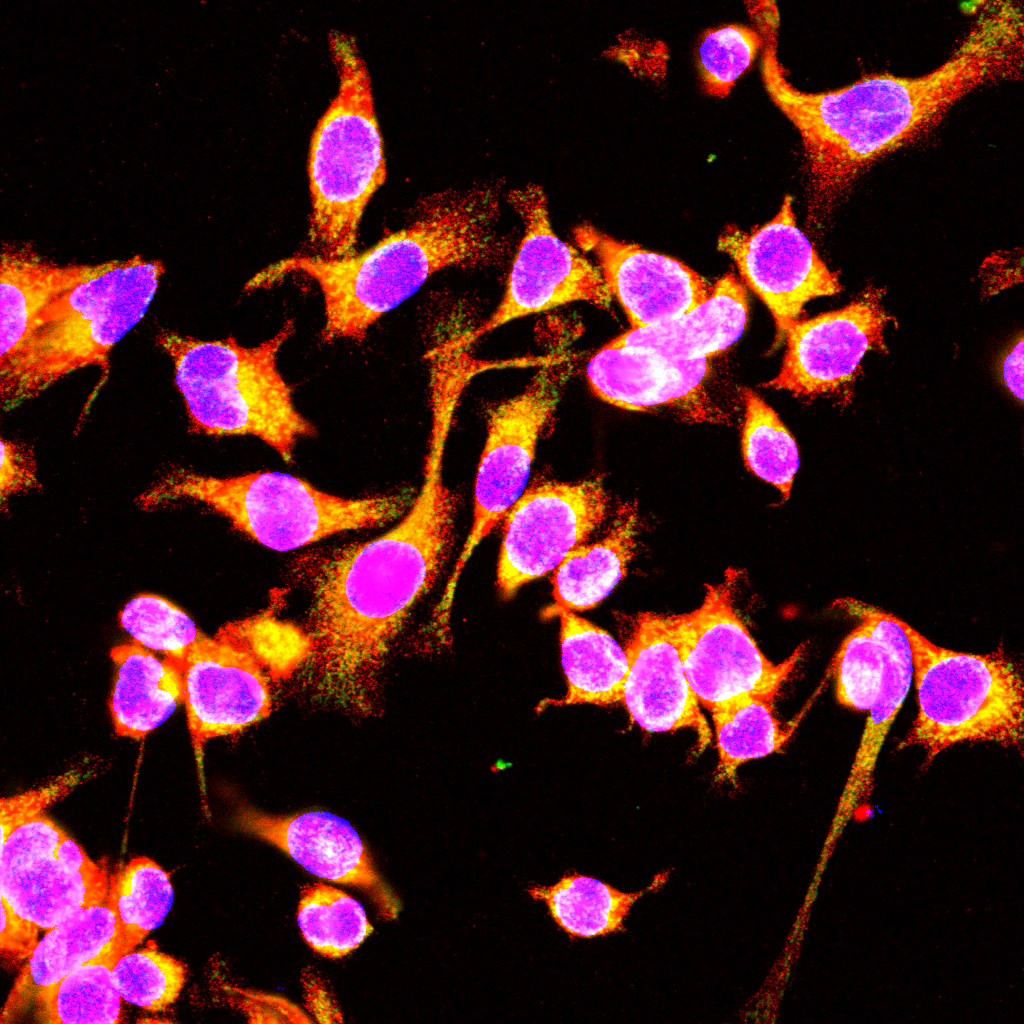

Supplement: Supplementary file 11 — Supplementary material [file mmc11.zip › SHSY5Y H2O2 Exposure - 1st Experiment/E1-G2-7.tif]

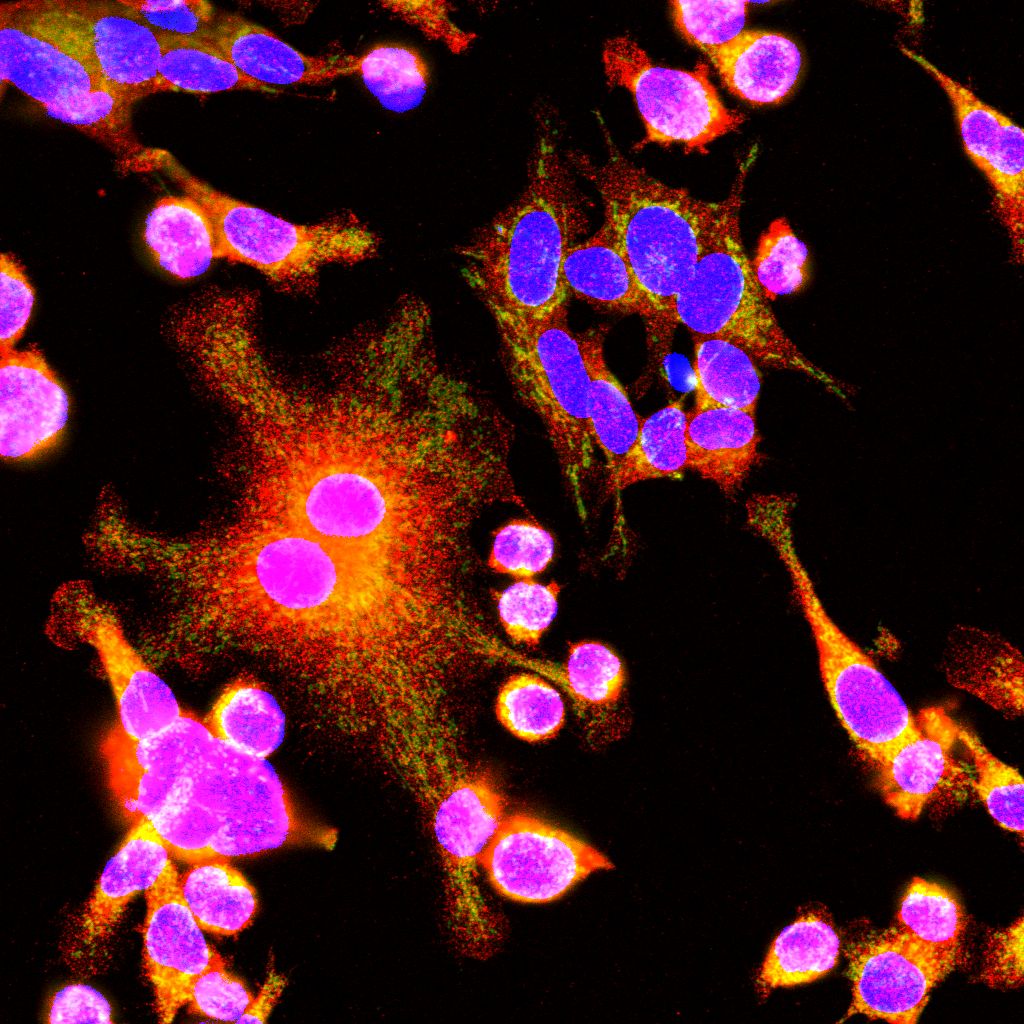

Supplement: Supplementary file 11 — Supplementary material [file mmc11.zip › SHSY5Y H2O2 Exposure - 1st Experiment/E1-G2-8.tif]

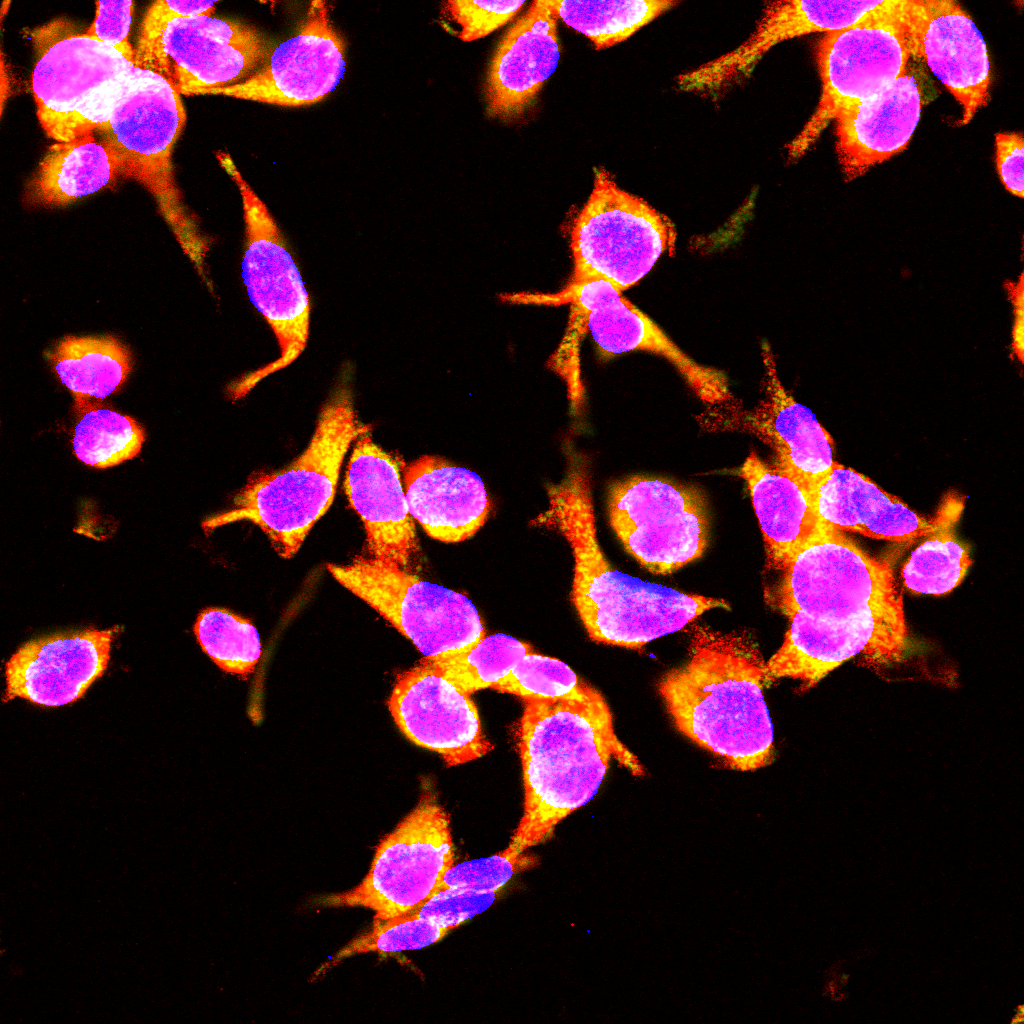

Supplement: Supplementary file 11 — Supplementary material [file mmc11.zip › SHSY5Y H2O2 Exposure - 1st Experiment/E1-G2-9.tif]

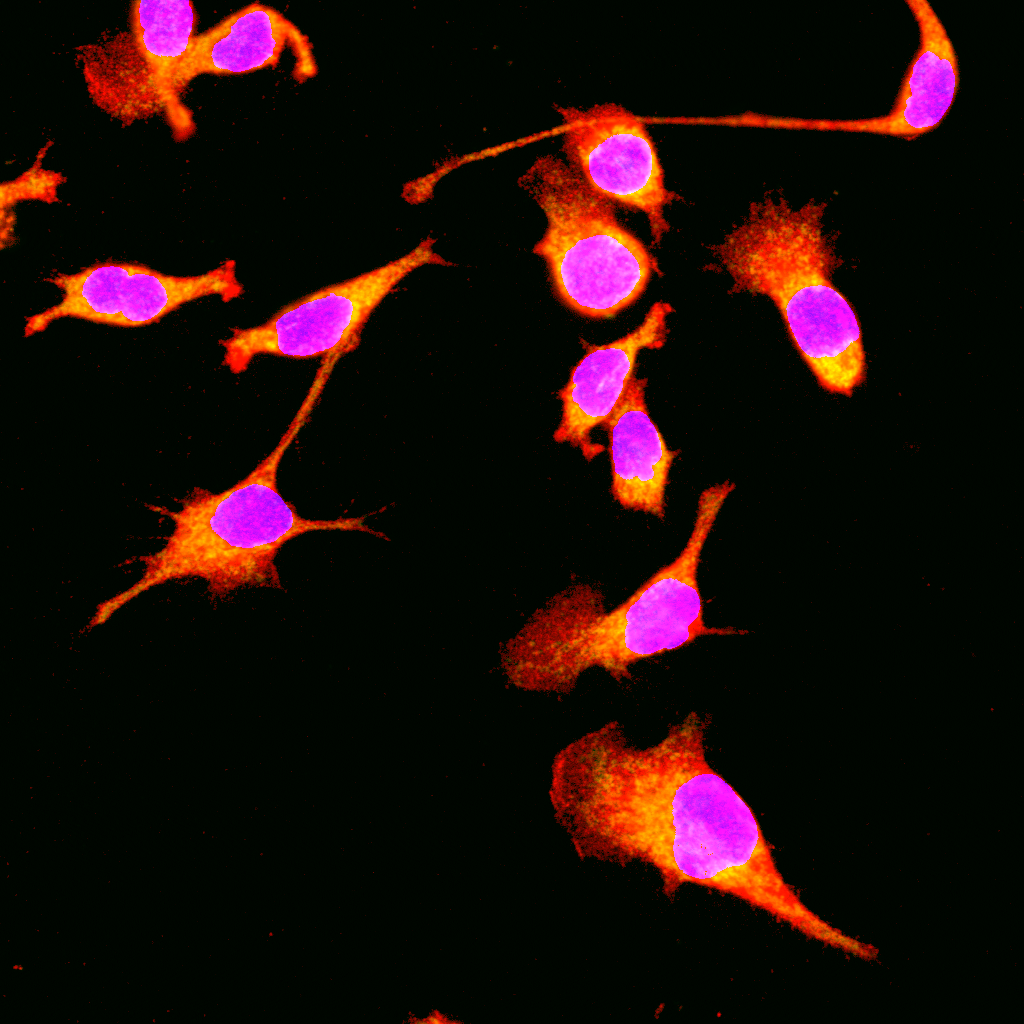

Supplement: Supplementary file 12 — Supplementary material [file mmc12.zip › SHSY5Y H2O2 Exposure - 2nd Experiment/E2-G2-1.tif]

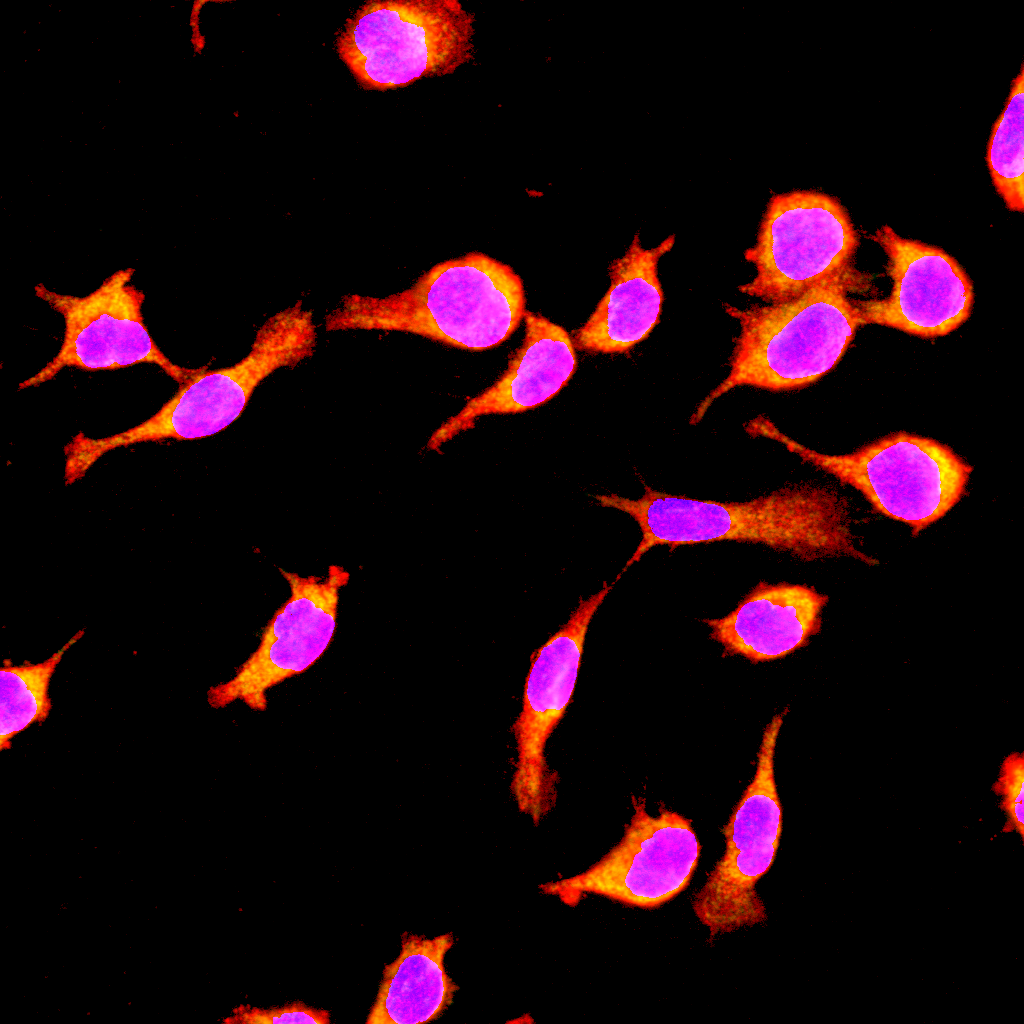

Supplement: Supplementary file 12 — Supplementary material [file mmc12.zip › SHSY5Y H2O2 Exposure - 2nd Experiment/E2-G2-2.tif]

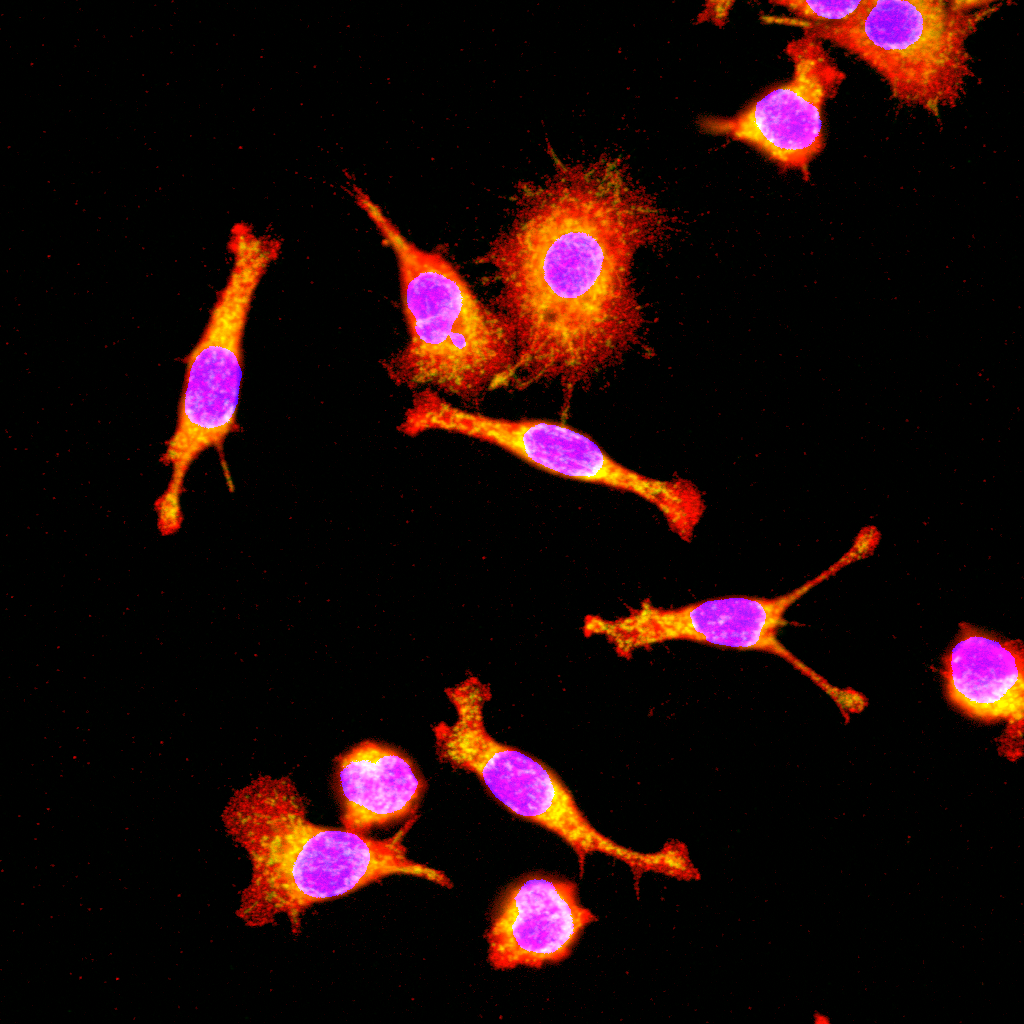

Supplement: Supplementary file 12 — Supplementary material [file mmc12.zip › SHSY5Y H2O2 Exposure - 2nd Experiment/E2-G2-3.tif]

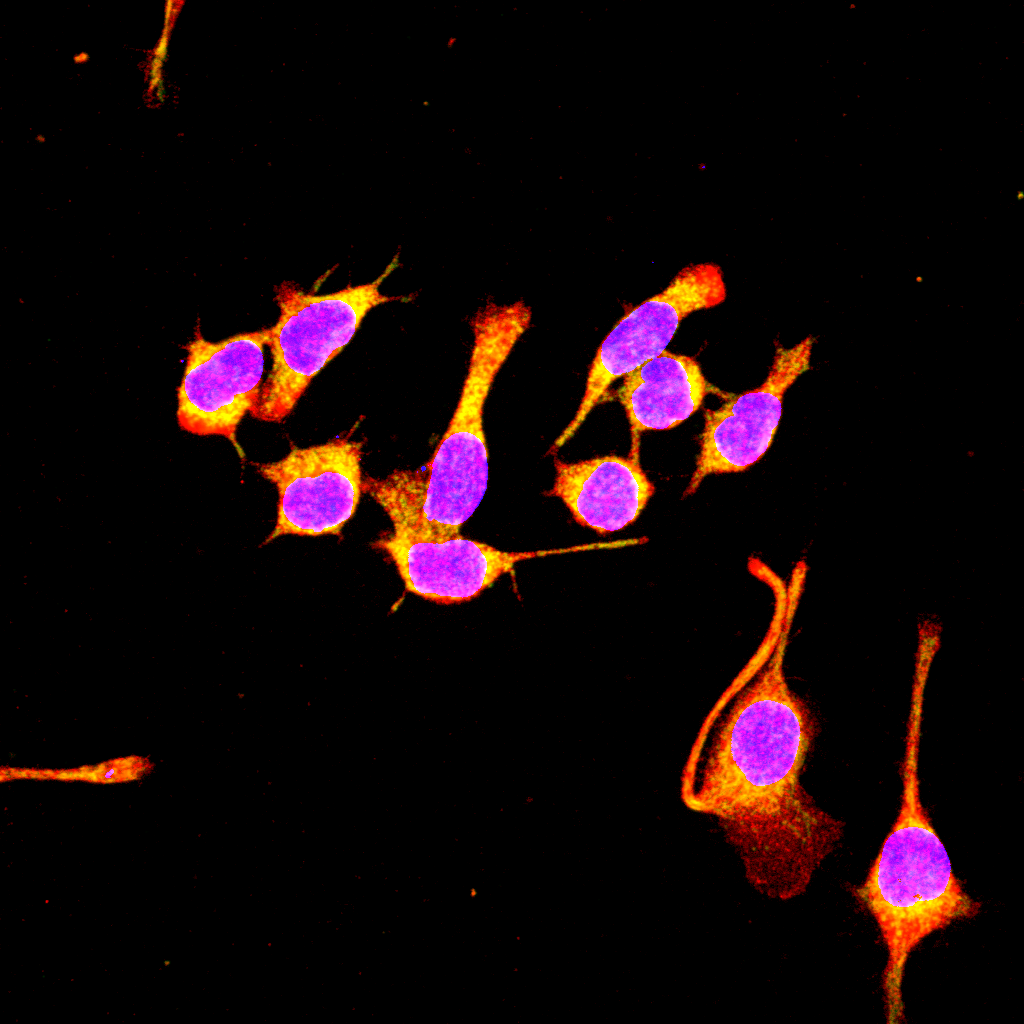

Supplement: Supplementary file 12 — Supplementary material [file mmc12.zip › SHSY5Y H2O2 Exposure - 2nd Experiment/E2-G2-4.tif]

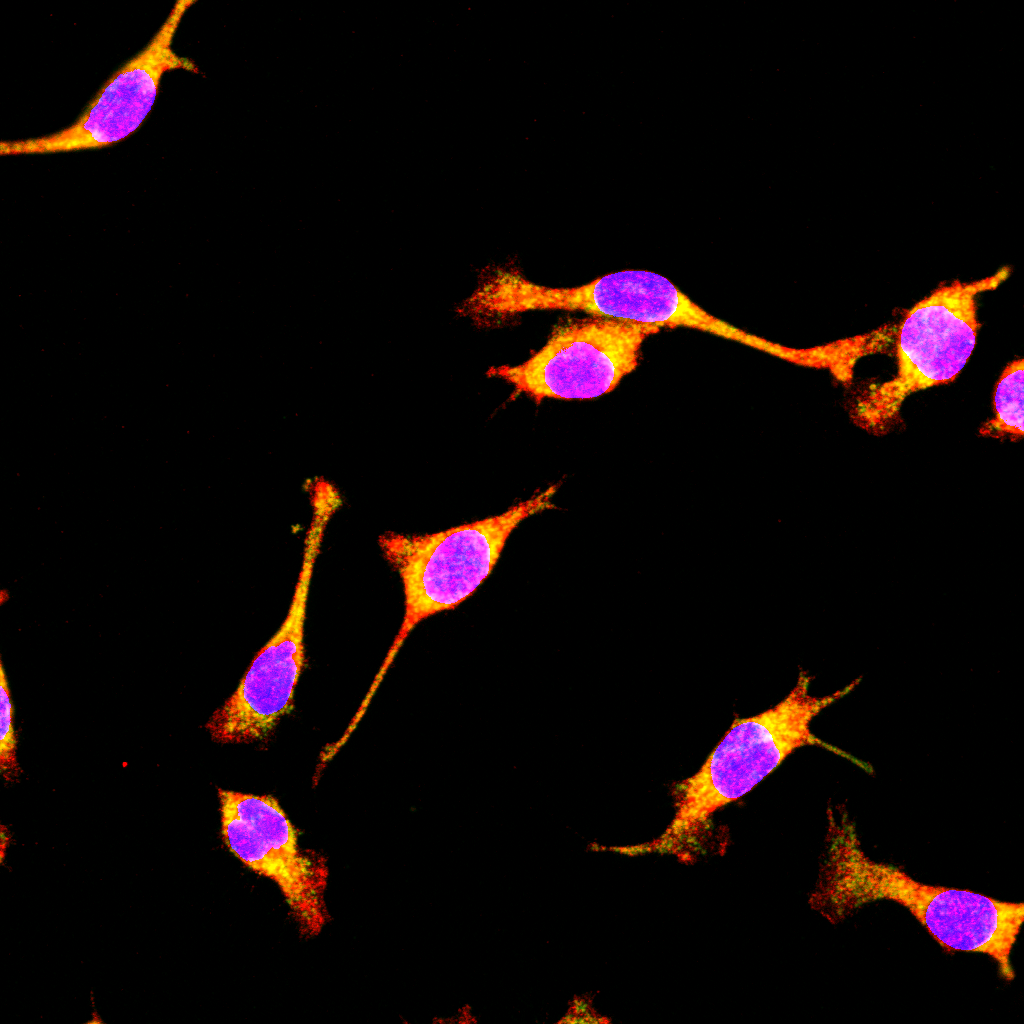

Supplement: Supplementary file 12 — Supplementary material [file mmc12.zip › SHSY5Y H2O2 Exposure - 2nd Experiment/E2-G2-5.tif]

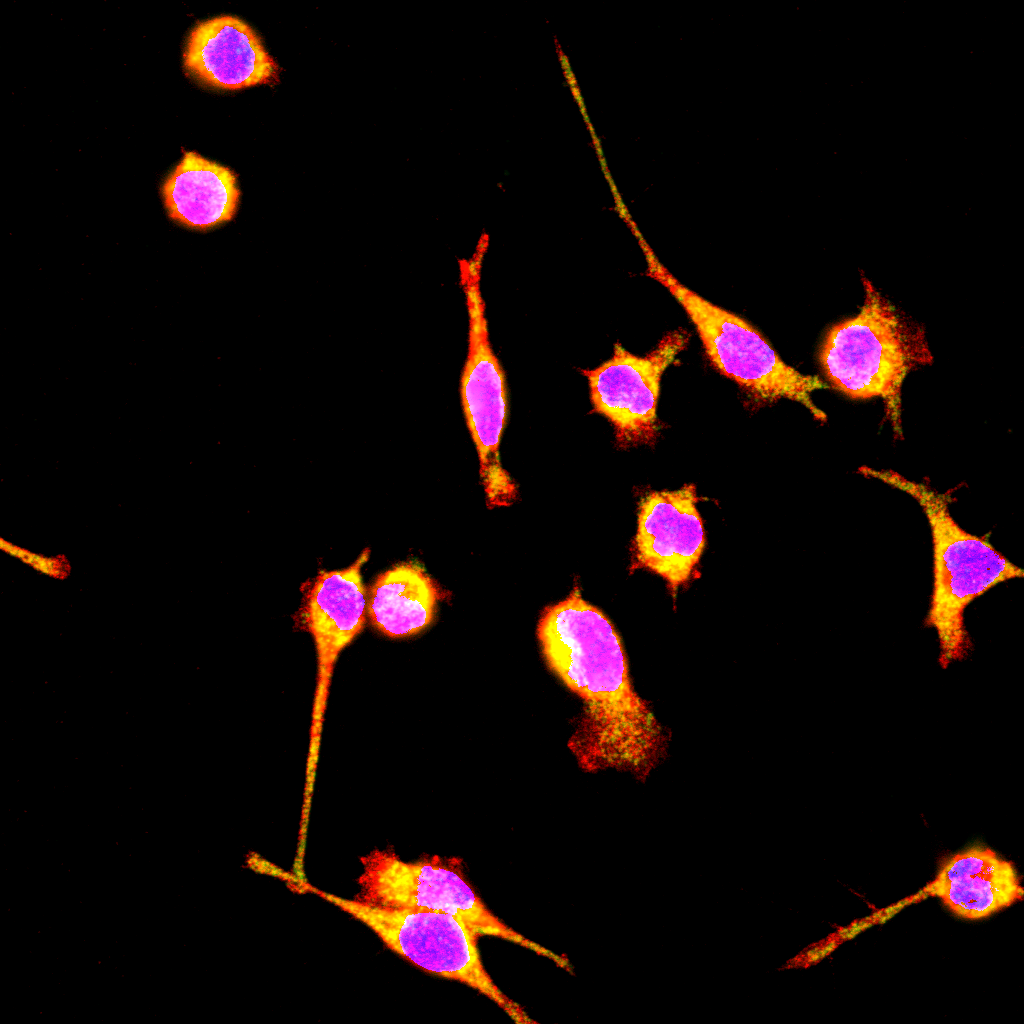

Supplement: Supplementary file 12 — Supplementary material [file mmc12.zip › SHSY5Y H2O2 Exposure - 2nd Experiment/E2-G2-6.tif]

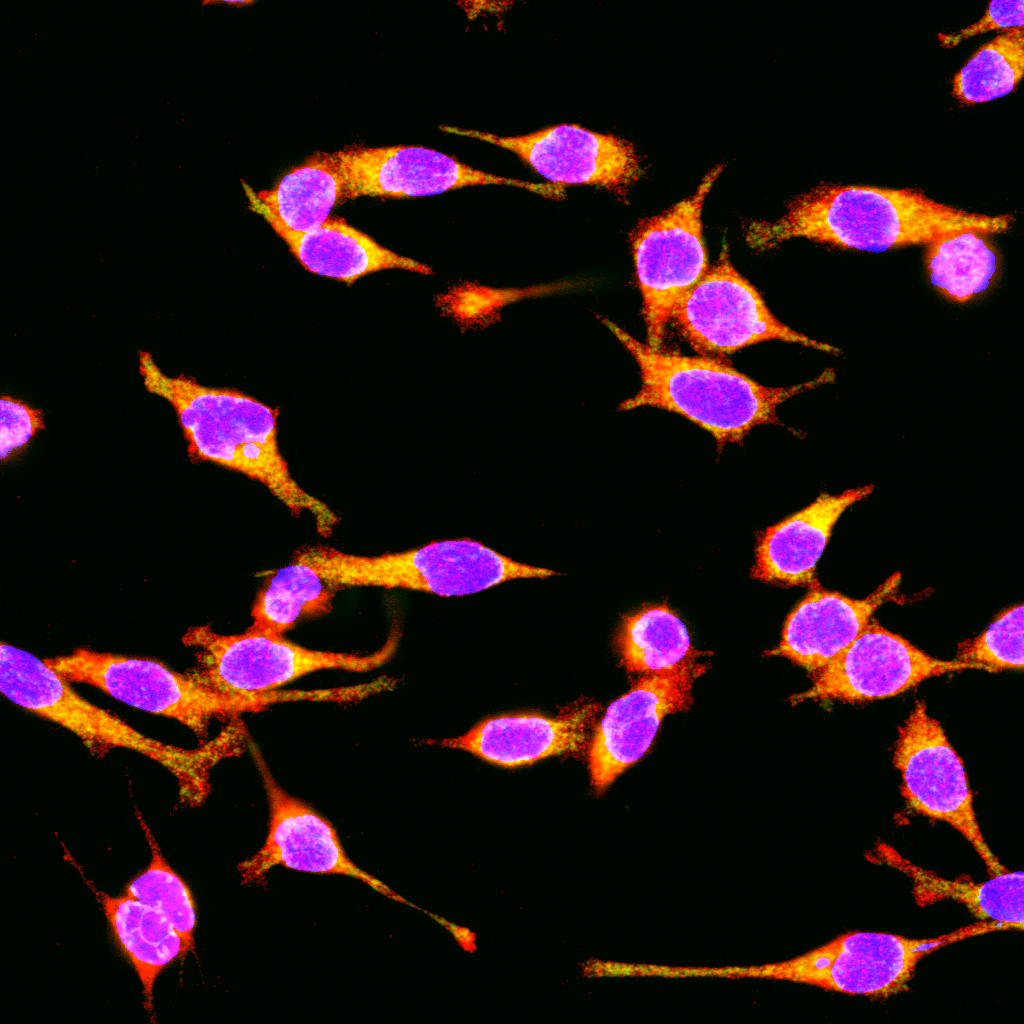

Supplement: Supplementary file 12 — Supplementary material [file mmc12.zip › SHSY5Y H2O2 Exposure - 2nd Experiment/E2-G2-7.tif]

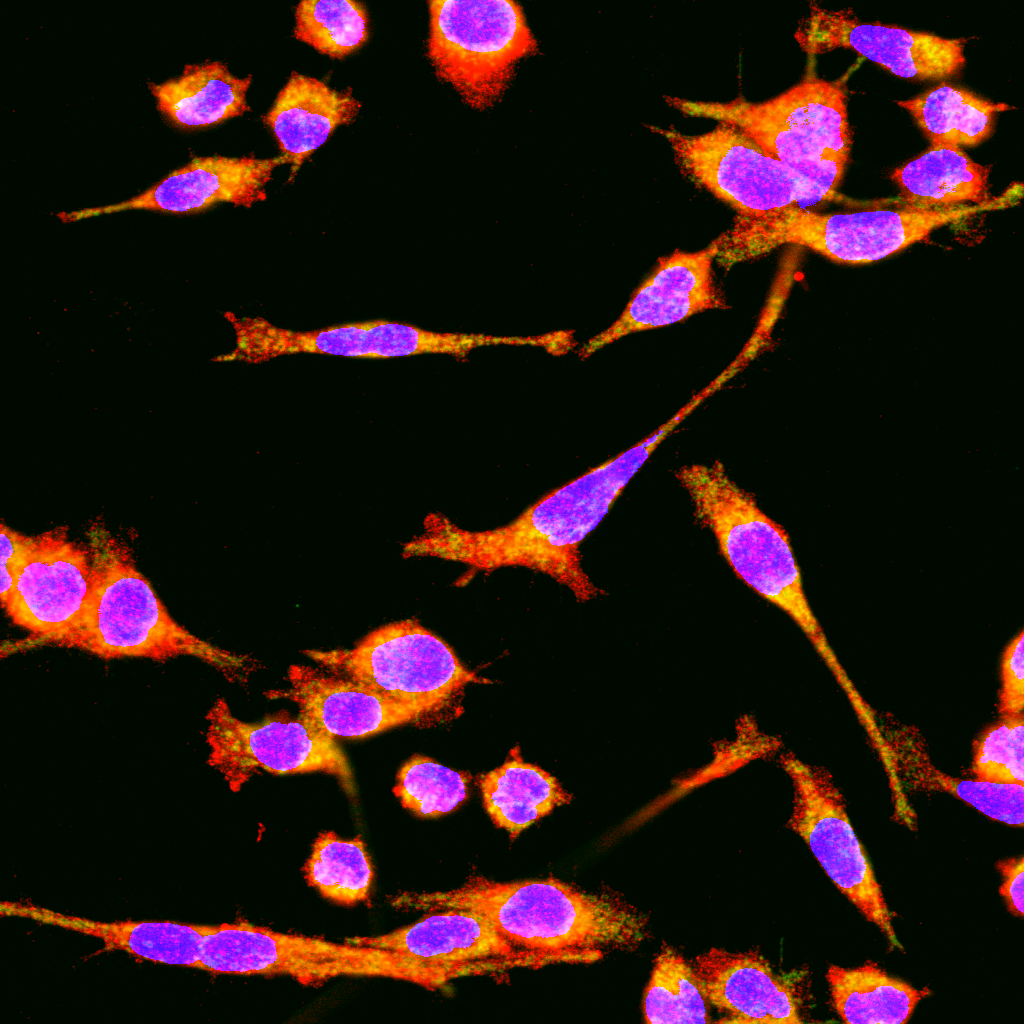

Supplement: Supplementary file 12 — Supplementary material [file mmc12.zip › SHSY5Y H2O2 Exposure - 2nd Experiment/E2-G2-8.tif]

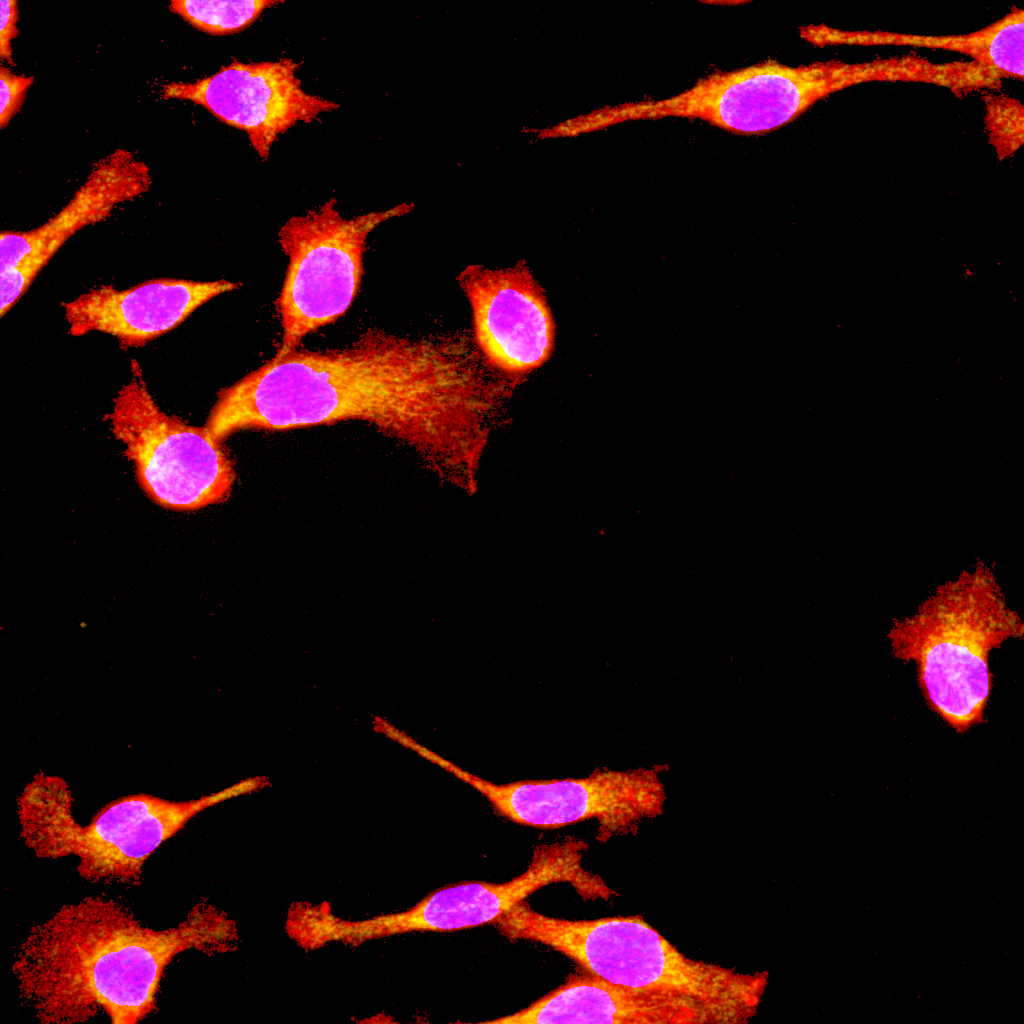

Supplement: Supplementary file 12 — Supplementary material [file mmc12.zip › SHSY5Y H2O2 Exposure - 2nd Experiment/E2-G2-9.tif]

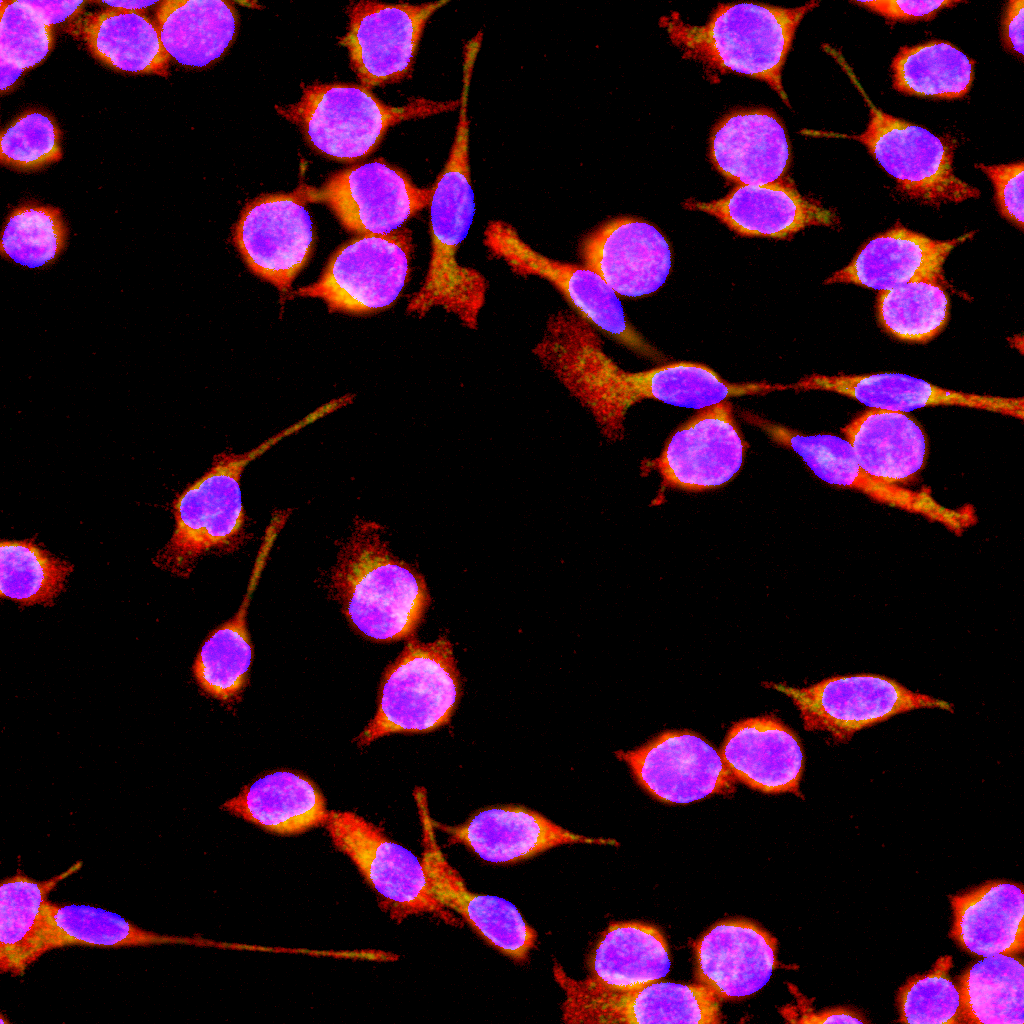

Supplement: Supplementary file 13 — Supplementary material [file mmc13.zip › SHSY5Y H2O2 Exposure - 3rd Experiment/E3-G2-1.tif]

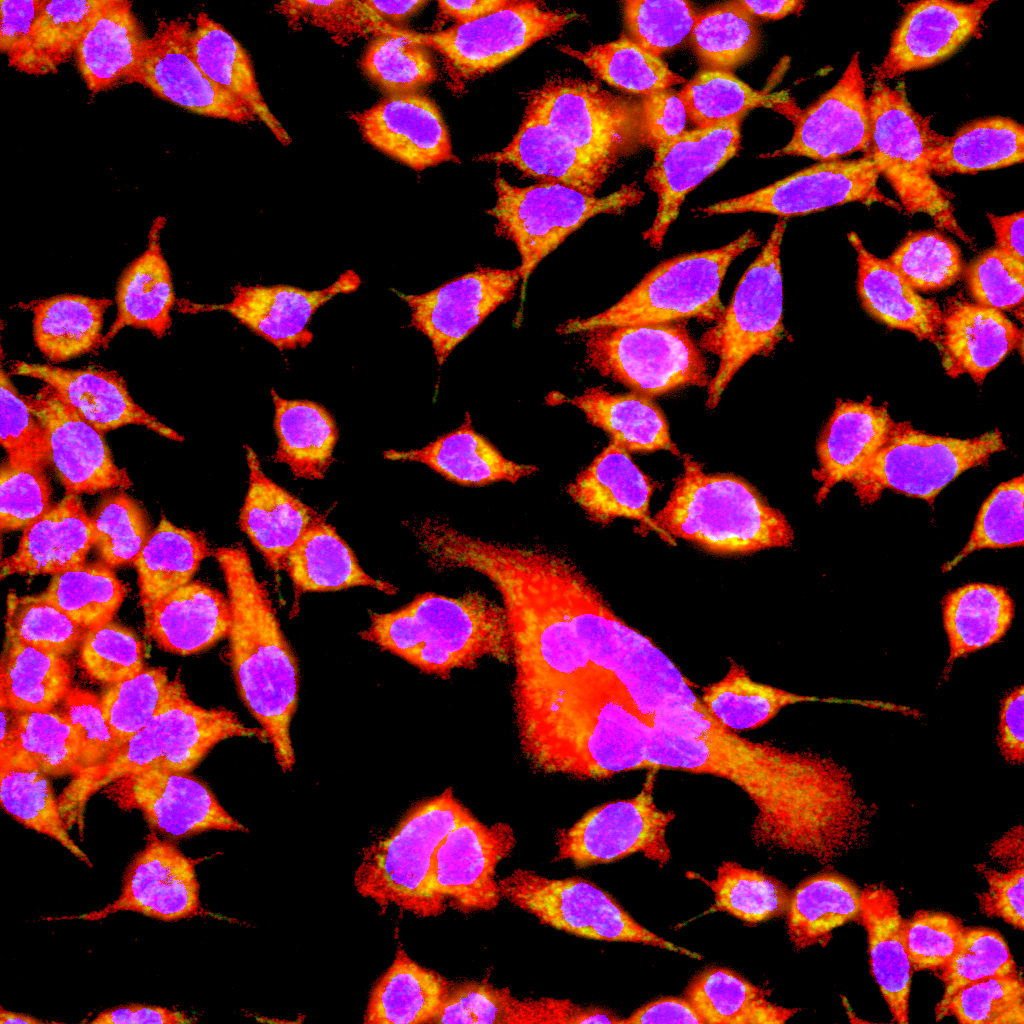

Supplement: Supplementary file 13 — Supplementary material [file mmc13.zip › SHSY5Y H2O2 Exposure - 3rd Experiment/E3-G2-2.tif]

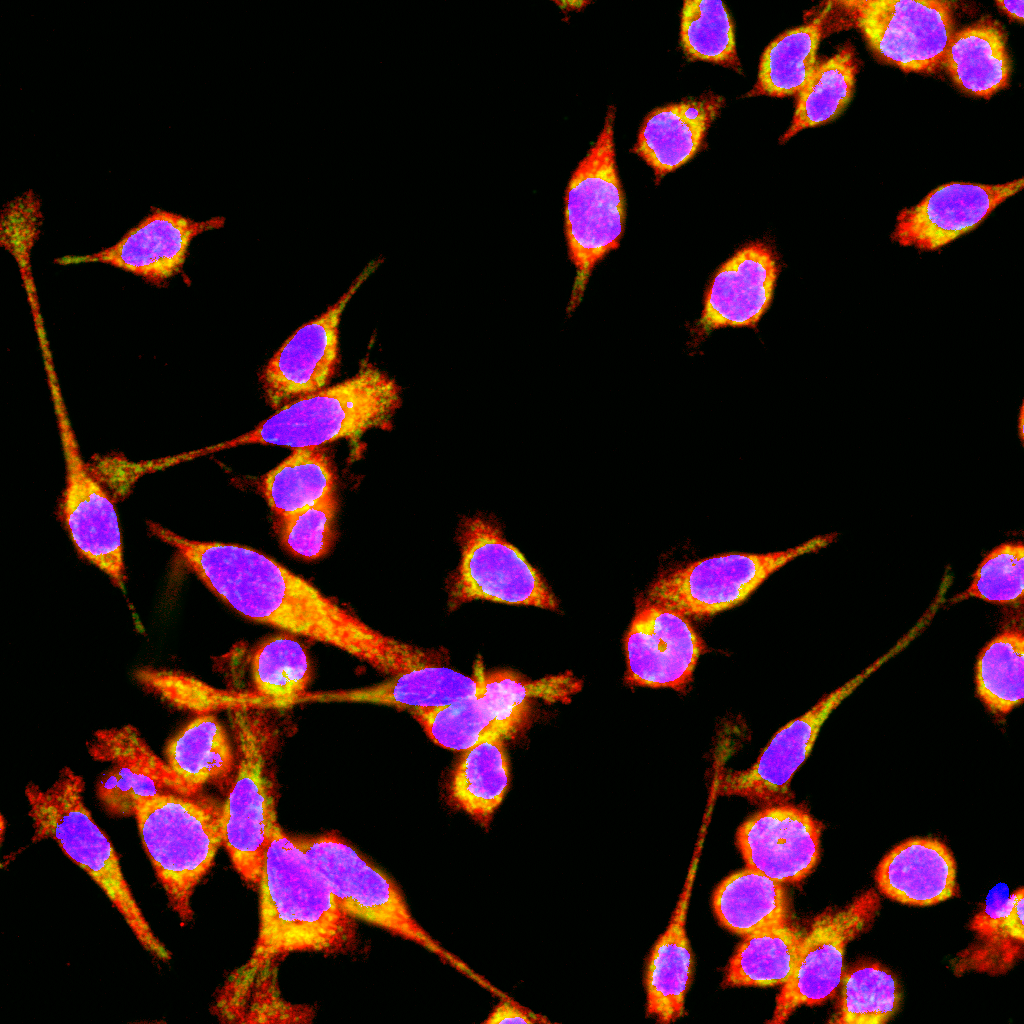

Supplement: Supplementary file 13 — Supplementary material [file mmc13.zip › SHSY5Y H2O2 Exposure - 3rd Experiment/E3-G2-3.tif]

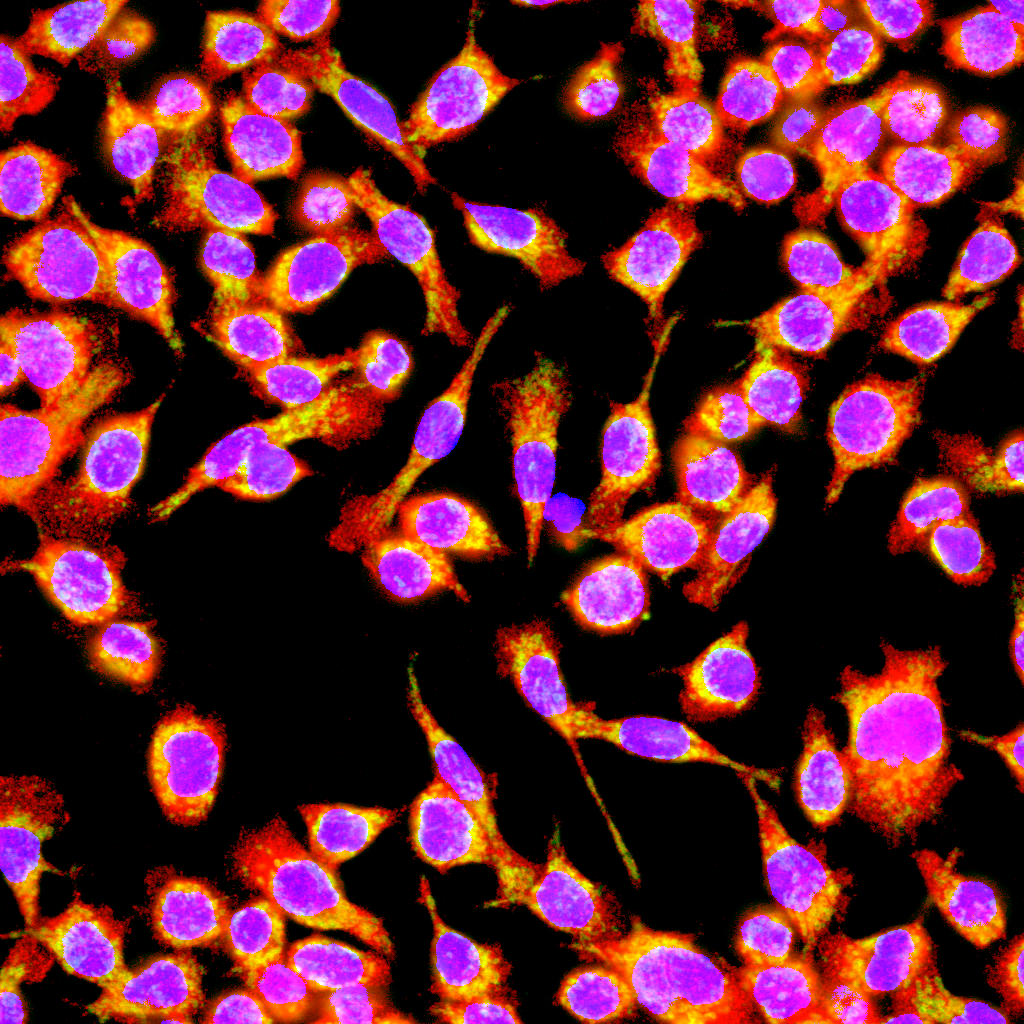

Supplement: Supplementary file 13 — Supplementary material [file mmc13.zip › SHSY5Y H2O2 Exposure - 3rd Experiment/E3-G2-4.tif]

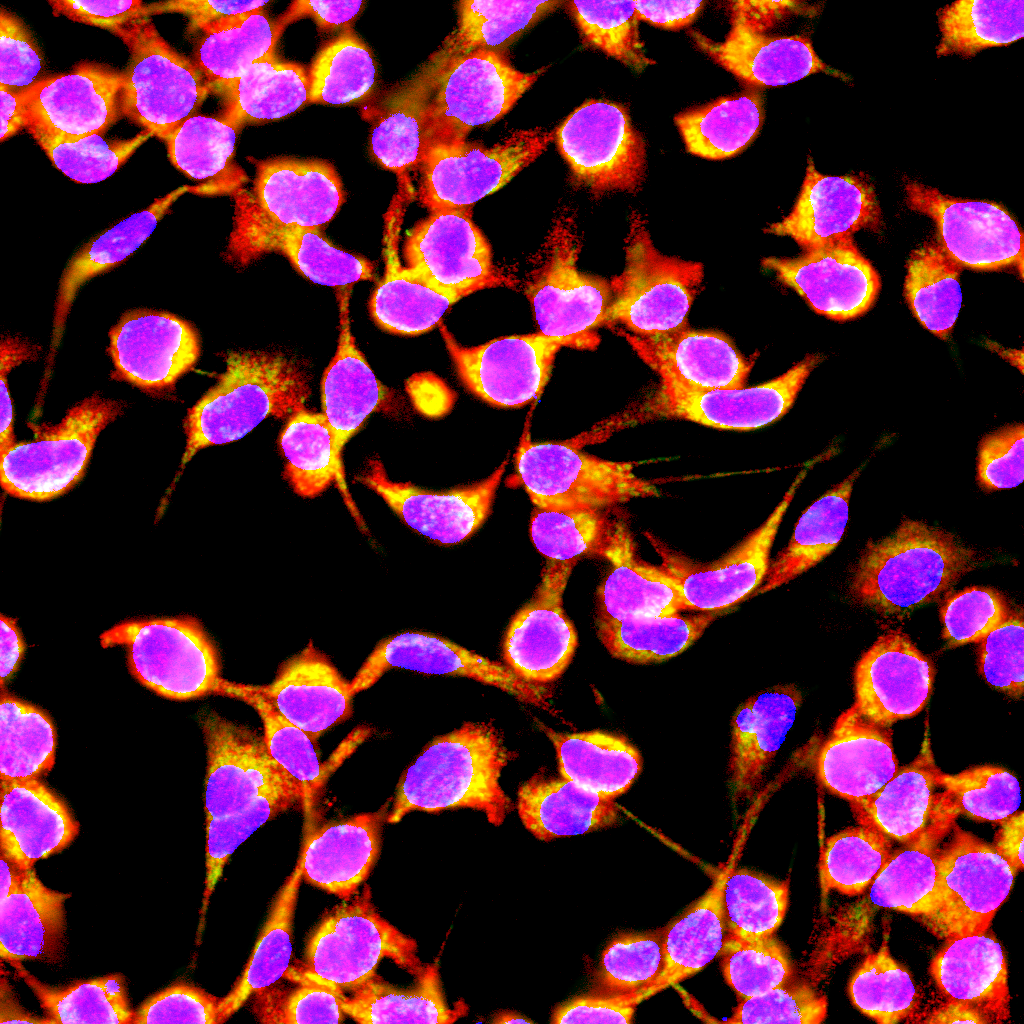

Supplement: Supplementary file 13 — Supplementary material [file mmc13.zip › SHSY5Y H2O2 Exposure - 3rd Experiment/E3-G2-5.tif]

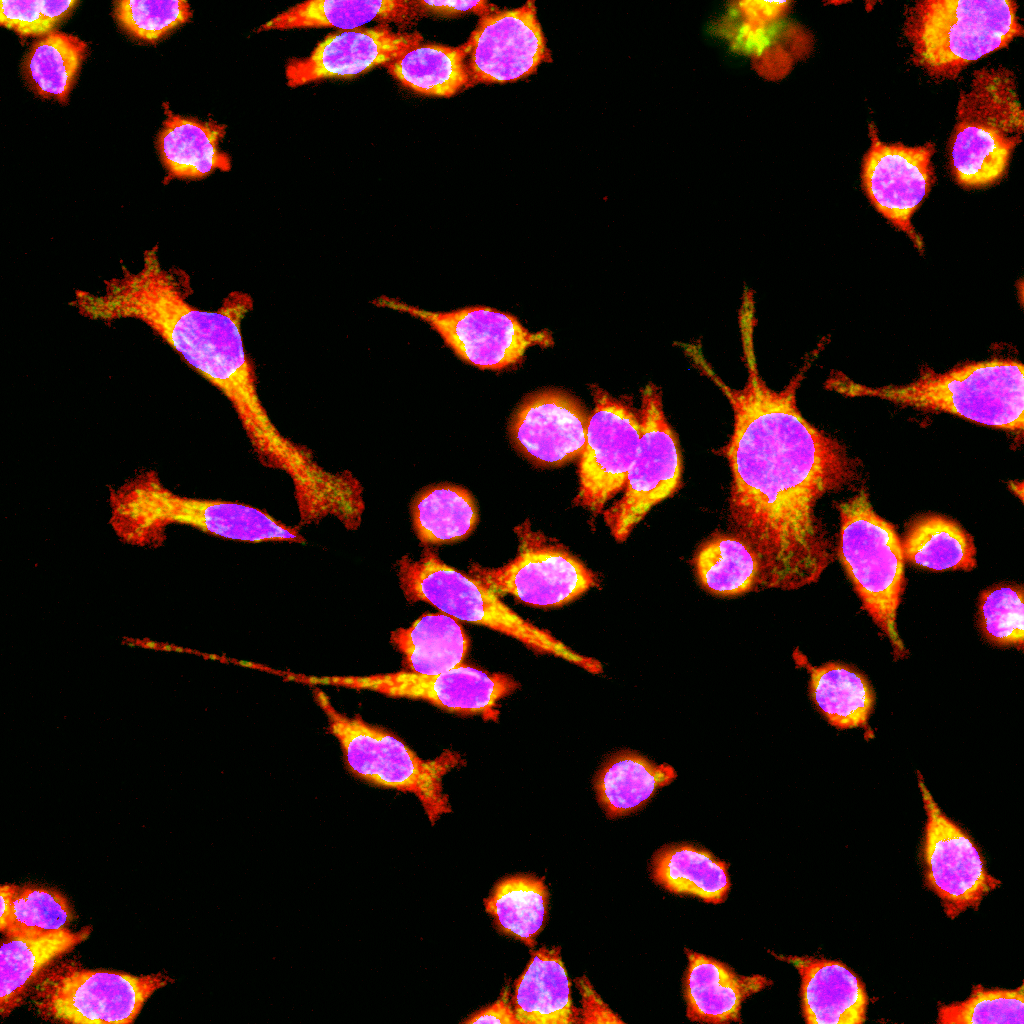

Supplement: Supplementary file 13 — Supplementary material [file mmc13.zip › SHSY5Y H2O2 Exposure - 3rd Experiment/E3-G2-6.tif]

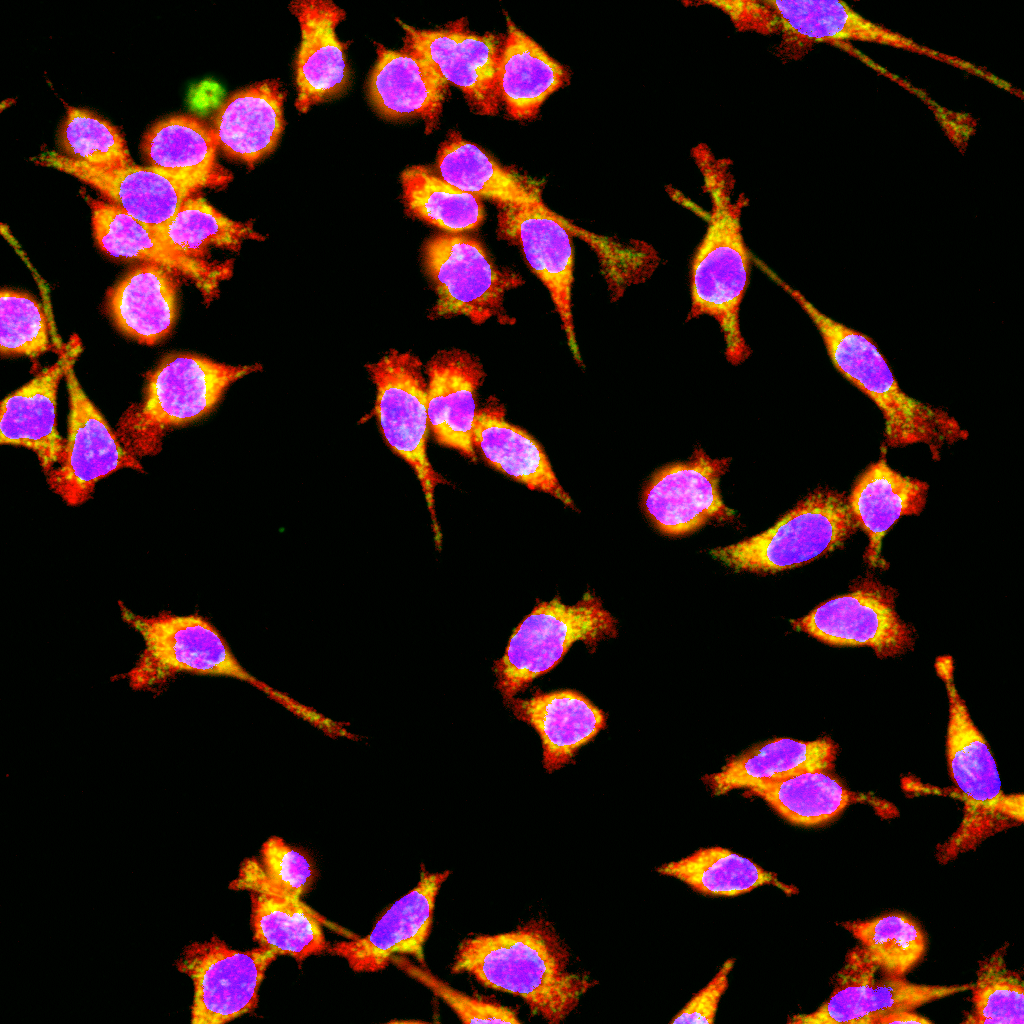

Supplement: Supplementary file 13 — Supplementary material [file mmc13.zip › SHSY5Y H2O2 Exposure - 3rd Experiment/E3-G2-7.tif]

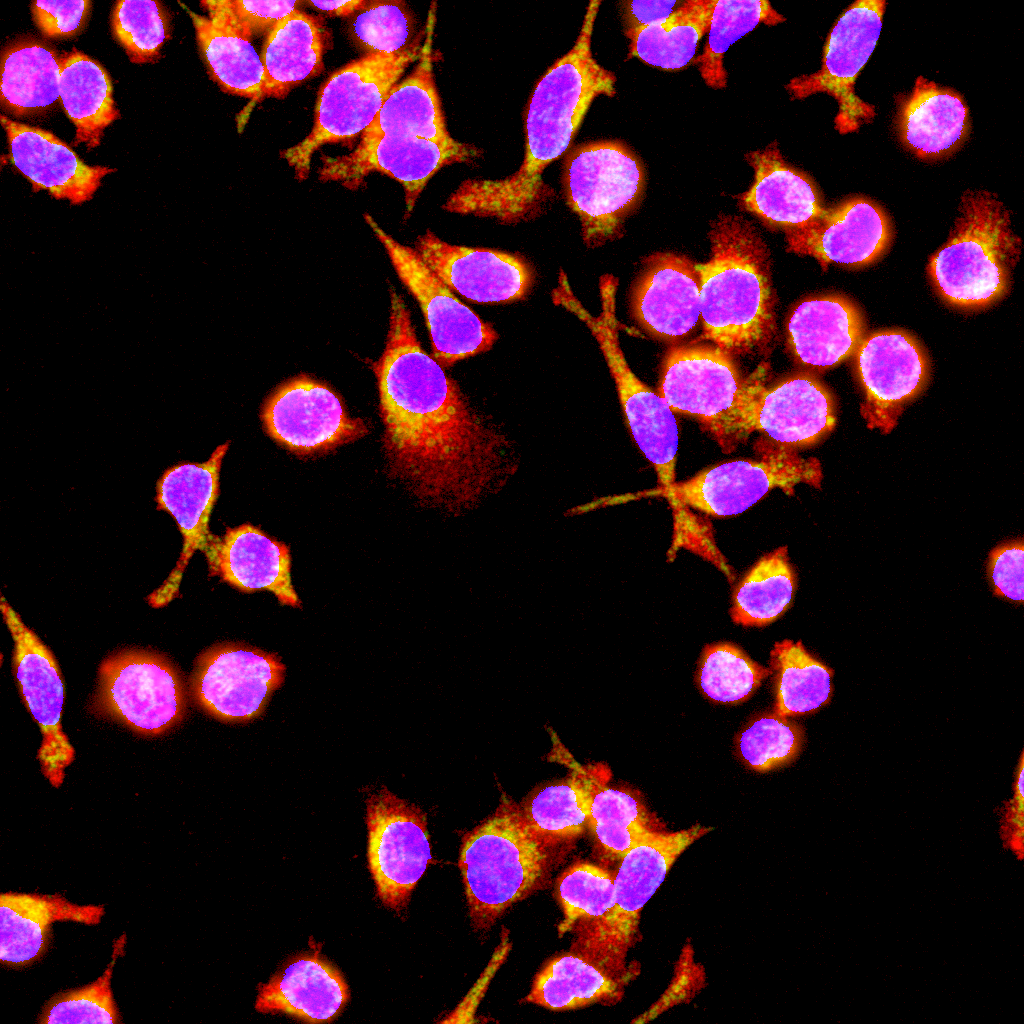

Supplement: Supplementary file 13 — Supplementary material [file mmc13.zip › SHSY5Y H2O2 Exposure - 3rd Experiment/E3-G2-8.tif]

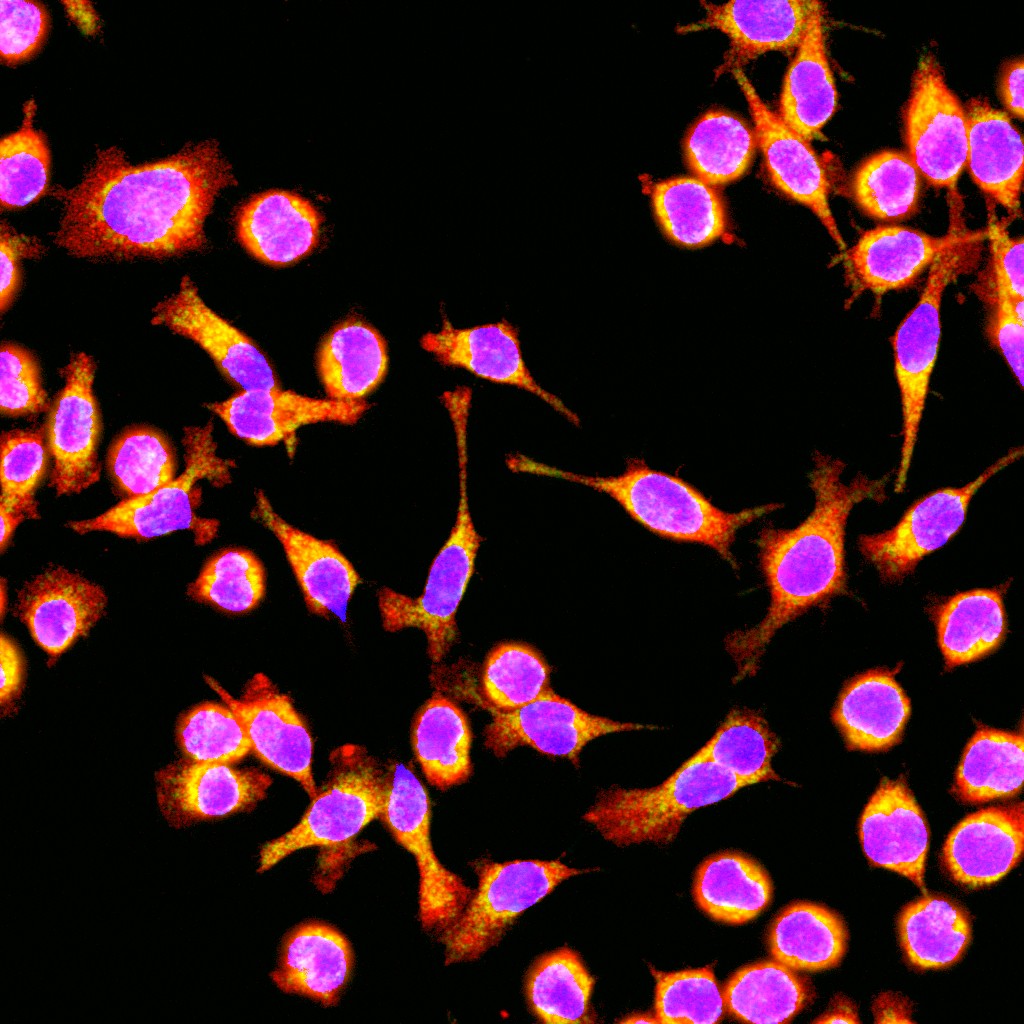

Supplement: Supplementary file 13 — Supplementary material [file mmc13.zip › SHSY5Y H2O2 Exposure - 3rd Experiment/E3-G2-9.tif]
