# Supplementary material for: Raw and processed microscope images of fixed cells at baseline and following various experimental perturbations
Source: Data Brief. 2016 Jan 29;6:998–1006. doi: 10.1016/j.dib.2016.01.044 (PMC4760184; doi:10.1016/j.dib.2016.01.044)
Supplement: Supplementary file 14 — Supplementary material [file mmc14.zip › Scripts/Instructions.docx]

The Matlab code included here is split into several sub-functions. The sub-function *master* calls each of the other subscripts in order. The sub-function *analyze* launches a dialogue box through which the user can select a directory containing the microscope images to be analyzed and subsequently processes all images in the user defined directory. The sub-function *contrastall* performs binarization of raw microscope images as directed by the sub-function *analyze*. The sub-function *post_process* removes statistical outliers from the data set and calculates the mean and standard error of each parameter for each treatment group. The sub-function *export* exports both the raw Matlab database generated in the previous sub-functions and an excel spreadsheet of image-specific measurements in each parameter. The binarization schemes included in the sub-function *contrastall* were developed from previously published manuscripts [2,3]. The sub-function *analyze_beads* can be used to measure the apparent diameter of fluorescent beads on a blank background. If applied with similar microscope settings to those used in mitochondrial image acquisition to beads of known size, calibration of software measurements can be performed as described in the Mitochondrion paper.

**Instructions for use**

- Set the Matlab directory to the folder containing the mitochondrial analysis scripts
- Required image properties
  - All images must be in TIF format
  - All images must contain a nuclear stain in the blue channel, a mitochondrial stain in the green channel, and a cytoplasmic stain in the green channel
  - Images must be named according to the scheme “EX-GY-Z” where X is the experimental repetition (i.e. if this is the first time the experiment has been run, substitute 1 for X, 2 for the second time, etc), Y is the treatment group number (note that consecutive integers starting with 1 must be used for treatment group numbers), and Z is a specific sample identifying number within the treatment group (optional).
  - Images must be square (i.e. 1024x1024, 512x512, etc).
  - All images must have the same resolution, pixel size, and staining properties.
- User inputs
  - The parameter *blur* in the sub-function *analyze* refers to the degree of overestimation of dimensions determined using the bead calibration technique described in the associated manuscript [1]. If bead calibration has not been employed, use *blur=0.* When performing bead calibration, *blur* is not used.
  - The parameter *image_size* in the sub-function *analyze* refers to the size of each image. For example, if 1024x1024 images are used, define *image_size=1024.*
  - The parameter *res* in the sub-function *analyze* refers to the edge length of a single pixel. This parameter can typically be determined from settings within a microscope’s user interface.
  - NOTE: the parameters *image_size* and *res* must also be set in the sub-function *analyze_beads* if bead calibration is desired.
- How to call the program
  - After setting all user inputs to their desired values, clear the Matlab command prompt and type *master* (if analyzing mitochondrial images ) or *analyze_beads* (if performing bead calibration).
    - If performing bead calibration, subtract the known diameter published by the manufacturer from the average diameter recorded by the program.
  - Select the directory containing the images to be analyzed.
  - The program will provide you will periodic updates as to its progress on the command prompt. A typical run time is ~20s per image at 1024x1024 resoution.
- Program outputs
  - A directory is created for processed images. The processed images will display the original microscope image side-by-side with the binarized image for every image in the original directory.
  - A directory is created for color-corrected images. The corrected images are gamma-adjusted to maximize visibility of mitochondrial network and cytoplasmic features. These images may be preferable for presentation compared to the raw microscope images, since image properties are made more consistent between images following color correction. Do NOT use these images for any quantitative analysis.
  - A directory is created for exported data. This will include an excel spreadsheet and a Matlab database.
    - In the Matlab database, the program outputs are included under the variable *data*. Image specific measurements are included under *data.cell*. Image specific measurements excluding statistical outliers are included under *data.groomed.* Treatment group means are included under *data.means.* Treatment group SEMs are included under *data.ses.* Experimental repetition-specific means and SEMs are included under *data.E1means, data.E1ses, data.E2means, data*.*E2ses,* etc.
    - In the excel spreadsheet, the following column headers are included:
      - “group”- contains the treatment group number for each image
      - “experiment”- contains the experimental repetition number for each image
      - “vol”- contains the number of illuminated pixels in the binarized cytoplasm image (a metric of cell size)
      - “number_of_networks”- contains the number of individual bodies detected in the mitochondrial network
      - “num_nuc”- contains the number of nuclei detected
      - “quality”- contains the fraction of total mitochondrial mass detected suitably for morphological analysis
      - “cell”- contains the sequence in which the images were imported by the software
      - “SNR”- contains the measured signal-noise ratio for each image
      - “mean_cell_size”- contains the average number of illuminated pixels in the cytoplasm channel per nuclear body detected
      - “mean_length”- contains the average length of a mitochondrial body in the image
      - “mean_width”- contains the average width of a mitochondrial body in the image
      - “mean_mito_size”- contains the average projected are of a mitochondrial body in the image
      - “mean_edges”- contains the average projected perimeter of a mitochondrial body in the image
      - “fill_frac”- contains the fraction of the cytoplasm filled by mitochondria
      - “perinuclear_preference”- describes the localization of mitochondria relative to the nucleus (see associated manuscript for details)
  - NOTE: the study authors strongly recommend visually comparing raw and binarized images to safeguard against detection errors. In the event of difficulty implementing this software, please do not hesitate the study authors for assistance.
